# Decoupling of maternal and neonatal inflammatory levels at the maternal-fetal interface: evidence from a population-based proteomic study

**DOI:** 10.3389/fimmu.2026.1715880

**Published:** 2026-01-30

**Authors:** Floriana Milazzo, Frederieke Gigase, Anna Suleri, Bushra Amreen, Darwin D’Souza, Seunghee Kim-Schulze, Veerle Bergink, Lot de Witte, Corina Lesseur, Anna-Sophie Rommel

**Affiliations:** 1 Department of Psychiatry, Icahn School of Medicine at Mount Sinai, New York, NY, United States; 2 Department of Child and Adolescent Psychology and Psychiatry, Erasmus Medical Center, Rotterdam, Netherlands; 3 Department of Environmental Medicine, Icahn School of Medicine at Mount Sinai, New York, NY, United States; 4 Human Immune Monitoring Center, Icahn School of Medicine at Mount Sinai, New York, NY, United States; 5 Department of Immunology and Immunotherapy, Icahn School of Medicine at Mount Sinai, New York, NY, United States; 6 Department of Psychiatry, Erasmus Medical Center, Rotterdam, Netherlands; 7 Department of Psychiatry, Radboud University Medical Center, Nijmegen, Netherlands

**Keywords:** maternal immune activation, maternal-fetal immune regulation, neonatal inflammation, perinatal immune system, pregnancy

## Abstract

**Background:**

Maternal inflammation during pregnancy has been linked to offspring physical and neurodevelopmental health. This association has been hypothesized to operate through fetal immune responses. Yet, population-based evidence exploring the relationship between maternal and neonatal inflammation remains limited. This study investigates the associations of maternal inflammatory markers measured in the third trimester and at birth with neonatal inflammatory profiles at birth.

**Methods:**

Interleukin (IL)-1β, IL-6, IL-17a (pg/mL), and CRP (mg/L) levels were measured in maternal plasma at birth and in the third trimester and standardized for the at-birth and third-trimester subset. In the neonates, 92 inflammatory markers were measured in Dried Blood Spots using Olink^®^. We used linear regression models with empirical Bayes moderation to investigate the association between maternal inflammatory marker levels at each timepoint and neonatal inflammatory marker levels. We adjusted for maternal factors, child factors, and technical factors. We applied the false discovery rate (FDR-adjusted *p*-value <0.05) to adjust results for multiple comparisons.

**Results:**

Maternal IL-1β, IL-6, IL-17a, and CRP levels measured at birth (n=194 mother-child dyads) or in the third trimester (n=235) were not significantly associated with levels of any of the 92 neonatal inflammatory markers. Effect estimates were small, and no associations survived FDR correction (FDR <0.05). Findings were observed with robust parsimonious adjustment for covariates and were similar across both maternal sampling timepoints.

**Conclusion:**

Maternal inflammation measured at birth and in the third trimester was not significantly associated with widespread changes in neonatal peripheral inflammatory profiles. These findings suggest that low-grade maternal inflammation may not elicit detectable systemic inflammatory responses in neonates at birth in a general population sample. Future research should replicate our findings and assess the role of neonatal inflammatory markers in subsequent offspring health outcomes.

## 1 Introduction

The fetal immune system develops in synergy with maternal immune modulation (1, 2), which enables tolerance and support of the semi-allogeneic fetus (2, 3). The innate immune cells (e.g., macrophages, mast cells, NK cells, microglia) emerge around four weeks’ gestation, followed by the hematopoietic stem cells migration to the fetal liver and then bone marrow (4). Adaptive immunity matures mid-gestation, with T-cell development in the thymus and B-cell maturation and immunoglobulin diversification predominantly taking place in the bone marrow (4).

The maternal immune system shapes fetal immune maturation through placental transfer of IgG antibodies, cytokines, and hormones (5, 6). The bidirectional signaling between mother and fetus provides both passive immunity and primes fetal immune responses before birth (5–7). By the third trimester, the placenta has matured into a thin syncytiotrophoblast layer to facilitate maternal-fetal exchange (8), coinciding with a time of rapid fetal brain development and synaptic pruning (9). These dynamic shifts in maternal inflammatory adaptation and fetal immune response suggest that the third trimester and labor may be sensitive windows for fetal immune programming and neurodevelopment. Maternal perturbations during these periods may have differential and long-lasting effects on offspring development depending on their timing.

Both maternal and neonatal inflammation have been implicated in adverse developmental outcomes in the child across the life course. Prenatal risk factors such as psychosocial stress, obesity, psychiatric conditions, infections and environmental exposures can influence the intrauterine environment by promoting maternal inflammatory and immune responses (10–13). Evidence from animal research demonstrates that acute and chronic maternal inflammation may be transduced to the fetus both via active fetal cytokine production and passive transfer of maternal cytokines (14). These processes upregulate inflammatory pathways in the placenta and fetal circulation (2, 15–20). Depending on their severity and timing, such increases in fetal inflammation may disrupt fetal brain development, impair nutrient and oxygen delivery, and hinder the growth and maturation of other organ systems (16, 21, 22). Accordingly, fetal immune dysregulation is hypothesized to be a key mechanistic link between maternal inflammation and long-term physical and neurodevelopmental outcomes in offspring.

The association between maternal inflammation and neonatal inflammation in human pregnancies remains poorly understood, with research yielding mixed results. Some studies have found positive associations of maternal inflammatory marker levels (e.g., Interleukin-6, IL-6; Interleukin-8, IL-8; Tumor Necrosis Factor alpha, TNF-a) in the second and third trimester with child inflammatory markers at birth (2) or in infancy (23, 24). Yet, population-based studies investigating the relationship between maternal CRP levels during pregnancy and a DNA methylation proxy for neonatal inflammation have reported no association (25, 26). A few studies have examined inflammatory markers in amniotic fluid as a proxy for fetal inflammation. These studies found weak or null associations between individual inflammatory markers in maternal serum and markers in amniotic fluid, both among women of advanced maternal age undergoing selective amniocentesis (27) and among women with cases of premature rupture of membranes without intra-uterine infection (28). However, these studies had small sample sizes and focused on specific clinical populations. Robust examination of the interplay between the maternal and neonatal immune system in humans has, in large part, been hampered by practical, ethical, and methodological challenges related to obtaining blood samples from neonates.

In this pre-registered study, we aimed to comprehensively examine the associations of maternal inflammatory markers in the peripartum period. To do so, we leveraged data from the prospective population-based Generation C (Gen C) cohort based at the Icahn School of Medicine at Mount Sinai in New York City. Specifically, we examined how maternal Interleukin-1-beta (IL-1β), Interleukin-6 (IL-6), and Interleukin-17A (IL-17a), and High-sensitivity C-reactive protein (CRP) inflammatory marker levels are associated with the expression of 92 neonatal inflammatory markers at birth. Our primary analysis focused on maternal inflammation measured at birth, given its close temporal proximity to neonatal marker collection. As a secondary analysis, we examined maternal inflammation during the third trimester.

## 2 Materials and methods

### 2.1 Deviations from the pre-registration

This analysis was pre-registered on (https://osf.io/j5ft). A few notable deviations were made from the approach outlined in the pre-registration as data analysis progressed.

Firstly, we adjusted our primary analytical approach, treating maternal inflammatory markers as continuous log-transformed predictors rather than binarizing maternal inflammation into high *vs*. low categories for each marker based on the 75^th^ percentile. This shift aligns with Olink^®^ practices, preserves more information, and avoids imposing an arbitrary threshold that reduces statistical power. However, to maintain consistency with the pre-registration, the binarized treatment of maternal inflammatory marker predictors was retained as a sensitivity analysis. Second, we used a more parsimonious model, including fewer covariates than initially considered, to maximize power. Thus, we did not include maternal age, pre-pregnancy BMI, education, maternal psychopathology, parity, prenatal SARS-CoV-2 exposure, tobacco use, child sex, and cardiometabolic disorders of pregnancy as covariates in the primary analyses. Third, the sensitivity analysis excluding those with prenatal exposure to SARS-CoV-2 was not performed since the number of SARS-CoV-2 positive individuals was too small in the primary (n=12) and secondary (n=31) analysis. Lastly, neonates admitted to the NICU were removed from analyses given their distinct inflammatory profiles and heterogenous clinical reasons for NICU admissions.

### 2.2 Study population

Generation C is a prospective pregnancy cohort conducted within the Mount Sinai Health System (MSHS), a large health care system in New York City (NYC). The study is described in detail elsewhere (29). Briefly, pregnant individuals (≥18 years old) receiving obstetrical care in the MSHS were eligible for participation. Recruitment began in April 2020 and data collection concluded in October 2022. All participants provided informed consent. Patient demographic and clinical data, as well as pregnancy outcomes, were extracted from a combination of survey data and electronic medical records (EMR). The study was approved by the Icahn School of Medicine at Mount Sinai Institutional Review Board (IRB-20-03352), reviewed by the US Centers for Disease Control and Prevention (CDC), and conducted in accordance with applicable federal law, CDC policy and the Declaration of Helsinki.

A total of 3,157 participants were included in the Generation C study. Here, we included those who: 1) delivered between April 2020 and October 2022, 2) had at least one maternal inflammatory marker measured in blood samples collected at birth and/or during the third trimester, and 3) provided informed consent to neonatal dried blood spot collection. Two exclusion criteria were applied. First, individuals whose children were admitted to the Neonatal Intensive Care Unit (NICU) were excluded because neonates admitted to the NICU have distinct inflammatory profiles (30). Second, subjects whose Olink^®^ neonatal inflammatory marker data met an outlier criterion indicative of processing errors (i.e., >5 standard deviations from the mean in a sample-level PCA) were excluded, as confirmed during the quality control process described below.

### 2.3 Maternal inflammatory markers

Blood specimens were obtained as part of routine blood draws (an additional 4cc EDTA tube) during scheduled prenatal visits or on admission to labor and delivery (29). Plasma aliquots were centrifuged, aliquoted into 500 μl vials and stored at −80 °C until further analysis.

A Principal Component Analysis (PCA) was performed in a subset of 200 Gen C participants. Based on a hierarchical clustering analysis described elsewhere (31), we selected IL-1β, IL-6, and IL-17a, to be measured in the entire sample, along with high sensitivity C-reactive protein (CRP), an established marker of general inflammation.

Maternal inflammatory markers (IL-1β, IL-6, IL-17a, and CRP) measured in plasma at various timepoints in pregnancy using the High Sensitivity T-cell Discovery Array 3-Plex (Millipore, St. Charles, MO, USA) were analyzed at Eve Technologies using the Bio-Plex™ 200 system (Bio-Rad Laboratories, Inc., Hercules, CA, USA).

For analyses, we defined two exposure windows for maternal inflammation that corresponded to our primary and secondary analyses: 1) an ‘at-birth’ sample consisting of maternal blood samples collected within 3 days of delivery constituted our primary analysis, and 2) the ‘third-trimester’ sample, consisting of blood samples collected at ≥28 gestational weeks and more than 7 days before delivery, which constituted our secondary analysis.

### 2.4 Neonatal inflammatory markers

We obtained residual Dried Blood Spots (DBS) of the Generation C children from the New York State Department of Health Newborn Screening Program (NYSDOH NBS). DBS offer a cost-effective, reliable approach to characterize neonatal inflammatory profiles (32).

In New York State, mandatory DBS are collected on standard Whatman 903 specimen cards within the first ~24–36 hours of life and stored at room temperature (21 °C) for up to 27 years. One 3 mm punch of filter paper (~50 ul of blood) was analyzed with the Olink^®^ Target 96 Inflammation Proteomics platform (Olink^®^ Bioscience, Uppsala, Sweden). This panel measures 92 inflammatory marker levels using a highly sensitive and specific proximity extension assay technique and real-time PCR to measure relative changes in protein expression (33). The neonatal inflammatory marker values are expressed as normalized protein expression (NPX values) on a log2 scale (33). Oligonucleotide-labeled antibody pairs, each targeting a specific protein, were added to samples. Upon dual epitope binding, the antibodies formed a double-stranded oligonucleotide template for PCR amplification. Amplicons were generated in 96-well plates using an extension master mix and quantified on the Biomark HD System (Fluidigm, South San Francisco, CA, USA).

To ensure consistency across plates, we applied reference-sample-based normalization using Plate 1 as the anchor. Five reference samples were used to calculate per-analyte, per-plate normalization factors based on signal deviations from Plate 1. The median of these five values was applied to normalize each analyte on non-anchor plates. Quality control (QC) included internal controls on every plate to monitor immunoreaction, extension, and amplification/detection stages of the Olink^®^ protocol. In addition, a sample-level PCA with the Olink^®^ inflammatory neonatal marker data was run to check the data for extreme outliers (5 or more standard deviations from the mean). Two participants met this outlier criterion and were excluded because their values suggested neonatal sample processing errors as confirmed by QC.

### 2.5 Covariates

Neonatal covariates included gestational age at birth (in days), length of hospital stay for the child (in days), and birthweight (in grams).

A PCA was performed using the “prcomp” function in R to reduce the multicollinearity and dimensions of these neonatal variables. Based on the resulting scree plot and variance explained, the first two principal components, which accounted for 80% of the variance in the data, were retained and included as two covariates.

Along with the first two principal components of the neonatal factors, the following technical and biological factors were included as individual covariates in analyses at both timepoints (birth, third trimester): maternal inflammatory marker batch (categorical), child age at DBS collection (in hours), and time between maternal sample date and child birthdate (in days).

Our primary models did not adjust for maternal sociodemographic and clinical characteristics (e.g., age, race and ethnicity, parity, cardiometabolic pregnancy complications, pre-pregnancy BMI) because these factors can directly influence maternal inflammatory levels, the primary exposure of interest (10, 34, 35). Adjusting for these upstream maternal factors may result in overcorrection and potentially obscure our ability to isolate and detect our effect of interest (maternal inflammation on neonatal markers).

Data on all covariates were collected through self-reported questionnaires at enrollment and at follow-ups and/or extracted from the Electronic Medical Record (EMR).

### 2.6 Statistical analysis

All analyses were conducted using R, version 4.1.3.

#### 2.6.1 Maternal inflammatory markers

Each maternal inflammatory marker (IL-1β, IL-6, IL-17a, and CRP) was modeled as a distinct continuous exposure. Maternal inflammatory markers were first log2-transformed given their skewed distribution and subsequently standardized (z-scored), so that effect estimates reflect a one standard-deviation increase in the maternal marker.

For our primary analysis, the exposure was maternal inflammation measured at birth, defined as blood samples taken within 3 days of delivery. This window offered the largest available sample of individuals with specimens taken at the exact same time (i.e., birth) while still accounting for variability in admission to labor and delivery and sample pick-up times. This window also maintains close temporal proximity to the neonatal inflammation measurements, which are particularly important given the short half-life of inflammatory markers.

In cases with multiple maternal inflammatory marker measures, the sample with the date closest to the child’s birthdate was analyzed (n=1). If multiple samples were taken on the date closest to the child’s birthdate (n=2), log2-transformed inflammatory marker values were averaged.

As a secondary exposure, we examined maternal inflammation in the third trimester (gestational age at sample ≥ 28 weeks). To best capture pregnancy-related maternal inflammation rather than inflammation associated with labor and delivery, we excluded samples collected within 7 days before the child’s birthdate. For participants with multiple eligible third trimester samples (n=19), the sample with the earliest date was chosen.

Values below the limit of detection (LOD) were replaced with the lowest detectable value for IL-1β (0.02 pg/mL) and/or IL-6 (0.01 pg/mL). In this sample, no values fell below the LOD for IL-17a (0.6 pg/mL) or CRP (196.69 mg/L) (**Supplementary Table 1**).

#### 2.6.2 Descriptive statistics

For descriptive purposes, we summarized sociodemographic and clinical characteristics using medians with interquartile ranges (IQR) and frequencies with percentages, comparing participants in the top 25% of maternal inflammation levels with those in the lower 75% for each maternal inflammatory marker value. We assessed differences between these groups using Wilcoxon rank, chi-squared, or Fisher’s exact tests where appropriate. **Table 1** displays a full comparison of all demographic variables by maternal inflammation groups for the at-birth sample.

**Table 1:** Maternal and child demographic characteristics among participants with maternal inflammation measured at birth (primary analysis) by inflammation levels.

Characteristic^†^	Overall	IL-1β at birth		IL-6 at birth		IL-17a at birth		CRP at birth	
	N=194^1^	High (N = 49^1^)	Low (N = 145^1^)	High (N = 49^1^)	Low (N = 145^1^)	High (N = 49^1^)	Low (N = 145^1^)	High (N = 48^1^)	Low (N = 145^1^)
**Maternal age at delivery (years)**	33 (30, 36)	33 (30, 36)	33 (30, 36)	33 (30, 36)	33 (30, 36)	32 (29, 35)	33 (30, 36)	33 (30, 36)	33 (30, 36)
**Race and ethnicity**								***	
Asian	12% (24)	16% (8)	11% (16)	24% (12)	8% (12)	16% (8)	11% (16)	13% (6)	12% (18)
Black	11% (22)	8% (4)	12% (18)	8% (4)	13% (18)	8% (4)	12% (18)	13% (6)	11% (16)
Hispanic	23% (45)	20% (10)	24% (35)	20% (10)	24% (35)	27% (13)	22% (32)	35% (17)	19% (27)
White	48% (94)	49% (24)	48% (70)	43% (21)	50% (73)	43% (21)	50% (73)	27% (13)	56% (81)
Other	5% (9)	6% (3)	4% (6)	4% (2)	5% (7)	6% (3)	4% (6)	13% (6)	2% (3)
**Parity**		**							
Nulliparous	47% (91)	65% (32)	41% (59)	41% (20)	49% (71)	57% (28)	43% (63)	40% (19)	49% (71)
Multiparous	53% (103)	35% (17)	59% (86)	59% (29)	51% (74)	43% (21)	57% (82)	60% (29)	51% (74)
**Education**								**	
<College	23% (36)	17% (8)	25% (28)	18% (8)	24% (28)	24% (11)	22% (25)	38% (15)	18% (21)
≥College	78% (124)	83% (39)	75% (85)	82% (36)	76% (88)	76% (35)	78% (89)	62% (24)	83% (99)
Missing	34	2	32	5	29	3	31	9	25
**Child sex**						**			
Female	51% (98)	49% (24)	51% (74)	45% (22)	52% (76)	69% (34)	44% (64)	58% (28)	48% (70)
Male	49% (96)	51% (25)	49% (71)	55% (27)	48% (69)	31% (15)	56% (81)	42% (20)	52% (75)
**Maternal history of mental illness**		***		*					
No	58% (113)	82% (40)	50% (73)	73% (36)	53% (77)	63% (31)	57% (82)	50% (24)	61% (89)
Yes	42% (81)	18% (9)	50% (72)	27% (13)	47% (68)	37% (18)	43% (63)	50% (24)	39% (56)
**SARS-CoV-2 infection during pregnancy**		*		*					
No	94% (182)	100% (49)	92% (133)	100% (49)	92% (133)	98% (48)	92% (134)	92% (44)	94% (137)
Yes	6% (12)	0% (0)	8% (12)	0% (0)	8% (12)	2% (1)	8% (11)	8% (4)	6% (8)
**Child age in hrs at DBS collection**	25 (24, 29)	24 (24, 28)	25 (24, 29)	25 (24, 29)	25 (24, 28)	25 (24, 29)	25 (24, 28)	25 (24, 30)	25 (24, 28)
**Pre-preg BMI (kg/m^2^)**								***	
	25 (22, 29)	26 (23, 29)	25 (21, 29)	26 (22, 29)	25 (22, 29)	26 (23, 28)	25 (21, 29)	28 (25, 36)	24 (21, 28)
Missing	1	0	1	0	1	0	1	0	1
**Delivery Mode**		**							
C-section	47% (91)	29% (14)	53% (77)	43% (21)	48% (70)	53% (26)	45% (65)	46% (22)	48% (69)
Vaginal	53% (103)	71% (35)	47% (68)	57% (28)	52% (75)	47% (23)	55% (80)	54% (26)	52% (76)
Cardiometabolic disorders of pregnancy									
No	72% (139)	73% (36)	71% (103)	71% (35)	72% (104)	71% (35)	72% (104)	67% (32)	74% (107)
Yes	28% (55)	27% (13)	29% (42)	29% (14)	28% (41)	29% (14)	28% (41)	33% (16)	26% (38)
**GA at delivery** (days)								**	
	274 (270, 279)	276 (270, 280)	274 (270, 278)	275 (272, 280)	274 (269, 278)	274 (268, 280)	274 (271, 278)	273 (266, 276)	275 (271, 280)
Preterm Birth									
No	96% (186)	96% (47)	96% (139)	100% (49)	94% (137)	100% (49)	94% (137)	98% (43)	95% (143)
Yes	4% (8)	4% (2)	4% (6)	0% (0)	6% (8)	0% (0)	6% (8)	2% (1)	5% (7)
**BW (grams)**	3,230 (2,990, 3,630)	3,150 (2,980, 3,535)	3,240 (3,010, 3,660)	3,240 (3,040, 3,710)	3,220 (2,980, 3,605)	3,140 (2,973, 3,440)	3,250 (3,000, 3,680)	3,238 (3,030, 3,722)	3,225 (2,990, 3,600)

^1^Median (IQR); %(n). Note: percentages may not sum to 100% due to rounding.

^†^*p* < 0.05 (*), p < 0.01 (**), p < 0.001 (***). *p*-values are based on Wilcoxon rank-sum tests for continuous variables and Chi-square or Fisher’s exact tests for categorical variables, comparing high *vs*. low groups within each maternal inflammatory marker.

BW, birthweight; GA, gestational age; hrs, hours; pre-preg, pre-pregnancy.

#### 2.6.3 Differential expression regression analysis

For our primary and secondary analyses, the limma package in R (36) was used to identify associations between maternal inflammatory marker levels (IL-1β, IL-6, IL-17a, CRP) and 92 neonatal inflammatory marker levels via linear regression models. The limma package is well-suited for high-dimensional inflammatory data because it utilizes an empirical Bayes approach to moderate the standard errors of residual variances (37). All models were adjusted for maternal inflammatory marker batch, child age at DBS collection, time between maternal sample date and child birthdate, and the first two principal components (PC1 and PC2) of the aforementioned collinear neonatal covariates (see 2.5 Covariates).

To correct for multiple testing, we applied the False Discovery Rate (FDR) method. An adjusted *p*-value of <0.05 was considered significant. For each association, we report the estimated regression coefficient (β) and corresponding confidence interval, representing the change in neonatal marker expression per one standard-deviation increase in the maternal log2-transformed marker value.

#### 2.6.4 Sensitivity analyses

We conducted three sensitivity analyses.

**Figure 1:**
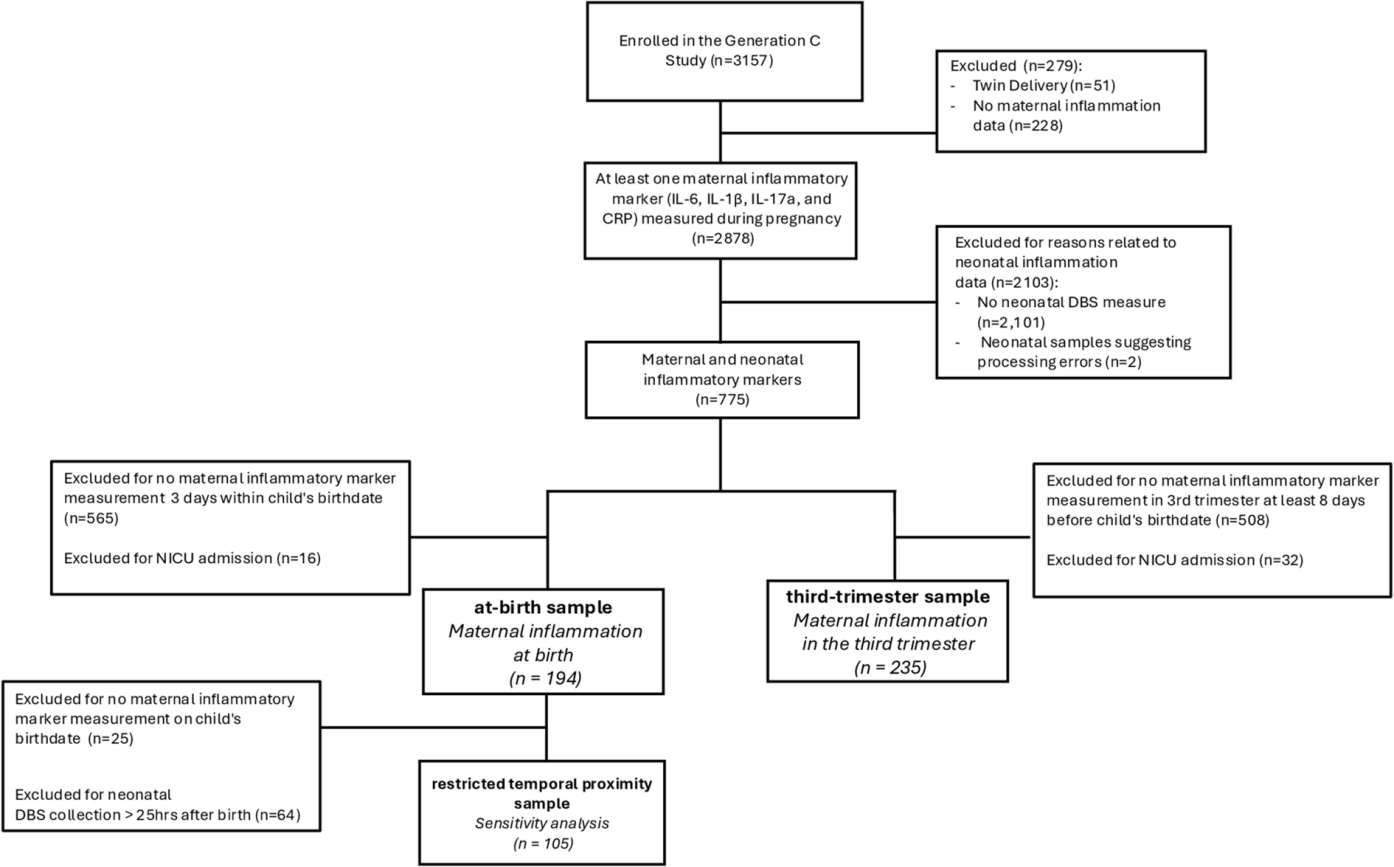
Flowchart of at-birth, third-trimester, and restricted temporal proximity study samples.

The first analysis applied the same differential expression methods as described above but in a reduced subset of the at-birth sample. For this analysis, we restricted the cohort to a subset of 105 mother-child dyads for whom maternal samples were collected on the child’s birthdate and neonatal DBS collection occurred within 25 hours of birth (the median collection time in the primary analysis). This sensitivity analysis was designed to capture rapid immune changes around delivery and maximize sample size, allowing us to assess the impact of temporal proximity between maternal plasma sample collection and neonatal DBS collection on the relationship between maternal and neonatal inflammation. This subset is referred to as the ‘restricted temporal proximity’ sample. **Figure 1** denotes inclusion criteria for this subset. Maternal inflammation at birth remained a continuous exposure. We included the following covariates: child age at DBS collection (hrs) and the first two principal components of the collinear neonatal covariates as described above.

Second, for each analysis we ran, we also conducted a sensitivity analysis in which we expanded the parsimonious primary model to include additional maternal clinical and demographic characteristics that may influence both maternal and neonatal inflammation. Specifically, these sensitivity models further adjusted for maternal age at delivery, pre-pregnancy BMI, and race and ethnicity.

Third, in line with our pre-registration, we conducted sensitivity analyses examining maternal inflammation at birth as a binary exposure to ensure transparency and comparability with the original analytic plan. For this analysis, maternal inflammatory markers at birth (IL-1β, IL-6, IL-17a, CRP) were categorized into “low” (≤75th percentile) and “high” (>75th percentile) maternal inflammation groups based on their log2-transformed values. Each inflammatory marker was treated as a distinct exposure. Hereafter, for this sensitivity analysis, participants with IL-1β, IL-6, IL-17a, and CRP levels above the 75^th^ percentile are referred to as having “high maternal [marker] levels” (e.g., high maternal IL-1β). Those at or below the 75^th^ percentile are referred to as having “low maternal [marker] levels.”

### 2.7 Power analyses

Power calculations were conducted *post hoc* relative to the pre-registration using the “pwr” package to estimate minimum detectable effect sizes for models examining associations between continuous maternal inflammatory markers and neonatal inflammatory marker expression, as well as for the pre-registered high versus low maternal inflammation comparisons. Assuming a two-sided alpha of 0.05 and 80% power, the minimum detectable partial R^2^ ranged from approximately 0.03 to 0.04 for the third-trimester and at-birth samples. These values correspond to standardized regression effects of approximately 0.17-0.20 NPX change per 1 standard deviation increase in maternal inflammation. For the group-based analyses, minimal detectable Cohen’s d values ranged from 0.42 to 0.48 for the third-trimester and at-birth samples. Minimum detectable Cohen’s d values were calculated following limma analyses using the “CohenD” function in the DescTools package. Both partial R² and Cohen’s d estimates are provided as a standardized reference for interpretability. However, they do not account for covariate adjustment and FDR correction applied in limma. A complete summary of corresponding minimum detectable effect sizes for every analysis can be found in **Supplementary Table 2**.

## 3 Results

### 3.1 At-birth and third-trimester analytical samples

After applying our inclusion and exclusion criteria, the total analytical sample size for the at-birth analysis was n=194 mother-child dyads. The total analytical sample size for the third-trimester analysis was n=235 mother-child dyads. There was an overlap of n=37 participants that met the inclusion criteria for both the at-birth and third-trimester samples. A flowchart of study inclusion and exclusion for each analysis is presented in **Figure 1**.

### 3.2 Sociodemographic characteristics and maternal inflammation categories

In the at-birth sample, maternal and neonatal sociodemographic and clinical characteristics stratified by maternal inflammation groups are shown in **Table 1**. Of the 194 individuals, the median maternal age at delivery was 33 (IQR: 30-36) years. Slightly less than half of the sample identified as white (48%, n=94). Just under a third of the sample (28%, n=55) had a cardiometabolic disorder of pregnancy and 42% (n=81) had a history of psychopathology. The majority were highly educated with 78% (n=124) having a bachelor’s degree or higher.

History of psychopathology was less prevalent among participants with high compared to low maternal IL-1β (18% *vs*. 50%, *p* < 0.001) and high compared to low maternal IL-6 (27% *vs*. 47%, *p* = 0.022). SARS-CoV-2 infection at any point during pregnancy was less common in the high maternal IL-1β (0% *vs*. 8%, *p* = 0.042) and IL-6 (0% *vs*. 8%, *p* = 0.039) groups compared to their respective low-expression groups. Participants with high compared to low maternal IL-1β were significantly more likely to undergo vaginal delivery (71% *vs.* 47%, *p* < 0.003) and be nulliparous (65% *vs*. 41%, *p* = 0.003).

Additionally, those in the high maternal IL-17a group were more likely to give birth to female babies compared to the low IL-17a group (69% *vs*. 44%, *p* = 0.002).

Furthermore, individuals with high maternal CRP were more likely to have lower education levels (38% *vs*. 18%, *p* = 0.007), a higher pre-pregnancy BMI (28 *vs*. 25 kg/m², *p* < 0.001), and lower gestational age at birth (273 *vs*. 275 days, *p* = 0.008) and were more likely to identify as Black, Hispanic, or “Other” (*p* < 0.001), compared to those in the low CRP group.

All demographic information for those in the at-birth subset can be found in **Table 1**. Corresponding maternal inflammatory marker information can be found in **Supplementary Table 3**.

In the third-trimester sample, high maternal CRP in the third trimester was significantly associated with lower education levels, a higher pre-pregnancy BMI, lower gestational age at birth, identifying as Black, Hispanic, or “Other,” and with prenatal SARS-CoV-2 infection. Additionally, low maternal IL-6 and low maternal CRP levels were significantly associated with higher maternal age. No other demographic variables were associated with third trimester inflammatory levels. See **Supplementary Tables 4** and **5** for additional demographic and maternal inflammatory information among this third-trimester subset.

### 3.3 Maternal inflammatory marker levels across samples

**Figure 2:**
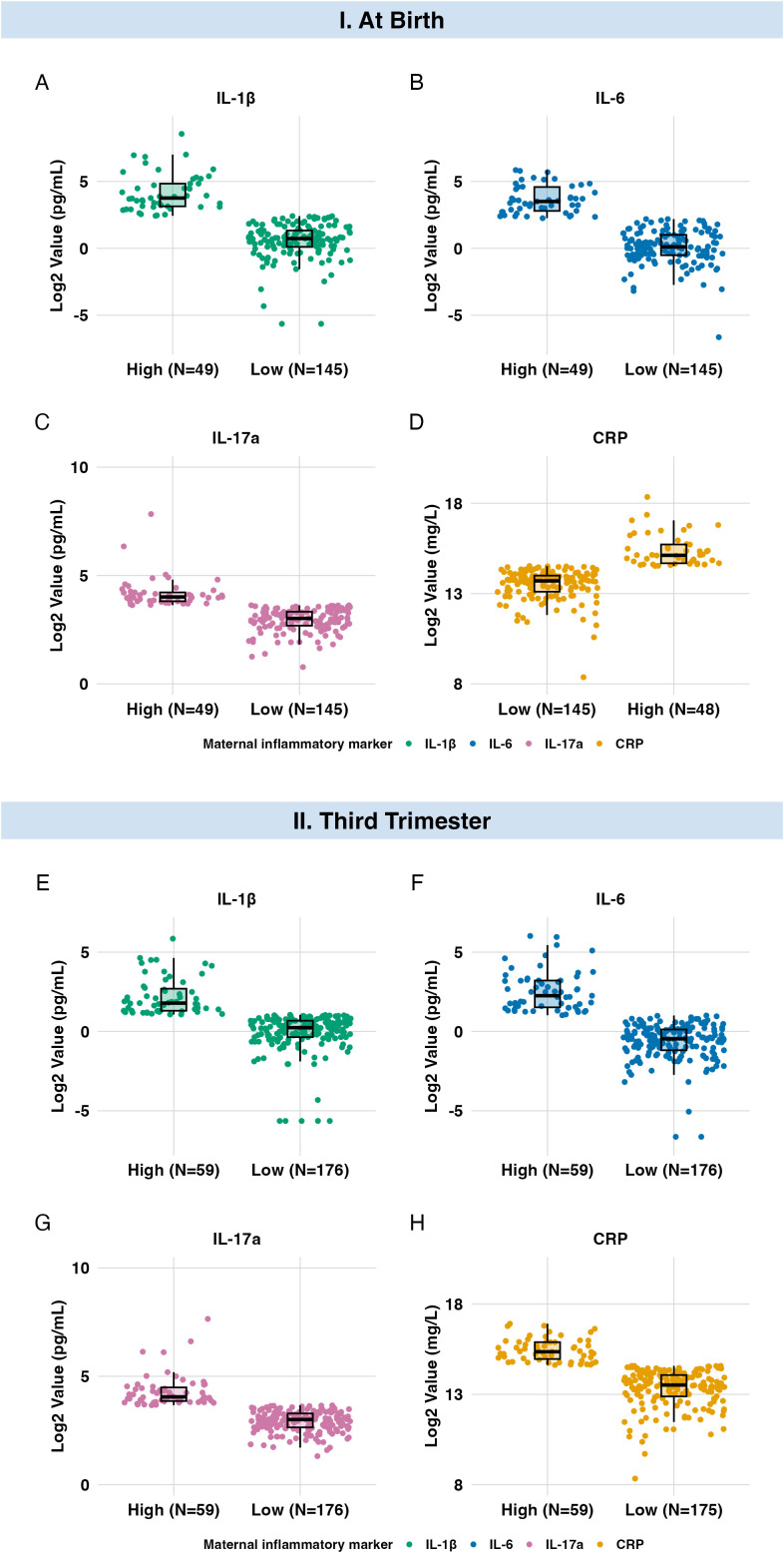
Box plots of high *vs*. low maternal inflammatory levels markers at birth and in the third trimester. Log2-transformed neonatal inflammatory marker levels are displayed in pg/mL for IL-1β, IL-6, and IL-17a, and mg/L for CRP (y-axis) stratified by maternal inflammatory status (x-axis: high *vs*. low) for each maternal inflammatory marker. **(A–D)** represent neonatal inflammatory marker distributions by maternal inflammation measured at birth (primary analysis; n=194). **(E–H)** represent distributions by maternal inflammation measured in the third trimester (secondary analysis; n=235). Each point represents a neonatal dried blood spot (DBS) sample. N values per group are noted along the x-axis. Marker-specific color coding is used: IL- 1β (green), IL-6 (blue), IL-17a (pink), and CRP (orange).

The levels of maternal inflammatory markers in plasma differed between the at-birth and third-trimester samples (**Figure 2**, **Table 2**). Median IL-1β and IL-6 levels were higher in the at-birth sample than in the third-trimester sample (*p* < 0.001). IL-17a and CRP medians did not significantly differ between the samples (*p* = 0.7). Median absolute concentrations of maternal inflammatory markers in the at-birth sample prior to log2-transformation were IL-1β = 2.12 (IQR: 1.26, 5.39) pg/mL; IL-6 = 1.55 (IQR: 0.78, 4.77) pg/mL; IL-17a = 9.68 (IQR: 7.04, 12.39) pg/mL; and CRP = 15.46 (IQR: 10.31, 23.54) mg/L. Full descriptives for the absolute concentrations of maternal inflammatory markers at birth and in the third trimester are presented in **Supplementary Table 1**.

**Table 2:** Median log2-transformed maternal inflammatory marker levels across the at-birth and third-trimester study samples.

Characteristic^†^	At-Birth (N = 194^1^)	Third trimester (N = 235^1^)	*p-*value^2^
**IL-1β log2**	1.08 (0.33, 2.43)	0.52 (-0.17, 1.07)	<0.001
**IL-6 log2**	0.63 (-0.36, 2.25)	-0.09 (-0.94, 1.02)	<0.001
**IL-17a log2**	3.28 (2.82, 3.63)	3.16 (2.75, 3.66)	0.7
**CRP log2**	13.92 (13.33, 14.52)	13.90 (13.21, 14.63)	0.7
Missing	1	1	

^†^Values for IL-1β, IL-6, and IL-17a are log2-transformed concentrations (pg/mL); CRP is reported in mg/L.

^1^Median (IQR).

^2^ Wilcoxon rank sum test.

### 3.4 Associations between maternal and neonatal inflammation

Our at-birth analysis examined associations between log2-transformed and z-scored maternal inflammatory marker levels measured at birth (n=194) and the expression of 92 neonatal inflammatory markers. Our third-trimester analysis examined the same associations using maternal inflammatory markers measured during the third trimester (n=235; median gestational age at blood draw=31.5 weeks, IQR: 28.7–36.1 weeks) (**Supplementary Table 3**). A distribution of the gestational age at maternal inflammatory marker measures for the third trimester subset is presented in **Supplementary Figure 1**.

**Figure 3:**
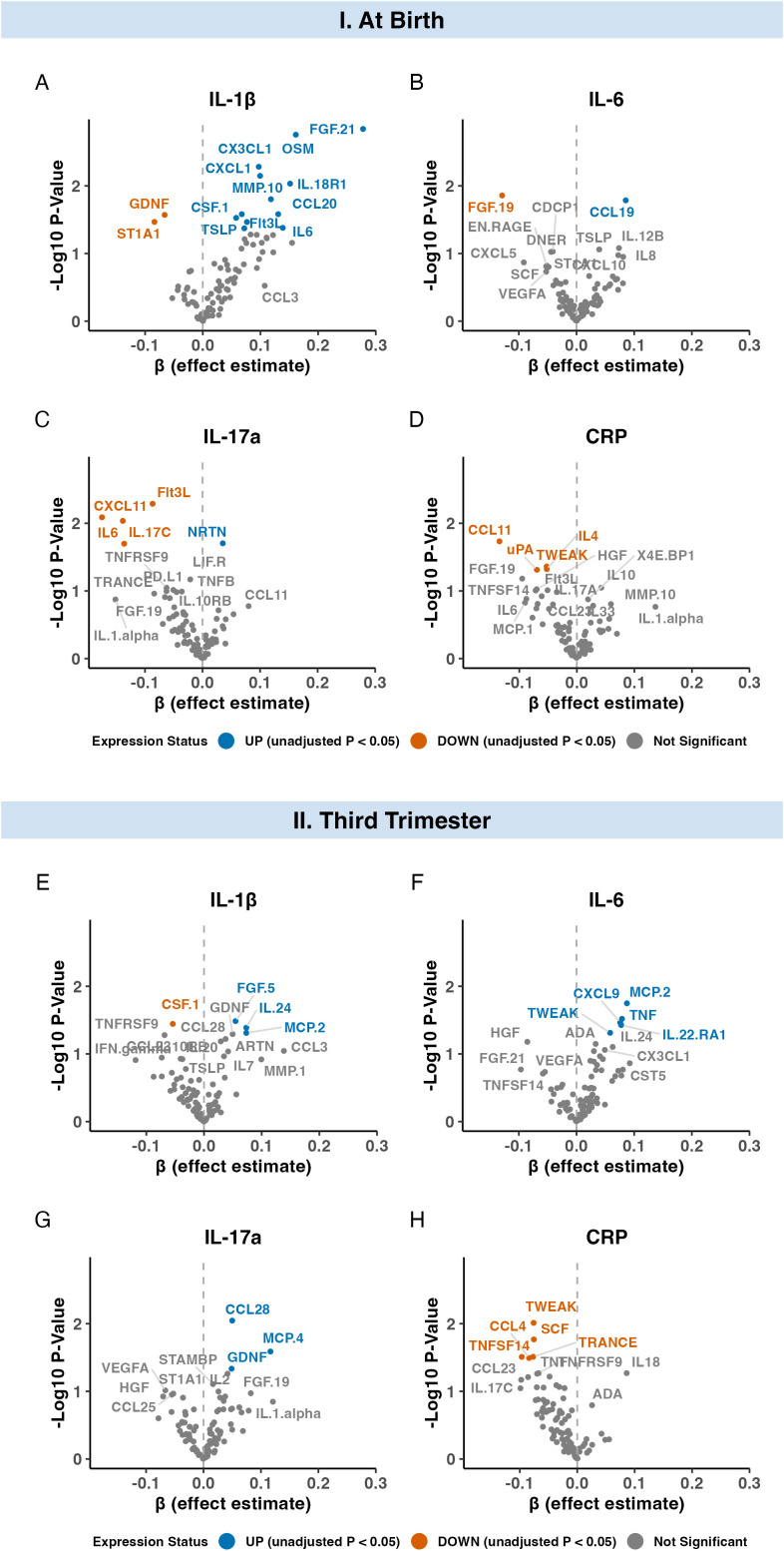
Volcano plots illustrating the associations between continuous maternal inflammation levels at birth (I) and during third trimester (II) and neonatal inflammatory marker expression for each maternal marker: IL-1β **(A, E)**, IL-6 **(B, F)**, IL-17a **(C, G)**, and CRP **(D, H)**. **(A–D)** correspond to the primary analysis of maternal inflammation measured at birth (n = 194); **(E–H)** correspond to the secondary analysis of maternal inflammation measured in the third trimester (n = 235). Each point corresponds to one neonatal inflammatory marker. Neonatal inflammatory markers are colored by statistical significance and the direction of the estimated effect size.

In the at-birth analysis, none of the 92 neonatal inflammatory markers were significantly associated with continuous maternal IL-1β, IL-6, IL-17a, or CRP levels after FDR correction (*p*-adjusted < 0.05 (**Figures 3A–D**). Similarly, in the third-trimester sample, none of the 92 neonatal inflammatory markers were significantly associated with continuous maternal IL-1β, IL-6, IL-17a, or CRP analyses after FDR correction (*p*-adjusted < 0.05) (**Figures 3E–H**). Volcano plots illustrating the estimated associations between each maternal and neonatal marker in the at-birth and third-trimester analysis are shown in **Figures 3A–H**. **Supplementary Tables 6**, **7** present the estimated regression coefficients (β) and confidence intervals for all neonatal inflammatory markers in relation to maternal inflammatory levels for the at-birth and third trimester samples respectively.

#### 3.4.1 Sensitivity analyses

The first sensitivity was conducted in a sample with restricted temporal proximity between maternal and neonatal inflammatory marker sampling. Demographic and maternal inflammatory marker information for the Restricted Temporal Proximity sample can be found in **Supplementary Tables 8** and **9**. Similar to the main analyses, there were no significant associations between continuous maternal and neonatal inflammatory markers in the restricted temporal proximity sample after adjusting for multiple testing correction (**Supplementary Figure 2A–D**). **Supplementary Table 10** displays the estimated regression coefficients (β) and confidence intervals for all neonatal inflammatory markers in relation to maternal inflammatory levels for this restricted temporal proximity subset. Across all analyses, these findings remained when adjusting for additional covariates (maternal age, pre-pregnancy BMI, and race and ethnicity). Volcano plots for these sensitivity analyses are shown in **Supplementary Figures 3** and **4**. **Supplementary Tables 11**–**13** present these adjusted estimated regression coefficients (β) and confidence intervals for all neonatal inflammatory markers in relation to maternal inflammatory levels for the at-birth, third trimester, and restricted temporal proximity samples, respectively.

Furthermore, results remained when maternal inflammation was binarized into high *vs*. low and treated as a categorical predictor in line with the pre-registration (**Supplementary Figures 5**, **6**; **Supplementary Tables 14**–**16**).

## 4 Discussion

This study is the first to comprehensively assess a broad proteomic panel of neonatal inflammatory markers in relation to maternal inflammation in a population-based pregnancy cohort. In this exploratory, prospective population-based study, we examined the association between maternal inflammatory markers IL-1β, IL-6, IL-17a (pg/mL) and CRP (mg/L) levels, measured in maternal plasma at birth and during the third trimester, and the expression of 92 inflammatory markers in neonates at birth. Maternal IL-1β, IL-6, IL-17a, and CRP levels at birth and during the third trimester were not strongly or consistently associated with normalized proteins expression (NPX) value levels of 92 neonatal inflammatory markers after multiple testing correction. IL-1β at birth, as well as IL-6 and CRP in the third trimester showed the greatest number of nominal associations with neonatal inflammatory markers, but these did not withstand multiple testing correction and should be interpreted cautiously. Across sensitivity analyses with reduced temporal sampling, additional adjustment for maternal factors, and binarization of high *vs.* low maternal inflammation, no association between maternal and neonatal inflammatory markers survived multiple testing correction. Overall, we found no evidence for strong links between maternal inflammatory markers levels and circulating inflammatory markers in the neonate. However, small-to-moderate associations cannot be ruled out.

Our findings contrast with previous work reporting positive associations between maternal and neonatal inflammation. In a smaller US study (n=87), Ross et al. (2016) (2) observed that higher maternal pro-inflammatory composite scores from the second and third trimester were associated with elevated cord blood cytokine levels at delivery (2). Similarly, Djuardi et al. (2016) reported positive associations between maternal inflammation and infant (at age 2 months) immune function in an Indonesian cohort (n= 146), including cord blood cytokine levels and a binary indicator for maternal parasitic infection during pregnancy (e.g., protozoans and/or helminths) (24). Several population and methodological factors may account for these discrepancies. First, timing and biospecimen source varied: Ross et al. (2016) (2) measured maternal inflammatory markers during pregnancy and neonatal cytokines in cord blood at delivery, while Djuardi et al. (2016) assessed maternal and infant cytokine responses after *in vitro* stimulation with phytohemagglutinin, reflecting maximal T-cell responsiveness rather than baseline *in utero* levels (24). By contrast, we analyzed maternal plasma at birth and in the third trimester and measured unstimulated neonatal inflammation in dried blood spots collected shortly after delivery. Second, the population context differed: the Indonesian cohort was characterized by higher infectious disease burden, nutritional deficiencies, and greater prevalence of neonatal sepsis, conditions that may amplify maternal–infant immune concordance (23, 24). Indeed, several associations attenuated after adjustment for maternal infection. Finally, the scope and definition of inflammatory markers varied: Ross et al. (2016) emphasized composite indices across a limited set of markers (2), and the Djuardi et al. (2016) focused largely on a select number of single markers such as IL-6 (24). In contrast, our comprehensive proteomic approach profiled 92 neonatal proteins with multiple-testing correction, providing insight into the potential regulation and relative stability of neonatal immune mechanisms.

Meanwhile, our results align with studies that have reported weak or absent associations between maternal, fetal, and neonatal inflammatory markers. Population-based studies have previously investigated associations between maternal circulating CRP and neonatal DNA methylation score of CRP, a proposed marker of persistent neonatal inflammation, and have generally observed only weak correlations (25, 26). Proposed explanations include the possibility that DNA methylation does not reliably index neonatal inflammatory status (26), that maternal CRP concentrations obtained at delivery are confounded by the inflammatory cascade of labor (25) and, as suggested here, that observed null associations reflect endogenous protective mechanisms (25, 26). Similarly, other work has reported that in the absence of severe maternal infections, maternal inflammatory markers in serum at mid-gestation (27) and during preterm premature rupture of membranes (28) do not correlate with corresponding inflammatory markers in amniotic fluid, a measure that more directly reflects fetal and intrauterine inflammatory processes. Consistent with these epigenetic reports, we identified no evidence of large effects across multiple maternal and neonatal inflammatory markers. Taken together, these findings provide convergent evidence that maternal low-grade inflammation during pregnancy may not directly translate into neonatal immune dysregulation, potentially reflecting the modulatory capacity of the placenta and fetus in buffering maternal inflammatory signals across gestation.

By examining a wide spectrum of maternal and neonatal inflammatory markers in a population-based cohort, this study adds nuance to the maternal immune activation literature, which has largely relied on animal models or high-risk clinical populations. Animal studies consistently show that experimentally induced maternal inflammation triggers a fetal immune activation response, alters immune cell activation, and leads to long-term developmental consequences, with maternal IL-6 identified as a central mediator (18, 38–40). Studies among women with acute chorioamnionitis (41) as well as those among a cohort of neonates born extremely preterm (<28 weeks) (42) similarly demonstrate that maternal immune activation is reflected in neonatal inflammation. These models and clinical studies suggest that severe or acute maternal inflammation can act as an “alarm” system, transmitting immune signals to the fetus and at the maternal-fetal interface to prime, train, and activate defense mechanisms should pathogens cross the placental barrier and infect the fetus (43). However, since severity of maternal inflammation and infection in these animal and high-risk cohorts is substantially higher than what is common in a healthy pregnancy, this transduction between maternal and fetal immune systems may only occur once a high threshold of inflammation is reached. Notably, the maternal inflammatory marker levels in our analytical samples predominantly reflect low-grade to moderately elevated inflammation rather than acute inflammatory states. For instance, CRP levels are known to be higher during pregnancy, and tend to increase across gestation, with typical values ranging from 3–10 mg/L (44). In obstetric contexts, CRP levels exceeding 10 mg/L may be interpreted as reflecting elevated, yet lower-grade, immune activation (44–46), whereas CRP levels associated with acute infections are typically substantially higher, commonly exceeding 20–25 mg/L (47). Accordingly, the median maternal CRP concentrations observed in our study (~15 mg/L at both timepoints) fall below ranges commonly reported for acute inflammatory states, while remaining within a spectrum of mild-to-moderate inflammatory activation in pregnancy. For cytokines such as IL-1β, IL-6, and IL-17A, clinically defined reference ranges in pregnancy are less well established. Nevertheless, the absolute concentrations observed in our study are, in general, markedly lower than levels reported in clinical samples characterized by pronounced inflammation, including pre-eclampsia, chorioamnionitis, and other severe placental or obstetric complications (46). Taken together, our at-birth and third-trimester results suggest that the predominantly low-to-moderate maternal inflammatory milieu observed in our general population sample may be insufficient, on average, to elicit a robust systemic fetal “alarm” response detectable in circulating neonatal inflammatory proteins at birth.

While our findings indicate no large-scale alterations in neonatal circulating inflammatory proteins, subtler processes, such as tissue-specific or local immune programming, trained immunity, or epigenetic changes, may occur beneath the detection limits of peripheral inflammatory protein measurements. Rather than indicating an absence of maternal-fetal immune communication, these findings are more consistent with active buffering and regulatory mechanisms operating during pregnancy that modulate how maternal inflammatory signals are transmitted to the fetus. From an evolutionary perspective, it may be maladaptive for every mild maternal inflammatory perturbation to provoke a widespread, durable systemic fetal immune response, given the increased susceptibility to infection during pregnancy. Under conditions of low-grade maternal inflammation, it may instead be advantageous for the fetus to remain buffered from maternal immune fluctuations, conserving energy for growth and development while avoiding unnecessary activation.

The lack of associations between maternal inflammation and neonatal inflammatory markers is also consistent with immune regulatory mechanisms operating at the placental interface, which are honed as the syncytiotrophoblast develops into a single continuous layer. As gestation progresses, compensatory placental or fetal immune mechanisms may allow the fetus to adapt to maternal inflammatory cues (48–50). In human pregnancies, such regulatory mechanisms include placental trophoblast receptors capable of detecting pathogens and triggering antimicrobial signaling that largely remains confined to the maternal side, thereby effectively protecting the fetus while activating maternal immune defenses. Simultaneously, increased selective transfer of maternal antibodies provides passive fetal immunity without provoking widespread activation (6, 8, 51). Therefore, these findings may underscore the functioning of these immune regulatory mechanisms in a healthy cohort, such that high maternal inflammation appears to be buffered by these placental immune mechanisms.

There are several strengths and limitations in this present study. Our study employed a large, diverse, prospective population-based cohort, a focus on a general pregnancy population, adjustment for critical covariates and a comprehensive assessment of neonatal inflammatory markers using advanced multiplex technology (Olink^®^). The consistency of our findings when treating maternal inflammation continuously and categorically supports the robustness of the observed associations. However, the limited number of sampling timepoints precludes characterization of longitudinal dynamics. Cumulative or chronic exposures of maternal inflammation, which are not captured by a single measurement of a maternal inflammatory marker, may exert more substantial effects on neonatal inflammatory profiles. Additionally, since neonates admitted to the NICU were not included in this cohort, the sickest infants were excluded from the analysis, potentially resulting in an underestimation of true immune coupling. Moreover, maternal and neonatal inflammation were measured across different assay platforms, which may limit comparability. Yet, Olink^®^ cytokine panels have demonstrated high consistency with other mid and low plex assays (52). Although our sample was larger than previous studies and the neonatal inflammatory marker measurement more extensive, the use of FDR correction inevitably increases the risk of Type II error, potentially obscuring more subtle associations (**Supplementary Table 2**). Lastly, while the overall effect sizes for the associations between maternal and neonatal inflammation remain relatively small, this does not preclude the possibility that these effects have meaningful clinical and biological implications, underscoring the need for further investigation.

## 5 Conclusion and future directions

In this large-scale, population-based study, we provide a novel and comprehensive assessment of the associations between maternal inflammation (IL-1β, IL-6, IL-17a, and CRP levels) and neonatal inflammatory profiles at birth. Reassuringly, maternal inflammation measured in an at-birth and third-trimester sample was not significantly or consistently associated with broad alterations in circulating neonatal inflammatory proteins. These results highlight the importance of distinguishing low-grade maternal inflammation from acute inflammatory activation when interpreting population-level studies of maternal-fetal immune relationships.

Whether the relative stability of neonatal inflammatory markers observed in this study reflects true immune resilience or limitations of single-timepoint exposure assessment remains uncertain. Future research incorporating a broader panel of maternal inflammatory markers repeated across pregnancy and trajectory-based or cumulative exposure metrics may be better positioned to capture biologically relevant inflammatory processes than isolated timepoints. Along with these approaches, targeted orthogonal validation via techniques such as Western blotting as well as correlation with cord-blood samples may bolster the biological interpretation of proteomic findings.

More broadly, future research should aim to disentangle the heterogeneous sources, timing, and sociodemographic contexts of maternal inflammation to better understand the pathways through which maternal immune signals may influence fetal development. Analyses that distinguish inflammatory responses arising from infection, chronic stress, and other acute or chronic immune conditions may clarify how maternal–neonatal immune concordance manifests across different contexts. Such efforts will be critical to determine whether specific immune pathways, cumulative inflammatory burden, severity thresholds, or sensitive developmental windows underlie the associations reported in animal models and select human studies linking maternal inflammation to altered brain and neurodevelopment, as well as increased risk for psychiatric conditions in offspring (9, 14, 16, 20).

Overall, our findings suggest that, in a general population sample, elevated maternal inflammation at single timepoints is unlikely to induce widespread neonatal immune dysregulation, although more subtle or context-specific effects cannot be excluded.

## Data Availability

The datasets presented in this study can be found in online repositories. The names of the repository/repositories and accession number(s) can be found below: https://osf.io/j5ft9/overview.
