# Supplementary material for: Decoupling of maternal and neonatal inflammatory levels at the maternal-fetal interface: evidence from a population-based proteomic study
Source: Front Immunol. 2026 Jan 30;17:1715880. doi: 10.3389/fimmu.2026.1715880 (PMC12907545; doi:10.3389/fimmu.2026.1715880)
Supplement: Supplementary file 1 [file DataSheet1.docx]

**SUPPLEMENTARY MATERIAL**

[**Supplementary Table 1.** Absolute maternal inflammatory marker level concentrations for maternal inflammation measured at birth and in the third trimester. 2](#_Toc216933978)

[**Supplementary Table 2**. Minimum detectable effect sizes (R^2^ and Cohen’s *d*) across analyses 2](#_Toc216933979)

[**Supplementary Table 3.** Maternal inflammatory marker characteristics for maternal inflammation measured at birth (primary analysis) by inflammation levels 3](#_Toc216933980)

[**Supplementary Table 4.** Maternal and child demographic characteristics for maternal inflammation measured in the third trimester (secondary analysis) by inflammation levels 4](#_Toc216933981)

[**Supplementary Table 5.** Maternal inflammatory marker characteristics for maternal inflammation measured in the third trimester (secondary analysis) by inflammation levels 5](#_Toc216933982)

[**Supplementary Figure 1.** Distribution of gestational age (weeks) at third-trimester maternal blood sampling. Histogram displays the gestational week at which maternal inflammatory markers were measured during the third trimester subset (N=235) 6](#_Toc216933983)

[**Supplementary Table 6.** Regression estimates for associations between continuous maternal inflammation levels measured at birth and neonatal inflammatory markers (n = 194), ordered by adjusted p-value 7](#_Toc216933984)

[**Supplementary Table 7.** Regression estimates for associations between continuous maternal inflammation levels measured in the third trimester and neonatal inflammatory markers (n = 235), ordered by adjusted p-value 16](#_Toc216933985)

[**Supplementary Table 8.** Maternal inflammatory marker characteristics in restricted temporal proximity by inflammation levels 25](#_Toc216933986)

[**Supplementary Table 9.** Maternal inflammatory marker characteristics in restricted temporal proximity analysis by inflammation levels 25](#_Toc216933987)

[**Supplementary Figure 2.** Volcano plots for differential expression of neonatal inflammatory markers relative to maternal inflammation levels at birth in first sensitivity analysis comparing high vs. low maternal inflammation groups for each maternal marker: IL-1β (A), IL-6 (B), IL-17a (C), and CRP (D) in a subset of n=105 with maternal inflammatory marker and neonatal DBS collected with restricted temporal proximity. 27](#_Toc216933988)

[**Supplementary Table 10.** Regression estimates for associations between continuous maternal inflammation levels measured among the restricted temporal proximity subset (n = 105), ordered by adjusted p-value 28](#_Toc216933989)

[**Supplementary Figure 3**. Volcano plots illustrating the associations between continuous maternal inflammation levels at birth (I) and during third trimester (II) and neonatal inflammatory marker expression for each maternal marker: IL-1β (A, E), IL-6 (B, F), IL-17a (C, G), and CRP (D, H), adjusting for maternal age, pre-pregnancy BMI, and race and ethnicity. 37](#_Toc216933990)

[**Supplementary Figure 4.** Volcano plots illustrating the associations between continuous maternal inflammation levels in the restricted temporal proximity sample (n=105) and neonatal inflammatory marker expression for each maternal marker: IL-1β (A), IL-6 (B), IL-17a (C), and CRP (D), adjusting for maternal age, pre-pregnancy BMI, and race and ethnicity. 38](#_Toc216933991)

[**Supplementary Table 11.** Regression estimates for associations between continuous maternal inflammation levels measured among the at-birth subset (n = 194), further adjusted for maternal age, pre-pregnancy BMI, and race and ethnicity, ordered by adjusted p-value 39](#_Toc216933992)

[**Supplementary Table 12.** Regression estimates for associations between continuous maternal inflammation levels measured among third trimester subset (n = 235), further adjusted for maternal age, pre-pregnancy BMI, and race and ethnicity, ordered by adjusted p-value 48](#_Toc216933993)

[**Supplementary Table 13.** Regression estimates for associations between continuous maternal inflammation levels measured among the restricted temporal proximity subset (n = 105), further adjusted for maternal age, pre-pregnancy BMI, and race and ethnicity, ordered by adjusted p-value 57](#_Toc216933994)

[**Supplementary Figure 5.** Volcano plots for differential expression of neonatal inflammatory markers relative to maternal inflammation levels at birth (I) and during third trimester (II) comparing high vs. low maternal inflammation groups for each maternal marker: IL-1β (A, E), IL-6 (B, F), IL-17a (C, G), and CRP (D, H). 66](#_Toc216933995)

[**Supplementary Figure 6**. Volcano plots for differential expression of neonatal inflammatory markers relative to maternal inflammation levels at birth in first sensitivity analysis comparing high vs. low maternal inflammation groups for each maternal marker: IL-1β (A), IL-6 (B), IL-17a (C), and CRP (D) in a subset of n=105 with maternal inflammatory marker and neonatal DBS collected with restricted temporal proximity. 67](#_Toc216933996)

[**Supplementary Table 14.** Differential expression results for all neonatal inflammatory markers between high vs. low maternal inflammation at birth (primary analysis; n=194), ordered by adjusted p-value 68](#_Toc216933997)

[**Supplementary Table 15.** Differential expression results for all neonatal inflammatory markers between high vs. low maternal inflammation in the third trimester (secondary analysis; n=235), ordered by adjusted p-value 77](#_Toc216933998)

[**Supplementary Table 16**. Differential expression results for all neonatal inflammatory markers between high and low maternal inflammation with restricted temporal proximity (sensitivity analysis; n=105), ordered by adjusted p-value 86](#_Toc216933999)

# **Supplementary Table 1.** Absolute maternal inflammatory marker level concentrations for maternal inflammation measured at birth and in the third trimester.

| **Marker** |  | **At-Birth**  N = 194^1^ | **Third Trimester**  N = 235^1^ |
| --- | --- | --- | --- |
| IL-1β (pg/mL) | Median (IQR) | 2.12 (1.26, 5.39) | 1.43 (0.89, 2.10) |
|  | Min; Max | 0.02; 376.34 | 0.02; 57.68 |
| IL-6 (pg/mL) | Median (IQR) | 1.55 (0.78, 4.77) | 0.94 (0.52, 2.03) |
|  | Min; Max | 0.01; 57.62 | 0.01; 65.08 |
| IL-17A (pg/mL) | Median (IQR) | 9.68 (7.04, 12.39) | 8.94 (6.74, 12.68) |
|  | Min; Max | 1.71; 228.11 | 2.49; 200.62 |
| CRP mg/L | Median (IQR) | 15.46 (10.31, 23.54) | 15.28 (9.47, 25.31) |
|  | Min; Max | 0.33; 333.51 | 0.32; 123.39 |
| Missing |  | 1 | 1 |
|  | |  | |

#

# **Supplementary Table 2**. Minimum detectable effect sizes (R^2^ and Cohen’s *d*) across analyses

| **Analysis** | **Maternal Comparison** | **Minimum detectable effect size** |
| --- | --- | --- |
| Maternal inflammation at birth  (primary, n=194) | Continuous | 0.0400^1^ |
|  | High vs. Low IL-1β | 0.465^2^ |
|  | High vs. Low IL-6 | 0.465^2^ |
|  | High vs. Low IL-17a | 0.465^2^ |
|  | High vs. Low CRP | 0.469^2^ |
| Maternal inflammation in third trimester (secondary, n=235) | Continuous | 0.0329^1^ |
|  | High vs. Low IL-1β | 0.423^2^ |
|  | High vs. Low IL-6 | 0.423^2^ |
|  | High vs. Low IL-17a | 0.423^2^ |
|  | High vs. Low CRP | 0.423^2^ |
| Restricted temporal proximity  (sensitivity analysis, n=105) | Continuous | 0.0728^1^ |
|  | High vs. Low IL-1β | 0.639^2^ |
|  | High vs. Low IL-6 | 0.639^2^ |
|  | High vs. Low IL-17a | 0.639^2^ |
|  | High vs. Low CRP | 0.639^2^ |

Post hoc power calculations were performed based on the available group sizes. These calculations estimate the minimum detectable effect size, namely ^1^**R^2^** or ^2^**Cohen’s d**, assuming 80% power at α = 0.05 and a two-sided test.

# **Supplementary Table 3.** Maternal inflammatory marker characteristics for maternal inflammation measured at birth (primary analysis) by inflammation levels

| **Characteristic**^†^ | **IL-1β at birth** | | **IL-6 at birth** | | **IL-17a at birth** | | **CRP at birth** | |
| --- | --- | --- | --- | --- | --- | --- | --- | --- |
|  | High  (N=49^1^) | Low  (N=145^1^) | High  (N=49^1^) | Low  (N=145^1^) | High  (N=49^1^) | Low  (N=145^1^) | High  (N=48^1^) | Low  (N=145^1^) |
| **IL-1β log2** | *** | | ** | | ** | |  | |
|  | 3.76  (3.14, 4.84) | 0.73  (0.11, 1.33) | 1.72  (1.06, 3.38) | 0.82  (0.19, 2.24) | 1.72  (0.80, 2.86) | 0.86  (0.11, 2.27) | 1.21  (-0.01, 3.29) | 1.07  (0.50, 2.35) |
| **IL-6 log2** |  | | *** | | ** | |  | |
|  | 1.12  (0.07, 2.89) | 0.51  (-0.40, 2.03) | 3.50  (2.80, 4.58) | 0.11  (-0.51, 1.01) | 1.41  (0.15, 3.33) | 0.40  (-0.43, 1.74) | 1.13 (0.04, 2.43) | 0.43  (-0.40, 2.05) |
| **IL-17a log2** |  |  |  |  | *** | |  |  |
|  | 3.23  (2.84, 3.73) | 3.30  (2.82, 3.62) | 3.56  (2.86, 3.98) | 3.24  (2.78, 3.54) | 4.01  (3.81, 4.22) | 3.02  (2.68, 3.33) | 3.20  (2.56, 3.75) | 3.29  (2.86, 3.62) |
| **CRP log2** |  |  |  |  |  |  | *** | |
|  | 13.99 (13.20, 14.65) | 13.81 (13.34, 14.47) | 13.97 (13.18, 14.67) | 13.89 (13.34, 14.47) | 13.92 (13.51, 14.54) | 13.92 (13.31, 14.50) | 15.12 (14.67, 15.72) | 13.70 (13.10, 13.99) |
| Missing | 0 | 1 | 0 | 1 | 0 | 1 | 0 | 1 |
| **IL-1β category** |  |  |  |  |  |  |  |  |
| High | -- | -- | 35% (17) | 22% (32) | 29% (14) | 24% (35) | 31% (15) | 23% (34) |
| Low | -- | -- | 65% (32) | 78% (113) | 71% (35) | 76% (110) | 69% (33) | 77% (111) |
| **IL-6 category** |  |  |  |  |  |  |  |  |
| High | 35% (17) | 22% (32) | -- | -- | 41% (20) | 20% (29) | 31% (15) | 23% (34) |
| Low | 65% (32) | 78% (113) | -- | -- | 59% (29) | 80% (116) | 69% (33) | 77% (111) |
| **IL-17a category** | |  | ** | |  |  |  |  |
| High | 29% (14) | 24% (35) | 41% (20) | 20% (29) | -- | -- | 29% (14) | 24% (35) |
| Low | 71% (35) | 76% (110) | 59% (29) | 80% (116) | -- | -- | 71% (34) | 76% (110) |
| **CRP category** |  |  |  |  |  |  |  |  |
| High | 31% (15) | 23% (33) | 31% (15) | 23% (33) | 29% (14) | 24% (34) | -- | --- |
| Low | 69% (34) | 77% (111) | 69% (34) | 77% (111) | 71% (35) | 76% (110) | --- | --- |

| ^1^Median (IQR); % (n). Note: percentages may not sum to 100% due to rounding.  ^†^ Values for IL-1β, IL-6, and IL-17a are log2-transformed concentrations (pg/mL); CRP is reported in mg/L. *p* < 0.05 (**), p < 0.01 (**),*  *p < 0.001 (****). *p*-values are based on Wilcoxon rank-sum tests for continuous variables and Chi-square or Fisher’s exact tests for categorical variables, comparing high vs. low groups within each maternal inflammatory marker |
| --- |

# **Supplementary Table 4.** Maternal and child demographic characteristics for maternal inflammation measured in the third trimester (secondary analysis) by inflammation levels

| **Characteristic**^†^ | **Overall** | **IL-1β in 3^rd^ trimester** | | **IL-6 in 3^rd^ trimester** | | **IL-17a in 3^rd^ trimester** | | **CRP in 3^rd^ trimester** | |
| --- | --- | --- | --- | --- | --- | --- | --- | --- | --- |
|  | **N=235**^1^ | High  (N=59^1^) | Low  (N=176^1^) | High  (N=59^1^) | Low  (N=176^1^) | High  (N=59^1^) | Low  (N=176^1^) | High  (N=59^1^) | Low  (N=176^1^) |
| **Maternal age at delivery (years)** |  |  |  | * | |  |  | * | |
|  | 33 (30, 36) | 33 (30, 36) | 33 (30, 36) | 31 (28, 35) | 33 (30, 36) | 33 (30, 35) | 33 (30, 36) | 31 (28, 35) | 33 (30, 36) |
| **Race-ethnicity** |  |  |  |  |  |  |  | ** | |
| Asian | 9% (20) | 15% (9) | 16% (28) | 12% (7) | 7.4% (13) | 12% (7) | 17% (30) | 9% (5) | 9% (15) |
| Black | 16% (37) | 32% (19) | 26% (45) | 12% (7) | 17% (30) | 25% (15) | 28% (49) | 20% (12) | 14% (25) |
| Hispanic | 27% (64) | 37% (22) | 43% (76) | 34% (20) | 25% (44) | 51% (30) | 39% (68) | 39% (23) | 23% (40) |
| White | 42% (98) | 5% (3) | 7% (13) | 32% (19) | 45% (79) | 3.4% (2) | 8.0% (14) | 32% (19) | 45% (79) |
| Other | 7% (16) | 15% (9) | 16% (28) | 10% (6) | 6% (10) | 12% (7) | 17% (30) | 0% (0) | 9% (16) |
| **Parity** |  |  |  |  |  |  |  |  |  |
| Nulliparous | 46% (107) | 44% (26) | 46% (81) | 41% (24) | 47% (83) | 49% (29) | 44% (78) | 36% (21) | 49% (85) |
| Multiparous | 54% (128) | 56% (33) | 54% (95) | 59% (35) | 53% (93) | 51% (30) | 56% (98) | 64% (38) | 51% (90) |
| **Education** |  |  |  |  |  |  |  | * | |
| Less than college | 25% (45) | 17% (8) | 28% (37) | 30% (14) | 23% (31) | 18% (9) | 28% (36) | 38% (19) | 20% (26) |
| $\geq$College | 75% (134) | 83% (40) | 72% (94) | 70% (32) | 77% (102) | 82% (41) | 72% (93) | 62% (31) | 80% (102) |
| Missing | 56 | 11 | 45 | 13 | 43 | 9 | 47 | 9 | 47 |
| **Child sex** |  |  |  |  |  |  |  |  |  |
| Female | 49% (114) | 53% (31) | 47% (83) | 42% (25) | 51% (89) | 49% (29) | 48% (85) | 49% (29) | 49% (85) |
| Male | 51% (121) | 47% (28) | 53% (93) | 58% (34) | 49% (87) | 51% (30) | 52% (91) | 51% (30) | 51% (90) |
| **Maternal history of mental illness** | | *** | |  |  |  |  |  |  |
| No | 52% (122) | 73% (43) | 45% (79) | 58% (34) | 50% (88) | 54% (32) | 51% (90) | 49% (28) | 53% (94) |
| Yes | 48% (113) | 27% (16) | 55% (97) | 42% (25) | 50% (88) | 46% (27) | 49% (86) | 51% (29) | 47% (84) |
| **SARS-CoV-2 infection during pregnancy** | |  |  |  |  |  |  | * | |
| No | 87% (204) | 92% (54) | 85% (150) | 83% (49) | 88% (155) | 88% (52) | 86% (152) | 78% (46) | 90% (157) |
| Yes | 13% (31) | 9% (5) | 15% (26) | 17% (10) | 12% (21) | 12% (7) | 14% (24) | 22% (13) | 10% (18) |
| **Neonate age at DBS collection (hrs)** |  |  |  |  |  |  |  | * | |
|  | 25 (24, 28) | 24 (24, 28) | 25 (24, 29) | 25 (24, 27) | 25 (24, 29) | 24 (24, 28) | 25 (24, 28) | 24 (24, 26) | 25 (24, 29) |
| **Pre-pregnancy BMI (kg/m^2^)** |  |  |  |  |  |  |  | *** | |
|  | 26 (22, 31) | 24 (21, 29) | 26 (23, 31) | 25 (22, 31) | 26 (22, 30) | 25 (22, 28) | 26 (22, 31) | 29 (24, 34) | 25 (22, 29) |
| **Delivery Mode** |  |  |  |  |  |  |  |  |  |
| C-section | 40% (95) | 37% (22) | 41% (73) | 36% (21) | 42% (74) | 39% (23) | 41% (72) | 42% (25) | 40% (70) |
| Vaginal Delivery | 60% (140) | 63% (37) | 59% (103) | 64% (38) | 58% (102) | 61% (36) | 59% (104) | 58% (34) | 60% (105) |
| **Cardiometabolic disorders of pregnancy** | |  |  |  |  |  |  |  |  |
| No | 69% (163) | 71% (42) | 69% (121) | 73% (43) | 68% (120) | 71% (44) | 69% (119) | 59% (35) | 73% (128) |
| Yes | 31% (72) | 29% (17) | 31% (55) | 27% (16) | 32% (56) | 29% (18) | 31% (54) | 41% (24) | 27% (47) |
| **Gestational age at delivery** (days) |  |  |  |  |  |  |  |  |  |
|  | 275  (271, 280) | 274  (272, 280) | 275  (271, 280) | 275  (272, 279) | 275  (271, 280) | 276  (273, 283) | 274  (271, 279) | 275  (269, 280) | 275  (272, 280) |
| **Preterm Birth** |  |  |  |  |  |  |  |  |  |
| No | 96% (225) | 93% (55) | 97% (170) | 97% (57) | 95% (168) | 95% (56) | 96% (169) | 98% (58) | 95% (167) |
| Yes | 4.3% (10) | 7% (4) | 3% (6) | 3% (2) | 5% (8) | 5% (3) | 4% (7) | 2% (1) | 5% (8) |
| **Birthweight (grams)** | 3,300 (3,020, 3,630) | 3,265 (2,980, 3,590) | 3,310 (3,047, 3,662) | 3,425 (3,060, 3,675) | 3,273 (3,010, 3,605) | 3,375 (3,010, 3,625) | 3,295 (3,022, 3,635) | 3,315 (3,005, 3,560) | 3,300 (3,025, 3,660) |
| **Gestational age sample (days)** |  |  |  | ** | |  |  |  |  |
|  | 221  (201, 253) | 235  (209, 253) | 216  (200, 253) | 247  (218, 254) | 214  (200, 253) | 238  (202, 254) | 218  (201, 253) | 275  (269, 280) | 275  (272, 280) |
| ^1^Median (IQR); %(n). Note: percentages may not sum to 100% due to rounding.  ^†^ *p* < 0.05 (**), p < 0.01 (**), p < 0.001 (****). *p*-values are based on Wilcoxon rank-sum tests for continuous variables and Chi-square or Fisher’s exact tests for categorical variables, comparing high vs. low groups within each maternal inflammatory marker | | | | | | | | | |

# **Supplementary Table 5.** Maternal inflammatory marker characteristics for maternal inflammation measured in the third trimester (secondary analysis) by inflammation levels

| **Characteristic**^†^ | **IL-1β in 3^rd^ trimester** | | **IL-6 in 3^rd^ trimester** | | **IL-17a in 3^rd^ trimester** | | **CRP in 3^rd^ trimester** | |
| --- | --- | --- | --- | --- | --- | --- | --- | --- |
|  | High  (N=59^1^) | Low  (N=176^1^) | High  (N=59^1^) | Low  (N=176^1^) | High  (N=59^1^) | Low  (N=176^1^) | High  (N=59^1^) | Low  (N=175^1^) |
| **IL-1β log2** | *** | | *** | | *** | |  |  |
|  | 1.79  (1.30, 2.75) | 0.24  (-0.36, 0.68) | 0.85  (0.37, 1.31) | 0.37  (-0.28, 0.96) | 1.23  (0.77, 2.00) | 0.30  (-0.36, 0.78) | 0.48  (-0.20, 0.98) | 0.53  (-0.17, 1.23) |
| **IL-6 log2** | ** | | *** | | ** | |  | |
|  | 0.14  (-0.47, 2.49) | -0.14  (-1.03, 0.67) | 2.25  (1.50, 3.21) | -0.46  (-1.18, 0.12) | 0.15  (-0.38, 1.82) | -0.20  (-1.07, 0.73) | 0.30  (-0.49, 1.24) | -0.22  (-0.97, 1.02) |
| **IL-17a log2** | *** | | *** | | *** | |  |  |
|  | 3.82  (3.05, 4.35) | 3.07  (2.68, 3.45) | 3.49  (3.03, 4.17) | 3.07  (2.72, 3.53) | 4.05  (3.85, 4.52) | 3.01  (2.64, 3.29) | 3.18  (2.82, 3.65) | 3.16  (2.75, 3.70) |
| **CRP log2** |  |  |  |  |  |  | *** | |
|  | 13.77 (13.27, 14.46) | 13.92 (13.17, 14.74) | 13.72 (12.95, 14.74) | 13.93 (13.25, 14.58) | 13.86 (13.30, 14.58) | 13.91 (13.14, 14.64) | 15.36 (14.92, 15.90) | 13.52 (12.87, 14.07) |
| Missing | 0 | 1 | 0 | 1 | 0 | 1 | 0 | 1 |
| **IL-1β category** |  | | ** | | *** | |  | |
| High | -- | -- | 39% (23) | 20% (36) | 58% (34) | 14% (25) | 17% (10) | 28% (49) |
| Low | -- | -- | 61% (36) | 80% (140) | 42% (25) | 86% (151) | 83% (49) | 72% (126) |
| **IL-6 category** | ** | |  | | ** | |  |  |
| High | 39% (23) | 20% (36) | -- | -- | 37% (22) | 21% (37) | 25% (15) | 25% (44) |
| Low | 61% (36) | 80% (140) | -- | -- | 63% (37) | 79% (139) | 75% (44) | 75% (131) |
| **IL-17a category** | *** | | ** | |  |  |  |  |
| High | 58% (34) | 14% (25) | 37% (22) | 21% (37) | -- | -- | 24% (14) | 26% (45) |
| Low | 42% (25) | 86% (151) | 63% (37) | 79% (139) | -- | -- | 76% (45) | 74% (130) |
| **CRP category** | * | |  |  |  |  |  |  |
| High | 17% (10) | 28% (49) | 25% (15) | 25% (44) | 24% (14) | 26% (45) | -- | -- |
| Low | 83% (49) | 72% (126) | 75% (44) | 75% (131) | 76% (45) | 74% (130) | -- | -- |

^1^Median (IQR); %(n). Note: percentages may not sum to 100% due to rounding.

^†^ Values for IL-1β, IL-6, and IL-17a are log2-transformed concentrations (pg/mL); CRP is reported in mg/L. *p* < 0.05 (**), p < 0.01 (**),*

*p < 0.001 (****). *p*-values are based on Wilcoxon rank-sum tests for continuous variables and Chi-square or Fisher’s exact tests for categorical variables, comparing high vs. low groups within each maternal inflammatory marker

**
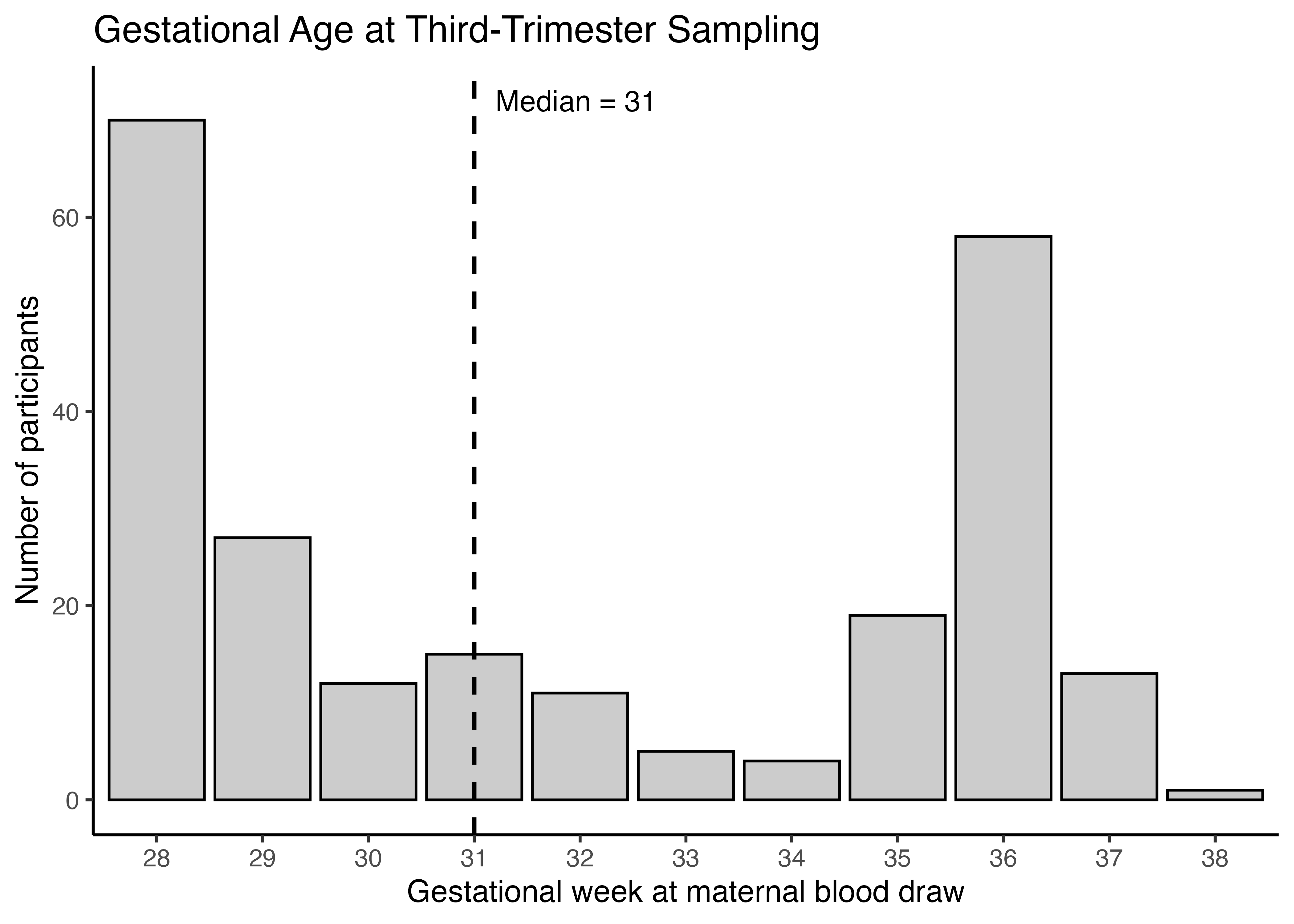
**

# **Supplementary Figure 1.** Distribution of gestational age (weeks) at third-trimester maternal blood sampling. Histogram displays the gestational week at which maternal inflammatory markers were measured during the third trimester subset (N=235)

# **Supplementary Table 6.** Regression estimates for associations between continuous maternal inflammation levels measured at birth and neonatal inflammatory markers (n = 194), ordered by adjusted p-value

| **Neonatal  inflammatory marker** | **Maternal Inflammatory Marker** | **Beta** | **95% CI Low** | **95% CI High** | **p-value** | **Adjusted p-value** |  |
| --- | --- | --- | --- | --- | --- | --- | --- |
| FGF.21 | IL1B | 0.2778 | 0.1083 | 0.4473 | 0.0014 | 0.0806 |  |
| OSM | IL1B | 0.1613 | 0.0611 | 0.2616 | 0.0018 | 0.0806 |  |
| CX3CL1 | IL1B | 0.0970 | 0.0293 | 0.1648 | 0.0052 | 0.1600 |  |
| CXCL1 | IL1B | 0.0994 | 0.0273 | 0.1714 | 0.0071 | 0.1636 |  |
| IL.18R1 | IL1B | 0.1514 | 0.0378 | 0.2650 | 0.0093 | 0.1706 |  |
| MMP.10 | IL1B | 0.1181 | 0.0225 | 0.2137 | 0.0157 | 0.2414 |  |
| CCL20 | IL1B | 0.1307 | 0.0157 | 0.2458 | 0.0262 | 0.2624 |  |
| CSF.1 | IL1B | 0.0676 | 0.0081 | 0.1272 | 0.0262 | 0.2624 |  |
| GDNF | IL1B | -0.0661 | -0.1245 | -0.0077 | 0.0269 | 0.2624 |  |
| TSLP | IL1B | 0.0579 | 0.0058 | 0.1100 | 0.0297 | 0.2624 |  |
| ST1A1 | IL1B | -0.0838 | -0.1613 | -0.0063 | 0.0341 | 0.2624 |  |
| Flt3L | IL1B | 0.0766 | 0.0058 | 0.1474 | 0.0342 | 0.2624 |  |
| IL6 | IL1B | 0.1387 | 0.0052 | 0.2722 | 0.0417 | 0.2787 |  |
| PD.L1 | IL1B | 0.0720 | 0.0025 | 0.1415 | 0.0424 | 0.2787 |  |
| SCF | IL1B | 0.0830 | -0.0009 | 0.1668 | 0.0524 | 0.2808 |  |
| IL18 | IL1B | 0.0936 | -0.0012 | 0.1884 | 0.0530 | 0.2808 |  |
| CD5 | IL1B | 0.1218 | -0.0018 | 0.2454 | 0.0534 | 0.2808 |  |
| CD6 | IL1B | 0.1106 | -0.0038 | 0.2249 | 0.0580 | 0.2808 |  |
| IL8 | IL1B | 0.1109 | -0.0046 | 0.2264 | 0.0597 | 0.2808 |  |
| TRAIL | IL1B | 0.0725 | -0.0035 | 0.1485 | 0.0614 | 0.2808 |  |
| CCL23 | IL1B | 0.1023 | -0.0082 | 0.2128 | 0.0694 | 0.2808 |  |
| IFN.gamma | IL1B | 0.1548 | -0.0125 | 0.3221 | 0.0695 | 0.2808 |  |
| uPA | IL1B | 0.0755 | -0.0063 | 0.1574 | 0.0702 | 0.2808 |  |
| Flt3L | IL17A | -0.0867 | -0.1472 | -0.0263 | 0.0051 | 0.2825 |  |
| CXCL11 | IL17A | -0.1748 | -0.3037 | -0.0458 | 0.0082 | 0.2825 |  |
| IL.17C | IL17A | -0.1385 | -0.2423 | -0.0347 | 0.0092 | 0.2825 |  |
| HGF | IL1B | 0.0874 | -0.0086 | 0.1834 | 0.0741 | 0.2841 |  |
| AXIN1 | IL1B | 0.0672 | -0.0093 | 0.1436 | 0.0846 | 0.3115 |  |
| IL.17C | IL1B | 0.1037 | -0.0182 | 0.2255 | 0.0950 | 0.3270 |  |
| MCP.1 | IL1B | 0.1222 | -0.0219 | 0.2664 | 0.0960 | 0.3270 |  |
| NRTN | IL17A | 0.0352 | 0.0057 | 0.0647 | 0.0198 | 0.3688 |  |
| IL6 | IL17A | -0.1362 | -0.2508 | -0.0217 | 0.0200 | 0.3688 |  |
| MCP.3 | IL1B | 0.0977 | -0.0260 | 0.2213 | 0.1209 | 0.3937 |  |
| IL.17A | IL1B | 0.0387 | -0.0107 | 0.0881 | 0.1241 | 0.3937 |  |
| LIF.R | IL1B | 0.0200 | -0.0067 | 0.0468 | 0.1415 | 0.4223 |  |
| CXCL6 | IL1B | 0.0712 | -0.0246 | 0.1670 | 0.1445 | 0.4223 |  |
| CASP.8 | IL1B | 0.0421 | -0.0149 | 0.0992 | 0.1469 | 0.4223 |  |
| ADA | IL1B | 0.0307 | -0.0126 | 0.0740 | 0.1636 | 0.4370 |  |
| CCL11 | IL1B | 0.0938 | -0.0386 | 0.2261 | 0.1638 | 0.4370 |  |
| TGF.alpha | IL1B | 0.0573 | -0.0252 | 0.1398 | 0.1724 | 0.4370 |  |
| IL.10RB | IL1B | 0.0439 | -0.0196 | 0.1075 | 0.1742 | 0.4370 |  |
| X4E.BP1 | IL1B | -0.0205 | -0.0505 | 0.0095 | 0.1799 | 0.4370 |  |
| CCL4 | IL1B | 0.0628 | -0.0300 | 0.1555 | 0.1835 | 0.4370 |  |
| NRTN | IL1B | -0.0233 | -0.0580 | 0.0113 | 0.1852 | 0.4370 |  |
| CD40 | IL1B | 0.0559 | -0.0352 | 0.1470 | 0.2276 | 0.5235 |  |
| CCL19 | IL1B | 0.0489 | -0.0326 | 0.1303 | 0.2384 | 0.5350 |  |
| CCL25 | IL1B | 0.0431 | -0.0315 | 0.1177 | 0.2561 | 0.5487 |  |
| IL7 | IL1B | 0.0277 | -0.0203 | 0.0757 | 0.2564 | 0.5487 |  |
| CD8A | IL1B | 0.0544 | -0.0432 | 0.1521 | 0.2730 | 0.5709 |  |
| IL10 | IL1B | 0.0308 | -0.0272 | 0.0887 | 0.2968 | 0.5860 |  |
| IL4 | IL1B | 0.0324 | -0.0290 | 0.0938 | 0.2989 | 0.5860 |  |
| CCL3 | IL1B | 0.1074 | -0.0962 | 0.3110 | 0.2994 | 0.5860 |  |
| EN.RAGE | IL1B | -0.0426 | -0.1248 | 0.0395 | 0.3072 | 0.5887 |  |
| IL5 | IL1B | -0.0266 | -0.0790 | 0.0258 | 0.3181 | 0.5922 |  |
| TNFRSF9 | IL1B | 0.0414 | -0.0431 | 0.1258 | 0.3352 | 0.5922 |  |
| IL.15RA | IL1B | 0.0238 | -0.0249 | 0.0726 | 0.3357 | 0.5922 |  |
| ARTN | IL1B | -0.0277 | -0.0844 | 0.0290 | 0.3366 | 0.5922 |  |
| VEGFA | IL1B | 0.0436 | -0.0474 | 0.1345 | 0.3458 | 0.5922 |  |
| IL.22.RA1 | IL1B | -0.0435 | -0.1346 | 0.0476 | 0.3476 | 0.5922 |  |
| DNER | IL1B | 0.0283 | -0.0321 | 0.0887 | 0.3571 | 0.5973 |  |
| CCL28 | IL1B | -0.0152 | -0.0486 | 0.0182 | 0.3703 | 0.6084 |  |
| IL.10RA | IL1B | 0.0145 | -0.0189 | 0.0478 | 0.3934 | 0.6349 |  |
| CXCL9 | IL1B | -0.0324 | -0.1096 | 0.0447 | 0.4083 | 0.6477 |  |
| CST5 | IL1B | 0.0474 | -0.0746 | 0.1693 | 0.4444 | 0.6742 |  |
| OPG | IL1B | 0.0274 | -0.0436 | 0.0983 | 0.4474 | 0.6742 |  |
| CXCL5 | IL1B | -0.0528 | -0.1927 | 0.0870 | 0.4572 | 0.6742 |  |
| STAMBP | IL1B | 0.0082 | -0.0136 | 0.0301 | 0.4581 | 0.6742 |  |
| CXCL10 | IL1B | 0.0383 | -0.0641 | 0.1407 | 0.4616 | 0.6742 |  |
| TRANCE | IL1B | -0.0317 | -0.1192 | 0.0559 | 0.4766 | 0.6851 |  |
| TNFSF14 | IL1B | 0.0331 | -0.0676 | 0.1338 | 0.5179 | 0.7250 |  |
| CDCP1 | IL1B | -0.0183 | -0.0745 | 0.0378 | 0.5201 | 0.7250 |  |
| TNFB | IL1B | -0.0162 | -0.0669 | 0.0345 | 0.5286 | 0.7255 |  |
| SLAMF1 | IL1B | 0.0174 | -0.0379 | 0.0726 | 0.5362 | 0.7255 |  |
| LIF | IL1B | -0.0126 | -0.0548 | 0.0295 | 0.5553 | 0.7340 |  |
| FGF.23 | IL1B | -0.0255 | -0.1112 | 0.0602 | 0.5585 | 0.7340 |  |
| FGF.19 | IL6 | -0.1290 | -0.2314 | -0.0266 | 0.0139 | 0.7532 |  |
| CCL19 | IL6 | 0.0854 | 0.0158 | 0.1549 | 0.0164 | 0.7532 |  |
| IL.20RA | IL1B | -0.0110 | -0.0514 | 0.0295 | 0.5942 | 0.7700 |  |
| CCL11 | CRP | -0.1345 | -0.2461 | -0.0228 | 0.0185 | 0.8080 |  |
| TWEAK | CRP | -0.0525 | -0.1035 | -0.0015 | 0.0435 | 0.8080 |  |
| IL4 | CRP | -0.0523 | -0.1041 | -0.0006 | 0.0476 | 0.8080 |  |
| uPA | CRP | -0.0695 | -0.1386 | -0.0004 | 0.0487 | 0.8080 |  |
| FGF.19 | CRP | -0.0950 | -0.1963 | 0.0063 | 0.0658 | 0.8080 |  |
| IL10 | CRP | 0.0424 | -0.0066 | 0.0915 | 0.0896 | 0.8080 |  |
| HGF | CRP | -0.0696 | -0.1513 | 0.0121 | 0.0943 | 0.8080 |  |
| Flt3L | CRP | -0.0510 | -0.1114 | 0.0094 | 0.0976 | 0.8080 |  |
| TNFSF14 | CRP | -0.0717 | -0.1569 | 0.0135 | 0.0985 | 0.8080 |  |
| IL.17A | CRP | -0.0348 | -0.0768 | 0.0072 | 0.1040 | 0.8080 |  |
| VEGFA | CRP | -0.0611 | -0.1383 | 0.0160 | 0.1198 | 0.8080 |  |
| IL6 | CRP | -0.0877 | -0.2019 | 0.0264 | 0.1312 | 0.8080 |  |
| X4E.BP1 | CRP | 0.0195 | -0.0060 | 0.0449 | 0.1332 | 0.8080 |  |
| MCP.1 | CRP | -0.0901 | -0.2131 | 0.0328 | 0.1499 | 0.8080 |  |
| CCL23 | CRP | -0.0682 | -0.1625 | 0.0261 | 0.1554 | 0.8080 |  |
| MMP.10 | CRP | 0.0591 | -0.0229 | 0.1412 | 0.1565 | 0.8080 |  |
| LAP.TGF.beta.1 | CRP | -0.0415 | -0.0993 | 0.0163 | 0.1583 | 0.8080 |  |
| IL33 | CRP | 0.0283 | -0.0118 | 0.0684 | 0.1654 | 0.8080 |  |
| IL.1.alpha | CRP | 0.1365 | -0.0598 | 0.3329 | 0.1718 | 0.8080 |  |
| CST5 | CRP | -0.0704 | -0.1739 | 0.0331 | 0.1813 | 0.8080 |  |
| SCF | CRP | -0.0485 | -0.1203 | 0.0233 | 0.1844 | 0.8080 |  |
| IL.2RB | IL1B | 0.0099 | -0.0320 | 0.0518 | 0.6405 | 0.8155 |  |
| NT.3 | IL1B | 0.0125 | -0.0413 | 0.0663 | 0.6471 | 0.8155 |  |
| MCP.4 | IL1B | 0.0304 | -0.1057 | 0.1666 | 0.6597 | 0.8202 |  |
| LIF.R | IL17A | -0.0214 | -0.0444 | 0.0016 | 0.0676 | 0.8206 |  |
| TNFRSF9 | IL17A | -0.0625 | -0.1349 | 0.0098 | 0.0897 | 0.8206 |  |
| PD.L1 | IL17A | -0.0508 | -0.1109 | 0.0092 | 0.0966 | 0.8206 |  |
| TRANCE | IL17A | -0.0627 | -0.1377 | 0.0123 | 0.1006 | 0.8206 |  |
| TNFB | IL17A | -0.0361 | -0.0795 | 0.0073 | 0.1023 | 0.8206 |  |
| IL.10RB | IL17A | -0.0452 | -0.0998 | 0.0094 | 0.1040 | 0.8206 |  |
| FGF.19 | IL17A | -0.0840 | -0.1871 | 0.0191 | 0.1096 | 0.8206 |  |
| IL.12B | IL17A | -0.0654 | -0.1486 | 0.0179 | 0.1233 | 0.8206 |  |
| FGF.23 | IL17A | -0.0569 | -0.1304 | 0.0165 | 0.1277 | 0.8206 |  |
| IL.1.alpha | IL17A | -0.1514 | -0.3496 | 0.0469 | 0.1338 | 0.8206 |  |
| CD244 | IL1B | 0.0175 | -0.0707 | 0.1057 | 0.6961 | 0.8438 |  |
| IL33 | IL1B | 0.0094 | -0.0383 | 0.0571 | 0.6970 | 0.8438 |  |
| SIRT2 | IL1B | 0.0058 | -0.0288 | 0.0403 | 0.7427 | 0.8874 |  |
| MMP.1 | CRP | -0.0772 | -0.2087 | 0.0544 | 0.2487 | 0.8890 |  |
| CCL20 | CRP | 0.0562 | -0.0416 | 0.1541 | 0.2584 | 0.8890 |  |
| FGF.23 | CRP | 0.0398 | -0.0328 | 0.1124 | 0.2805 | 0.8890 |  |
| NT.3 | CRP | 0.0241 | -0.0212 | 0.0694 | 0.2947 | 0.8890 |  |
| LIF.R | CRP | -0.0121 | -0.0349 | 0.0106 | 0.2948 | 0.8890 |  |
| MCP.4 | CRP | -0.0595 | -0.1750 | 0.0561 | 0.3112 | 0.8890 |  |
| TRAIL | CRP | -0.0331 | -0.0981 | 0.0319 | 0.3160 | 0.8890 |  |
| TNFRSF9 | CRP | -0.0359 | -0.1078 | 0.0360 | 0.3256 | 0.8890 |  |
| ST1A1 | CRP | -0.0323 | -0.0989 | 0.0343 | 0.3404 | 0.8890 |  |
| TSLP | CRP | -0.0215 | -0.0658 | 0.0229 | 0.3408 | 0.8890 |  |
| FGF.5 | CRP | -0.0247 | -0.0766 | 0.0271 | 0.3480 | 0.8890 |  |
| SIRT2 | CRP | -0.0138 | -0.0432 | 0.0155 | 0.3546 | 0.8890 |  |
| CXCL5 | CRP | 0.0549 | -0.0641 | 0.1739 | 0.3638 | 0.8890 |  |
| MCP.2 | CRP | 0.0392 | -0.0489 | 0.1273 | 0.3811 | 0.8890 |  |
| IL.17C | CRP | 0.0452 | -0.0579 | 0.1482 | 0.3885 | 0.8890 |  |
| IL.20 | CRP | -0.0140 | -0.0465 | 0.0184 | 0.3955 | 0.8890 |  |
| LIF | CRP | 0.0152 | -0.0205 | 0.0509 | 0.4018 | 0.8890 |  |
| IL.2RB | CRP | 0.0150 | -0.0202 | 0.0502 | 0.4025 | 0.8890 |  |
| IL.22.RA1 | CRP | -0.0330 | -0.1106 | 0.0446 | 0.4028 | 0.8890 |  |
| IL8 | CRP | 0.0416 | -0.0573 | 0.1405 | 0.4078 | 0.8890 |  |
| CD40 | CRP | -0.0324 | -0.1101 | 0.0454 | 0.4123 | 0.8890 |  |
| EN.RAGE | CRP | -0.0288 | -0.0985 | 0.0408 | 0.4155 | 0.8890 |  |
| CCL11 | IL17A | 0.0800 | -0.0339 | 0.1940 | 0.1676 | 0.8939 |  |
| TNF | IL17A | -0.0493 | -0.1206 | 0.0221 | 0.1747 | 0.8939 |  |
| IL.15RA | IL17A | 0.0277 | -0.0142 | 0.0696 | 0.1934 | 0.8939 |  |
| DNER | IL17A | -0.0334 | -0.0854 | 0.0185 | 0.2056 | 0.8939 |  |
| SCF | IL17A | -0.0453 | -0.1179 | 0.0273 | 0.2201 | 0.8939 |  |
| TNFSF14 | IL17A | 0.0538 | -0.0326 | 0.1403 | 0.2208 | 0.8939 |  |
| IL4 | IL17A | -0.0328 | -0.0856 | 0.0199 | 0.2213 | 0.8939 |  |
| CDCP1 | IL17A | -0.0297 | -0.0779 | 0.0185 | 0.2262 | 0.8939 |  |
| CD8A | IL17A | -0.0505 | -0.1346 | 0.0335 | 0.2373 | 0.8939 |  |
| IL8 | IL17A | -0.0579 | -0.1579 | 0.0421 | 0.2550 | 0.8939 |  |
| IL.17A | IL17A | 0.0245 | -0.0181 | 0.0672 | 0.2580 | 0.8939 |  |
| EN.RAGE | IL17A | 0.0403 | -0.0304 | 0.1110 | 0.2624 | 0.8939 |  |
| CCL3 | CRP | 0.0696 | -0.1040 | 0.2432 | 0.4298 | 0.8961 |  |
| OPG | CRP | -0.0234 | -0.0834 | 0.0365 | 0.4417 | 0.8961 |  |
| IL.20RA | CRP | 0.0132 | -0.0211 | 0.0475 | 0.4481 | 0.8961 |  |
| CSF.1 | IL17A | -0.0284 | -0.0802 | 0.0234 | 0.2815 | 0.9071 |  |
| MMP.1 | IL17A | -0.0690 | -0.2021 | 0.0640 | 0.3074 | 0.9071 |  |
| CXCL9 | IL17A | -0.0336 | -0.1000 | 0.0327 | 0.3189 | 0.9071 |  |
| IL.2RB | IL17A | -0.0182 | -0.0542 | 0.0178 | 0.3194 | 0.9071 |  |
| uPA | IL17A | 0.0350 | -0.0359 | 0.1059 | 0.3318 | 0.9071 |  |
| MMP.10 | IL17A | -0.0393 | -0.1228 | 0.0441 | 0.3534 | 0.9071 |  |
| IL.22.RA1 | IL17A | 0.0366 | -0.0419 | 0.1150 | 0.3592 | 0.9071 |  |
| MCP.3 | IL17A | -0.0483 | -0.1552 | 0.0587 | 0.3743 | 0.9071 |  |
| OSM | IL17A | -0.0398 | -0.1282 | 0.0485 | 0.3750 | 0.9071 |  |
| IL13 | IL17A | -0.0158 | -0.0511 | 0.0194 | 0.3769 | 0.9071 |  |
| VEGFA | IL17A | -0.0346 | -0.1129 | 0.0438 | 0.3854 | 0.9071 |  |
| CXCL5 | IL17A | -0.0523 | -0.1727 | 0.0681 | 0.3926 | 0.9071 |  |
| CASP.8 | IL17A | 0.0213 | -0.0280 | 0.0706 | 0.3949 | 0.9071 |  |
| CCL20 | IL17A | -0.0425 | -0.1427 | 0.0577 | 0.4042 | 0.9071 |  |
| AXIN1 | IL17A | -0.0256 | -0.0918 | 0.0407 | 0.4471 | 0.9200 |  |
| Beta.NGF | IL17A | 0.0057 | -0.0092 | 0.0206 | 0.4523 | 0.9200 |  |
| CCL4 | IL17A | -0.0302 | -0.1103 | 0.0500 | 0.4586 | 0.9200 |  |
| IL.10RA | IL17A | -0.0096 | -0.0383 | 0.0191 | 0.5104 | 0.9200 |  |
| TWEAK | IL17A | 0.0174 | -0.0347 | 0.0694 | 0.5113 | 0.9200 |  |
| TGF.alpha | IL17A | 0.0232 | -0.0481 | 0.0945 | 0.5219 | 0.9200 |  |
| STAMBP | IL17A | 0.0061 | -0.0127 | 0.0249 | 0.5256 | 0.9200 |  |
| TSLP | IL17A | 0.0140 | -0.0314 | 0.0594 | 0.5434 | 0.9200 |  |
| ST1A1 | IL17A | 0.0206 | -0.0468 | 0.0880 | 0.5476 | 0.9200 |  |
| CST5 | IL17A | -0.0309 | -0.1360 | 0.0741 | 0.5620 | 0.9200 |  |
| CD244 | IL17A | -0.0222 | -0.0981 | 0.0537 | 0.5641 | 0.9200 |  |
| IL18 | IL17A | 0.0237 | -0.0587 | 0.1061 | 0.5710 | 0.9200 |  |
| SLAMF1 | IL17A | 0.0136 | -0.0340 | 0.0612 | 0.5732 | 0.9200 |  |
| MCP.4 | IL17A | -0.0322 | -0.1494 | 0.0850 | 0.5887 | 0.9200 |  |
| CD6 | IL17A | -0.0265 | -0.1259 | 0.0728 | 0.5991 | 0.9200 |  |
| FGF.21 | IL17A | 0.0397 | -0.1101 | 0.1895 | 0.6014 | 0.9200 |  |
| CXCL1 | IL17A | 0.0165 | -0.0466 | 0.0797 | 0.6062 | 0.9200 |  |
| ADA | IL17A | 0.0097 | -0.0278 | 0.0471 | 0.6109 | 0.9200 |  |
| IL.20 | IL17A | -0.0085 | -0.0416 | 0.0247 | 0.6156 | 0.9200 |  |
| CCL3 | IL17A | -0.0434 | -0.2191 | 0.1323 | 0.6267 | 0.9200 |  |
| OPG | IL17A | -0.0151 | -0.0762 | 0.0461 | 0.6274 | 0.9200 |  |
| IL2 | IL17A | 0.0108 | -0.0333 | 0.0549 | 0.6300 | 0.9200 |  |
| NT.3 | IL17A | 0.0109 | -0.0354 | 0.0572 | 0.6439 | 0.9215 |  |
| X4E.BP1 | IL17A | 0.0059 | -0.0200 | 0.0318 | 0.6545 | 0.9215 |  |
| IL5 | IL17A | 0.0099 | -0.0353 | 0.0551 | 0.6654 | 0.9215 |  |
| CCL28 | IL17A | 0.0060 | -0.0228 | 0.0348 | 0.6812 | 0.9215 |  |
| CCL25 | IL17A | -0.0134 | -0.0779 | 0.0510 | 0.6815 | 0.9215 |  |
| IL33 | IL17A | -0.0083 | -0.0493 | 0.0328 | 0.6911 | 0.9215 |  |
| IL.12B | IL6 | 0.0736 | -0.0097 | 0.1570 | 0.0831 | 0.9267 |  |
| TSLP | IL6 | 0.0394 | -0.0058 | 0.0846 | 0.0871 | 0.9267 |  |
| CDCP1 | IL6 | -0.0412 | -0.0894 | 0.0070 | 0.0935 | 0.9267 |  |
| DNER | IL6 | -0.0442 | -0.0961 | 0.0077 | 0.0947 | 0.9267 |  |
| CXCL10 | IL6 | 0.0724 | -0.0155 | 0.1604 | 0.1059 | 0.9267 |  |
| IL8 | IL6 | 0.0809 | -0.0190 | 0.1809 | 0.1120 | 0.9267 |  |
| CXCL5 | IL6 | -0.0914 | -0.2116 | 0.0288 | 0.1354 | 0.9267 |  |
| EN.RAGE | IL6 | -0.0517 | -0.1224 | 0.0190 | 0.1511 | 0.9267 |  |
| SCF | IL6 | -0.0526 | -0.1253 | 0.0201 | 0.1551 | 0.9267 |  |
| ST1A1 | IL6 | -0.0484 | -0.1157 | 0.0190 | 0.1582 | 0.9267 |  |
| VEGFA | IL6 | -0.0527 | -0.1310 | 0.0257 | 0.1862 | 0.9267 |  |
| IL.20RA | IL6 | 0.0220 | -0.0129 | 0.0568 | 0.2152 | 0.9267 |  |
| CCL20 | IL6 | 0.0628 | -0.0374 | 0.1631 | 0.2179 | 0.9267 |  |
| MCP.4 | IL6 | 0.0704 | -0.0468 | 0.1876 | 0.2375 | 0.9267 |  |
| CCL11 | IL6 | 0.0680 | -0.0464 | 0.1824 | 0.2427 | 0.9267 |  |
| OPG | IL6 | -0.0358 | -0.0969 | 0.0254 | 0.2500 | 0.9267 |  |
| IL.20 | IL6 | -0.0188 | -0.0520 | 0.0144 | 0.2646 | 0.9267 |  |
| LAP.TGF.beta.1 | IL6 | -0.0326 | -0.0914 | 0.0261 | 0.2742 | 0.9267 |  |
| IFN.gamma | IL6 | 0.0805 | -0.0647 | 0.2258 | 0.2755 | 0.9267 |  |
| IL10 | IL6 | -0.0274 | -0.0775 | 0.0226 | 0.2815 | 0.9267 |  |
| CXCL9 | IL6 | 0.0364 | -0.0301 | 0.1029 | 0.2820 | 0.9267 |  |
| CD5 | IL6 | 0.0584 | -0.0490 | 0.1658 | 0.2849 | 0.9267 |  |
| TRANCE | IL6 | -0.0401 | -0.1156 | 0.0353 | 0.2955 | 0.9267 |  |
| MCP.3 | IL6 | 0.0565 | -0.0507 | 0.1636 | 0.2999 | 0.9267 |  |
| CD6 | IL6 | 0.0496 | -0.0498 | 0.1490 | 0.3265 | 0.9267 |  |
| CCL25 | IL6 | 0.0318 | -0.0327 | 0.0963 | 0.3317 | 0.9267 |  |
| FGF.21 | IL6 | 0.0721 | -0.0778 | 0.2220 | 0.3440 | 0.9267 |  |
| TWEAK | IL6 | -0.0234 | -0.0755 | 0.0288 | 0.3777 | 0.9267 |  |
| TGF.alpha | IL6 | -0.0307 | -0.1021 | 0.0408 | 0.3981 | 0.9267 |  |
| MMP.10 | IL6 | 0.0348 | -0.0489 | 0.1185 | 0.4130 | 0.9267 |  |
| IL5 | IL6 | -0.0178 | -0.0631 | 0.0275 | 0.4396 | 0.9267 |  |
| CCL4 | IL6 | 0.0312 | -0.0491 | 0.1115 | 0.4448 | 0.9267 |  |
| CST5 | IL6 | 0.0392 | -0.0661 | 0.1445 | 0.4637 | 0.9267 |  |
| FGF.23 | IL6 | -0.0271 | -0.1011 | 0.0468 | 0.4704 | 0.9267 |  |
| OSM | IL6 | 0.0316 | -0.0571 | 0.1203 | 0.4832 | 0.9267 |  |
| CASP.8 | IL6 | -0.0174 | -0.0669 | 0.0320 | 0.4874 | 0.9267 |  |
| IL.22.RA1 | IL6 | -0.0278 | -0.1065 | 0.0510 | 0.4877 | 0.9267 |  |
| IL.10RA | IL6 | -0.0098 | -0.0386 | 0.0190 | 0.5038 | 0.9267 |  |
| IL7 | IL6 | -0.0138 | -0.0553 | 0.0278 | 0.5132 | 0.9267 |  |
| MCP.1 | IL6 | 0.0415 | -0.0837 | 0.1667 | 0.5140 | 0.9267 |  |
| NT.3 | IL6 | 0.0152 | -0.0312 | 0.0616 | 0.5189 | 0.9267 |  |
| TNFRSF9 | IL6 | -0.0239 | -0.0969 | 0.0491 | 0.5192 | 0.9267 |  |
| IL4 | IL6 | 0.0165 | -0.0366 | 0.0696 | 0.5400 | 0.9267 |  |
| HGF | IL6 | 0.0258 | -0.0577 | 0.1093 | 0.5427 | 0.9267 |  |
| PD.L1 | IL6 | 0.0182 | -0.0424 | 0.0788 | 0.5538 | 0.9267 |  |
| IL.17C | IL6 | 0.0305 | -0.0754 | 0.1364 | 0.5704 | 0.9267 |  |
| MMP.1 | IL6 | 0.0384 | -0.0953 | 0.1721 | 0.5718 | 0.9267 |  |
| NRTN | IL6 | 0.0084 | -0.0216 | 0.0384 | 0.5796 | 0.9267 |  |
| CXCL11 | IL6 | 0.0362 | -0.0954 | 0.1678 | 0.5876 | 0.9267 |  |
| Beta.NGF | IL6 | -0.0041 | -0.0190 | 0.0109 | 0.5918 | 0.9267 |  |
| TRAIL | IL6 | 0.0180 | -0.0482 | 0.0841 | 0.5927 | 0.9267 |  |
| IL.15RA | IL6 | -0.0112 | -0.0533 | 0.0310 | 0.6021 | 0.9267 |  |
| LIF.R | IL6 | -0.0061 | -0.0294 | 0.0171 | 0.6023 | 0.9267 |  |
| CD244 | IL6 | -0.0197 | -0.0959 | 0.0564 | 0.6095 | 0.9267 |  |
| CSF.1 | IL6 | 0.0135 | -0.0386 | 0.0655 | 0.6103 | 0.9267 |  |
| AXIN1 | IL6 | 0.0171 | -0.0494 | 0.0835 | 0.6128 | 0.9267 |  |
| IL6 | IL6 | -0.0292 | -0.1456 | 0.0873 | 0.6217 | 0.9267 |  |
| IL2 | IL6 | -0.0100 | -0.0542 | 0.0342 | 0.6551 | 0.9267 |  |
| SIRT2 | IL6 | -0.0067 | -0.0365 | 0.0232 | 0.6603 | 0.9267 |  |
| CCL28 | IL6 | 0.0064 | -0.0225 | 0.0353 | 0.6620 | 0.9267 |  |
| TNFB | IL6 | -0.0097 | -0.0534 | 0.0341 | 0.6639 | 0.9267 |  |
| X4E.BP1 | IL6 | -0.0056 | -0.0316 | 0.0204 | 0.6739 | 0.9267 |  |
| CXCL1 | IL6 | 0.0132 | -0.0502 | 0.0765 | 0.6827 | 0.9267 |  |
| ADA | IL6 | 0.0077 | -0.0298 | 0.0453 | 0.6854 | 0.9267 |  |
| IL18 | IL6 | -0.0165 | -0.0991 | 0.0661 | 0.6939 | 0.9267 |  |
| IL.10RB | IL6 | -0.0109 | -0.0660 | 0.0442 | 0.6964 | 0.9267 |  |
| CX3CL1 | IL6 | 0.0117 | -0.0480 | 0.0714 | 0.6992 | 0.9267 |  |
| TNF | IL6 | -0.0131 | -0.0849 | 0.0588 | 0.7198 | 0.9267 |  |
| IL.18R1 | IL6 | 0.0180 | -0.0818 | 0.1178 | 0.7222 | 0.9267 |  |
| SLAMF1 | IL6 | 0.0085 | -0.0392 | 0.0563 | 0.7253 | 0.9267 |  |
| CCL19 | IL17A | -0.0135 | -0.0839 | 0.0569 | 0.7053 | 0.9270 |  |
| IL7 | CRP | -0.0138 | -0.0547 | 0.0272 | 0.5076 | 0.9292 |  |
| IL.15RA | CRP | -0.0137 | -0.0551 | 0.0278 | 0.5156 | 0.9292 |  |
| CD8A | CRP | -0.0257 | -0.1091 | 0.0576 | 0.5433 | 0.9292 |  |
| ARTN | CRP | 0.0143 | -0.0340 | 0.0626 | 0.5599 | 0.9292 |  |
| CCL28 | CRP | 0.0084 | -0.0200 | 0.0368 | 0.5600 | 0.9292 |  |
| AXIN1 | CRP | -0.0193 | -0.0848 | 0.0462 | 0.5614 | 0.9292 |  |
| IL.24 | CRP | -0.0200 | -0.0905 | 0.0505 | 0.5758 | 0.9292 |  |
| TNFB | CRP | 0.0122 | -0.0309 | 0.0553 | 0.5772 | 0.9292 |  |
| CDCP1 | CRP | 0.0126 | -0.0352 | 0.0605 | 0.6028 | 0.9292 |  |
| CXCL10 | CRP | 0.0226 | -0.0648 | 0.1099 | 0.6110 | 0.9292 |  |
| Beta.NGF | CRP | 0.0037 | -0.0110 | 0.0184 | 0.6184 | 0.9292 |  |
| SLAMF1 | CRP | 0.0113 | -0.0353 | 0.0579 | 0.6334 | 0.9292 |  |
| IL.10RB | CRP | 0.0125 | -0.0417 | 0.0668 | 0.6492 | 0.9292 |  |
| IL2 | CRP | 0.0091 | -0.0331 | 0.0513 | 0.6722 | 0.9292 |  |
| PD.L1 | CRP | 0.0125 | -0.0473 | 0.0723 | 0.6814 | 0.9292 |  |
| CXCL6 | CRP | 0.0166 | -0.0653 | 0.0985 | 0.6899 | 0.9292 |  |
| ADA | CRP | -0.0074 | -0.0445 | 0.0296 | 0.6924 | 0.9292 |  |
| CXCL1 | CRP | -0.0123 | -0.0748 | 0.0501 | 0.6974 | 0.9292 |  |
| CD244 | CRP | -0.0143 | -0.0894 | 0.0608 | 0.7070 | 0.9292 |  |
| OSM | CRP | -0.0155 | -0.1030 | 0.0721 | 0.7278 | 0.9292 |  |
| CX3CL1 | CRP | 0.0101 | -0.0487 | 0.0690 | 0.7346 | 0.9292 |  |
| CD5 | CRP | -0.0180 | -0.1239 | 0.0880 | 0.7384 | 0.9292 |  |
| IFN.gamma | CRP | 0.0239 | -0.1188 | 0.1666 | 0.7413 | 0.9292 |  |
| MCP.3 | CRP | 0.0171 | -0.0888 | 0.1230 | 0.7505 | 0.9292 |  |
| GDNF | CRP | -0.0078 | -0.0574 | 0.0418 | 0.7568 | 0.9292 |  |
| DNER | CRP | -0.0074 | -0.0585 | 0.0436 | 0.7739 | 0.9292 |  |
| CCL19 | CRP | 0.0097 | -0.0599 | 0.0793 | 0.7841 | 0.9292 |  |
| TNF | CRP | 0.0089 | -0.0620 | 0.0798 | 0.8057 | 0.9292 |  |
| CCL4 | CRP | 0.0099 | -0.0694 | 0.0891 | 0.8060 | 0.9292 |  |
| IL18 | CRP | 0.0100 | -0.0715 | 0.0915 | 0.8090 | 0.9292 |  |
| CCL25 | CRP | -0.0076 | -0.0712 | 0.0560 | 0.8142 | 0.9292 |  |
| IL.12B | CRP | -0.0098 | -0.0924 | 0.0728 | 0.8156 | 0.9292 |  |
| CD6 | CRP | -0.0112 | -0.1093 | 0.0870 | 0.8225 | 0.9292 |  |
| CSF.1 | CRP | 0.0054 | -0.0460 | 0.0568 | 0.8365 | 0.9292 |  |
| TRANCE | CRP | -0.0075 | -0.0821 | 0.0672 | 0.8435 | 0.9292 |  |
| CASP.8 | CRP | -0.0048 | -0.0536 | 0.0441 | 0.8478 | 0.9292 |  |
| FGF.21 | CRP | 0.0138 | -0.1321 | 0.1597 | 0.8523 | 0.9292 |  |
| IL5 | CRP | -0.0041 | -0.0482 | 0.0400 | 0.8557 | 0.9292 |  |
| IL13 | CRP | -0.0031 | -0.0376 | 0.0314 | 0.8585 | 0.9292 |  |
| Beta.NGF | IL1B | -0.0024 | -0.0197 | 0.0150 | 0.7893 | 0.9310 |  |
| CX3CL1 | IL17A | -0.0108 | -0.0704 | 0.0487 | 0.7198 | 0.9327 |  |
| FGF.5 | IL1B | 0.0076 | -0.0539 | 0.0691 | 0.8072 | 0.9336 |  |
| IL.1.alpha | IL1B | 0.0280 | -0.2036 | 0.2596 | 0.8118 | 0.9336 |  |
| STAMBP | CRP | 0.0014 | -0.0172 | 0.0201 | 0.8790 | 0.9349 |  |
| IL.18R1 | CRP | -0.0070 | -0.1055 | 0.0915 | 0.8889 | 0.9349 |  |
| CXCL11 | CRP | -0.0086 | -0.1384 | 0.1212 | 0.8961 | 0.9349 |  |
| NRTN | CRP | 0.0016 | -0.0278 | 0.0311 | 0.9124 | 0.9349 |  |
| TGF.alpha | CRP | 0.0038 | -0.0666 | 0.0742 | 0.9146 | 0.9349 |  |
| CXCL11 | IL1B | 0.0168 | -0.1358 | 0.1693 | 0.8287 | 0.9389 |  |
| IL.12B | IL1B | 0.0102 | -0.0871 | 0.1075 | 0.8368 | 0.9389 |  |
| SIRT2 | IL17A | -0.0051 | -0.0348 | 0.0247 | 0.7363 | 0.9407 |  |
| CD5 | IL17A | -0.0176 | -0.1251 | 0.0898 | 0.7464 | 0.9407 |  |
| IL2 | IL1B | 0.0048 | -0.0464 | 0.0560 | 0.8532 | 0.9430 |  |
| IL.20 | IL1B | 0.0031 | -0.0354 | 0.0417 | 0.8735 | 0.9430 |  |
| FGF.19 | IL1B | -0.0096 | -0.1301 | 0.1110 | 0.8757 | 0.9430 |  |
| IL13 | IL1B | 0.0029 | -0.0381 | 0.0440 | 0.8877 | 0.9430 |  |
| IL.24 | IL1B | -0.0057 | -0.0886 | 0.0771 | 0.8917 | 0.9430 |  |
| CXCL9 | CRP | -0.0028 | -0.0684 | 0.0628 | 0.9334 | 0.9437 |  |
| TNF | IL1B | -0.0045 | -0.0877 | 0.0788 | 0.9159 | 0.9504 |  |
| MMP.1 | IL1B | -0.0080 | -0.1629 | 0.1470 | 0.9194 | 0.9504 |  |
| uPA | IL6 | -0.0102 | -0.0814 | 0.0611 | 0.7788 | 0.9513 |  |
| CCL3 | IL6 | -0.0246 | -0.2009 | 0.1516 | 0.7832 | 0.9513 |  |
| IL.1.alpha | IL6 | 0.0273 | -0.1727 | 0.2272 | 0.7882 | 0.9513 |  |
| CD8A | IL6 | 0.0111 | -0.0735 | 0.0957 | 0.7954 | 0.9513 |  |
| IL.24 | IL6 | 0.0093 | -0.0622 | 0.0809 | 0.7973 | 0.9513 |  |
| CCL23 | IL6 | 0.0119 | -0.0843 | 0.1081 | 0.8079 | 0.9513 |  |
| IL.2RB | IL6 | -0.0039 | -0.0400 | 0.0323 | 0.8334 | 0.9513 |  |
| ARTN | IL6 | -0.0051 | -0.0541 | 0.0440 | 0.8390 | 0.9513 |  |
| MCP.2 | IL6 | 0.0092 | -0.0807 | 0.0991 | 0.8408 | 0.9513 |  |
| CXCL6 | IL6 | 0.0081 | -0.0751 | 0.0913 | 0.8479 | 0.9513 |  |
| TWEAK | IL1B | -0.0026 | -0.0631 | 0.0579 | 0.9330 | 0.9537 |  |
| CXCL10 | IL17A | -0.0127 | -0.1011 | 0.0756 | 0.7761 | 0.9554 |  |
| CD40 | IL17A | -0.0112 | -0.0899 | 0.0676 | 0.7802 | 0.9554 |  |
| LAP.TGF.beta.1 | IL17A | -0.0080 | -0.0667 | 0.0508 | 0.7892 | 0.9554 |  |
| LAP.TGF.beta.1 | IL1B | -0.0023 | -0.0705 | 0.0660 | 0.9480 | 0.9584 |  |
| TNFSF14 | IL6 | -0.0064 | -0.0934 | 0.0807 | 0.8851 | 0.9635 |  |
| IL13 | IL6 | -0.0024 | -0.0379 | 0.0330 | 0.8918 | 0.9635 |  |
| Flt3L | IL6 | 0.0040 | -0.0579 | 0.0659 | 0.8987 | 0.9635 |  |
| LIF | IL6 | -0.0019 | -0.0383 | 0.0345 | 0.9184 | 0.9635 |  |
| FGF.5 | IL6 | 0.0027 | -0.0504 | 0.0558 | 0.9206 | 0.9635 |  |
| STAMBP | IL6 | -0.0009 | -0.0198 | 0.0179 | 0.9216 | 0.9635 |  |
| IL.17A | IL6 | -0.0018 | -0.0447 | 0.0411 | 0.9347 | 0.9662 |  |
| IL.10RA | CRP | -0.0005 | -0.0288 | 0.0278 | 0.9731 | 0.9731 |  |
| ARTN | IL17A | -0.0055 | -0.0544 | 0.0435 | 0.8252 | 0.9740 |  |
| IL.20RA | IL17A | -0.0035 | -0.0384 | 0.0313 | 0.8417 | 0.9740 |  |
| GDNF | IL17A | 0.0051 | -0.0459 | 0.0561 | 0.8443 | 0.9740 |  |
| IL.18R1 | IL17A | 0.0093 | -0.0903 | 0.1089 | 0.8544 | 0.9740 |  |
| LIF | IL17A | -0.0033 | -0.0396 | 0.0331 | 0.8600 | 0.9740 |  |
| IFN.gamma | IL17A | -0.0108 | -0.1561 | 0.1345 | 0.8837 | 0.9740 |  |
| IL10 | IL17A | -0.0035 | -0.0535 | 0.0466 | 0.8916 | 0.9740 |  |
| IL.24 | IL17A | 0.0049 | -0.0664 | 0.0763 | 0.8919 | 0.9740 |  |
| FGF.5 | IL17A | -0.0029 | -0.0559 | 0.0500 | 0.9138 | 0.9740 |  |
| TRAIL | IL17A | -0.0031 | -0.0691 | 0.0630 | 0.9271 | 0.9740 |  |
| CCL23 | IL17A | -0.0036 | -0.0996 | 0.0923 | 0.9408 | 0.9740 |  |
| CXCL6 | IL17A | 0.0030 | -0.0799 | 0.0860 | 0.9429 | 0.9740 |  |
| HGF | IL17A | -0.0027 | -0.0861 | 0.0807 | 0.9495 | 0.9740 |  |
| MCP.2 | IL17A | 0.0023 | -0.0874 | 0.0920 | 0.9598 | 0.9740 |  |
| MCP.1 | IL17A | 0.0029 | -0.1221 | 0.1279 | 0.9634 | 0.9740 |  |
| IL33 | IL6 | -0.0008 | -0.0420 | 0.0404 | 0.9687 | 0.9802 |  |
| CD40 | IL6 | -0.0015 | -0.0805 | 0.0774 | 0.9696 | 0.9802 |  |
| GDNF | IL6 | 0.0006 | -0.0505 | 0.0517 | 0.9820 | 0.9820 |  |
| IL7 | IL17A | -0.0005 | -0.0419 | 0.0410 | 0.9829 | 0.9829 |  |
| MCP.2 | IL1B | 0.0004 | -0.1037 | 0.1045 | 0.9939 | 0.9939 |  |

# **Supplementary Table 7.** Regression estimates for associations between continuous maternal inflammation levels measured in the third trimester and neonatal inflammatory markers (n = 235), ordered by adjusted p-value

| **Neonatal  inflammatory marker** | **Maternal Inflammatory Marker** | **Beta** | **95% CI Low** | **95% CI High** | **p-value** | **Adjusted p-value** |
| --- | --- | --- | --- | --- | --- | --- |
| TWEAK | CRP | -0.076 | -0.133 | -0.019 | 0.010 | 0.487 |
| SCF | CRP | -0.076 | -0.137 | -0.014 | 0.017 | 0.487 |
| TRANCE | CRP | -0.077 | -0.146 | -0.007 | 0.031 | 0.487 |
| TNFSF14 | CRP | -0.097 | -0.184 | -0.009 | 0.031 | 0.487 |
| CCL4 | CRP | -0.084 | -0.161 | -0.007 | 0.032 | 0.487 |
| IL18 | CRP | 0.086 | -0.001 | 0.174 | 0.054 | 0.487 |
| TNFRSF9 | CRP | -0.067 | -0.135 | 0.001 | 0.054 | 0.487 |
| TNF | CRP | -0.070 | -0.141 | 0.001 | 0.055 | 0.487 |
| IL.10RB | CRP | -0.048 | -0.098 | 0.002 | 0.061 | 0.487 |
| CCL23 | CRP | -0.086 | -0.175 | 0.004 | 0.062 | 0.487 |
| IL.17C | CRP | -0.098 | -0.202 | 0.007 | 0.068 | 0.487 |
| IL4 | CRP | -0.036 | -0.077 | 0.005 | 0.081 | 0.487 |
| IL13 | CRP | -0.031 | -0.067 | 0.004 | 0.086 | 0.487 |
| VEGFA | CRP | -0.070 | -0.151 | 0.010 | 0.087 | 0.487 |
| TRAIL | CRP | -0.059 | -0.126 | 0.009 | 0.088 | 0.487 |
| LIF.R | CRP | -0.022 | -0.048 | 0.003 | 0.090 | 0.487 |
| CCL11 | CRP | -0.099 | -0.213 | 0.016 | 0.090 | 0.487 |
| LIF | CRP | -0.024 | -0.055 | 0.007 | 0.127 | 0.539 |
| CXCL6 | CRP | -0.068 | -0.157 | 0.021 | 0.132 | 0.539 |
| EN.RAGE | CRP | -0.059 | -0.136 | 0.018 | 0.133 | 0.539 |
| OSM | CRP | -0.072 | -0.168 | 0.023 | 0.135 | 0.539 |
| uPA | CRP | -0.053 | -0.123 | 0.018 | 0.144 | 0.539 |
| CD244 | CRP | -0.055 | -0.131 | 0.020 | 0.150 | 0.539 |
| CDCP1 | CRP | -0.042 | -0.099 | 0.016 | 0.154 | 0.539 |
| ADA | CRP | 0.026 | -0.010 | 0.062 | 0.160 | 0.539 |
| CD8A | CRP | -0.059 | -0.146 | 0.027 | 0.177 | 0.539 |
| PD.L1 | CRP | -0.044 | -0.108 | 0.021 | 0.185 | 0.539 |
| MMP.10 | CRP | -0.050 | -0.125 | 0.025 | 0.191 | 0.539 |
| IL.22.RA1 | CRP | -0.049 | -0.122 | 0.024 | 0.191 | 0.539 |
| Beta.NGF | CRP | -0.009 | -0.023 | 0.005 | 0.191 | 0.539 |
| IL.17A | CRP | -0.030 | -0.076 | 0.015 | 0.194 | 0.539 |
| MCP.3 | CRP | -0.062 | -0.155 | 0.032 | 0.197 | 0.539 |
| CD40 | CRP | -0.054 | -0.137 | 0.029 | 0.202 | 0.539 |
| CXCL10 | CRP | -0.060 | -0.152 | 0.033 | 0.204 | 0.539 |
| IL7 | CRP | -0.027 | -0.069 | 0.015 | 0.209 | 0.539 |
| SLAMF1 | CRP | -0.031 | -0.079 | 0.017 | 0.211 | 0.539 |
| IL6 | CRP | -0.070 | -0.182 | 0.042 | 0.218 | 0.541 |
| X4E.BP1 | CRP | -0.015 | -0.040 | 0.010 | 0.231 | 0.560 |
| CD5 | CRP | -0.062 | -0.166 | 0.043 | 0.245 | 0.567 |
| LAP.TGF.beta.1 | CRP | -0.035 | -0.093 | 0.024 | 0.246 | 0.567 |
| IL.20RA | CRP | -0.021 | -0.058 | 0.016 | 0.260 | 0.580 |
| ST1A1 | CRP | -0.038 | -0.104 | 0.029 | 0.265 | 0.580 |
| DNER | CRP | -0.027 | -0.077 | 0.023 | 0.294 | 0.629 |
| MCP.1 | CRP | -0.060 | -0.181 | 0.062 | 0.334 | 0.699 |
| CCL28 | IL17A | 0.050 | 0.013 | 0.087 | 0.009 | 0.699 |
| MCP.4 | IL17A | 0.117 | 0.014 | 0.219 | 0.026 | 0.699 |
| GDNF | IL17A | 0.049 | 0.001 | 0.097 | 0.046 | 0.699 |
| IL2 | IL17A | 0.042 | -0.001 | 0.084 | 0.055 | 0.699 |
| STAMBP | IL17A | 0.016 | -0.002 | 0.035 | 0.078 | 0.699 |
| VEGFA | IL17A | -0.067 | -0.145 | 0.012 | 0.097 | 0.699 |
| NRTN | IL17A | 0.026 | -0.005 | 0.057 | 0.101 | 0.699 |
| FGF.19 | IL17A | 0.082 | -0.018 | 0.182 | 0.107 | 0.699 |
| ST1A1 | IL17A | -0.053 | -0.117 | 0.012 | 0.108 | 0.699 |
| CCL25 | IL17A | -0.055 | -0.124 | 0.013 | 0.112 | 0.699 |
| HGF | IL17A | -0.070 | -0.159 | 0.018 | 0.119 | 0.699 |
| NT.3 | IL17A | 0.038 | -0.010 | 0.085 | 0.119 | 0.699 |
| IL33 | IL17A | 0.029 | -0.008 | 0.066 | 0.124 | 0.699 |
| TSLP | IL17A | -0.036 | -0.081 | 0.010 | 0.124 | 0.699 |
| IL5 | IL17A | 0.035 | -0.011 | 0.082 | 0.139 | 0.699 |
| IL.1.alpha | IL17A | 0.121 | -0.041 | 0.283 | 0.142 | 0.699 |
| CXCL10 | IL17A | 0.062 | -0.027 | 0.152 | 0.172 | 0.699 |
| MMP.10 | IL17A | 0.050 | -0.023 | 0.122 | 0.180 | 0.699 |
| MCP.2 | IL17A | 0.048 | -0.023 | 0.119 | 0.184 | 0.699 |
| FGF.23 | IL17A | 0.035 | -0.017 | 0.086 | 0.184 | 0.699 |
| LIF.R | IL17A | -0.017 | -0.042 | 0.008 | 0.185 | 0.699 |
| IL4 | IL17A | 0.027 | -0.013 | 0.067 | 0.188 | 0.699 |
| CST5 | IL17A | 0.079 | -0.040 | 0.197 | 0.193 | 0.699 |
| IL.20RA | IL17A | 0.024 | -0.012 | 0.060 | 0.198 | 0.699 |
| IL18 | IL17A | -0.056 | -0.141 | 0.030 | 0.202 | 0.699 |
| IL.10RB | IL17A | -0.032 | -0.081 | 0.017 | 0.202 | 0.699 |
| IL.17C | IL17A | 0.066 | -0.036 | 0.169 | 0.205 | 0.699 |
| FGF.5 | IL1B | 0.055 | 0.005 | 0.105 | 0.033 | 0.709 |
| CSF.1 | IL1B | -0.054 | -0.105 | -0.004 | 0.036 | 0.709 |
| IL.24 | IL1B | 0.073 | 0.003 | 0.144 | 0.041 | 0.709 |
| MCP.2 | IL1B | 0.073 | 0.000 | 0.146 | 0.049 | 0.709 |
| GDNF | IL1B | 0.050 | 0.000 | 0.099 | 0.051 | 0.709 |
| TNFRSF9 | IL1B | -0.068 | -0.138 | 0.001 | 0.053 | 0.709 |
| CCL28 | IL1B | 0.037 | -0.002 | 0.076 | 0.060 | 0.709 |
| IL.20 | IL1B | 0.029 | -0.002 | 0.060 | 0.065 | 0.709 |
| CCL3 | IL1B | 0.138 | -0.022 | 0.299 | 0.090 | 0.709 |
| ARTN | IL1B | 0.042 | -0.007 | 0.091 | 0.092 | 0.709 |
| IL7 | IL1B | 0.035 | -0.008 | 0.077 | 0.108 | 0.709 |
| CCL23 | IL1B | -0.073 | -0.164 | 0.018 | 0.113 | 0.709 |
| IL.10RB | IL1B | -0.041 | -0.091 | 0.010 | 0.118 | 0.709 |
| TSLP | IL1B | -0.038 | -0.085 | 0.010 | 0.119 | 0.709 |
| MMP.1 | IL1B | 0.099 | -0.026 | 0.225 | 0.120 | 0.709 |
| IFN.gamma | IL1B | -0.119 | -0.270 | 0.032 | 0.123 | 0.709 |
| CASP.8 | IL17A | -0.031 | -0.082 | 0.019 | 0.223 | 0.731 |
| CD6 | CRP | -0.045 | -0.143 | 0.053 | 0.365 | 0.741 |
| TGF.alpha | CRP | -0.035 | -0.114 | 0.043 | 0.375 | 0.741 |
| IL.2RB | CRP | -0.016 | -0.051 | 0.020 | 0.383 | 0.741 |
| IL2 | CRP | -0.019 | -0.063 | 0.025 | 0.403 | 0.741 |
| IL.18R1 | CRP | -0.040 | -0.136 | 0.055 | 0.406 | 0.741 |
| CXCL9 | CRP | -0.029 | -0.098 | 0.040 | 0.406 | 0.741 |
| CCL19 | CRP | -0.026 | -0.090 | 0.038 | 0.418 | 0.741 |
| CCL20 | CRP | 0.042 | -0.062 | 0.146 | 0.423 | 0.741 |
| NRTN | CRP | -0.013 | -0.045 | 0.019 | 0.427 | 0.741 |
| MCP.2 | IL6 | 0.087 | 0.015 | 0.160 | 0.018 | 0.765 |
| TNF | IL6 | 0.079 | 0.008 | 0.150 | 0.030 | 0.765 |
| CXCL9 | IL6 | 0.076 | 0.006 | 0.146 | 0.034 | 0.765 |
| IL.22.RA1 | IL6 | 0.077 | 0.005 | 0.150 | 0.037 | 0.765 |
| TWEAK | IL6 | 0.058 | 0.000 | 0.116 | 0.049 | 0.765 |
| HGF | IL6 | -0.085 | -0.176 | 0.006 | 0.066 | 0.765 |
| ADA | IL6 | 0.033 | -0.003 | 0.069 | 0.071 | 0.765 |
| IL.24 | IL6 | 0.063 | -0.007 | 0.133 | 0.079 | 0.765 |
| CX3CL1 | IL6 | 0.049 | -0.007 | 0.105 | 0.087 | 0.765 |
| IL.2RB | IL6 | 0.030 | -0.005 | 0.065 | 0.091 | 0.765 |
| GDNF | IL6 | 0.041 | -0.009 | 0.090 | 0.108 | 0.765 |
| CDCP1 | IL6 | 0.046 | -0.012 | 0.103 | 0.119 | 0.765 |
| IL.20 | IL6 | 0.024 | -0.007 | 0.055 | 0.126 | 0.765 |
| CCL28 | IL6 | 0.030 | -0.009 | 0.069 | 0.130 | 0.765 |
| CST5 | IL6 | 0.092 | -0.030 | 0.214 | 0.137 | 0.765 |
| IL5 | IL6 | 0.036 | -0.012 | 0.083 | 0.146 | 0.765 |
| FGF.21 | IL6 | -0.096 | -0.234 | 0.042 | 0.170 | 0.765 |
| CCL11 | IL6 | 0.080 | -0.035 | 0.196 | 0.170 | 0.765 |
| ARTN | IL6 | 0.034 | -0.015 | 0.082 | 0.173 | 0.765 |
| SCF | IL6 | 0.043 | -0.019 | 0.106 | 0.174 | 0.765 |
| IL.17C | IL6 | 0.072 | -0.033 | 0.178 | 0.178 | 0.765 |
| VEGFA | IL6 | -0.055 | -0.136 | 0.026 | 0.184 | 0.765 |
| TNFSF14 | IL6 | -0.058 | -0.147 | 0.030 | 0.195 | 0.765 |
| MCP.1 | IL6 | 0.078 | -0.044 | 0.200 | 0.209 | 0.765 |
| MCP.4 | IL6 | 0.067 | -0.039 | 0.173 | 0.214 | 0.765 |
| IL4 | IL6 | 0.026 | -0.015 | 0.067 | 0.216 | 0.765 |
| FGF.21 | IL17A | -0.079 | -0.213 | 0.056 | 0.250 | 0.765 |
| IL.24 | IL17A | -0.037 | -0.106 | 0.031 | 0.283 | 0.765 |
| IL.2RB | IL17A | 0.018 | -0.016 | 0.053 | 0.287 | 0.765 |
| SCF | IL17A | -0.033 | -0.094 | 0.028 | 0.290 | 0.765 |
| uPA | IL17A | -0.037 | -0.106 | 0.032 | 0.292 | 0.765 |
| MCP.3 | IL17A | 0.048 | -0.044 | 0.139 | 0.306 | 0.765 |
| LIF | IL17A | 0.016 | -0.015 | 0.046 | 0.311 | 0.765 |
| TNFSF14 | IL17A | -0.044 | -0.130 | 0.042 | 0.319 | 0.765 |
| IL.10RA | IL17A | -0.013 | -0.040 | 0.014 | 0.333 | 0.765 |
| CSF.1 | IL17A | -0.024 | -0.073 | 0.025 | 0.334 | 0.765 |
| DNER | IL17A | -0.024 | -0.073 | 0.025 | 0.335 | 0.765 |
| X4E.BP1 | IL17A | 0.011 | -0.013 | 0.035 | 0.366 | 0.765 |
| IL6 | IL17A | 0.050 | -0.059 | 0.159 | 0.369 | 0.765 |
| CX3CL1 | IL17A | 0.025 | -0.030 | 0.080 | 0.375 | 0.765 |
| TNFB | IL17A | -0.017 | -0.054 | 0.021 | 0.379 | 0.765 |
| OSM | IL17A | -0.041 | -0.134 | 0.052 | 0.384 | 0.765 |
| CCL3 | IL17A | 0.069 | -0.087 | 0.224 | 0.386 | 0.765 |
| CXCL9 | IL17A | 0.030 | -0.039 | 0.098 | 0.395 | 0.765 |
| SIRT2 | IL17A | 0.012 | -0.016 | 0.040 | 0.417 | 0.765 |
| CD244 | IL17A | -0.030 | -0.104 | 0.044 | 0.425 | 0.765 |
| IL13 | IL17A | 0.014 | -0.021 | 0.049 | 0.426 | 0.765 |
| IL.22.RA1 | IL17A | 0.029 | -0.043 | 0.100 | 0.430 | 0.765 |
| PD.L1 | IL17A | -0.025 | -0.088 | 0.038 | 0.432 | 0.765 |
| AXIN1 | IL17A | -0.030 | -0.105 | 0.046 | 0.437 | 0.765 |
| IL.18R1 | IL17A | -0.037 | -0.130 | 0.057 | 0.441 | 0.765 |
| IL8 | CRP | -0.041 | -0.149 | 0.068 | 0.460 | 0.775 |
| MMP.1 | CRP | -0.043 | -0.168 | 0.082 | 0.498 | 0.775 |
| CSF.1 | CRP | -0.017 | -0.068 | 0.033 | 0.500 | 0.775 |
| IL.12B | CRP | -0.026 | -0.105 | 0.052 | 0.507 | 0.775 |
| IL5 | CRP | 0.016 | -0.032 | 0.064 | 0.509 | 0.775 |
| IL.1.alpha | CRP | 0.055 | -0.111 | 0.222 | 0.512 | 0.775 |
| OPG | CRP | -0.019 | -0.077 | 0.039 | 0.514 | 0.775 |
| IFN.gamma | CRP | 0.049 | -0.100 | 0.197 | 0.520 | 0.775 |
| IL.24 | CRP | -0.023 | -0.093 | 0.047 | 0.522 | 0.775 |
| TNFRSF9 | IL17A | -0.025 | -0.092 | 0.043 | 0.472 | 0.793 |
| CDCP1 | IL17A | 0.020 | -0.036 | 0.076 | 0.487 | 0.793 |
| CCL4 | IL17A | 0.025 | -0.050 | 0.101 | 0.507 | 0.793 |
| EN.RAGE | IL17A | -0.025 | -0.101 | 0.050 | 0.509 | 0.793 |
| TNF | IL17A | -0.023 | -0.093 | 0.047 | 0.518 | 0.793 |
| Flt3L | IL17A | 0.019 | -0.041 | 0.079 | 0.540 | 0.793 |
| ADA | IL17A | 0.011 | -0.024 | 0.046 | 0.542 | 0.793 |
| CD5 | IL17A | -0.031 | -0.133 | 0.070 | 0.547 | 0.793 |
| FGF.5 | IL17A | 0.015 | -0.034 | 0.064 | 0.550 | 0.793 |
| MCP.1 | IL17A | 0.036 | -0.083 | 0.154 | 0.551 | 0.793 |
| LAP.TGF.beta.1 | IL17A | -0.017 | -0.075 | 0.040 | 0.552 | 0.793 |
| SLAMF1 | IL17A | 0.014 | -0.033 | 0.061 | 0.568 | 0.804 |
| IL.15RA | CRP | -0.013 | -0.057 | 0.031 | 0.564 | 0.812 |
| CST5 | CRP | -0.036 | -0.158 | 0.086 | 0.565 | 0.812 |
| ARTN | IL17A | 0.013 | -0.035 | 0.060 | 0.600 | 0.820 |
| CD8A | IL17A | 0.022 | -0.062 | 0.107 | 0.604 | 0.820 |
| IL7 | IL17A | 0.011 | -0.030 | 0.052 | 0.606 | 0.820 |
| TNFB | CRP | -0.011 | -0.049 | 0.028 | 0.588 | 0.825 |
| ARTN | CRP | 0.013 | -0.035 | 0.062 | 0.592 | 0.825 |
| HGF | CRP | -0.023 | -0.115 | 0.068 | 0.613 | 0.826 |
| FGF.19 | CRP | 0.026 | -0.077 | 0.129 | 0.617 | 0.826 |
| CX3CL1 | CRP | -0.014 | -0.071 | 0.042 | 0.620 | 0.826 |
| CASP.8 | CRP | -0.012 | -0.064 | 0.040 | 0.641 | 0.837 |
| IL10 | CRP | 0.010 | -0.034 | 0.054 | 0.646 | 0.837 |
| CCL20 | IL6 | 0.062 | -0.044 | 0.168 | 0.251 | 0.855 |
| IL.20 | IL17A | 0.007 | -0.023 | 0.037 | 0.644 | 0.858 |
| CCL28 | CRP | -0.008 | -0.047 | 0.031 | 0.673 | 0.860 |
| IL.20 | CRP | -0.006 | -0.037 | 0.025 | 0.692 | 0.864 |
| STAMBP | CRP | 0.004 | -0.015 | 0.023 | 0.700 | 0.864 |
| MCP.4 | CRP | -0.020 | -0.126 | 0.086 | 0.710 | 0.864 |
| IL.10RA | CRP | -0.005 | -0.033 | 0.023 | 0.714 | 0.864 |
| IL.15RA | IL6 | -0.024 | -0.069 | 0.020 | 0.284 | 0.874 |
| CASP.8 | IL6 | -0.027 | -0.079 | 0.025 | 0.309 | 0.874 |
| IL.10RA | IL6 | 0.014 | -0.014 | 0.042 | 0.314 | 0.874 |
| FGF.5 | IL6 | 0.026 | -0.025 | 0.076 | 0.316 | 0.874 |
| ST1A1 | IL6 | -0.034 | -0.100 | 0.033 | 0.320 | 0.874 |
| LAP.TGF.beta.1 | IL6 | 0.030 | -0.029 | 0.089 | 0.323 | 0.874 |
| IL10 | IL6 | 0.022 | -0.022 | 0.066 | 0.325 | 0.874 |
| CXCL6 | IL6 | -0.044 | -0.134 | 0.045 | 0.333 | 0.874 |
| CXCL1 | CRP | -0.012 | -0.085 | 0.060 | 0.742 | 0.886 |
| CXCL5 | CRP | 0.021 | -0.108 | 0.150 | 0.751 | 0.886 |
| FGF.5 | CRP | -0.007 | -0.057 | 0.043 | 0.773 | 0.888 |
| CCL3 | CRP | -0.023 | -0.183 | 0.137 | 0.778 | 0.888 |
| SIRT2 | CRP | 0.004 | -0.025 | 0.033 | 0.782 | 0.888 |
| TGF.alpha | IL17A | -0.016 | -0.092 | 0.060 | 0.680 | 0.889 |
| Beta.NGF | IL17A | 0.003 | -0.011 | 0.016 | 0.700 | 0.889 |
| CD6 | IL17A | -0.018 | -0.114 | 0.077 | 0.704 | 0.889 |
| CCL20 | IL17A | -0.019 | -0.122 | 0.084 | 0.717 | 0.889 |
| IL10 | IL17A | 0.008 | -0.035 | 0.050 | 0.724 | 0.889 |
| IL.12B | IL17A | -0.014 | -0.090 | 0.063 | 0.726 | 0.889 |
| CXCL1 | IL17A | -0.011 | -0.082 | 0.060 | 0.753 | 0.889 |
| CXCL5 | IL17A | 0.020 | -0.106 | 0.145 | 0.757 | 0.889 |
| TWEAK | IL17A | 0.009 | -0.048 | 0.066 | 0.759 | 0.889 |
| CCL19 | IL17A | 0.010 | -0.053 | 0.072 | 0.763 | 0.889 |
| CCL23 | IL6 | -0.039 | -0.130 | 0.051 | 0.392 | 0.889 |
| Beta.NGF | IL6 | -0.006 | -0.020 | 0.008 | 0.396 | 0.889 |
| CCL19 | IL6 | 0.028 | -0.037 | 0.092 | 0.398 | 0.889 |
| Flt3L | IL6 | 0.024 | -0.038 | 0.086 | 0.439 | 0.889 |
| CXCL10 | IL6 | 0.035 | -0.057 | 0.128 | 0.453 | 0.889 |
| TSLP | IL6 | 0.018 | -0.029 | 0.065 | 0.459 | 0.889 |
| STAMBP | IL6 | 0.007 | -0.012 | 0.026 | 0.480 | 0.889 |
| IL7 | IL6 | 0.015 | -0.028 | 0.057 | 0.496 | 0.889 |
| MCP.3 | IL6 | 0.032 | -0.062 | 0.126 | 0.501 | 0.889 |
| CXCL5 | IL6 | -0.044 | -0.173 | 0.085 | 0.506 | 0.889 |
| NT.3 | IL6 | -0.016 | -0.065 | 0.033 | 0.524 | 0.889 |
| TRANCE | IL6 | 0.023 | -0.048 | 0.093 | 0.524 | 0.889 |
| IL.12B | IL6 | 0.025 | -0.053 | 0.104 | 0.528 | 0.889 |
| TNFRSF9 | IL6 | -0.022 | -0.091 | 0.047 | 0.531 | 0.889 |
| uPA | IL6 | -0.023 | -0.094 | 0.049 | 0.533 | 0.889 |
| CXCL11 | IL6 | 0.036 | -0.078 | 0.150 | 0.535 | 0.889 |
| IL.10RB | IL6 | -0.016 | -0.067 | 0.035 | 0.541 | 0.889 |
| IL.1.alpha | IL6 | 0.049 | -0.118 | 0.216 | 0.562 | 0.889 |
| OSM | IL6 | -0.028 | -0.124 | 0.068 | 0.566 | 0.889 |
| PD.L1 | IL6 | -0.019 | -0.084 | 0.046 | 0.568 | 0.889 |
| IL.18R1 | IL6 | -0.028 | -0.124 | 0.069 | 0.571 | 0.889 |
| LIF.R | IL6 | 0.007 | -0.018 | 0.033 | 0.574 | 0.889 |
| SIRT2 | IL6 | 0.008 | -0.021 | 0.037 | 0.590 | 0.889 |
| CD8A | IL6 | 0.024 | -0.064 | 0.111 | 0.595 | 0.889 |
| IL2 | IL6 | 0.012 | -0.032 | 0.056 | 0.595 | 0.889 |
| CD6 | IL6 | 0.026 | -0.072 | 0.124 | 0.598 | 0.889 |
| FGF.23 | IL6 | 0.014 | -0.039 | 0.067 | 0.599 | 0.889 |
| FGF.19 | IL6 | 0.026 | -0.077 | 0.130 | 0.617 | 0.891 |
| CD40 | IL6 | 0.021 | -0.062 | 0.104 | 0.620 | 0.891 |
| CXCL6 | IL17A | -0.012 | -0.099 | 0.075 | 0.785 | 0.892 |
| IL.17A | IL17A | 0.006 | -0.039 | 0.051 | 0.786 | 0.892 |
| CXCL11 | CRP | -0.015 | -0.129 | 0.099 | 0.796 | 0.893 |
| FGF.21 | CRP | 0.017 | -0.121 | 0.155 | 0.808 | 0.896 |
| IFN.gamma | IL17A | 0.018 | -0.128 | 0.165 | 0.804 | 0.899 |
| TRAIL | IL17A | 0.008 | -0.058 | 0.074 | 0.811 | 0.899 |
| TGF.alpha | IL6 | -0.019 | -0.097 | 0.060 | 0.638 | 0.900 |
| EN.RAGE | IL6 | -0.018 | -0.096 | 0.060 | 0.646 | 0.900 |
| IL.15RA | IL1B | -0.032 | -0.076 | 0.013 | 0.167 | 0.903 |
| DNER | IL6 | -0.011 | -0.062 | 0.039 | 0.660 | 0.904 |
| SLAMF1 | IL6 | 0.011 | -0.038 | 0.059 | 0.668 | 0.904 |
| CD40 | IL17A | -0.008 | -0.089 | 0.072 | 0.837 | 0.913 |
| TRANCE | IL17A | 0.007 | -0.062 | 0.075 | 0.844 | 0.913 |
| CD244 | IL6 | -0.016 | -0.092 | 0.061 | 0.686 | 0.915 |
| IL8 | IL6 | 0.022 | -0.088 | 0.131 | 0.699 | 0.918 |
| CD40 | IL1B | -0.056 | -0.139 | 0.028 | 0.190 | 0.919 |
| CCL11 | IL1B | -0.073 | -0.189 | 0.043 | 0.215 | 0.919 |
| FGF.21 | IL1B | -0.087 | -0.226 | 0.052 | 0.217 | 0.919 |
| CCL19 | IL1B | -0.040 | -0.105 | 0.025 | 0.224 | 0.919 |
| CDCP1 | IL1B | 0.036 | -0.022 | 0.094 | 0.224 | 0.919 |
| IL.12B | IL1B | -0.047 | -0.126 | 0.031 | 0.237 | 0.919 |
| Beta.NGF | IL1B | -0.008 | -0.023 | 0.006 | 0.242 | 0.919 |
| TNFB | IL1B | -0.023 | -0.061 | 0.016 | 0.250 | 0.919 |
| IL.10RA | IL1B | 0.015 | -0.013 | 0.043 | 0.282 | 0.921 |
| MCP.3 | IL1B | -0.052 | -0.146 | 0.043 | 0.284 | 0.921 |
| uPA | IL1B | -0.037 | -0.108 | 0.035 | 0.310 | 0.921 |
| FGF.23 | IL1B | -0.026 | -0.080 | 0.027 | 0.328 | 0.921 |
| CD5 | IL1B | -0.052 | -0.157 | 0.053 | 0.334 | 0.921 |
| IL33 | IL1B | -0.018 | -0.057 | 0.020 | 0.344 | 0.921 |
| MCP.1 | IL1B | -0.058 | -0.181 | 0.065 | 0.351 | 0.921 |
| LIF.R | IL1B | -0.012 | -0.038 | 0.014 | 0.372 | 0.921 |
| TWEAK | IL1B | 0.026 | -0.032 | 0.085 | 0.378 | 0.921 |
| CCL25 | IL1B | -0.032 | -0.103 | 0.039 | 0.379 | 0.921 |
| CXCL5 | IL1B | 0.056 | -0.074 | 0.186 | 0.396 | 0.921 |
| STAMBP | IL1B | 0.008 | -0.011 | 0.027 | 0.399 | 0.921 |
| IL.2RB | IL1B | -0.015 | -0.050 | 0.021 | 0.408 | 0.921 |
| CASP.8 | IL1B | 0.022 | -0.030 | 0.074 | 0.412 | 0.921 |
| DNER | IL1B | -0.021 | -0.072 | 0.030 | 0.413 | 0.921 |
| CX3CL1 | IL1B | 0.023 | -0.034 | 0.080 | 0.422 | 0.921 |
| IL.18R1 | IL1B | -0.039 | -0.135 | 0.058 | 0.433 | 0.921 |
| IL6 | IL1B | -0.043 | -0.156 | 0.071 | 0.461 | 0.921 |
| PD.L1 | IL1B | -0.024 | -0.090 | 0.041 | 0.463 | 0.921 |
| CCL4 | IL1B | -0.029 | -0.107 | 0.049 | 0.470 | 0.921 |
| CD244 | IL1B | -0.028 | -0.104 | 0.049 | 0.476 | 0.921 |
| EN.RAGE | IL1B | -0.028 | -0.107 | 0.050 | 0.479 | 0.921 |
| LAP.TGF.beta.1 | IL1B | 0.020 | -0.040 | 0.079 | 0.513 | 0.921 |
| CCL20 | IL1B | -0.035 | -0.142 | 0.072 | 0.517 | 0.921 |
| IL10 | IL1B | -0.014 | -0.059 | 0.030 | 0.522 | 0.921 |
| Flt3L | IL1B | 0.019 | -0.043 | 0.081 | 0.550 | 0.921 |
| IL.20RA | IL1B | -0.011 | -0.049 | 0.026 | 0.554 | 0.921 |
| IL.17A | IL1B | 0.014 | -0.033 | 0.060 | 0.565 | 0.921 |
| SIRT2 | IL1B | 0.008 | -0.021 | 0.037 | 0.575 | 0.921 |
| IL13 | IL1B | -0.010 | -0.046 | 0.026 | 0.584 | 0.921 |
| CD6 | IL1B | -0.027 | -0.126 | 0.072 | 0.589 | 0.921 |
| CXCL10 | IL1B | 0.024 | -0.069 | 0.117 | 0.610 | 0.921 |
| LIF | IL1B | -0.008 | -0.040 | 0.023 | 0.611 | 0.921 |
| NT.3 | IL1B | -0.012 | -0.062 | 0.037 | 0.630 | 0.921 |
| ADA | IL1B | -0.009 | -0.046 | 0.028 | 0.631 | 0.921 |
| SCF | IL1B | -0.015 | -0.078 | 0.048 | 0.642 | 0.921 |
| IL2 | IL1B | -0.010 | -0.055 | 0.034 | 0.646 | 0.921 |
| IL4 | IL1B | 0.009 | -0.032 | 0.051 | 0.658 | 0.921 |
| MMP.10 | IL1B | -0.017 | -0.092 | 0.059 | 0.665 | 0.921 |
| FGF.19 | IL1B | 0.023 | -0.081 | 0.127 | 0.666 | 0.921 |
| TGF.alpha | IL1B | -0.017 | -0.096 | 0.062 | 0.671 | 0.921 |
| TNFSF14 | IL1B | -0.018 | -0.108 | 0.071 | 0.688 | 0.921 |
| IL18 | IL1B | 0.018 | -0.071 | 0.107 | 0.696 | 0.921 |
| CD8A | IL1B | -0.017 | -0.105 | 0.070 | 0.696 | 0.921 |
| IL5 | IL1B | 0.009 | -0.039 | 0.058 | 0.703 | 0.921 |
| VEGFA | IL1B | 0.014 | -0.068 | 0.096 | 0.729 | 0.921 |
| IL.1.alpha | IL1B | 0.029 | -0.139 | 0.198 | 0.732 | 0.921 |
| CXCL9 | IL1B | 0.012 | -0.059 | 0.083 | 0.741 | 0.921 |
| NRTN | IL1B | -0.005 | -0.037 | 0.028 | 0.769 | 0.921 |
| OPG | IL1B | -0.008 | -0.067 | 0.050 | 0.778 | 0.921 |
| IL.22.RA1 | IL1B | 0.011 | -0.063 | 0.085 | 0.778 | 0.921 |
| CXCL1 | IL1B | 0.010 | -0.063 | 0.084 | 0.786 | 0.921 |
| HGF | IL1B | -0.012 | -0.105 | 0.080 | 0.792 | 0.921 |
| AXIN1 | IL1B | -0.010 | -0.088 | 0.068 | 0.796 | 0.921 |
| IL.17C | IL1B | 0.013 | -0.093 | 0.120 | 0.804 | 0.921 |
| TNF | IL1B | -0.009 | -0.081 | 0.064 | 0.811 | 0.921 |
| NT.3 | CRP | -0.005 | -0.054 | 0.044 | 0.846 | 0.927 |
| CCL3 | IL6 | -0.030 | -0.190 | 0.131 | 0.716 | 0.928 |
| OPG | IL17A | -0.005 | -0.061 | 0.052 | 0.872 | 0.929 |
| IL8 | IL17A | -0.008 | -0.114 | 0.098 | 0.878 | 0.929 |
| ST1A1 | IL1B | -0.007 | -0.074 | 0.060 | 0.832 | 0.934 |
| CCL4 | IL6 | 0.013 | -0.064 | 0.091 | 0.734 | 0.937 |
| IL8 | IL1B | -0.011 | -0.121 | 0.099 | 0.847 | 0.939 |
| IL.15RA | IL17A | -0.003 | -0.046 | 0.041 | 0.902 | 0.941 |
| MMP.1 | IL17A | 0.007 | -0.115 | 0.129 | 0.910 | 0.941 |
| TRANCE | IL1B | -0.006 | -0.077 | 0.065 | 0.876 | 0.959 |
| TNFB | IL6 | 0.005 | -0.033 | 0.044 | 0.787 | 0.960 |
| IL13 | IL6 | 0.005 | -0.031 | 0.041 | 0.794 | 0.960 |
| IL6 | IL6 | 0.015 | -0.098 | 0.128 | 0.797 | 0.960 |
| IL.20RA | IL6 | 0.004 | -0.033 | 0.042 | 0.828 | 0.960 |
| AXIN1 | IL6 | -0.009 | -0.086 | 0.069 | 0.828 | 0.960 |
| CXCL1 | IL6 | -0.008 | -0.081 | 0.065 | 0.830 | 0.960 |
| CD5 | IL6 | 0.011 | -0.093 | 0.116 | 0.831 | 0.960 |
| CSF.1 | IL6 | 0.005 | -0.045 | 0.056 | 0.835 | 0.960 |
| CXCL6 | IL1B | -0.006 | -0.096 | 0.084 | 0.895 | 0.961 |
| TRAIL | IL1B | 0.004 | -0.064 | 0.073 | 0.898 | 0.961 |
| IL18 | IL6 | -0.008 | -0.096 | 0.080 | 0.857 | 0.970 |
| MMP.1 | IL6 | -0.011 | -0.136 | 0.115 | 0.864 | 0.970 |
| CCL11 | IL17A | 0.004 | -0.109 | 0.116 | 0.949 | 0.970 |
| CXCL11 | IL1B | -0.006 | -0.121 | 0.109 | 0.919 | 0.972 |
| CCL25 | CRP | -0.004 | -0.075 | 0.066 | 0.906 | 0.974 |
| IL33 | CRP | -0.002 | -0.039 | 0.036 | 0.927 | 0.974 |
| TSLP | CRP | -0.002 | -0.049 | 0.045 | 0.933 | 0.974 |
| FGF.23 | CRP | -0.002 | -0.055 | 0.050 | 0.936 | 0.974 |
| AXIN1 | CRP | -0.003 | -0.080 | 0.074 | 0.942 | 0.974 |
| MCP.2 | CRP | -0.002 | -0.075 | 0.071 | 0.953 | 0.974 |
| NRTN | IL6 | 0.002 | -0.030 | 0.035 | 0.884 | 0.980 |
| Flt3L | CRP | -0.001 | -0.063 | 0.061 | 0.974 | 0.980 |
| GDNF | CRP | 0.001 | -0.049 | 0.050 | 0.980 | 0.980 |
| X4E.BP1 | IL1B | -0.001 | -0.026 | 0.024 | 0.946 | 0.987 |
| OSM | IL1B | 0.003 | -0.094 | 0.099 | 0.955 | 0.987 |
| TRAIL | IL6 | 0.003 | -0.065 | 0.072 | 0.920 | 0.989 |
| IFN.gamma | IL6 | 0.007 | -0.144 | 0.158 | 0.928 | 0.989 |
| IL33 | IL6 | -0.002 | -0.040 | 0.036 | 0.929 | 0.989 |
| MMP.10 | IL6 | 0.002 | -0.073 | 0.077 | 0.956 | 0.989 |
| OPG | IL6 | -0.001 | -0.060 | 0.057 | 0.967 | 0.989 |
| CCL25 | IL6 | -0.001 | -0.072 | 0.070 | 0.974 | 0.989 |
| IL.17A | IL6 | -0.001 | -0.047 | 0.045 | 0.976 | 0.989 |
| X4E.BP1 | IL6 | 0.000 | -0.025 | 0.025 | 0.979 | 0.989 |
| LIF | IL6 | 0.000 | -0.031 | 0.032 | 0.989 | 0.989 |
| CXCL11 | IL17A | -0.001 | -0.112 | 0.110 | 0.985 | 0.993 |
| CCL23 | IL17A | 0.000 | -0.089 | 0.088 | 0.993 | 0.993 |
| MCP.4 | IL1B | -0.001 | -0.109 | 0.106 | 0.978 | 0.998 |
| SLAMF1 | IL1B | 0.000 | -0.048 | 0.049 | 0.987 | 0.998 |
| CST5 | IL1B | 0.000 | -0.124 | 0.123 | 0.998 | 0.998 |

# **Supplementary Table 8.** Maternal inflammatory marker characteristics in restricted temporal proximity by inflammation levels

| **Characteristic** | **Overall** | **IL-1β closer temporal proximity** | | **IL-6 closer temporal proximity** | | **IL-17a closer temporal proximity** | | **CRP closer temporal proximity** | |
| --- | --- | --- | --- | --- | --- | --- | --- | --- | --- |
|  | **N=105** | High  (N=26^1^) | Low  (N=79^1^) | High  (N=26^1^) | Low  (N=79^1^) | High  (N=26^1^) | Low  (N=79^1^) | High  (N=26^1^) | Low  (N=79^1^) |
| **Maternal age at delivery (years)** | 33 (30, 36) | 33 (30, 36) | 34 (31, 36) | 34 (30, 36) | 33 (31, 36) | 33 (30, 34) | 34 (31, 36) | 33 (31, 36) | 34 (30, 36) |
| **Race-ethnicity** |  |  |  |  |  |  |  | ** | |
| Asian | 14% (15) | 19% (5) | 13% (10) | 15% (4) | 14% (11) | 12% (3) | 15% (12) | 12% (3) | 15% (12) |
| Black | 10% (10) | 4% (1) | 11% (9) | 4% (1) | 11% (9) | 7.7% (2) | 10% (8) | 12% (3) | 9% (7) |
| Hispanic | 18% (19) | 12% (3) | 20% (16) | 19% (5) | 18% (14) | 31% (8) | 14% (11) | 27% (7) | 15% (12) |
| White | 51% (54) | 58% (15) | 49% (39) | 54% (14) | 51% (40) | 42% (11) | 54% (43) | 31% (8) | 58% (46) |
| Other | 7% (7) | 8% (2) | 6.3% (5) | 8% (2) | 6% (5) | 8% (2) | 6% (5) | 19% (5) | 3% (2) |
| **Parity** |  | *** | |  |  |  | |  |  |
| Nulliparous | 37% (39) | 62% (16) | 29% (23) | 31% (8) | 39% (31) | 50% (13) | 33% (26) | 42% (11) | 35% (28) |
| Multiparous | 63% (66) | 38% (10) | 71% (56) | 69% (18) | 61% (48) | 50% (13) | 67% (53) | 58% (15) | 65% (51) |
| **Education** |  |  |  |  |  |  |  |  |  |
| Less than college | 21% (18) | 12% (3) | 24% (15) | 14% (3) | 23% (15) | 20% (5) | 21% (13) | 30% (6) | 18% (12) |
| $\geq$College | 79% (69) | 88% (22) | 76% (47) | 86% (19) | 77% (50) | 80% (20) | 79% (49) | 70% (14) | 82% (55) |
| Missing | 18 | 1 | 17 | 4 | 14 | 1 | 17 | 6 | 12 |
| **Child sex** |  |  |  |  |  | * | |  |  |
| Female | 51% (54) | 54% (14) | 51% (40) | 46% (12) | 53% (42) | 69% (18) | 46% (36) | 46% (12) | 53% (42) |
| Male | 49% (51) | 46% (12) | 49% (39) | 54% (14) | 47% (37) | 31% (8) | 54% (43) | 54% (14) | 47% (37) |
| **Maternal history of mental illness** | | ** | |  |  |  |  | * | |
| No | 59% (62) | 85% (22) | 51% (40) | 65% (17) | 57% (45) | 65% (17) | 57% (45) | 42% (11) | 65% (51) |
| Yes | 41% (43) | 15% (4) | 49% (39) | 35% (9) | 43% (34) | 35% (9) | 43% (34) | 58% (15) | 35% (28) |
| **SARS-CoV-2 infection during pregnancy** | |  |  |  |  |  |  |  |  |
| No | 94% (99) | 100% (26) | 92% (73) | 100% (26) | 92% (73) | 100% (26) | 92% (73) | 92% (24) | 95% (75) |
| Yes | 6% (6) | 0% (0) | 8% (6) | 0% (0) | 8 % (6) | 0% (0) | 8% (6) | 8 % (2) | 5 % (4) |
| **Neonate age at DBS collection (hrs)** |  |  |  |  |  |  | |  |  |
|  | 24 (24, 24) | 24 (24, 24) | 24 (24, 25) | 24 (24, 24) | 24 (24, 25) | 24 (24, 25) | 24 (24, 24) | 24 (24, 25) | 24 (24, 24) |
| **Pre-pregnancy BMI (kg/m^2^)** |  |  |  |  |  |  |  | * | |
|  | 25 (22, 29) | 26 (23, 29) | 25 (22, 29) | 26 (23, 30) | 25 (22, 28) | 26 (23, 28) | 25 (22, 29) | 27 (23, 31) | 25 (21, 28) |
| **Delivery Mode** |  | ** | |  |  |  |  |  |  |
| C-section | 44% (46) | 23% (6) | 51% (40) | 35% (9) | 47% (37) | 50% (13) | 42% (33) | 46% (12) | 43% (34) |
| Vaginal Delivery | 56% (59) | 77% (20) | 49% (39) | 65% (17) | 53% (42) | 50% (13) | 58% (46) | 54% (14) | 57% (45) |
| **Cardiometabolic disorders of pregnancy** | |  |  |  |  |  |  |  |  |
| No | 69% (72) | 77% (20) | 66% (52) | 69% (18) | 68% (54) | 69% (18) | 68% (54) | 69% (18) | 68% (54) |
| Yes | 31% (33) | 23% (6) | 34% (27) | 31% (8) | 32% (25) | 31% (8) | 32% (25) | 31% (8) | 32% (25) |
| **Gestational age at delivery** (days) |  |  | |  |  |  |  |  |  |
|  | 274  (271, 278) | 276  (273, 281) | 274  (271, 277) | 274  (272, 279) | 274  (271, 278) | 274  (268, 276) | 275  (273, 278) | 274  (270, 276) | 275  (272, 279) |
| **Preterm Birth** |  |  |  |  |  |  |  |  |  |
| No | 99% (104) | 100% (28) | 99% (76) | 100% (26) | 99% (78) | 100% (26) | 99% (78) | 100% (26) | 99% (78) |
| Yes | 1 % (1) | 0% (0) | 1 % (1) | 0% (0) | 1 % (1) | 0% (0) | 1 % (1) | 0% (0) | 1 % (1) |
| **Birthweight (grams)** | 3,260 (3,020, 3,605) | 3,235 (3,010, 3,640) | 3,260 (3,055, 3,600) | 3,240 (3,107, 3,587) | 3,260 (3,000, 3,605) | 3,233 (3,005, 3,485) | 3,270 (3,020, 3,665) | 3,230 (3,040, 3,665) | 3,290 (3,020, 3,660) |

¹ Median (IQR); % (n). Note: percentages may not sum to 100% due to rounding.

^†^*p* < 0.05 (**), p < 0.01 (**), p < 0.001 (****). *P*-values are based on Wilcoxon rank-sum tests for continuous variables and Chi-square or Fisher’s exact tests for categorical variables, comparing high vs. low groups within each maternal marker.

# **Supplementary Table 9.** Maternal inflammatory marker characteristics in restricted temporal proximity analysis by inflammation levels

| **Characteristic**^†^ | **IL-1β in closer temporal proximity** | | **IL-6 in closer temporal proximity** | | **IL-17a in in closer temporal proximity** | | **CRP in closer temporal proximity** | |
| --- | --- | --- | --- | --- | --- | --- | --- | --- |
|  | High  (N=26^1^) | Low  (N=79^1^) | High  (N=26^1^) | Low  (N=79^1^) | High  (N=26^1^) | Low  (N=79^1^) | High  (N=26^1^) | Low  (N=79^1^) |
| **IL-1β log2** | *** | |  |  | * | |  |  |
|  | 3.93  (3.16, 5.30) | 0.79  (0.11, 1.51) | 1.63  (0.79, 3.87) | 0.86  (0.26, 2.46) | 1.67  (0.80, 2.92) | 0.86  (0.11, 2.52) | 1.11  (0.00, 2.63) | 1.07  (0.65, 2.86) |
| **IL-6 log2** |  |  | *** | |  | |  |  |
|  | 1.15  (-0.18, 3.00) | 0.43  (-0.40, 2.19) | 3.28  (2.60, 3.72) | 0.10  (-0.51, 0.99) | 1.43  (0.07, 3.33) | 0.40  (-0.40, 2.05) | 1.39  (0.07, 2.47) | 0.39  (-0.40, 2.05) |
| **IL-17a log2** |  |  |  |  |  |  |  |  |
|  | 3.27  (3.01, 3.91) | 3.32  (2.77, 3.62) | 3.44  (2.93, 4.02) | 3.26  (2.77, 3.60) | 4.05  (3.81, 4.51) | 3.05  (2.68, 3.37) | 3.20  (2.63, 3.91) | 3.33  (2.85, 3.63) |
| **CRP log2** |  |  |  |  |  |  | *** | |
|  | 14.05 (13.19, 14.53) | 13.95 (13.34, 14.62) | 13.93 (12.84, 14.89) | 14.08 (13.36, 14.58) | 14.19 (13.66, 14.89) | 13.95 (13.20, 14.58) | 15.25 (14.85, 15.94) | 13.74 (13.07, 14.16) |
| **IL-1β category** |  |  |  |  |  |  |  |  |
| High | -- | -- | 35% (9) | 22% (17) | 31% (8) | 23% (18) | 23% (6) | 25% (20) |
| Low | -- | -- | 65% (17) | 78% (62) | 69% (18) | 77% (61) | 77% (20) | 75% (59) |
| **IL-6 category** |  |  |  |  |  |  |  |  |
| High | 35% (9) | 22% (17) | -- | -- | 38% (10) | 20% (16) | 31% (8) | 23% (18) |
| Low | 65% (17) | 78% (62) | -- | -- | 62% (16) | 80% (63) | 69% (18) | 77% (61) |
| **IL-17a category** |  |  |  |  |  |  |  |  |
| High | 31% (8) | 23% (18) | 38% (10) | 20% (16) | -- | -- | 31% (8) | 23% (18) |
| Low | 69% (18) | 77% (61) | 62% (16) | 80% (63) | -- | -- | 69% (18) | 77% (61) |
| **CRP category** |  |  |  |  |  |  |  |  |
| High | 23% (6) | 25% (20) | 31% (8) | 23% (18) | 31% (8) | 23% (18) | -- | -- |
| Low | 77% (20) | 75% (59) | 69% (18) | 77% (61) | 69% (18) | 77% (61) | -- | -- |

¹ Median (IQR); % (n). Note: percentages may not sum to 100% due to rounding.

^†^Values for IL-1β, IL-6, and IL-17a are log2-transformed concentrations (pg/mL); CRP is reported in mg/L.

*p* < 0.05 (**), p < 0.01 (**), p < 0.001 (****). *P*-values are based on Wilcoxon rank-sum tests for continuous variables and Chi-square or Fisher’s exact tests for categorical variables, comparing high vs. low groups within each maternal marker.


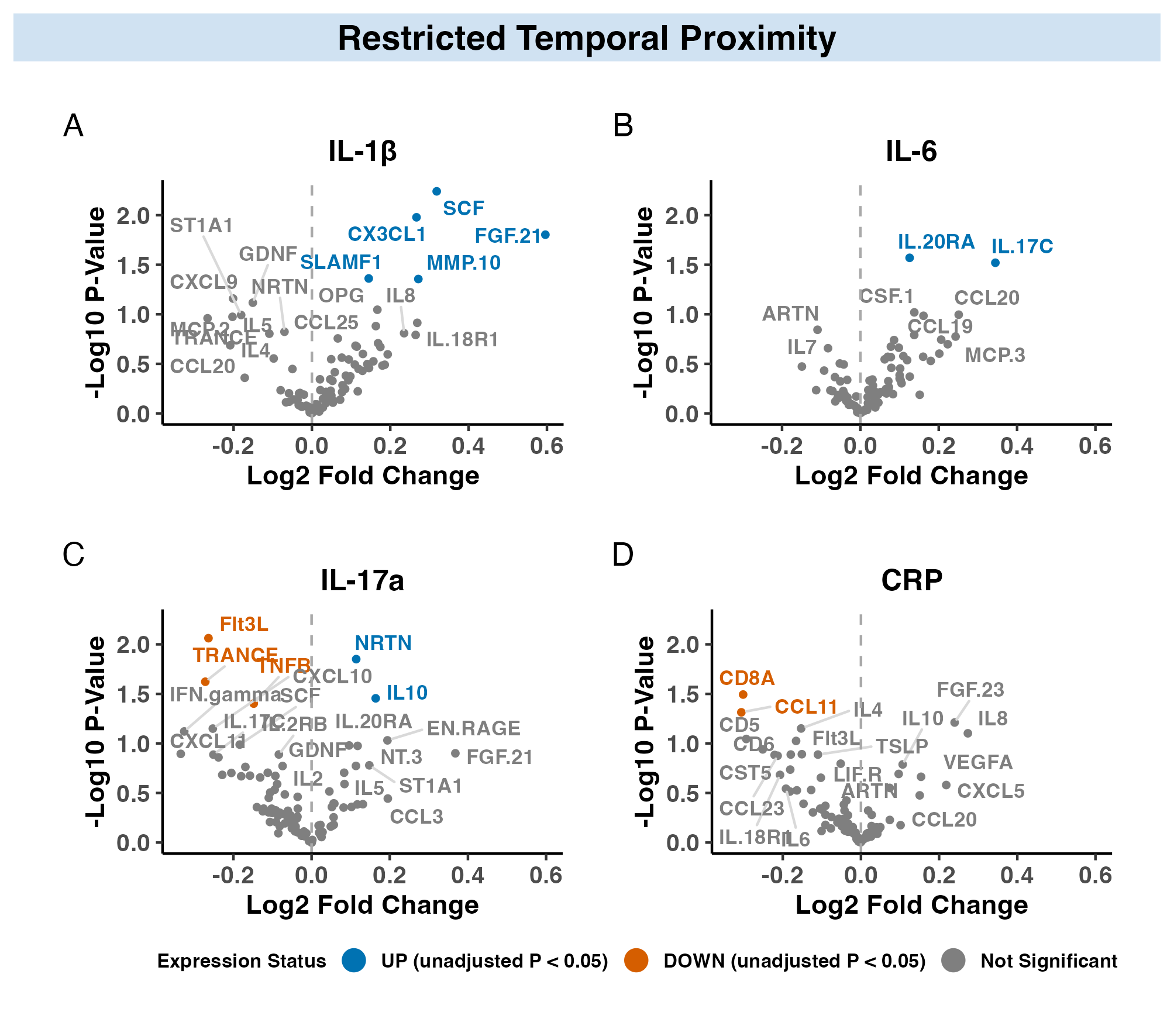


# **Supplementary Figure 2.** Volcano plots for differential expression of neonatal inflammatory markers relative to maternal inflammation levels at birth in first sensitivity analysis comparing high vs. low maternal inflammation groups for each maternal marker: IL-1β (A), IL-6 (B), IL-17a (C), and CRP (D) in a subset of n=105 with maternal inflammatory marker and neonatal DBS collected with restricted temporal proximity.

Each point corresponds to one neonatal inflammatory marker. Markers are colored by statistical significance and direction of differential expression.

# **Supplementary Table 10.** Regression estimates for associations between continuous maternal inflammation levels measured among the restricted temporal proximity subset (n = 105), ordered by adjusted p-value

| **Neonatal  inflammatory marker** | **Maternal Inflammatory Marker** | **Beta** | **95% CI Low** | **95% CI High** | **p-value** | **Adjusted p-value** |
| --- | --- | --- | --- | --- | --- | --- |
| IL.18R1 | IL1B | 0.219 | 0.056 | 0.382 | 0.009 | 0.309 |
| FGF.21 | IL1B | 0.312 | 0.080 | 0.545 | 0.009 | 0.309 |
| CX3CL1 | IL1B | 0.129 | 0.030 | 0.228 | 0.011 | 0.309 |
| OSM | IL1B | 0.168 | 0.036 | 0.301 | 0.013 | 0.309 |
| SCF | IL1B | 0.124 | 0.014 | 0.235 | 0.028 | 0.462 |
| CXCL1 | IL1B | 0.115 | 0.008 | 0.222 | 0.035 | 0.462 |
| CCL23 | IL1B | 0.149 | 0.007 | 0.292 | 0.041 | 0.462 |
| CASP.8 | IL1B | 0.083 | -0.001 | 0.168 | 0.053 | 0.462 |
| IL.17A | IL1B | 0.064 | -0.002 | 0.130 | 0.057 | 0.462 |
| MCP.3 | IL1B | 0.173 | -0.007 | 0.353 | 0.059 | 0.462 |
| ST1A1 | IL1B | -0.100 | -0.205 | 0.005 | 0.063 | 0.462 |
| GDNF | IL1B | -0.077 | -0.158 | 0.005 | 0.064 | 0.462 |
| IL8 | IL1B | 0.145 | -0.012 | 0.303 | 0.070 | 0.462 |
| IL6 | IL1B | 0.163 | -0.019 | 0.344 | 0.078 | 0.462 |
| MCP.1 | IL1B | 0.158 | -0.021 | 0.337 | 0.082 | 0.462 |
| TGF.alpha | IL1B | 0.091 | -0.014 | 0.196 | 0.090 | 0.462 |
| LIF.R | IL1B | 0.031 | -0.006 | 0.068 | 0.100 | 0.462 |
| MMP.10 | IL1B | 0.107 | -0.022 | 0.237 | 0.103 | 0.462 |
| TRANCE | IL1B | -0.099 | -0.219 | 0.021 | 0.106 | 0.462 |
| CSF.1 | IL1B | 0.069 | -0.016 | 0.154 | 0.110 | 0.462 |
| IL18 | IL1B | 0.100 | -0.025 | 0.225 | 0.115 | 0.462 |
| HGF | IL1B | 0.110 | -0.027 | 0.248 | 0.115 | 0.462 |
| CXCL6 | IL1B | 0.113 | -0.028 | 0.254 | 0.115 | 0.462 |
| TRAIL | IL1B | 0.085 | -0.023 | 0.193 | 0.123 | 0.472 |
| SLAMF1 | IL1B | 0.053 | -0.016 | 0.122 | 0.131 | 0.483 |
| IL.10RB | IL1B | 0.064 | -0.024 | 0.151 | 0.152 | 0.509 |
| PD.L1 | IL1B | 0.073 | -0.028 | 0.175 | 0.156 | 0.509 |
| AXIN1 | IL1B | 0.083 | -0.033 | 0.199 | 0.158 | 0.509 |
| CD40 | IL1B | 0.096 | -0.039 | 0.230 | 0.160 | 0.509 |
| uPA | IL1B | 0.084 | -0.037 | 0.206 | 0.171 | 0.525 |
| Flt3L | IL17A | -0.123 | -0.209 | -0.036 | 0.006 | 0.532 |
| IFN.gamma | IL1B | 0.123 | -0.060 | 0.306 | 0.185 | 0.548 |
| CXCL9 | IL1B | -0.069 | -0.176 | 0.037 | 0.201 | 0.576 |
| NRTN | IL1B | -0.030 | -0.077 | 0.017 | 0.207 | 0.576 |
| CD6 | IL1B | 0.095 | -0.067 | 0.258 | 0.248 | 0.670 |
| CD5 | IL1B | 0.100 | -0.077 | 0.276 | 0.267 | 0.700 |
| OPG | IL1B | 0.053 | -0.042 | 0.148 | 0.275 | 0.700 |
| IL5 | IL1B | -0.040 | -0.114 | 0.034 | 0.282 | 0.700 |
| TNFB | IL1B | -0.039 | -0.111 | 0.033 | 0.291 | 0.704 |
| CCL25 | IL1B | 0.054 | -0.050 | 0.159 | 0.304 | 0.716 |
| LIF | IL1B | 0.030 | -0.030 | 0.090 | 0.318 | 0.732 |
| CCL4 | IL1B | 0.067 | -0.071 | 0.204 | 0.339 | 0.759 |
| CCL11 | IL1B | 0.076 | -0.083 | 0.235 | 0.347 | 0.759 |
| IL10 | IL1B | 0.036 | -0.042 | 0.114 | 0.362 | 0.773 |
| IL7 | IL1B | 0.032 | -0.038 | 0.101 | 0.370 | 0.773 |
| CXCL10 | IL1B | 0.061 | -0.079 | 0.201 | 0.387 | 0.791 |
| CXCL11 | IL17A | -0.223 | -0.412 | -0.034 | 0.021 | 0.816 |
| EN.RAGE | IL17A | 0.100 | 0.000034 | 0.201 | 0.050 | 0.816 |
| IL4 | IL17A | -0.075 | -0.150 | 0.000 | 0.051 | 0.816 |
| NRTN | IL17A | 0.040 | 0.000 | 0.081 | 0.053 | 0.816 |
| IL.17C | IL17A | -0.133 | -0.277 | 0.011 | 0.070 | 0.816 |
| CDCP1 | IL17A | -0.053 | -0.111 | 0.006 | 0.077 | 0.816 |
| IL10 | IL17A | 0.057 | -0.011 | 0.125 | 0.100 | 0.816 |
| CD8A | IL17A | -0.099 | -0.226 | 0.027 | 0.122 | 0.816 |
| FGF.19 | IL17A | -0.109 | -0.249 | 0.031 | 0.127 | 0.816 |
| IL.20 | IL17A | -0.034 | -0.081 | 0.013 | 0.153 | 0.816 |
| IL.12B | IL17A | -0.070 | -0.173 | 0.033 | 0.178 | 0.816 |
| uPA | IL17A | 0.073 | -0.034 | 0.180 | 0.180 | 0.816 |
| LIF.R | IL17A | -0.022 | -0.055 | 0.011 | 0.182 | 0.816 |
| TRANCE | IL17A | -0.071 | -0.177 | 0.035 | 0.188 | 0.816 |
| CCL25 | IL17A | -0.061 | -0.152 | 0.031 | 0.189 | 0.816 |
| TGF.alpha | IL17A | 0.062 | -0.031 | 0.155 | 0.191 | 0.816 |
| TNFB | IL17A | -0.040 | -0.104 | 0.023 | 0.209 | 0.816 |
| TNFSF14 | IL17A | 0.077 | -0.044 | 0.198 | 0.210 | 0.816 |
| CCL19 | IL17A | -0.057 | -0.147 | 0.033 | 0.215 | 0.816 |
| IL.22.RA1 | IL17A | 0.068 | -0.045 | 0.181 | 0.237 | 0.816 |
| CXCL10 | IL17A | -0.073 | -0.196 | 0.050 | 0.243 | 0.816 |
| ST1A1 | IL17A | 0.054 | -0.039 | 0.148 | 0.254 | 0.816 |
| MCP.3 | IL17A | -0.089 | -0.249 | 0.071 | 0.274 | 0.816 |
| MCP.1 | IL17A | -0.086 | -0.245 | 0.073 | 0.283 | 0.816 |
| CD5 | IL17A | -0.084 | -0.240 | 0.071 | 0.285 | 0.816 |
| CD40 | IL17A | -0.062 | -0.181 | 0.056 | 0.300 | 0.816 |
| AXIN1 | IL17A | -0.053 | -0.156 | 0.049 | 0.304 | 0.816 |
| IL.17A | IL17A | 0.030 | -0.029 | 0.089 | 0.308 | 0.816 |
| IL13 | IL17A | -0.025 | -0.072 | 0.023 | 0.312 | 0.816 |
| MMP.1 | IL17A | -0.096 | -0.284 | 0.092 | 0.314 | 0.816 |
| CXCL5 | IL17A | -0.088 | -0.263 | 0.087 | 0.321 | 0.816 |
| MCP.4 | IL17A | -0.080 | -0.238 | 0.079 | 0.321 | 0.816 |
| CXCL1 | IL17A | 0.048 | -0.048 | 0.144 | 0.328 | 0.816 |
| Beta.NGF | IL17A | 0.011 | -0.012 | 0.034 | 0.340 | 0.816 |
| CXCL6 | IL17A | 0.060 | -0.065 | 0.185 | 0.346 | 0.816 |
| IL.20RA | IL17A | 0.025 | -0.028 | 0.077 | 0.352 | 0.816 |
| IL.1.alpha | IL17A | -0.142 | -0.444 | 0.160 | 0.353 | 0.816 |
| TNFRSF9 | IL17A | -0.051 | -0.160 | 0.058 | 0.355 | 0.816 |
| CD6 | IL17A | -0.065 | -0.208 | 0.079 | 0.374 | 0.816 |
| DNER | IL17A | -0.033 | -0.108 | 0.041 | 0.378 | 0.816 |
| FGF.23 | IL17A | -0.051 | -0.167 | 0.064 | 0.380 | 0.816 |
| CXCL9 | IL17A | -0.042 | -0.136 | 0.053 | 0.383 | 0.816 |
| SLAMF1 | IL17A | 0.025 | -0.036 | 0.087 | 0.413 | 0.816 |
| OPG | IL17A | -0.034 | -0.118 | 0.050 | 0.426 | 0.816 |
| CCL20 | IL17A | -0.055 | -0.195 | 0.085 | 0.436 | 0.816 |
| ARTN | IL17A | -0.026 | -0.095 | 0.042 | 0.445 | 0.816 |
| CASP.8 | IL17A | 0.029 | -0.047 | 0.104 | 0.451 | 0.816 |
| CCL28 | IL17A | 0.014 | -0.023 | 0.052 | 0.453 | 0.816 |
| IL.18R1 | IL17A | 0.056 | -0.092 | 0.203 | 0.458 | 0.816 |
| IL.10RA | IL17A | -0.015 | -0.057 | 0.026 | 0.460 | 0.816 |
| TNF | IL17A | -0.047 | -0.175 | 0.080 | 0.463 | 0.816 |
| LIF | IL17A | -0.019 | -0.072 | 0.034 | 0.481 | 0.816 |
| IL.10RB | IL17A | -0.027 | -0.105 | 0.051 | 0.495 | 0.816 |
| CST5 | IL17A | -0.045 | -0.177 | 0.086 | 0.496 | 0.816 |
| STAMBP | IL17A | 0.010 | -0.019 | 0.039 | 0.497 | 0.816 |
| PD.L1 | IL17A | -0.029 | -0.119 | 0.062 | 0.532 | 0.816 |
| SIRT2 | IL17A | 0.014 | -0.032 | 0.060 | 0.551 | 0.816 |
| IL8 | IL17A | -0.042 | -0.183 | 0.099 | 0.556 | 0.816 |
| IL33 | IL17A | -0.017 | -0.076 | 0.041 | 0.560 | 0.816 |
| FGF.5 | IL17A | -0.023 | -0.102 | 0.056 | 0.569 | 0.816 |
| IL.15RA | IL17A | 0.019 | -0.046 | 0.084 | 0.569 | 0.816 |
| IL6 | IL17A | -0.046 | -0.207 | 0.116 | 0.577 | 0.816 |
| IL.2RB | IL17A | -0.014 | -0.062 | 0.035 | 0.583 | 0.816 |
| HGF | IL17A | 0.031 | -0.091 | 0.154 | 0.613 | 0.816 |
| CD244 | IL17A | -0.027 | -0.133 | 0.079 | 0.614 | 0.816 |
| CSF.1 | IL17A | -0.019 | -0.095 | 0.056 | 0.616 | 0.816 |
| GDNF | IL17A | -0.018 | -0.091 | 0.054 | 0.617 | 0.816 |
| IL18 | IL17A | 0.028 | -0.083 | 0.139 | 0.623 | 0.816 |
| IL5 | IL17A | 0.016 | -0.049 | 0.081 | 0.628 | 0.816 |
| IL7 | IL17A | -0.015 | -0.076 | 0.046 | 0.630 | 0.816 |
| IL.24 | IL1B | -0.053 | -0.179 | 0.073 | 0.409 | 0.819 |
| CD8A | IL1B | 0.059 | -0.086 | 0.204 | 0.421 | 0.825 |
| IL.17C | IL1B | 0.066 | -0.100 | 0.231 | 0.433 | 0.827 |
| NT.3 | IL1B | 0.028 | -0.044 | 0.101 | 0.441 | 0.827 |
| TWEAK | IL17A | 0.017 | -0.058 | 0.092 | 0.654 | 0.836 |
| NT.3 | IL17A | 0.013 | -0.051 | 0.077 | 0.686 | 0.838 |
| TSLP | IL17A | 0.013 | -0.052 | 0.079 | 0.690 | 0.838 |
| MCP.2 | IL17A | -0.028 | -0.170 | 0.114 | 0.692 | 0.838 |
| ADA | IL17A | 0.011 | -0.046 | 0.069 | 0.693 | 0.838 |
| IL.24 | IL17A | -0.021 | -0.132 | 0.090 | 0.710 | 0.838 |
| TRAIL | IL17A | 0.018 | -0.078 | 0.114 | 0.711 | 0.838 |
| SCF | IL17A | -0.018 | -0.118 | 0.081 | 0.719 | 0.838 |
| OSM | IL17A | -0.020 | -0.140 | 0.101 | 0.746 | 0.849 |
| VEGFA | IL17A | 0.017 | -0.095 | 0.130 | 0.761 | 0.849 |
| CCL23 | IL17A | 0.020 | -0.109 | 0.148 | 0.763 | 0.849 |
| IFN.gamma | IL17A | -0.024 | -0.187 | 0.138 | 0.766 | 0.849 |
| CCL3 | IL1B | 0.078 | -0.134 | 0.290 | 0.466 | 0.855 |
| FGF.19 | IL1B | 0.058 | -0.103 | 0.218 | 0.478 | 0.855 |
| IL.15RA | IL1B | 0.026 | -0.048 | 0.100 | 0.488 | 0.855 |
| CCL20 | IL1B | 0.054 | -0.105 | 0.213 | 0.504 | 0.855 |
| IL13 | IL1B | 0.018 | -0.036 | 0.073 | 0.510 | 0.855 |
| ADA | IL1B | 0.022 | -0.044 | 0.087 | 0.511 | 0.855 |
| TWEAK | IL1B | 0.026 | -0.058 | 0.111 | 0.538 | 0.882 |
| IL.12B | IL1B | -0.036 | -0.154 | 0.082 | 0.547 | 0.882 |
| CDCP1 | IL1B | -0.020 | -0.087 | 0.047 | 0.558 | 0.882 |
| IL.22.RA1 | IL1B | -0.036 | -0.166 | 0.093 | 0.578 | 0.882 |
| TSLP | IL1B | 0.019 | -0.055 | 0.093 | 0.612 | 0.882 |
| DNER | IL1B | 0.022 | -0.063 | 0.107 | 0.612 | 0.882 |
| CD244 | IL1B | 0.028 | -0.093 | 0.148 | 0.650 | 0.882 |
| Beta.NGF | IL1B | 0.006 | -0.020 | 0.032 | 0.655 | 0.882 |
| CCL19 | IL1B | 0.023 | -0.080 | 0.125 | 0.665 | 0.882 |
| MCP.4 | IL1B | 0.038 | -0.142 | 0.219 | 0.675 | 0.882 |
| ARTN | IL1B | -0.016 | -0.094 | 0.061 | 0.677 | 0.882 |
| VEGFA | IL1B | 0.027 | -0.101 | 0.154 | 0.679 | 0.882 |
| EN.RAGE | IL1B | -0.024 | -0.140 | 0.092 | 0.680 | 0.882 |
| IL.10RA | IL1B | 0.009 | -0.038 | 0.056 | 0.695 | 0.882 |
| MMP.1 | IL1B | 0.041 | -0.173 | 0.255 | 0.706 | 0.882 |
| TNFSF14 | IL1B | 0.026 | -0.113 | 0.164 | 0.712 | 0.882 |
| IL.1.alpha | IL1B | -0.064 | -0.408 | 0.280 | 0.714 | 0.882 |
| CST5 | IL1B | -0.027 | -0.177 | 0.123 | 0.720 | 0.882 |
| STAMBP | IL1B | 0.006 | -0.027 | 0.039 | 0.720 | 0.882 |
| IL33 | IL1B | 0.012 | -0.055 | 0.078 | 0.730 | 0.882 |
| IL.20RA | IL1B | -0.010 | -0.070 | 0.049 | 0.730 | 0.882 |
| TNFRSF9 | IL1B | 0.021 | -0.103 | 0.145 | 0.738 | 0.882 |
| CCL28 | IL1B | -0.007 | -0.050 | 0.036 | 0.750 | 0.885 |
| IL4 | IL1B | -0.011 | -0.098 | 0.076 | 0.795 | 0.908 |
| IL.2RB | IL1B | -0.007 | -0.062 | 0.048 | 0.805 | 0.908 |
| Flt3L | IL1B | 0.012 | -0.089 | 0.114 | 0.810 | 0.908 |
| LAP.TGF.beta.1 | IL1B | 0.012 | -0.090 | 0.114 | 0.812 | 0.908 |
| MCP.2 | IL1B | -0.017 | -0.178 | 0.144 | 0.833 | 0.908 |
| CXCL11 | IL1B | -0.022 | -0.242 | 0.198 | 0.843 | 0.908 |
| IL.20 | IL1B | -0.005 | -0.059 | 0.049 | 0.844 | 0.908 |
| SIRT2 | IL1B | -0.005 | -0.057 | 0.047 | 0.849 | 0.908 |
| CST5 | CRP | -0.158 | -0.279 | -0.037 | 0.011 | 0.916 |
| SLAMF1 | CRP | 0.065 | 0.008 | 0.121 | 0.026 | 0.916 |
| CCL20 | CRP | 0.132 | 0.002 | 0.262 | 0.047 | 0.916 |
| Flt3L | CRP | -0.084 | -0.167 | -0.001 | 0.049 | 0.916 |
| IL10 | CRP | 0.064 | -0.001 | 0.128 | 0.052 | 0.916 |
| MMP.10 | CRP | 0.097 | -0.011 | 0.205 | 0.077 | 0.916 |
| IL4 | CRP | -0.063 | -0.135 | 0.008 | 0.083 | 0.916 |
| CCL11 | CRP | -0.114 | -0.245 | 0.018 | 0.090 | 0.916 |
| X4E.BP1 | CRP | 0.029 | -0.008 | 0.065 | 0.121 | 0.916 |
| CD8A | CRP | -0.093 | -0.213 | 0.027 | 0.126 | 0.916 |
| CX3CL1 | CRP | -0.062 | -0.147 | 0.022 | 0.145 | 0.916 |
| CXCL5 | CRP | 0.120 | -0.045 | 0.284 | 0.153 | 0.916 |
| IL8 | CRP | 0.094 | -0.038 | 0.226 | 0.162 | 0.916 |
| CD5 | CRP | -0.103 | -0.250 | 0.044 | 0.168 | 0.916 |
| HGF | CRP | -0.079 | -0.194 | 0.037 | 0.179 | 0.916 |
| CD6 | CRP | -0.092 | -0.227 | 0.044 | 0.182 | 0.916 |
| AXIN1 | CRP | -0.062 | -0.159 | 0.035 | 0.206 | 0.916 |
| TSLP | CRP | -0.038 | -0.100 | 0.024 | 0.226 | 0.916 |
| FGF.23 | CRP | 0.065 | -0.044 | 0.175 | 0.239 | 0.916 |
| IL33 | CRP | 0.033 | -0.023 | 0.088 | 0.247 | 0.916 |
| FGF.21 | CRP | 0.116 | -0.084 | 0.315 | 0.254 | 0.916 |
| TNFSF14 | CRP | -0.065 | -0.180 | 0.050 | 0.262 | 0.916 |
| TGF.alpha | CRP | 0.050 | -0.039 | 0.138 | 0.266 | 0.916 |
| uPA | CRP | -0.056 | -0.158 | 0.046 | 0.277 | 0.916 |
| TWEAK | CRP | -0.036 | -0.106 | 0.035 | 0.317 | 0.916 |
| NT.3 | CRP | 0.029 | -0.032 | 0.089 | 0.353 | 0.916 |
| TRANCE | CRP | -0.045 | -0.146 | 0.056 | 0.375 | 0.916 |
| EN.RAGE | CRP | 0.043 | -0.053 | 0.140 | 0.376 | 0.916 |
| IL.18R1 | CRP | -0.060 | -0.200 | 0.080 | 0.398 | 0.916 |
| STAMBP | CRP | -0.012 | -0.039 | 0.016 | 0.399 | 0.916 |
| IL18 | CRP | -0.044 | -0.149 | 0.061 | 0.411 | 0.916 |
| ST1A1 | CRP | -0.037 | -0.126 | 0.052 | 0.414 | 0.916 |
| OPG | CRP | 0.031 | -0.048 | 0.111 | 0.440 | 0.916 |
| VEGFA | CRP | -0.041 | -0.147 | 0.065 | 0.448 | 0.916 |
| FGF.19 | CRP | -0.051 | -0.185 | 0.083 | 0.452 | 0.916 |
| CDCP1 | CRP | -0.021 | -0.077 | 0.035 | 0.455 | 0.916 |
| CCL19 | CRP | 0.032 | -0.053 | 0.118 | 0.456 | 0.916 |
| ADA | CRP | -0.019 | -0.074 | 0.035 | 0.487 | 0.916 |
| CCL25 | CRP | 0.031 | -0.057 | 0.118 | 0.488 | 0.916 |
| ARTN | CRP | 0.023 | -0.042 | 0.087 | 0.488 | 0.916 |
| CXCL11 | CRP | -0.064 | -0.247 | 0.120 | 0.492 | 0.916 |
| MCP.4 | CRP | -0.052 | -0.202 | 0.099 | 0.498 | 0.916 |
| TRAIL | CRP | -0.031 | -0.122 | 0.060 | 0.499 | 0.916 |
| TNFRSF9 | CRP | -0.035 | -0.139 | 0.068 | 0.500 | 0.916 |
| MCP.3 | CRP | 0.052 | -0.101 | 0.204 | 0.502 | 0.916 |
| LIF | CRP | 0.017 | -0.033 | 0.067 | 0.507 | 0.916 |
| IL.17A | CRP | -0.019 | -0.075 | 0.037 | 0.512 | 0.916 |
| SIRT2 | CRP | -0.014 | -0.058 | 0.029 | 0.518 | 0.916 |
| MCP.1 | CRP | -0.046 | -0.197 | 0.105 | 0.548 | 0.916 |
| CD40 | CRP | -0.034 | -0.147 | 0.079 | 0.551 | 0.916 |
| IL5 | CRP | 0.018 | -0.043 | 0.080 | 0.556 | 0.916 |
| GDNF | CRP | 0.020 | -0.048 | 0.089 | 0.558 | 0.916 |
| IL.20RA | CRP | 0.013 | -0.036 | 0.063 | 0.601 | 0.916 |
| MMP.1 | CRP | -0.047 | -0.226 | 0.132 | 0.605 | 0.916 |
| MCP.2 | CRP | 0.034 | -0.100 | 0.169 | 0.612 | 0.916 |
| CCL23 | CRP | -0.030 | -0.152 | 0.091 | 0.620 | 0.916 |
| DNER | CRP | 0.018 | -0.053 | 0.089 | 0.621 | 0.916 |
| OSM | CRP | -0.028 | -0.142 | 0.086 | 0.622 | 0.916 |
| IL.17C | CRP | 0.034 | -0.105 | 0.172 | 0.632 | 0.916 |
| SCF | CRP | -0.023 | -0.117 | 0.072 | 0.634 | 0.916 |
| LIF.R | CRP | -0.007 | -0.039 | 0.024 | 0.649 | 0.916 |
| IL2 | CRP | -0.012 | -0.066 | 0.042 | 0.651 | 0.916 |
| CD244 | CRP | -0.023 | -0.123 | 0.078 | 0.654 | 0.916 |
| IL13 | CRP | -0.010 | -0.056 | 0.035 | 0.656 | 0.916 |
| CCL28 | CRP | -0.008 | -0.044 | 0.028 | 0.658 | 0.916 |
| IFN.gamma | CRP | -0.034 | -0.187 | 0.120 | 0.665 | 0.916 |
| Beta.NGF | CRP | 0.005 | -0.017 | 0.027 | 0.667 | 0.916 |
| CCL4 | IL17A | -0.012 | -0.134 | 0.110 | 0.846 | 0.920 |
| FGF.21 | IL17A | 0.020 | -0.192 | 0.232 | 0.850 | 0.920 |
| IL.10RB | CRP | 0.015 | -0.059 | 0.089 | 0.690 | 0.933 |
| PD.L1 | CRP | -0.016 | -0.102 | 0.069 | 0.706 | 0.935 |
| TNFB | CRP | 0.011 | -0.050 | 0.071 | 0.722 | 0.935 |
| IL.22.RA1 | CRP | -0.019 | -0.128 | 0.089 | 0.722 | 0.935 |
| IL2 | IL1B | 0.005 | -0.060 | 0.069 | 0.888 | 0.940 |
| IL.15RA | CRP | -0.010 | -0.072 | 0.051 | 0.737 | 0.942 |
| FGF.23 | IL1B | 0.007 | -0.125 | 0.139 | 0.917 | 0.944 |
| X4E.BP1 | IL1B | -0.002 | -0.046 | 0.042 | 0.928 | 0.944 |
| FGF.5 | IL1B | -0.004 | -0.094 | 0.086 | 0.930 | 0.944 |
| CXCL5 | IL1B | 0.008 | -0.191 | 0.208 | 0.934 | 0.944 |
| IL.2RB | CRP | -0.007 | -0.053 | 0.039 | 0.761 | 0.945 |
| CXCL10 | CRP | 0.016 | -0.101 | 0.134 | 0.783 | 0.945 |
| IL.24 | CRP | 0.014 | -0.091 | 0.120 | 0.786 | 0.945 |
| IL7 | CRP | -0.008 | -0.066 | 0.051 | 0.797 | 0.945 |
| NRTN | CRP | 0.005 | -0.034 | 0.044 | 0.798 | 0.945 |
| IL6 | CRP | -0.020 | -0.173 | 0.134 | 0.801 | 0.945 |
| IL2 | IL17A | 0.004 | -0.053 | 0.061 | 0.888 | 0.948 |
| X4E.BP1 | IL17A | 0.003 | -0.036 | 0.042 | 0.897 | 0.948 |
| CASP.8 | CRP | -0.007 | -0.079 | 0.064 | 0.838 | 0.951 |
| IL.10RA | CRP | 0.004 | -0.035 | 0.043 | 0.846 | 0.951 |
| TNF | CRP | -0.012 | -0.133 | 0.110 | 0.850 | 0.951 |
| LAP.TGF.beta.1 | CRP | -0.008 | -0.093 | 0.077 | 0.857 | 0.951 |
| CSF.1 | CRP | -0.006 | -0.078 | 0.066 | 0.867 | 0.951 |
| IL.20 | CRP | -0.003 | -0.048 | 0.042 | 0.889 | 0.951 |
| CCL3 | CRP | 0.012 | -0.165 | 0.190 | 0.892 | 0.951 |
| FGF.5 | CRP | 0.005 | -0.070 | 0.080 | 0.898 | 0.951 |
| CCL4 | CRP | 0.007 | -0.108 | 0.123 | 0.899 | 0.951 |
| CX3CL1 | IL17A | 0.005 | -0.085 | 0.095 | 0.912 | 0.954 |
| CXCL1 | CRP | -0.004 | -0.096 | 0.087 | 0.927 | 0.956 |
| CXCL6 | CRP | -0.005 | -0.124 | 0.114 | 0.929 | 0.956 |
| IL.12B | CRP | -0.004 | -0.103 | 0.094 | 0.935 | 0.956 |
| CCL11 | IL17A | 0.006 | -0.135 | 0.147 | 0.934 | 0.965 |
| LAP.TGF.beta.1 | IL17A | -0.002 | -0.092 | 0.087 | 0.958 | 0.974 |
| CCL3 | IL17A | 0.004 | -0.183 | 0.192 | 0.964 | 0.974 |
| TNF | IL1B | 0.001 | -0.144 | 0.147 | 0.984 | 0.984 |
| IL.1.alpha | CRP | 0.004 | -0.284 | 0.291 | 0.979 | 0.990 |
| CXCL9 | CRP | 0.000 | -0.090 | 0.089 | 0.992 | 0.992 |
| MCP.3 | IL6 | 0.185 | 0.035 | 0.335 | 0.016 | 0.992 |
| DNER | IL6 | -0.074 | -0.144 | -0.003 | 0.041 | 0.992 |
| IL.17C | IL6 | 0.140 | 0.003 | 0.278 | 0.046 | 0.992 |
| IL.20RA | IL6 | 0.041 | -0.008 | 0.091 | 0.100 | 0.992 |
| SLAMF1 | IL6 | 0.045 | -0.013 | 0.104 | 0.126 | 0.992 |
| CCL11 | IL6 | 0.099 | -0.034 | 0.233 | 0.143 | 0.992 |
| IL8 | IL6 | 0.099 | -0.035 | 0.233 | 0.147 | 0.992 |
| IL7 | IL6 | -0.043 | -0.101 | 0.015 | 0.148 | 0.992 |
| MCP.1 | IL6 | 0.102 | -0.050 | 0.254 | 0.186 | 0.992 |
| IL13 | IL6 | -0.028 | -0.074 | 0.018 | 0.233 | 0.992 |
| TRANCE | IL6 | -0.061 | -0.163 | 0.041 | 0.240 | 0.992 |
| FGF.19 | IL6 | -0.080 | -0.216 | 0.055 | 0.241 | 0.992 |
| ST1A1 | IL6 | -0.053 | -0.142 | 0.037 | 0.248 | 0.992 |
| MMP.1 | IL6 | -0.101 | -0.281 | 0.079 | 0.269 | 0.992 |
| IL.10RA | IL6 | -0.022 | -0.062 | 0.018 | 0.275 | 0.992 |
| GDNF | IL6 | 0.038 | -0.031 | 0.108 | 0.279 | 0.992 |
| OSM | IL6 | 0.060 | -0.055 | 0.175 | 0.302 | 0.992 |
| IL.1.alpha | IL6 | 0.151 | -0.139 | 0.440 | 0.304 | 0.992 |
| CXCL5 | IL6 | -0.086 | -0.254 | 0.082 | 0.313 | 0.992 |
| CCL3 | IL6 | -0.088 | -0.267 | 0.091 | 0.330 | 0.992 |
| IL.17A | IL6 | 0.028 | -0.029 | 0.085 | 0.331 | 0.992 |
| ADA | IL6 | 0.027 | -0.029 | 0.082 | 0.342 | 0.992 |
| CCL20 | IL6 | 0.064 | -0.070 | 0.198 | 0.343 | 0.992 |
| TNFB | IL6 | -0.027 | -0.088 | 0.034 | 0.386 | 0.992 |
| ARTN | IL6 | -0.029 | -0.094 | 0.037 | 0.388 | 0.992 |
| CDCP1 | IL6 | -0.025 | -0.081 | 0.032 | 0.388 | 0.992 |
| TNF | IL6 | -0.053 | -0.175 | 0.069 | 0.393 | 0.992 |
| TWEAK | IL6 | -0.030 | -0.101 | 0.042 | 0.413 | 0.992 |
| FGF.5 | IL6 | -0.031 | -0.107 | 0.045 | 0.417 | 0.992 |
| MCP.4 | IL6 | 0.062 | -0.090 | 0.214 | 0.422 | 0.992 |
| CSF.1 | IL6 | 0.029 | -0.044 | 0.101 | 0.434 | 0.992 |
| VEGFA | IL6 | -0.041 | -0.149 | 0.066 | 0.449 | 0.992 |
| IL18 | IL6 | 0.041 | -0.066 | 0.147 | 0.452 | 0.992 |
| OPG | IL6 | -0.029 | -0.110 | 0.051 | 0.470 | 0.992 |
| X4E.BP1 | IL6 | -0.014 | -0.051 | 0.024 | 0.471 | 0.992 |
| IL.20 | IL6 | -0.017 | -0.062 | 0.029 | 0.472 | 0.992 |
| CXCL11 | IL6 | 0.065 | -0.121 | 0.251 | 0.488 | 0.992 |
| MCP.2 | IL6 | -0.047 | -0.183 | 0.089 | 0.492 | 0.992 |
| IL4 | IL6 | 0.023 | -0.051 | 0.096 | 0.544 | 0.992 |
| NRTN | IL6 | 0.011 | -0.029 | 0.051 | 0.581 | 0.992 |
| CCL19 | IL6 | 0.024 | -0.063 | 0.111 | 0.588 | 0.992 |
| STAMBP | IL6 | 0.008 | -0.020 | 0.036 | 0.590 | 0.992 |
| FGF.21 | IL6 | 0.054 | -0.149 | 0.257 | 0.600 | 0.992 |
| IL5 | IL6 | -0.016 | -0.079 | 0.046 | 0.605 | 0.992 |
| LIF | IL6 | 0.013 | -0.038 | 0.064 | 0.615 | 0.992 |
| IL33 | IL6 | 0.014 | -0.043 | 0.070 | 0.632 | 0.992 |
| IL.22.RA1 | IL6 | -0.026 | -0.136 | 0.083 | 0.632 | 0.992 |
| IFN.gamma | IL6 | 0.038 | -0.118 | 0.193 | 0.633 | 0.992 |
| AXIN1 | IL6 | 0.024 | -0.075 | 0.122 | 0.637 | 0.992 |
| IL10 | IL6 | -0.015 | -0.082 | 0.051 | 0.648 | 0.992 |
| IL2 | IL6 | 0.012 | -0.042 | 0.067 | 0.660 | 0.992 |
| TNFSF14 | IL6 | -0.026 | -0.143 | 0.091 | 0.661 | 0.992 |
| CD5 | IL6 | 0.031 | -0.119 | 0.182 | 0.681 | 0.992 |
| TSLP | IL6 | -0.013 | -0.076 | 0.050 | 0.683 | 0.992 |
| CCL23 | IL6 | 0.025 | -0.098 | 0.148 | 0.685 | 0.992 |
| NT.3 | IL6 | 0.012 | -0.049 | 0.074 | 0.691 | 0.992 |
| CCL25 | IL6 | -0.017 | -0.106 | 0.071 | 0.696 | 0.992 |
| EN.RAGE | IL6 | -0.019 | -0.117 | 0.079 | 0.701 | 0.992 |
| CX3CL1 | IL6 | 0.016 | -0.070 | 0.102 | 0.714 | 0.992 |
| IL.15RA | IL6 | -0.011 | -0.074 | 0.051 | 0.722 | 0.992 |
| IL6 | IL6 | 0.025 | -0.130 | 0.181 | 0.748 | 0.992 |
| TNFRSF9 | IL6 | -0.017 | -0.122 | 0.088 | 0.748 | 0.992 |
| MMP.10 | IL6 | -0.017 | -0.128 | 0.094 | 0.765 | 0.992 |
| LIF.R | IL6 | -0.005 | -0.037 | 0.027 | 0.772 | 0.992 |
| CD244 | IL6 | -0.014 | -0.116 | 0.087 | 0.779 | 0.992 |
| CXCL1 | IL6 | 0.013 | -0.080 | 0.105 | 0.788 | 0.992 |
| CD6 | IL6 | 0.018 | -0.120 | 0.157 | 0.793 | 0.992 |
| TRAIL | IL6 | 0.012 | -0.080 | 0.105 | 0.794 | 0.992 |
| Beta.NGF | IL6 | -0.003 | -0.025 | 0.019 | 0.801 | 0.992 |
| Flt3L | IL6 | 0.011 | -0.075 | 0.097 | 0.806 | 0.992 |
| HGF | IL6 | 0.015 | -0.103 | 0.132 | 0.807 | 0.992 |
| CASP.8 | IL6 | -0.009 | -0.081 | 0.064 | 0.813 | 0.992 |
| IL.10RB | IL6 | -0.009 | -0.084 | 0.066 | 0.815 | 0.992 |
| IL.18R1 | IL6 | 0.017 | -0.126 | 0.159 | 0.819 | 0.992 |
| CXCL10 | IL6 | 0.013 | -0.106 | 0.132 | 0.827 | 0.992 |
| CCL4 | IL6 | 0.012 | -0.105 | 0.129 | 0.836 | 0.992 |
| FGF.23 | IL6 | 0.011 | -0.100 | 0.123 | 0.843 | 0.992 |
| SIRT2 | IL6 | 0.004 | -0.040 | 0.048 | 0.874 | 0.992 |
| IL.12B | IL6 | 0.007 | -0.093 | 0.107 | 0.887 | 0.992 |
| SCF | IL6 | -0.006 | -0.101 | 0.090 | 0.904 | 0.992 |
| LAP.TGF.beta.1 | IL6 | -0.005 | -0.091 | 0.081 | 0.905 | 0.992 |
| uPA | IL6 | -0.006 | -0.110 | 0.097 | 0.906 | 0.992 |
| PD.L1 | IL6 | -0.005 | -0.092 | 0.082 | 0.915 | 0.992 |
| CD40 | IL6 | 0.006 | -0.109 | 0.121 | 0.920 | 0.992 |
| CXCL9 | IL6 | 0.004 | -0.087 | 0.095 | 0.936 | 0.992 |
| CST5 | IL6 | 0.004 | -0.123 | 0.131 | 0.948 | 0.992 |
| TGF.alpha | IL6 | 0.002 | -0.089 | 0.092 | 0.973 | 0.992 |
| CCL28 | IL6 | 0.001 | -0.036 | 0.037 | 0.973 | 0.992 |
| IL.24 | IL6 | 0.002 | -0.105 | 0.108 | 0.977 | 0.992 |
| CXCL6 | IL6 | 0.001 | -0.119 | 0.122 | 0.985 | 0.992 |
| IL.2RB | IL6 | 0.000 | -0.047 | 0.047 | 0.990 | 0.992 |
| CD8A | IL6 | 0.001 | -0.122 | 0.123 | 0.992 | 0.992 |
| MMP.10 | IL17A | 0.0000618 | -0.115 | 0.116 | 0.999 | 0.999 |


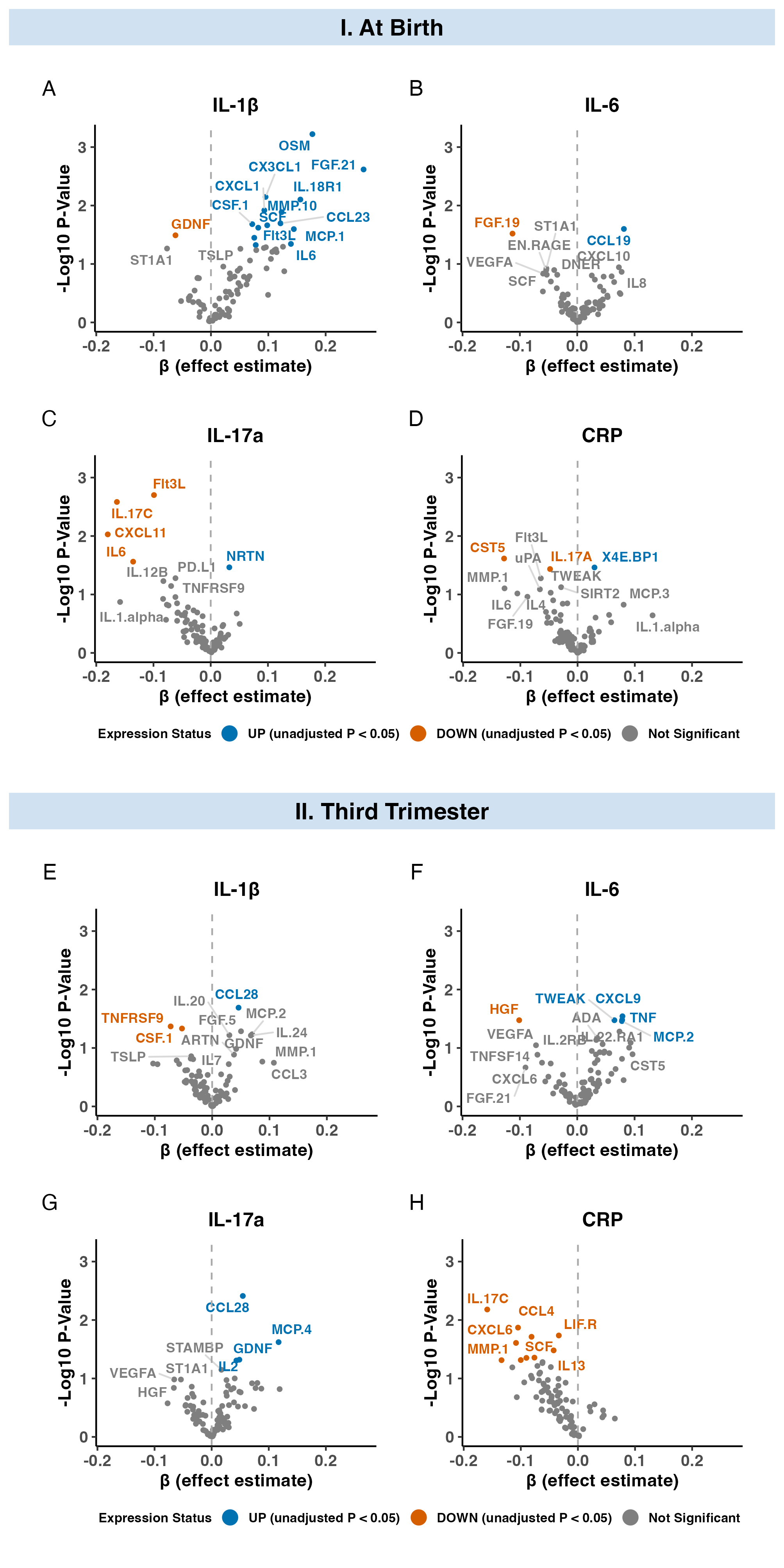


# **Supplementary Figure 3**. Volcano plots illustrating the associations between continuous maternal inflammation levels at birth (I) and during third trimester (II) and neonatal inflammatory marker expression for each maternal marker: IL-1β (A, E), IL-6 (B, F), IL-17a (C, G), and CRP (D, H), adjusting for maternal age, pre-pregnancy BMI, and race and ethnicity.

Panels A–D correspond to the primary analysis of maternal inflammation measured at birth (n = 194); panels E–H correspond to the secondary analysis of maternal inflammation measured in the third trimester (n = 235). Each point corresponds to one neonatal inflammatory marker. Neonatal inflammatory markers are colored by statistical significance and the direction of the estimated effect size.


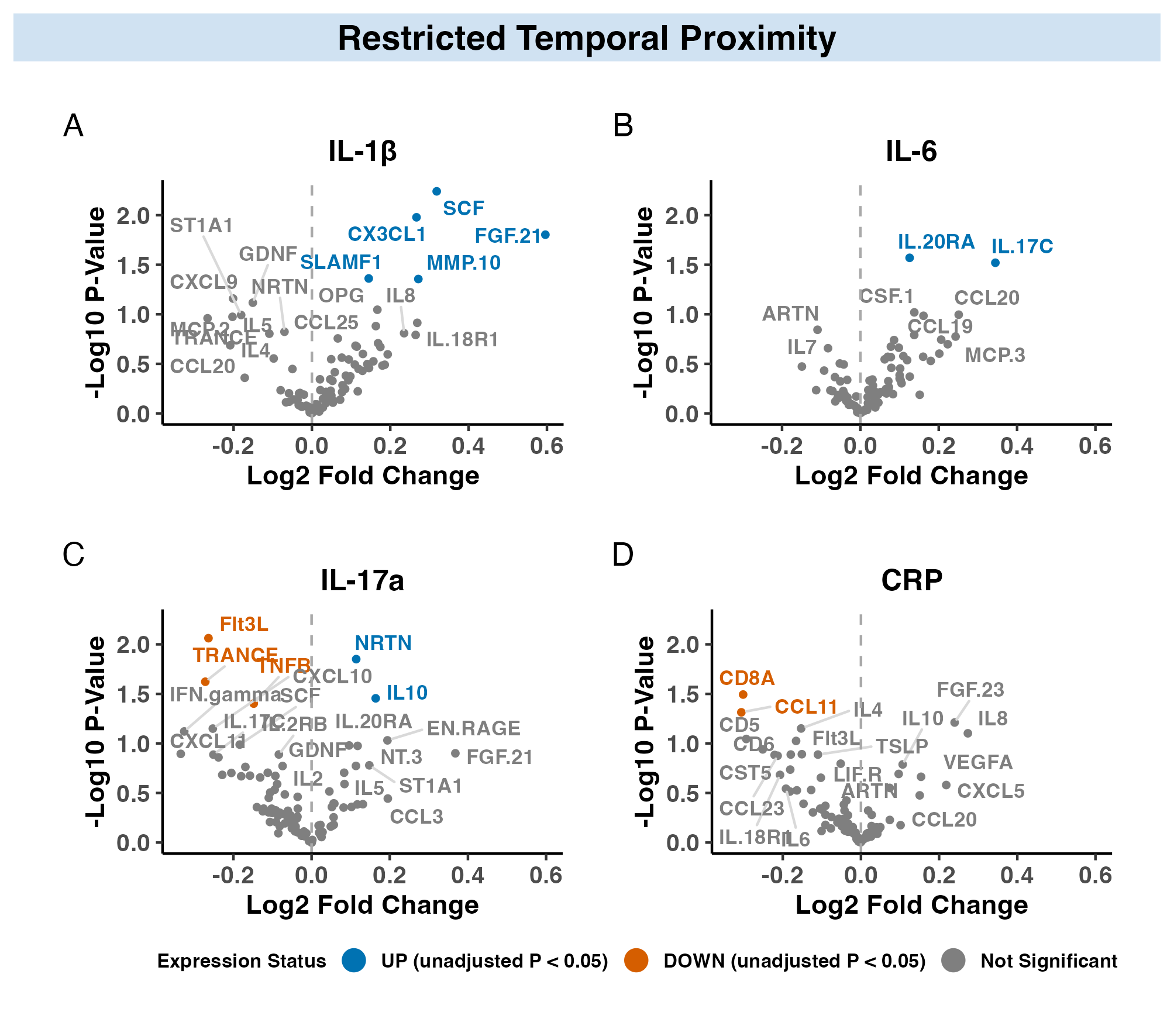


# **Supplementary Figure 4.** Volcano plots illustrating the associations between continuous maternal inflammation levels in the restricted temporal proximity sample (n=105) and neonatal inflammatory marker expression for each maternal marker: IL-1β (A), IL-6 (B), IL-17a (C), and CRP (D), adjusting for maternal age, pre-pregnancy BMI, and race and ethnicity.

Each point corresponds to one neonatal inflammatory marker. Neonatal inflammatory markers are colored by statistical significance and the direction of the estimated effect size.

# **Supplementary Table 11.** Regression estimates for associations between continuous maternal inflammation levels measured among the at-birth subset (n = 194), further adjusted for maternal age, pre-pregnancy BMI, and race and ethnicity, ordered by adjusted p-value

| **Neonatal  inflammatory marker** | **Maternal Inflammatory Marker** | **Beta** | **95% CI Low** | **95% CI High** | **p-value** | **Adjusted p-value** |
| --- | --- | --- | --- | --- | --- | --- |
| OSM | IL1B | 0.177 | 0.077 | 0.277 | 0.001 | 0.055 |
| FGF.21 | IL1B | 0.266 | 0.095 | 0.437 | 0.002 | 0.111 |
| Flt3L | IL17A | -0.099 | -0.162 | -0.037 | 0.002 | 0.120 |
| IL.17C | IL17A | -0.164 | -0.270 | -0.058 | 0.003 | 0.120 |
| CX3CL1 | IL1B | 0.095 | 0.026 | 0.164 | 0.007 | 0.182 |
| IL.18R1 | IL1B | 0.156 | 0.041 | 0.270 | 0.008 | 0.182 |
| CXCL1 | IL1B | 0.093 | 0.021 | 0.165 | 0.012 | 0.199 |
| MMP.10 | IL1B | 0.123 | 0.026 | 0.220 | 0.013 | 0.199 |
| CCL23 | IL1B | 0.121 | 0.019 | 0.223 | 0.020 | 0.212 |
| CSF.1 | IL1B | 0.072 | 0.011 | 0.133 | 0.021 | 0.212 |
| SCF | IL1B | 0.098 | 0.014 | 0.182 | 0.022 | 0.212 |
| Flt3L | IL1B | 0.082 | 0.011 | 0.154 | 0.024 | 0.212 |
| MCP.1 | IL1B | 0.145 | 0.018 | 0.271 | 0.025 | 0.212 |
| GDNF | IL1B | -0.062 | -0.119 | -0.005 | 0.032 | 0.231 |
| PD.L1 | IL1B | 0.076 | 0.005 | 0.146 | 0.036 | 0.231 |
| IL6 | IL1B | 0.139 | 0.003 | 0.276 | 0.045 | 0.231 |
| TRAIL | IL1B | 0.078 | 0.001 | 0.155 | 0.047 | 0.231 |
| CD5 | IL1B | 0.126 | 0.000 | 0.252 | 0.051 | 0.231 |
| HGF | IL1B | 0.096 | -0.001 | 0.192 | 0.052 | 0.231 |
| IL18 | IL1B | 0.092 | -0.001 | 0.186 | 0.053 | 0.231 |
| ST1A1 | IL1B | -0.077 | -0.155 | 0.001 | 0.054 | 0.231 |
| TSLP | IL1B | 0.051 | -0.001 | 0.104 | 0.055 | 0.231 |
| CD6 | IL1B | 0.113 | -0.003 | 0.229 | 0.056 | 0.231 |
| uPA | IL1B | 0.079 | -0.003 | 0.161 | 0.058 | 0.231 |
| CCL20 | IL1B | 0.111 | -0.004 | 0.225 | 0.058 | 0.231 |
| CCL11 | IL1B | 0.107 | -0.005 | 0.219 | 0.062 | 0.231 |
| MCP.3 | IL1B | 0.114 | -0.006 | 0.235 | 0.063 | 0.231 |
| CXCL11 | IL17A | -0.180 | -0.315 | -0.045 | 0.009 | 0.287 |
| IL8 | IL1B | 0.105 | -0.013 | 0.222 | 0.082 | 0.290 |
| AXIN1 | IL1B | 0.068 | -0.010 | 0.146 | 0.087 | 0.297 |
| LIF.R | IL1B | 0.021 | -0.005 | 0.048 | 0.111 | 0.364 |
| IL.17C | IL1B | 0.097 | -0.025 | 0.219 | 0.119 | 0.367 |
| IL.10RB | IL1B | 0.051 | -0.013 | 0.114 | 0.120 | 0.367 |
| IL4 | IL1B | 0.046 | -0.014 | 0.107 | 0.131 | 0.383 |
| IFN.gamma | IL1B | 0.128 | -0.039 | 0.295 | 0.133 | 0.383 |
| ADA | IL1B | 0.033 | -0.011 | 0.077 | 0.145 | 0.391 |
| CXCL6 | IL1B | 0.067 | -0.027 | 0.162 | 0.162 | 0.391 |
| CASP.8 | IL1B | 0.041 | -0.017 | 0.099 | 0.164 | 0.391 |
| TGF.alpha | IL1B | 0.057 | -0.024 | 0.139 | 0.165 | 0.391 |
| CCL28 | IL1B | -0.023 | -0.056 | 0.010 | 0.173 | 0.391 |
| IL.17A | IL1B | 0.035 | -0.015 | 0.085 | 0.174 | 0.391 |
| CD40 | IL1B | 0.064 | -0.029 | 0.157 | 0.174 | 0.391 |
| NRTN | IL1B | -0.024 | -0.058 | 0.011 | 0.175 | 0.391 |
| X4E.BP1 | IL1B | -0.021 | -0.051 | 0.009 | 0.175 | 0.391 |
| IL7 | IL1B | 0.033 | -0.015 | 0.081 | 0.179 | 0.391 |
| CCL4 | IL1B | 0.064 | -0.031 | 0.159 | 0.185 | 0.396 |
| CCL25 | IL1B | 0.048 | -0.028 | 0.123 | 0.212 | 0.443 |
| CD8A | IL1B | 0.062 | -0.038 | 0.162 | 0.224 | 0.459 |
| TNFRSF9 | IL1B | 0.051 | -0.034 | 0.137 | 0.239 | 0.478 |
| CCL19 | IL1B | 0.045 | -0.038 | 0.129 | 0.283 | 0.546 |
| IL5 | IL1B | -0.029 | -0.081 | 0.024 | 0.285 | 0.546 |
| IL.15RA | IL1B | 0.026 | -0.023 | 0.075 | 0.297 | 0.551 |
| DNER | IL1B | 0.032 | -0.028 | 0.092 | 0.300 | 0.551 |
| VEGFA | IL1B | 0.047 | -0.047 | 0.140 | 0.324 | 0.585 |
| OPG | IL1B | 0.035 | -0.037 | 0.107 | 0.334 | 0.587 |
| CCL3 | IL1B | 0.100 | -0.105 | 0.304 | 0.338 | 0.587 |
| CXCL9 | IL1B | -0.037 | -0.114 | 0.041 | 0.355 | 0.605 |
| IL10 | IL1B | 0.027 | -0.032 | 0.086 | 0.375 | 0.623 |
| FGF.23 | IL1B | -0.039 | -0.125 | 0.048 | 0.379 | 0.623 |
| IL.10RA | IL1B | 0.015 | -0.019 | 0.048 | 0.389 | 0.628 |
| IL6 | IL17A | -0.136 | -0.256 | -0.015 | 0.027 | 0.632 |
| NRTN | IL17A | 0.033 | 0.002 | 0.063 | 0.034 | 0.632 |
| IL.22.RA1 | IL1B | -0.038 | -0.131 | 0.055 | 0.416 | 0.659 |
| CXCL5 | IL1B | -0.052 | -0.183 | 0.078 | 0.431 | 0.665 |
| TNFSF14 | IL1B | 0.039 | -0.060 | 0.137 | 0.441 | 0.665 |
| CDCP1 | IL1B | -0.022 | -0.077 | 0.034 | 0.444 | 0.665 |
| EN.RAGE | IL1B | -0.031 | -0.112 | 0.050 | 0.449 | 0.665 |
| ARTN | IL1B | -0.021 | -0.077 | 0.035 | 0.456 | 0.665 |
| TNFB | IL1B | -0.018 | -0.068 | 0.033 | 0.495 | 0.711 |
| IL.20RA | IL1B | -0.014 | -0.055 | 0.027 | 0.512 | 0.715 |
| STAMBP | IL1B | 0.007 | -0.015 | 0.029 | 0.513 | 0.715 |
| PD.L1 | IL17A | -0.062 | -0.124 | 0.001 | 0.053 | 0.776 |
| IL.12B | IL17A | -0.083 | -0.169 | 0.003 | 0.059 | 0.776 |
| LIF | IL1B | -0.012 | -0.054 | 0.030 | 0.584 | 0.791 |
| CXCL10 | IL1B | 0.029 | -0.076 | 0.134 | 0.585 | 0.791 |
| SLAMF1 | IL1B | 0.015 | -0.041 | 0.070 | 0.607 | 0.810 |
| CST5 | IL1B | 0.032 | -0.093 | 0.156 | 0.617 | 0.811 |
| TNFRSF9 | IL17A | -0.069 | -0.145 | 0.006 | 0.072 | 0.824 |
| MCP.4 | IL1B | 0.032 | -0.103 | 0.166 | 0.644 | 0.835 |
| TRANCE | IL1B | -0.019 | -0.109 | 0.070 | 0.667 | 0.843 |
| CD244 | IL1B | 0.019 | -0.070 | 0.108 | 0.669 | 0.843 |
| FGF.23 | IL17A | -0.062 | -0.138 | 0.014 | 0.111 | 0.882 |
| FGF.19 | IL17A | -0.083 | -0.188 | 0.022 | 0.119 | 0.882 |
| IL.1.alpha | IL17A | -0.158 | -0.366 | 0.049 | 0.134 | 0.882 |
| TRANCE | IL17A | -0.059 | -0.137 | 0.020 | 0.142 | 0.882 |
| IL8 | IL17A | -0.077 | -0.181 | 0.028 | 0.150 | 0.882 |
| CCL20 | IL17A | -0.074 | -0.175 | 0.028 | 0.154 | 0.882 |
| IL.10RB | IL17A | -0.040 | -0.096 | 0.017 | 0.166 | 0.882 |
| TNFB | IL17A | -0.030 | -0.075 | 0.015 | 0.187 | 0.882 |
| CD8A | IL17A | -0.057 | -0.146 | 0.031 | 0.203 | 0.882 |
| IL.2RB | IL17A | -0.024 | -0.060 | 0.013 | 0.203 | 0.882 |
| AXIN1 | IL17A | -0.045 | -0.114 | 0.025 | 0.206 | 0.882 |
| OSM | IL17A | -0.058 | -0.149 | 0.033 | 0.208 | 0.882 |
| EN.RAGE | IL17A | 0.045 | -0.026 | 0.117 | 0.212 | 0.882 |
| VEGFA | IL17A | -0.051 | -0.133 | 0.032 | 0.225 | 0.882 |
| SCF | IL17A | -0.046 | -0.120 | 0.029 | 0.229 | 0.882 |
| LIF.R | IL17A | -0.014 | -0.038 | 0.009 | 0.235 | 0.882 |
| MMP.1 | IL17A | -0.078 | -0.218 | 0.061 | 0.271 | 0.882 |
| TNF | IL17A | -0.042 | -0.116 | 0.033 | 0.274 | 0.882 |
| MCP.3 | IL17A | -0.059 | -0.166 | 0.049 | 0.282 | 0.882 |
| DNER | IL17A | -0.028 | -0.081 | 0.025 | 0.299 | 0.882 |
| CCL4 | IL17A | -0.044 | -0.128 | 0.040 | 0.300 | 0.882 |
| CCL11 | IL17A | 0.051 | -0.049 | 0.151 | 0.319 | 0.882 |
| CXCL9 | IL17A | -0.034 | -0.103 | 0.034 | 0.324 | 0.882 |
| IL.17A | IL17A | 0.022 | -0.023 | 0.066 | 0.332 | 0.882 |
| IL.10RA | IL17A | -0.014 | -0.044 | 0.015 | 0.342 | 0.882 |
| IL.15RA | IL17A | 0.021 | -0.023 | 0.064 | 0.350 | 0.882 |
| CSF.1 | IL17A | -0.026 | -0.080 | 0.029 | 0.357 | 0.882 |
| CDCP1 | IL17A | -0.023 | -0.072 | 0.026 | 0.363 | 0.882 |
| Beta.NGF | IL17A | 0.007 | -0.008 | 0.022 | 0.381 | 0.882 |
| IL4 | IL17A | -0.023 | -0.077 | 0.031 | 0.396 | 0.882 |
| CD244 | IL17A | -0.034 | -0.112 | 0.045 | 0.400 | 0.882 |
| CD6 | IL17A | -0.043 | -0.147 | 0.061 | 0.414 | 0.882 |
| CST5 | IL17A | -0.045 | -0.155 | 0.065 | 0.423 | 0.882 |
| CXCL5 | IL17A | -0.046 | -0.161 | 0.069 | 0.430 | 0.882 |
| MCP.4 | IL17A | -0.048 | -0.166 | 0.071 | 0.430 | 0.882 |
| IL13 | IL17A | -0.015 | -0.051 | 0.022 | 0.434 | 0.882 |
| NT.3 | IL17A | 0.018 | -0.030 | 0.065 | 0.464 | 0.882 |
| TNFSF14 | IL17A | 0.031 | -0.057 | 0.118 | 0.490 | 0.882 |
| SLAMF1 | IL17A | 0.017 | -0.032 | 0.067 | 0.491 | 0.882 |
| MMP.10 | IL17A | -0.029 | -0.116 | 0.058 | 0.506 | 0.882 |
| CCL19 | IL17A | -0.024 | -0.098 | 0.049 | 0.513 | 0.882 |
| CD40 | IL17A | -0.027 | -0.109 | 0.055 | 0.517 | 0.882 |
| CASP.8 | IL17A | 0.016 | -0.035 | 0.068 | 0.533 | 0.882 |
| IFN.gamma | IL17A | -0.046 | -0.195 | 0.102 | 0.538 | 0.882 |
| IL18 | IL17A | 0.026 | -0.058 | 0.109 | 0.542 | 0.882 |
| FGF.5 | IL17A | -0.016 | -0.070 | 0.038 | 0.555 | 0.882 |
| LAP.TGF.beta.1 | IL17A | -0.018 | -0.079 | 0.043 | 0.556 | 0.882 |
| IL7 | IL17A | -0.012 | -0.055 | 0.030 | 0.568 | 0.882 |
| IL.22.RA1 | IL17A | 0.023 | -0.059 | 0.106 | 0.576 | 0.882 |
| TWEAK | IL17A | 0.015 | -0.039 | 0.069 | 0.577 | 0.882 |
| CXCL10 | IL17A | -0.025 | -0.117 | 0.068 | 0.602 | 0.882 |
| ADA | IL17A | 0.010 | -0.029 | 0.050 | 0.605 | 0.882 |
| IL.20 | IL17A | -0.008 | -0.043 | 0.026 | 0.629 | 0.882 |
| HGF | IL17A | -0.021 | -0.107 | 0.065 | 0.630 | 0.882 |
| GDNF | IL17A | 0.012 | -0.039 | 0.062 | 0.648 | 0.882 |
| CCL25 | IL17A | -0.015 | -0.082 | 0.052 | 0.654 | 0.882 |
| CCL3 | IL17A | -0.041 | -0.222 | 0.140 | 0.656 | 0.882 |
| CD5 | IL17A | -0.025 | -0.138 | 0.087 | 0.657 | 0.882 |
| STAMBP | IL17A | 0.004 | -0.015 | 0.024 | 0.660 | 0.882 |
| TSLP | IL17A | 0.010 | -0.037 | 0.057 | 0.672 | 0.882 |
| X4E.BP1 | IL17A | 0.006 | -0.021 | 0.033 | 0.672 | 0.882 |
| uPA | IL17A | 0.016 | -0.057 | 0.088 | 0.675 | 0.882 |
| IL2 | IL17A | 0.009 | -0.035 | 0.053 | 0.686 | 0.882 |
| ARTN | IL17A | -0.010 | -0.059 | 0.040 | 0.700 | 0.882 |
| TRAIL | IL17A | -0.013 | -0.082 | 0.055 | 0.700 | 0.882 |
| NT.3 | IL1B | 0.009 | -0.044 | 0.063 | 0.737 | 0.897 |
| IL.2RB | IL1B | 0.007 | -0.034 | 0.048 | 0.739 | 0.897 |
| CXCL11 | IL1B | 0.025 | -0.131 | 0.181 | 0.751 | 0.897 |
| MCP.2 | IL1B | 0.016 | -0.087 | 0.120 | 0.758 | 0.897 |
| FGF.5 | IL1B | 0.009 | -0.053 | 0.070 | 0.775 | 0.897 |
| IL.1.alpha | IL1B | 0.031 | -0.205 | 0.268 | 0.793 | 0.897 |
| SIRT2 | IL1B | 0.004 | -0.031 | 0.040 | 0.802 | 0.897 |
| MMP.1 | IL1B | -0.020 | -0.178 | 0.138 | 0.802 | 0.897 |
| IL13 | IL1B | 0.005 | -0.036 | 0.047 | 0.803 | 0.897 |
| FGF.19 | IL1B | 0.015 | -0.105 | 0.134 | 0.809 | 0.897 |
| IL33 | IL1B | 0.005 | -0.041 | 0.052 | 0.829 | 0.908 |
| Beta.NGF | IL1B | -0.002 | -0.019 | 0.016 | 0.842 | 0.909 |
| TNF | IL1B | 0.008 | -0.077 | 0.093 | 0.850 | 0.909 |
| ST1A1 | IL17A | 0.010 | -0.059 | 0.080 | 0.769 | 0.930 |
| IL5 | IL17A | 0.007 | -0.040 | 0.053 | 0.777 | 0.930 |
| IL.24 | IL17A | -0.010 | -0.084 | 0.064 | 0.791 | 0.930 |
| CCL28 | IL17A | 0.004 | -0.026 | 0.033 | 0.803 | 0.930 |
| MCP.1 | IL17A | -0.014 | -0.127 | 0.099 | 0.807 | 0.930 |
| FGF.21 | IL17A | 0.019 | -0.136 | 0.173 | 0.811 | 0.930 |
| IL33 | IL17A | -0.005 | -0.046 | 0.036 | 0.814 | 0.930 |
| SIRT2 | IL17A | -0.003 | -0.035 | 0.028 | 0.828 | 0.930 |
| IL10 | IL17A | -0.006 | -0.058 | 0.047 | 0.829 | 0.930 |
| OPG | IL17A | -0.006 | -0.069 | 0.058 | 0.861 | 0.930 |
| IL.18R1 | IL17A | -0.009 | -0.112 | 0.094 | 0.867 | 0.930 |
| CXCL6 | IL17A | 0.007 | -0.077 | 0.091 | 0.869 | 0.930 |
| TGF.alpha | IL17A | 0.006 | -0.066 | 0.078 | 0.869 | 0.930 |
| IL.20 | IL1B | 0.003 | -0.036 | 0.042 | 0.883 | 0.934 |
| MCP.2 | IL17A | 0.006 | -0.085 | 0.098 | 0.889 | 0.940 |
| CCL19 | IL6 | 0.081 | 0.010 | 0.153 | 0.025 | 0.954 |
| FGF.19 | IL6 | -0.113 | -0.215 | -0.011 | 0.030 | 0.954 |
| CXCL10 | IL6 | 0.073 | -0.018 | 0.163 | 0.114 | 0.954 |
| ST1A1 | IL6 | -0.054 | -0.121 | 0.014 | 0.119 | 0.954 |
| DNER | IL6 | -0.040 | -0.092 | 0.012 | 0.127 | 0.954 |
| EN.RAGE | IL6 | -0.054 | -0.124 | 0.016 | 0.128 | 0.954 |
| IL8 | IL6 | 0.077 | -0.025 | 0.180 | 0.136 | 0.954 |
| VEGFA | IL6 | -0.060 | -0.140 | 0.021 | 0.146 | 0.954 |
| SCF | IL6 | -0.053 | -0.126 | 0.020 | 0.153 | 0.954 |
| CDCP1 | IL6 | -0.035 | -0.083 | 0.013 | 0.153 | 0.954 |
| IL.20RA | IL6 | 0.025 | -0.010 | 0.061 | 0.157 | 0.954 |
| IL.12B | IL6 | 0.060 | -0.024 | 0.144 | 0.162 | 0.954 |
| CCL25 | IL6 | 0.046 | -0.019 | 0.111 | 0.165 | 0.954 |
| TSLP | IL6 | 0.031 | -0.015 | 0.076 | 0.185 | 0.954 |
| TGF.alpha | IL6 | -0.046 | -0.116 | 0.025 | 0.200 | 0.954 |
| CCL20 | IL6 | 0.064 | -0.035 | 0.164 | 0.204 | 0.954 |
| CXCL9 | IL6 | 0.040 | -0.027 | 0.107 | 0.244 | 0.954 |
| OPG | IL6 | -0.036 | -0.098 | 0.027 | 0.260 | 0.954 |
| MMP.10 | IL6 | 0.046 | -0.039 | 0.131 | 0.288 | 0.954 |
| CXCL5 | IL6 | -0.060 | -0.172 | 0.053 | 0.295 | 0.954 |
| IFN.gamma | IL6 | 0.074 | -0.071 | 0.219 | 0.315 | 0.954 |
| FGF.21 | IL6 | 0.075 | -0.076 | 0.226 | 0.326 | 0.954 |
| IL.20 | IL6 | -0.017 | -0.050 | 0.017 | 0.331 | 0.954 |
| IL10 | IL6 | -0.025 | -0.076 | 0.026 | 0.334 | 0.954 |
| CD5 | IL6 | 0.052 | -0.057 | 0.162 | 0.347 | 0.954 |
| MCP.4 | IL6 | 0.053 | -0.063 | 0.169 | 0.369 | 0.954 |
| LAP.TGF.beta.1 | IL6 | -0.027 | -0.086 | 0.033 | 0.375 | 0.954 |
| TWEAK | IL6 | -0.023 | -0.076 | 0.029 | 0.381 | 0.954 |
| MCP.3 | IL6 | 0.045 | -0.061 | 0.150 | 0.404 | 0.954 |
| CD6 | IL6 | 0.040 | -0.061 | 0.142 | 0.434 | 0.954 |
| CCL4 | IL6 | 0.032 | -0.051 | 0.114 | 0.450 | 0.954 |
| IL4 | IL6 | 0.020 | -0.033 | 0.072 | 0.454 | 0.954 |
| TNFRSF9 | IL6 | -0.028 | -0.102 | 0.046 | 0.458 | 0.954 |
| TRANCE | IL6 | -0.029 | -0.106 | 0.048 | 0.463 | 0.954 |
| NT.3 | IL6 | 0.017 | -0.029 | 0.063 | 0.466 | 0.954 |
| MCP.2 | IL6 | 0.032 | -0.057 | 0.122 | 0.476 | 0.954 |
| IL5 | IL6 | -0.016 | -0.062 | 0.029 | 0.477 | 0.954 |
| FGF.23 | IL6 | -0.027 | -0.101 | 0.048 | 0.481 | 0.954 |
| CASP.8 | IL6 | -0.018 | -0.068 | 0.033 | 0.490 | 0.954 |
| CST5 | IL6 | 0.037 | -0.071 | 0.145 | 0.497 | 0.954 |
| IL.22.RA1 | IL6 | -0.027 | -0.107 | 0.053 | 0.509 | 0.954 |
| TNFSF14 | IL6 | -0.028 | -0.113 | 0.057 | 0.516 | 0.954 |
| CXCL11 | IL6 | 0.044 | -0.090 | 0.179 | 0.517 | 0.954 |
| ADA | IL6 | 0.011 | -0.027 | 0.050 | 0.558 | 0.954 |
| X4E.BP1 | IL6 | -0.008 | -0.034 | 0.019 | 0.568 | 0.954 |
| NRTN | IL6 | 0.008 | -0.021 | 0.038 | 0.577 | 0.954 |
| IL.10RA | IL6 | -0.008 | -0.037 | 0.021 | 0.577 | 0.954 |
| MMP.1 | IL6 | 0.039 | -0.098 | 0.175 | 0.578 | 0.954 |
| CSF.1 | IL6 | 0.015 | -0.038 | 0.068 | 0.581 | 0.954 |
| Beta.NGF | IL6 | -0.004 | -0.019 | 0.011 | 0.583 | 0.954 |
| uPA | IL6 | -0.020 | -0.091 | 0.051 | 0.584 | 0.954 |
| CCL11 | IL6 | 0.027 | -0.071 | 0.125 | 0.587 | 0.954 |
| PD.L1 | IL6 | 0.017 | -0.045 | 0.078 | 0.588 | 0.954 |
| IL.17C | IL6 | 0.028 | -0.078 | 0.135 | 0.597 | 0.954 |
| IL7 | IL6 | -0.011 | -0.053 | 0.030 | 0.599 | 0.954 |
| IL2 | IL6 | -0.011 | -0.054 | 0.032 | 0.608 | 0.954 |
| IL.15RA | IL6 | -0.011 | -0.053 | 0.032 | 0.622 | 0.954 |
| OSM | IL6 | 0.022 | -0.067 | 0.111 | 0.623 | 0.954 |
| GDNF | IL6 | 0.012 | -0.037 | 0.062 | 0.631 | 0.954 |
| CXCL6 | IL6 | 0.020 | -0.062 | 0.102 | 0.635 | 0.954 |
| TRAIL | IL6 | 0.016 | -0.051 | 0.083 | 0.639 | 0.954 |
| CXCL1 | IL6 | 0.015 | -0.049 | 0.079 | 0.645 | 0.954 |
| IL6 | IL6 | -0.027 | -0.146 | 0.093 | 0.660 | 0.954 |
| CX3CL1 | IL6 | 0.013 | -0.047 | 0.074 | 0.665 | 0.954 |
| SLAMF1 | IL6 | 0.010 | -0.038 | 0.059 | 0.677 | 0.954 |
| CD8A | IL6 | 0.018 | -0.069 | 0.105 | 0.686 | 0.954 |
| IL18 | IL6 | -0.016 | -0.097 | 0.066 | 0.706 | 0.954 |
| AXIN1 | IL6 | 0.013 | -0.055 | 0.081 | 0.712 | 0.954 |
| IL.1.alpha | IL6 | 0.036 | -0.168 | 0.241 | 0.726 | 0.954 |
| SIRT2 | IL6 | -0.005 | -0.036 | 0.025 | 0.739 | 0.954 |
| CD244 | IL6 | -0.013 | -0.090 | 0.064 | 0.742 | 0.954 |
| TNFB | IL6 | -0.007 | -0.051 | 0.037 | 0.747 | 0.954 |
| IL.10RB | IL6 | -0.009 | -0.064 | 0.047 | 0.759 | 0.954 |
| IL.24 | IL6 | 0.011 | -0.062 | 0.084 | 0.767 | 0.954 |
| LIF.R | IL6 | -0.003 | -0.026 | 0.020 | 0.784 | 0.955 |
| HGF | IL6 | 0.011 | -0.073 | 0.096 | 0.789 | 0.955 |
| IL2 | IL1B | -0.003 | -0.052 | 0.047 | 0.913 | 0.955 |
| TWEAK | IL1B | 0.003 | -0.058 | 0.064 | 0.927 | 0.955 |
| LAP.TGF.beta.1 | IL1B | 0.003 | -0.066 | 0.072 | 0.936 | 0.955 |
| IL.12B | IL1B | -0.003 | -0.102 | 0.095 | 0.944 | 0.955 |
| IL.24 | IL1B | -0.002 | -0.086 | 0.082 | 0.957 | 0.957 |
| IL33 | IL6 | 0.005 | -0.035 | 0.045 | 0.801 | 0.957 |
| MCP.1 | IL6 | 0.013 | -0.098 | 0.123 | 0.823 | 0.959 |
| IL.2RB | IL6 | -0.004 | -0.039 | 0.032 | 0.843 | 0.959 |
| CCL23 | IL6 | -0.008 | -0.097 | 0.082 | 0.867 | 0.959 |
| TNF | IL6 | -0.006 | -0.079 | 0.067 | 0.870 | 0.959 |
| Flt3L | IL6 | 0.005 | -0.057 | 0.068 | 0.871 | 0.959 |
| CCL28 | IL6 | 0.002 | -0.027 | 0.031 | 0.873 | 0.959 |
| CD40 | IL6 | -0.006 | -0.086 | 0.075 | 0.891 | 0.959 |
| FGF.5 | IL6 | 0.003 | -0.050 | 0.056 | 0.900 | 0.959 |
| ARTN | IL6 | -0.003 | -0.051 | 0.046 | 0.911 | 0.959 |
| LIF | IL6 | 0.002 | -0.035 | 0.038 | 0.917 | 0.959 |
| STAMBP | IL6 | -0.001 | -0.020 | 0.018 | 0.918 | 0.959 |
| CX3CL1 | IL17A | -0.003 | -0.065 | 0.059 | 0.929 | 0.961 |
| IL.20RA | IL17A | -0.002 | -0.038 | 0.035 | 0.933 | 0.961 |
| CXCL1 | IL17A | 0.002 | -0.063 | 0.067 | 0.948 | 0.961 |
| LIF | IL17A | 0.001 | -0.036 | 0.038 | 0.951 | 0.961 |
| CST5 | CRP | -0.128 | -0.240 | -0.017 | 0.024 | 0.961 |
| X4E.BP1 | CRP | 0.030 | 0.002 | 0.057 | 0.035 | 0.961 |
| IL.17A | CRP | -0.048 | -0.093 | -0.003 | 0.037 | 0.961 |
| Flt3L | CRP | -0.064 | -0.129 | 0.001 | 0.053 | 0.961 |
| SIRT2 | CRP | -0.029 | -0.060 | 0.003 | 0.075 | 0.961 |
| MMP.1 | CRP | -0.128 | -0.270 | 0.015 | 0.079 | 0.961 |
| uPA | CRP | -0.066 | -0.140 | 0.008 | 0.082 | 0.961 |
| TWEAK | CRP | -0.047 | -0.102 | 0.008 | 0.093 | 0.961 |
| IL6 | CRP | -0.105 | -0.229 | 0.019 | 0.096 | 0.961 |
| FGF.19 | CRP | -0.088 | -0.195 | 0.020 | 0.110 | 0.961 |
| IL4 | CRP | -0.043 | -0.097 | 0.012 | 0.126 | 0.961 |
| LIF.R | CRP | -0.018 | -0.042 | 0.006 | 0.142 | 0.961 |
| IL.20 | CRP | -0.026 | -0.061 | 0.009 | 0.144 | 0.961 |
| MCP.3 | CRP | 0.080 | -0.029 | 0.190 | 0.150 | 0.961 |
| LAP.TGF.beta.1 | CRP | -0.041 | -0.103 | 0.021 | 0.198 | 0.961 |
| VEGFA | CRP | -0.055 | -0.140 | 0.029 | 0.199 | 0.961 |
| MMP.10 | CRP | 0.055 | -0.034 | 0.144 | 0.224 | 0.961 |
| TSLP | CRP | -0.030 | -0.077 | 0.018 | 0.224 | 0.961 |
| IL.1.alpha | CRP | 0.131 | -0.083 | 0.345 | 0.228 | 0.961 |
| HGF | CRP | -0.052 | -0.140 | 0.036 | 0.245 | 0.961 |
| IL10 | CRP | 0.031 | -0.022 | 0.085 | 0.249 | 0.961 |
| CXCL1 | CRP | -0.038 | -0.104 | 0.029 | 0.265 | 0.961 |
| FGF.5 | CRP | -0.030 | -0.086 | 0.025 | 0.283 | 0.961 |
| IL.17C | CRP | 0.059 | -0.052 | 0.170 | 0.299 | 0.961 |
| CCL11 | CRP | -0.053 | -0.156 | 0.049 | 0.306 | 0.961 |
| TNFSF14 | CRP | -0.046 | -0.136 | 0.043 | 0.307 | 0.961 |
| NT.3 | CRP | 0.022 | -0.027 | 0.070 | 0.381 | 0.961 |
| TRAIL | CRP | -0.029 | -0.100 | 0.041 | 0.415 | 0.961 |
| IL.10RB | CRP | 0.024 | -0.035 | 0.082 | 0.424 | 0.961 |
| ARTN | CRP | 0.021 | -0.030 | 0.072 | 0.425 | 0.961 |
| MCP.2 | CRP | 0.037 | -0.056 | 0.131 | 0.434 | 0.961 |
| IL.15RA | CRP | -0.017 | -0.062 | 0.027 | 0.445 | 0.961 |
| CASP.8 | CRP | -0.020 | -0.073 | 0.033 | 0.450 | 0.961 |
| CD244 | CRP | -0.031 | -0.112 | 0.050 | 0.451 | 0.961 |
| DNER | CRP | -0.020 | -0.074 | 0.035 | 0.477 | 0.961 |
| ADA | CRP | -0.014 | -0.055 | 0.026 | 0.481 | 0.961 |
| CCL25 | CRP | -0.024 | -0.093 | 0.044 | 0.485 | 0.961 |
| CXCL9 | CRP | -0.025 | -0.096 | 0.046 | 0.486 | 0.961 |
| TNF | CRP | 0.026 | -0.050 | 0.103 | 0.497 | 0.961 |
| TGF.alpha | CRP | 0.025 | -0.049 | 0.100 | 0.501 | 0.961 |
| IL.24 | CRP | -0.026 | -0.102 | 0.050 | 0.506 | 0.961 |
| LIF | CRP | 0.012 | -0.026 | 0.051 | 0.523 | 0.961 |
| CD8A | CRP | -0.029 | -0.120 | 0.062 | 0.528 | 0.961 |
| IL.10RA | CRP | -0.010 | -0.040 | 0.021 | 0.531 | 0.961 |
| CCL19 | CRP | 0.024 | -0.052 | 0.100 | 0.532 | 0.961 |
| IL.22.RA1 | CRP | -0.026 | -0.111 | 0.058 | 0.539 | 0.961 |
| IL18 | CRP | -0.026 | -0.112 | 0.059 | 0.543 | 0.961 |
| CDCP1 | CRP | -0.015 | -0.066 | 0.035 | 0.546 | 0.961 |
| AXIN1 | CRP | -0.021 | -0.093 | 0.050 | 0.558 | 0.961 |
| IL7 | CRP | -0.013 | -0.056 | 0.031 | 0.560 | 0.961 |
| STAMBP | CRP | -0.006 | -0.026 | 0.014 | 0.571 | 0.961 |
| MCP.4 | CRP | -0.034 | -0.156 | 0.088 | 0.578 | 0.961 |
| SCF | CRP | -0.021 | -0.098 | 0.056 | 0.596 | 0.961 |
| OSM | CRP | 0.025 | -0.069 | 0.118 | 0.599 | 0.961 |
| OPG | CRP | -0.017 | -0.083 | 0.048 | 0.603 | 0.961 |
| CCL20 | CRP | 0.027 | -0.078 | 0.132 | 0.612 | 0.961 |
| FGF.21 | CRP | -0.039 | -0.198 | 0.119 | 0.626 | 0.961 |
| TNFRSF9 | CRP | -0.019 | -0.097 | 0.059 | 0.632 | 0.961 |
| GDNF | CRP | -0.012 | -0.064 | 0.040 | 0.643 | 0.961 |
| CXCL5 | CRP | 0.027 | -0.091 | 0.146 | 0.650 | 0.961 |
| CD5 | CRP | -0.027 | -0.142 | 0.089 | 0.650 | 0.961 |
| IL8 | CRP | 0.024 | -0.083 | 0.132 | 0.654 | 0.961 |
| CXCL11 | CRP | -0.032 | -0.173 | 0.110 | 0.658 | 0.961 |
| IL5 | CRP | -0.010 | -0.057 | 0.038 | 0.695 | 0.968 |
| IL.12B | CRP | -0.016 | -0.105 | 0.073 | 0.716 | 0.968 |
| CXCL6 | CRP | -0.016 | -0.102 | 0.070 | 0.717 | 0.968 |
| IL.20RA | CRP | 0.006 | -0.031 | 0.044 | 0.736 | 0.968 |
| CCL28 | CRP | 0.005 | -0.025 | 0.035 | 0.740 | 0.968 |
| PD.L1 | CRP | 0.011 | -0.054 | 0.075 | 0.744 | 0.968 |
| CD40 | CRP | -0.014 | -0.098 | 0.071 | 0.751 | 0.968 |
| IL.2RB | CRP | 0.006 | -0.032 | 0.043 | 0.754 | 0.968 |
| CCL23 | CRP | -0.015 | -0.109 | 0.079 | 0.759 | 0.968 |
| CCL4 | CRP | 0.013 | -0.074 | 0.099 | 0.773 | 0.968 |
| CD6 | CRP | -0.015 | -0.122 | 0.091 | 0.779 | 0.968 |
| Beta.NGF | CRP | 0.002 | -0.014 | 0.018 | 0.791 | 0.970 |
| CX3CL1 | CRP | -0.008 | -0.072 | 0.056 | 0.806 | 0.975 |
| TNFB | CRP | 0.005 | -0.041 | 0.051 | 0.836 | 0.977 |
| IL33 | CRP | 0.004 | -0.038 | 0.047 | 0.837 | 0.977 |
| FGF.23 | CRP | 0.008 | -0.070 | 0.087 | 0.839 | 0.977 |
| CCL23 | IL17A | 0.001 | -0.090 | 0.093 | 0.980 | 0.980 |
| IL13 | IL6 | -0.001 | -0.037 | 0.035 | 0.960 | 0.984 |
| IL.18R1 | IL6 | 0.002 | -0.099 | 0.103 | 0.964 | 0.984 |
| CCL3 | IL6 | 0.003 | -0.174 | 0.180 | 0.974 | 0.984 |
| IL.17A | IL6 | 0.000 | -0.044 | 0.043 | 0.988 | 0.988 |
| MCP.1 | CRP | -0.009 | -0.125 | 0.107 | 0.878 | 0.997 |
| IFN.gamma | CRP | -0.011 | -0.164 | 0.141 | 0.883 | 0.997 |
| ST1A1 | CRP | -0.005 | -0.076 | 0.067 | 0.899 | 0.997 |
| CCL3 | CRP | 0.011 | -0.175 | 0.197 | 0.909 | 0.997 |
| IL2 | CRP | -0.003 | -0.048 | 0.042 | 0.911 | 0.997 |
| NRTN | CRP | 0.001 | -0.030 | 0.032 | 0.937 | 0.997 |
| TRANCE | CRP | 0.003 | -0.078 | 0.084 | 0.947 | 0.997 |
| IL.18R1 | CRP | 0.003 | -0.103 | 0.109 | 0.950 | 0.997 |
| IL13 | CRP | 0.001 | -0.037 | 0.039 | 0.963 | 0.997 |
| CSF.1 | CRP | -0.001 | -0.057 | 0.055 | 0.976 | 0.997 |
| SLAMF1 | CRP | 0.000 | -0.051 | 0.050 | 0.987 | 0.997 |
| CXCL10 | CRP | 0.001 | -0.095 | 0.096 | 0.991 | 0.997 |
| EN.RAGE | CRP | 0.000 | -0.074 | 0.074 | 0.997 | 0.997 |

# **Supplementary Table 12.** Regression estimates for associations between continuous maternal inflammation levels measured among third trimester subset (n = 235), further adjusted for maternal age, pre-pregnancy BMI, and race and ethnicity, ordered by adjusted p-value

| **Neonatal  inflammatory marker** | **Maternal Inflammatory Marker** | **Beta** | **95% CI Low** | **95% CI High** | **p-value** | **Adjusted p-value** |
| --- | --- | --- | --- | --- | --- | --- |
| CCL28 | IL17A | 0.055 | 0.018 | 0.091 | 0.004 | 0.357 |
| IL.17C | CRP | -0.159 | -0.273 | -0.045 | 0.007 | 0.396 |
| CCL4 | CRP | -0.105 | -0.188 | -0.022 | 0.013 | 0.396 |
| LIF.R | CRP | -0.033 | -0.061 | -0.006 | 0.018 | 0.396 |
| SCF | CRP | -0.081 | -0.149 | -0.013 | 0.019 | 0.396 |
| CXCL6 | CRP | -0.108 | -0.203 | -0.014 | 0.025 | 0.396 |
| IL13 | CRP | -0.043 | -0.082 | -0.003 | 0.033 | 0.396 |
| TRAIL | CRP | -0.076 | -0.150 | -0.002 | 0.044 | 0.396 |
| VEGFA | CRP | -0.090 | -0.178 | -0.002 | 0.044 | 0.396 |
| CXCL10 | CRP | -0.100 | -0.200 | -0.001 | 0.048 | 0.396 |
| MMP.1 | CRP | -0.134 | -0.267 | -0.001 | 0.049 | 0.396 |
| CDCP1 | CRP | -0.062 | -0.125 | 0.001 | 0.052 | 0.396 |
| TWEAK | CRP | -0.062 | -0.126 | 0.001 | 0.055 | 0.396 |
| CCL25 | CRP | -0.071 | -0.145 | 0.003 | 0.061 | 0.396 |
| IL.17A | CRP | -0.047 | -0.098 | 0.003 | 0.064 | 0.396 |
| IL6 | CRP | -0.115 | -0.237 | 0.007 | 0.065 | 0.396 |
| CXCL9 | CRP | -0.066 | -0.140 | 0.008 | 0.079 | 0.449 |
| TNF | CRP | -0.068 | -0.144 | 0.009 | 0.083 | 0.449 |
| CD8A | CRP | -0.082 | -0.177 | 0.013 | 0.090 | 0.449 |
| TNFSF14 | CRP | -0.080 | -0.176 | 0.015 | 0.099 | 0.449 |
| IL.20RA | CRP | -0.034 | -0.075 | 0.007 | 0.101 | 0.449 |
| LAP.TGF.beta.1 | CRP | -0.053 | -0.117 | 0.011 | 0.106 | 0.449 |
| CD244 | CRP | -0.068 | -0.152 | 0.015 | 0.108 | 0.449 |
| IL8 | CRP | -0.094 | -0.212 | 0.024 | 0.117 | 0.449 |
| X4E.BP1 | CRP | -0.022 | -0.049 | 0.006 | 0.118 | 0.449 |
| CXCL1 | CRP | -0.060 | -0.137 | 0.016 | 0.123 | 0.449 |
| TRANCE | CRP | -0.060 | -0.137 | 0.017 | 0.127 | 0.449 |
| IL4 | CRP | -0.034 | -0.079 | 0.011 | 0.133 | 0.454 |
| PD.L1 | CRP | -0.053 | -0.124 | 0.018 | 0.142 | 0.465 |
| MMP.10 | CRP | -0.059 | -0.140 | 0.023 | 0.157 | 0.478 |
| IL.10RB | CRP | -0.039 | -0.093 | 0.016 | 0.160 | 0.478 |
| TGF.alpha | CRP | -0.061 | -0.147 | 0.025 | 0.163 | 0.478 |
| CD40 | CRP | -0.064 | -0.155 | 0.028 | 0.171 | 0.478 |
| TNFRSF9 | CRP | -0.051 | -0.126 | 0.023 | 0.175 | 0.478 |
| IL7 | CRP | -0.032 | -0.078 | 0.014 | 0.177 | 0.478 |
| DNER | CRP | -0.036 | -0.090 | 0.019 | 0.199 | 0.499 |
| CCL19 | CRP | -0.046 | -0.117 | 0.025 | 0.200 | 0.499 |
| CD5 | CRP | -0.073 | -0.187 | 0.041 | 0.208 | 0.499 |
| CCL3 | CRP | -0.107 | -0.275 | 0.060 | 0.209 | 0.499 |
| ST1A1 | CRP | -0.046 | -0.119 | 0.026 | 0.211 | 0.499 |
| LIF | CRP | -0.021 | -0.055 | 0.013 | 0.232 | 0.511 |
| OSM | CRP | -0.062 | -0.167 | 0.042 | 0.240 | 0.511 |
| SLAMF1 | CRP | -0.031 | -0.084 | 0.021 | 0.242 | 0.511 |
| IL.12B | CRP | -0.050 | -0.135 | 0.035 | 0.247 | 0.511 |
| Beta.NGF | CRP | -0.009 | -0.024 | 0.006 | 0.248 | 0.511 |
| uPA | CRP | -0.045 | -0.122 | 0.032 | 0.251 | 0.511 |
| CX3CL1 | CRP | -0.036 | -0.098 | 0.026 | 0.255 | 0.511 |
| OPG | CRP | -0.035 | -0.098 | 0.027 | 0.269 | 0.526 |
| IL5 | CRP | 0.029 | -0.023 | 0.082 | 0.277 | 0.530 |
| IL.24 | CRP | -0.041 | -0.116 | 0.035 | 0.288 | 0.540 |
| ADA | CRP | 0.021 | -0.019 | 0.060 | 0.306 | 0.564 |
| CSF.1 | CRP | -0.028 | -0.083 | 0.028 | 0.324 | 0.574 |
| CXCL5 | CRP | -0.064 | -0.194 | 0.067 | 0.338 | 0.574 |
| CST5 | CRP | -0.064 | -0.196 | 0.069 | 0.345 | 0.574 |
| IL18 | CRP | 0.044 | -0.049 | 0.137 | 0.352 | 0.574 |
| IL.18R1 | CRP | -0.049 | -0.152 | 0.054 | 0.353 | 0.574 |
| IL.22.RA1 | CRP | -0.037 | -0.117 | 0.042 | 0.357 | 0.574 |
| CD6 | CRP | -0.050 | -0.157 | 0.057 | 0.358 | 0.574 |
| IL10 | CRP | 0.022 | -0.026 | 0.070 | 0.367 | 0.574 |
| TNFB | CRP | -0.019 | -0.061 | 0.023 | 0.370 | 0.574 |
| IL2 | CRP | -0.022 | -0.070 | 0.026 | 0.374 | 0.574 |
| CCL23 | CRP | -0.040 | -0.133 | 0.053 | 0.394 | 0.595 |
| CXCL9 | IL6 | 0.079 | 0.008 | 0.149 | 0.029 | 0.600 |
| TNF | IL6 | 0.078 | 0.006 | 0.150 | 0.033 | 0.600 |
| HGF | IL6 | -0.101 | -0.194 | -0.008 | 0.033 | 0.600 |
| TWEAK | IL6 | 0.065 | 0.005 | 0.125 | 0.033 | 0.600 |
| MCP.2 | IL6 | 0.078 | 0.006 | 0.150 | 0.035 | 0.600 |
| IL.22.RA1 | IL6 | 0.074 | -0.001 | 0.148 | 0.052 | 0.600 |
| ADA | IL6 | 0.035 | -0.002 | 0.072 | 0.067 | 0.600 |
| IL.2RB | IL6 | 0.033 | -0.003 | 0.069 | 0.073 | 0.600 |
| CCL11 | IL6 | 0.093 | -0.012 | 0.198 | 0.081 | 0.600 |
| GDNF | IL6 | 0.044 | -0.006 | 0.095 | 0.086 | 0.600 |
| VEGFA | IL6 | -0.072 | -0.155 | 0.011 | 0.090 | 0.600 |
| MCP.1 | IL6 | 0.091 | -0.017 | 0.198 | 0.099 | 0.600 |
| IL.20 | IL6 | 0.025 | -0.006 | 0.057 | 0.113 | 0.600 |
| CDCP1 | IL6 | 0.047 | -0.012 | 0.107 | 0.115 | 0.600 |
| IL5 | IL6 | 0.039 | -0.010 | 0.089 | 0.118 | 0.600 |
| CX3CL1 | IL6 | 0.046 | -0.012 | 0.105 | 0.121 | 0.600 |
| IL.24 | IL6 | 0.056 | -0.015 | 0.127 | 0.123 | 0.600 |
| CST5 | IL6 | 0.096 | -0.028 | 0.221 | 0.128 | 0.600 |
| IL4 | IL6 | 0.033 | -0.010 | 0.075 | 0.129 | 0.600 |
| TNFSF14 | IL6 | -0.069 | -0.159 | 0.021 | 0.130 | 0.600 |
| FGF.19 | CRP | 0.045 | -0.067 | 0.157 | 0.427 | 0.634 |
| IL.10RA | CRP | -0.012 | -0.042 | 0.018 | 0.437 | 0.638 |
| EN.RAGE | CRP | -0.032 | -0.116 | 0.051 | 0.448 | 0.641 |
| FGF.5 | CRP | -0.021 | -0.075 | 0.034 | 0.456 | 0.641 |
| MCP.1 | CRP | 0.043 | -0.072 | 0.157 | 0.462 | 0.641 |
| IL.20 | CRP | -0.012 | -0.046 | 0.021 | 0.467 | 0.641 |
| IL.1.alpha | CRP | 0.065 | -0.118 | 0.247 | 0.485 | 0.653 |
| AXIN1 | CRP | -0.030 | -0.114 | 0.055 | 0.489 | 0.653 |
| MCP.3 | CRP | -0.033 | -0.131 | 0.065 | 0.504 | 0.653 |
| CCL28 | CRP | -0.014 | -0.056 | 0.028 | 0.504 | 0.653 |
| IL.2RB | CRP | -0.013 | -0.052 | 0.026 | 0.512 | 0.654 |
| MCP.4 | IL6 | 0.078 | -0.029 | 0.186 | 0.153 | 0.671 |
| ARTN | IL6 | 0.035 | -0.014 | 0.085 | 0.160 | 0.671 |
| HGF | CRP | -0.031 | -0.131 | 0.068 | 0.537 | 0.676 |
| CXCL6 | IL6 | -0.061 | -0.151 | 0.028 | 0.179 | 0.679 |
| IL10 | IL6 | 0.031 | -0.014 | 0.076 | 0.182 | 0.679 |
| ST1A1 | IL6 | -0.046 | -0.115 | 0.022 | 0.184 | 0.679 |
| NRTN | CRP | -0.011 | -0.046 | 0.025 | 0.557 | 0.692 |
| MCP.4 | IL17A | 0.117 | 0.016 | 0.219 | 0.024 | 0.728 |
| GDNF | IL17A | 0.049 | 0.000 | 0.097 | 0.048 | 0.728 |
| IL2 | IL17A | 0.043 | 0.000 | 0.086 | 0.049 | 0.728 |
| STAMBP | IL17A | 0.017 | -0.001 | 0.036 | 0.071 | 0.728 |
| NT.3 | IL17A | 0.040 | -0.008 | 0.088 | 0.100 | 0.728 |
| VEGFA | IL17A | -0.066 | -0.145 | 0.014 | 0.104 | 0.728 |
| ST1A1 | IL17A | -0.054 | -0.119 | 0.011 | 0.104 | 0.728 |
| NRTN | IL17A | 0.026 | -0.006 | 0.058 | 0.107 | 0.728 |
| FGF.19 | IL17A | 0.080 | -0.021 | 0.181 | 0.119 | 0.728 |
| CXCL10 | IL17A | 0.071 | -0.019 | 0.160 | 0.121 | 0.728 |
| IL33 | IL17A | 0.029 | -0.009 | 0.066 | 0.133 | 0.728 |
| TSLP | IL17A | -0.035 | -0.081 | 0.011 | 0.138 | 0.728 |
| HGF | IL17A | -0.066 | -0.155 | 0.023 | 0.145 | 0.728 |
| IL.17C | IL17A | 0.077 | -0.027 | 0.181 | 0.146 | 0.728 |
| FGF.23 | IL17A | 0.038 | -0.013 | 0.090 | 0.146 | 0.728 |
| CST5 | IL17A | 0.087 | -0.031 | 0.205 | 0.149 | 0.728 |
| IL.1.alpha | IL17A | 0.119 | -0.044 | 0.282 | 0.152 | 0.728 |
| IL5 | IL17A | 0.034 | -0.013 | 0.081 | 0.154 | 0.728 |
| MCP.2 | IL17A | 0.048 | -0.021 | 0.118 | 0.169 | 0.728 |
| MMP.10 | IL17A | 0.051 | -0.022 | 0.124 | 0.173 | 0.728 |
| IL.20RA | IL17A | 0.025 | -0.011 | 0.062 | 0.174 | 0.728 |
| CASP.8 | IL17A | -0.034 | -0.085 | 0.016 | 0.183 | 0.731 |
| IL4 | IL17A | 0.026 | -0.014 | 0.067 | 0.198 | 0.753 |
| IL.10RB | IL17A | -0.032 | -0.081 | 0.017 | 0.205 | 0.753 |
| CCL25 | IL17A | -0.042 | -0.109 | 0.025 | 0.215 | 0.753 |
| CXCL9 | IL17A | 0.039 | -0.029 | 0.107 | 0.256 | 0.753 |
| LIF.R | IL17A | -0.015 | -0.040 | 0.011 | 0.257 | 0.753 |
| FGF.21 | IL17A | -0.077 | -0.214 | 0.059 | 0.266 | 0.753 |
| IL18 | IL17A | -0.045 | -0.129 | 0.038 | 0.284 | 0.753 |
| CX3CL1 | IL17A | 0.030 | -0.026 | 0.086 | 0.286 | 0.753 |
| uPA | IL17A | -0.037 | -0.107 | 0.032 | 0.293 | 0.753 |
| TNFSF14 | IL17A | -0.046 | -0.132 | 0.040 | 0.293 | 0.753 |
| IL.2RB | IL17A | 0.018 | -0.016 | 0.053 | 0.296 | 0.753 |
| IL6 | IL17A | 0.058 | -0.052 | 0.169 | 0.298 | 0.753 |
| MCP.3 | IL17A | 0.046 | -0.042 | 0.134 | 0.302 | 0.753 |
| SCF | IL17A | -0.032 | -0.094 | 0.029 | 0.303 | 0.753 |
| FGF.21 | IL6 | -0.090 | -0.233 | 0.053 | 0.216 | 0.764 |
| CCL3 | IL17A | 0.074 | -0.076 | 0.225 | 0.332 | 0.780 |
| IL.24 | IL17A | -0.033 | -0.101 | 0.035 | 0.342 | 0.780 |
| SIRT2 | IL17A | 0.013 | -0.015 | 0.041 | 0.351 | 0.780 |
| X4E.BP1 | IL17A | 0.011 | -0.013 | 0.036 | 0.360 | 0.780 |
| DNER | IL17A | -0.023 | -0.072 | 0.026 | 0.361 | 0.780 |
| OSM | IL17A | -0.043 | -0.136 | 0.051 | 0.371 | 0.780 |
| LIF | IL17A | 0.014 | -0.017 | 0.044 | 0.382 | 0.780 |
| IL13 | IL17A | 0.015 | -0.020 | 0.051 | 0.396 | 0.780 |
| PD.L1 | IL17A | -0.027 | -0.091 | 0.037 | 0.407 | 0.780 |
| TNFB | IL17A | -0.016 | -0.053 | 0.022 | 0.419 | 0.780 |
| CD244 | IL17A | -0.031 | -0.106 | 0.045 | 0.422 | 0.780 |
| CSF.1 | IL17A | -0.020 | -0.070 | 0.030 | 0.423 | 0.780 |
| IL.10RA | IL17A | -0.011 | -0.038 | 0.016 | 0.433 | 0.780 |
| TNFRSF9 | IL17A | -0.026 | -0.093 | 0.041 | 0.449 | 0.780 |
| IL.22.RA1 | IL17A | 0.027 | -0.044 | 0.099 | 0.451 | 0.780 |
| ADA | IL17A | 0.013 | -0.022 | 0.049 | 0.463 | 0.780 |
| FGF.5 | IL17A | 0.018 | -0.031 | 0.067 | 0.464 | 0.780 |
| AXIN1 | IL17A | -0.027 | -0.103 | 0.049 | 0.484 | 0.780 |
| Flt3L | IL17A | 0.021 | -0.040 | 0.082 | 0.489 | 0.780 |
| IL.18R1 | IL17A | -0.032 | -0.125 | 0.060 | 0.492 | 0.780 |
| EN.RAGE | IL17A | -0.026 | -0.101 | 0.049 | 0.498 | 0.780 |
| CDCP1 | IL17A | 0.019 | -0.037 | 0.076 | 0.500 | 0.780 |
| IL.17C | IL6 | 0.067 | -0.042 | 0.176 | 0.229 | 0.781 |
| IL.20 | IL17A | 0.010 | -0.020 | 0.040 | 0.514 | 0.789 |
| CASP.8 | CRP | -0.013 | -0.070 | 0.043 | 0.644 | 0.790 |
| CCL28 | IL6 | 0.023 | -0.016 | 0.063 | 0.241 | 0.792 |
| FGF.23 | CRP | -0.012 | -0.070 | 0.045 | 0.673 | 0.800 |
| SIRT2 | CRP | -0.007 | -0.038 | 0.025 | 0.676 | 0.800 |
| NT.3 | CRP | -0.011 | -0.065 | 0.043 | 0.678 | 0.800 |
| ARTN | IL17A | 0.015 | -0.032 | 0.062 | 0.531 | 0.801 |
| TNF | IL17A | -0.021 | -0.090 | 0.048 | 0.545 | 0.801 |
| CCL4 | IL17A | 0.023 | -0.052 | 0.098 | 0.549 | 0.801 |
| CCL28 | IL1B | 0.046 | 0.007 | 0.085 | 0.020 | 0.803 |
| TNFRSF9 | IL1B | -0.072 | -0.142 | -0.002 | 0.043 | 0.803 |
| CSF.1 | IL1B | -0.053 | -0.104 | -0.001 | 0.046 | 0.803 |
| FGF.5 | IL1B | 0.050 | 0.000 | 0.101 | 0.052 | 0.803 |
| MCP.2 | IL1B | 0.070 | -0.002 | 0.142 | 0.058 | 0.803 |
| IL.20 | IL1B | 0.030 | -0.001 | 0.061 | 0.060 | 0.803 |
| IL.24 | IL1B | 0.067 | -0.003 | 0.138 | 0.061 | 0.803 |
| CCL20 | CRP | -0.023 | -0.135 | 0.090 | 0.692 | 0.806 |
| SLAMF1 | IL17A | 0.014 | -0.034 | 0.061 | 0.576 | 0.813 |
| MCP.1 | IL17A | 0.029 | -0.074 | 0.132 | 0.576 | 0.813 |
| CD5 | IL17A | -0.028 | -0.130 | 0.075 | 0.596 | 0.813 |
| CD8A | IL17A | 0.023 | -0.063 | 0.109 | 0.598 | 0.813 |
| LAP.TGF.beta.1 | IL17A | -0.015 | -0.073 | 0.042 | 0.601 | 0.813 |
| CCL11 | CRP | -0.021 | -0.132 | 0.090 | 0.711 | 0.818 |
| GDNF | CRP | 0.009 | -0.045 | 0.063 | 0.736 | 0.835 |
| IL7 | IL17A | 0.010 | -0.031 | 0.052 | 0.629 | 0.838 |
| SCF | IL6 | 0.037 | -0.028 | 0.101 | 0.265 | 0.838 |
| CCL20 | IL6 | 0.060 | -0.048 | 0.167 | 0.277 | 0.838 |
| IL.15RA | IL6 | -0.025 | -0.071 | 0.021 | 0.287 | 0.838 |
| IL.18R1 | IL6 | -0.050 | -0.148 | 0.047 | 0.310 | 0.838 |
| FGF.5 | IL6 | 0.025 | -0.026 | 0.076 | 0.338 | 0.838 |
| TSLP | IL6 | 0.023 | -0.025 | 0.072 | 0.348 | 0.838 |
| IL.1.alpha | IL6 | 0.081 | -0.091 | 0.253 | 0.355 | 0.838 |
| IL.12B | IL6 | 0.037 | -0.043 | 0.117 | 0.361 | 0.838 |
| CXCL11 | IL6 | 0.054 | -0.064 | 0.172 | 0.369 | 0.838 |
| CXCL5 | IL6 | -0.056 | -0.179 | 0.067 | 0.374 | 0.838 |
| TNFRSF9 | IL6 | -0.031 | -0.102 | 0.039 | 0.382 | 0.838 |
| IL.10RA | IL6 | 0.013 | -0.016 | 0.041 | 0.386 | 0.838 |
| STAMBP | IL6 | 0.009 | -0.011 | 0.028 | 0.390 | 0.838 |
| uPA | IL6 | -0.031 | -0.104 | 0.042 | 0.402 | 0.838 |
| CCL19 | IL6 | 0.028 | -0.038 | 0.095 | 0.403 | 0.838 |
| TRANCE | IL6 | 0.030 | -0.042 | 0.103 | 0.411 | 0.838 |
| MCP.3 | IL6 | 0.039 | -0.054 | 0.131 | 0.411 | 0.838 |
| CASP.8 | IL6 | -0.022 | -0.075 | 0.031 | 0.419 | 0.838 |
| CXCL10 | IL6 | 0.035 | -0.059 | 0.130 | 0.462 | 0.838 |
| OSM | IL6 | -0.037 | -0.135 | 0.062 | 0.465 | 0.838 |
| SIRT2 | IL6 | 0.010 | -0.019 | 0.040 | 0.490 | 0.838 |
| EN.RAGE | IL6 | -0.026 | -0.105 | 0.054 | 0.524 | 0.838 |
| Flt3L | IL6 | 0.020 | -0.044 | 0.084 | 0.535 | 0.838 |
| IL7 | IL6 | 0.014 | -0.030 | 0.057 | 0.539 | 0.838 |
| LAP.TGF.beta.1 | IL6 | 0.019 | -0.042 | 0.079 | 0.544 | 0.838 |
| IL18 | IL6 | -0.027 | -0.115 | 0.061 | 0.546 | 0.838 |
| FGF.19 | IL6 | 0.032 | -0.074 | 0.139 | 0.550 | 0.838 |
| IL2 | IL6 | 0.014 | -0.032 | 0.060 | 0.551 | 0.838 |
| CXCL1 | IL6 | -0.022 | -0.095 | 0.051 | 0.554 | 0.838 |
| TGF.alpha | IL6 | -0.023 | -0.104 | 0.059 | 0.581 | 0.838 |
| SLAMF1 | IL6 | 0.013 | -0.036 | 0.063 | 0.595 | 0.838 |
| IL13 | IL6 | 0.010 | -0.027 | 0.047 | 0.597 | 0.838 |
| CCL3 | IL6 | -0.042 | -0.200 | 0.116 | 0.602 | 0.838 |
| TNFB | IL6 | 0.010 | -0.029 | 0.050 | 0.605 | 0.838 |
| CD244 | IL6 | -0.021 | -0.100 | 0.058 | 0.605 | 0.838 |
| IL.10RB | IL6 | -0.013 | -0.065 | 0.038 | 0.610 | 0.838 |
| CD8A | IL6 | 0.023 | -0.067 | 0.113 | 0.620 | 0.838 |
| LIF.R | IL6 | 0.006 | -0.020 | 0.033 | 0.631 | 0.838 |
| NT.3 | IL6 | -0.012 | -0.063 | 0.039 | 0.634 | 0.838 |
| CCL25 | IL6 | -0.017 | -0.087 | 0.054 | 0.641 | 0.838 |
| FGF.23 | IL6 | 0.013 | -0.042 | 0.067 | 0.647 | 0.838 |
| OPG | IL6 | 0.014 | -0.046 | 0.073 | 0.652 | 0.838 |
| LIF | IL6 | 0.007 | -0.025 | 0.039 | 0.657 | 0.838 |
| CCL23 | IL6 | -0.020 | -0.107 | 0.068 | 0.661 | 0.838 |
| DNER | IL6 | -0.011 | -0.063 | 0.040 | 0.665 | 0.838 |
| PD.L1 | IL6 | -0.013 | -0.081 | 0.054 | 0.697 | 0.852 |
| Beta.NGF | IL6 | -0.003 | -0.017 | 0.012 | 0.702 | 0.852 |
| CD6 | IL6 | 0.019 | -0.081 | 0.120 | 0.704 | 0.852 |
| CXCL5 | IL17A | 0.026 | -0.091 | 0.144 | 0.661 | 0.868 |
| CCL19 | IL17A | 0.014 | -0.050 | 0.077 | 0.670 | 0.868 |
| IFN.gamma | IL17A | 0.030 | -0.116 | 0.176 | 0.686 | 0.877 |
| AXIN1 | IL6 | -0.014 | -0.093 | 0.066 | 0.740 | 0.878 |
| CD40 | IL6 | 0.014 | -0.072 | 0.101 | 0.744 | 0.878 |
| IL.17A | IL17A | 0.008 | -0.037 | 0.054 | 0.723 | 0.911 |
| MMP.1 | IL17A | 0.020 | -0.101 | 0.140 | 0.747 | 0.912 |
| Beta.NGF | IL17A | 0.002 | -0.012 | 0.016 | 0.755 | 0.912 |
| TRAIL | IL17A | 0.011 | -0.057 | 0.078 | 0.757 | 0.912 |
| CD6 | IL17A | -0.015 | -0.111 | 0.081 | 0.763 | 0.912 |
| CXCL6 | IL17A | -0.012 | -0.098 | 0.073 | 0.775 | 0.914 |
| GDNF | IL1B | 0.042 | -0.009 | 0.092 | 0.104 | 0.917 |
| ARTN | IL1B | 0.038 | -0.011 | 0.087 | 0.130 | 0.917 |
| TSLP | IL1B | -0.037 | -0.085 | 0.012 | 0.139 | 0.917 |
| IL.10RB | IL1B | -0.037 | -0.089 | 0.014 | 0.152 | 0.917 |
| IL.15RA | IL1B | -0.033 | -0.079 | 0.013 | 0.156 | 0.917 |
| CCL23 | IL1B | -0.062 | -0.149 | 0.025 | 0.163 | 0.917 |
| MMP.1 | IL1B | 0.087 | -0.038 | 0.213 | 0.171 | 0.917 |
| CCL3 | IL1B | 0.108 | -0.050 | 0.265 | 0.179 | 0.917 |
| IFN.gamma | IL1B | -0.103 | -0.255 | 0.049 | 0.185 | 0.917 |
| CD40 | IL1B | -0.058 | -0.143 | 0.028 | 0.188 | 0.917 |
| IL7 | IL1B | 0.029 | -0.014 | 0.072 | 0.189 | 0.917 |
| FGF.21 | IL1B | -0.095 | -0.238 | 0.047 | 0.190 | 0.917 |
| uPA | IL1B | -0.044 | -0.116 | 0.029 | 0.239 | 0.917 |
| CCL4 | IL1B | -0.047 | -0.126 | 0.032 | 0.242 | 0.917 |
| IL33 | IL1B | -0.023 | -0.062 | 0.016 | 0.251 | 0.917 |
| IL.10RA | IL1B | 0.016 | -0.013 | 0.044 | 0.283 | 0.917 |
| Beta.NGF | IL1B | -0.008 | -0.022 | 0.007 | 0.289 | 0.917 |
| CCL19 | IL1B | -0.035 | -0.102 | 0.031 | 0.295 | 0.917 |
| TNFB | IL1B | -0.021 | -0.060 | 0.019 | 0.302 | 0.917 |
| DNER | IL1B | -0.027 | -0.078 | 0.024 | 0.305 | 0.917 |
| CDCP1 | IL1B | 0.031 | -0.029 | 0.090 | 0.309 | 0.917 |
| IL.18R1 | IL1B | -0.045 | -0.142 | 0.052 | 0.361 | 0.917 |
| CX3CL1 | IL1B | 0.027 | -0.031 | 0.086 | 0.364 | 0.917 |
| IL.12B | IL1B | -0.036 | -0.116 | 0.044 | 0.372 | 0.917 |
| CD5 | IL1B | -0.048 | -0.155 | 0.059 | 0.379 | 0.917 |
| PD.L1 | IL1B | -0.030 | -0.097 | 0.037 | 0.380 | 0.917 |
| LIF | IL1B | -0.014 | -0.046 | 0.018 | 0.381 | 0.917 |
| CCL11 | IL1B | -0.046 | -0.151 | 0.059 | 0.392 | 0.917 |
| CCL25 | IL1B | -0.030 | -0.100 | 0.040 | 0.404 | 0.917 |
| CD244 | IL1B | -0.033 | -0.112 | 0.046 | 0.413 | 0.917 |
| IL10 | IL1B | -0.019 | -0.064 | 0.026 | 0.416 | 0.917 |
| TWEAK | IL1B | 0.024 | -0.036 | 0.084 | 0.428 | 0.917 |
| FGF.23 | IL1B | -0.021 | -0.075 | 0.034 | 0.450 | 0.917 |
| EN.RAGE | IL1B | -0.029 | -0.108 | 0.050 | 0.465 | 0.917 |
| IL.2RB | IL1B | -0.013 | -0.050 | 0.023 | 0.473 | 0.917 |
| LIF.R | IL1B | -0.009 | -0.036 | 0.017 | 0.485 | 0.917 |
| MCP.3 | IL1B | -0.030 | -0.123 | 0.062 | 0.516 | 0.917 |
| CXCL5 | IL1B | 0.040 | -0.083 | 0.163 | 0.524 | 0.917 |
| IL13 | IL1B | -0.012 | -0.049 | 0.025 | 0.530 | 0.917 |
| STAMBP | IL1B | 0.006 | -0.013 | 0.026 | 0.534 | 0.917 |
| TNFSF14 | IL1B | -0.028 | -0.118 | 0.062 | 0.540 | 0.917 |
| IL.17A | IL1B | 0.014 | -0.033 | 0.062 | 0.550 | 0.917 |
| IL2 | IL1B | -0.014 | -0.059 | 0.032 | 0.554 | 0.917 |
| IL.20RA | IL1B | -0.012 | -0.050 | 0.027 | 0.555 | 0.917 |
| LAP.TGF.beta.1 | IL1B | 0.017 | -0.044 | 0.077 | 0.583 | 0.917 |
| CXCL10 | IL1B | 0.025 | -0.069 | 0.119 | 0.598 | 0.917 |
| SCF | IL1B | -0.017 | -0.082 | 0.048 | 0.602 | 0.917 |
| MCP.1 | IL1B | -0.028 | -0.136 | 0.080 | 0.608 | 0.917 |
| CCL20 | IL1B | -0.027 | -0.135 | 0.081 | 0.623 | 0.917 |
| IL6 | IL1B | -0.029 | -0.145 | 0.087 | 0.624 | 0.917 |
| CXCL6 | IL1B | -0.022 | -0.111 | 0.068 | 0.634 | 0.917 |
| CD6 | IL1B | -0.024 | -0.124 | 0.077 | 0.645 | 0.917 |
| HGF | IL1B | -0.022 | -0.116 | 0.072 | 0.648 | 0.917 |
| NRTN | IL1B | -0.008 | -0.041 | 0.026 | 0.654 | 0.917 |
| Flt3L | IL1B | 0.014 | -0.050 | 0.078 | 0.665 | 0.917 |
| IL4 | IL1B | 0.009 | -0.033 | 0.051 | 0.673 | 0.917 |
| IL.17C | IL1B | 0.023 | -0.086 | 0.133 | 0.675 | 0.917 |
| FGF.19 | IL1B | 0.023 | -0.084 | 0.129 | 0.675 | 0.917 |
| MMP.10 | IL1B | -0.016 | -0.093 | 0.061 | 0.682 | 0.917 |
| AXIN1 | IL1B | -0.016 | -0.095 | 0.064 | 0.701 | 0.917 |
| IL8 | IL1B | -0.021 | -0.133 | 0.090 | 0.709 | 0.917 |
| SIRT2 | IL1B | 0.005 | -0.024 | 0.035 | 0.722 | 0.917 |
| TGF.alpha | IL1B | -0.014 | -0.095 | 0.068 | 0.742 | 0.917 |
| CASP.8 | IL1B | 0.009 | -0.044 | 0.062 | 0.745 | 0.917 |
| OPG | IL1B | -0.010 | -0.069 | 0.050 | 0.748 | 0.917 |
| IL.1.alpha | IL1B | 0.028 | -0.144 | 0.200 | 0.748 | 0.917 |
| ST1A1 | IL1B | -0.011 | -0.079 | 0.058 | 0.756 | 0.917 |
| ADA | IL1B | -0.006 | -0.043 | 0.032 | 0.762 | 0.917 |
| CXCL9 | IL1B | 0.011 | -0.060 | 0.082 | 0.764 | 0.917 |
| NT.3 | IL1B | -0.008 | -0.058 | 0.043 | 0.768 | 0.917 |
| TRANCE | IL1B | -0.010 | -0.083 | 0.062 | 0.780 | 0.920 |
| IL6 | IL6 | 0.015 | -0.101 | 0.131 | 0.798 | 0.927 |
| MMP.1 | IL6 | -0.013 | -0.140 | 0.113 | 0.836 | 0.927 |
| CCL4 | IL6 | 0.008 | -0.071 | 0.088 | 0.838 | 0.927 |
| IL33 | IL6 | 0.004 | -0.036 | 0.043 | 0.847 | 0.927 |
| IFN.gamma | IL6 | 0.013 | -0.140 | 0.167 | 0.866 | 0.927 |
| CSF.1 | IL6 | -0.004 | -0.057 | 0.048 | 0.872 | 0.927 |
| CD5 | IL6 | 0.009 | -0.099 | 0.116 | 0.876 | 0.927 |
| X4E.BP1 | IL6 | 0.002 | -0.024 | 0.028 | 0.888 | 0.927 |
| NRTN | IL6 | 0.002 | -0.031 | 0.036 | 0.890 | 0.927 |
| IL8 | IL6 | 0.007 | -0.104 | 0.119 | 0.896 | 0.927 |
| IL.20RA | IL6 | 0.003 | -0.036 | 0.041 | 0.898 | 0.927 |
| TRAIL | IL6 | -0.004 | -0.075 | 0.066 | 0.907 | 0.927 |
| TGF.alpha | IL17A | -0.010 | -0.088 | 0.067 | 0.797 | 0.928 |
| MCP.2 | CRP | -0.008 | -0.085 | 0.069 | 0.833 | 0.935 |
| IL10 | IL17A | 0.005 | -0.038 | 0.048 | 0.814 | 0.936 |
| MCP.4 | IL1B | 0.013 | -0.095 | 0.120 | 0.814 | 0.943 |
| CD8A | IL1B | -0.010 | -0.100 | 0.079 | 0.820 | 0.943 |
| IL.17A | IL6 | -0.002 | -0.050 | 0.046 | 0.938 | 0.943 |
| MMP.10 | IL6 | -0.003 | -0.080 | 0.074 | 0.943 | 0.943 |
| CXCL11 | IL1B | -0.012 | -0.130 | 0.106 | 0.844 | 0.949 |
| VEGFA | IL1B | 0.008 | -0.075 | 0.092 | 0.845 | 0.949 |
| IL33 | CRP | -0.003 | -0.045 | 0.038 | 0.869 | 0.951 |
| Flt3L | CRP | -0.006 | -0.074 | 0.062 | 0.872 | 0.951 |
| CXCL11 | CRP | -0.010 | -0.135 | 0.116 | 0.879 | 0.951 |
| FGF.21 | CRP | -0.011 | -0.163 | 0.142 | 0.891 | 0.953 |
| IL.12B | IL17A | -0.006 | -0.082 | 0.070 | 0.874 | 0.961 |
| CD40 | IL17A | -0.006 | -0.088 | 0.077 | 0.891 | 0.961 |
| CXCL1 | IL17A | -0.005 | -0.074 | 0.065 | 0.892 | 0.961 |
| IL.15RA | IL17A | -0.003 | -0.047 | 0.041 | 0.896 | 0.961 |
| CCL23 | IL17A | -0.005 | -0.089 | 0.078 | 0.899 | 0.961 |
| CXCL11 | IL17A | -0.007 | -0.120 | 0.106 | 0.905 | 0.961 |
| TWEAK | IL17A | 0.003 | -0.054 | 0.061 | 0.909 | 0.961 |
| IL.15RA | CRP | -0.002 | -0.051 | 0.046 | 0.920 | 0.967 |
| IFN.gamma | CRP | -0.007 | -0.168 | 0.155 | 0.934 | 0.967 |
| ARTN | CRP | 0.002 | -0.051 | 0.055 | 0.945 | 0.967 |
| TSLP | CRP | 0.002 | -0.050 | 0.053 | 0.951 | 0.967 |
| STAMBP | CRP | 0.001 | -0.020 | 0.021 | 0.956 | 0.967 |
| OPG | IL17A | -0.003 | -0.059 | 0.054 | 0.928 | 0.971 |
| MCP.4 | CRP | 0.002 | -0.112 | 0.116 | 0.974 | 0.974 |
| IL18 | IL1B | 0.007 | -0.081 | 0.094 | 0.884 | 0.980 |
| CCL11 | IL17A | -0.003 | -0.103 | 0.098 | 0.958 | 0.982 |
| CCL20 | IL17A | -0.002 | -0.105 | 0.101 | 0.966 | 0.982 |
| IL8 | IL17A | 0.002 | -0.105 | 0.108 | 0.971 | 0.982 |
| TRANCE | IL17A | 0.001 | -0.069 | 0.070 | 0.987 | 0.987 |
| SLAMF1 | IL1B | -0.002 | -0.052 | 0.048 | 0.939 | 0.997 |
| X4E.BP1 | IL1B | 0.001 | -0.025 | 0.027 | 0.941 | 0.997 |
| IL5 | IL1B | 0.002 | -0.048 | 0.051 | 0.946 | 0.997 |
| CXCL1 | IL1B | -0.002 | -0.074 | 0.071 | 0.962 | 0.997 |
| TNF | IL1B | -0.001 | -0.074 | 0.071 | 0.974 | 0.997 |
| CST5 | IL1B | 0.002 | -0.123 | 0.127 | 0.975 | 0.997 |
| IL.22.RA1 | IL1B | 0.000 | -0.074 | 0.075 | 0.990 | 0.997 |
| OSM | IL1B | 0.000 | -0.098 | 0.099 | 0.994 | 0.997 |
| TRAIL | IL1B | 0.000 | -0.070 | 0.071 | 0.997 | 0.997 |

# **Supplementary Table 13.** Regression estimates for associations between continuous maternal inflammation levels measured among the restricted temporal proximity subset (n = 105), further adjusted for maternal age, pre-pregnancy BMI, and race and ethnicity, ordered by adjusted p-value

| **Neonatal  inflammatory marker** | **Maternal Inflammatory Marker** | **Beta** | **95% CI Low** | **95% CI High** | **p-value** | **Adjusted p-value** |
| --- | --- | --- | --- | --- | --- | --- |
| OSM | IL1B | 0.193 | 0.061 | 0.326 | 0.005 | 0.164 |
| FGF.21 | IL1B | 0.348 | 0.109 | 0.587 | 0.005 | 0.164 |
| IL.18R1 | IL1B | 0.231 | 0.070 | 0.392 | 0.005 | 0.164 |
| CST5 | CRP | -0.201 | -0.327 | -0.074 | 0.002 | 0.203 |
| CX3CL1 | IL1B | 0.135 | 0.031 | 0.238 | 0.011 | 0.217 |
| MCP.1 | IL1B | 0.215 | 0.049 | 0.381 | 0.012 | 0.217 |
| MCP.3 | IL1B | 0.209 | 0.029 | 0.389 | 0.023 | 0.316 |
| CXCL1 | IL1B | 0.119 | 0.014 | 0.225 | 0.027 | 0.316 |
| CCL23 | IL1B | 0.165 | 0.018 | 0.311 | 0.028 | 0.316 |
| CASP.8 | IL1B | 0.096 | 0.009 | 0.182 | 0.031 | 0.316 |
| Flt3L | IL17A | -0.133 | -0.223 | -0.042 | 0.004 | 0.404 |
| IL6 | IL1B | 0.189 | 0.002 | 0.376 | 0.047 | 0.426 |
| ST1A1 | IL1B | -0.106 | -0.213 | 0.000 | 0.051 | 0.426 |
| SCF | IL1B | 0.106 | -0.007 | 0.218 | 0.065 | 0.434 |
| CSF.1 | IL1B | 0.081 | -0.006 | 0.169 | 0.067 | 0.434 |
| HGF | IL1B | 0.128 | -0.009 | 0.266 | 0.068 | 0.434 |
| PD.L1 | IL1B | 0.092 | -0.009 | 0.193 | 0.074 | 0.434 |
| IL8 | IL1B | 0.143 | -0.015 | 0.302 | 0.075 | 0.434 |
| IL.17A | IL1B | 0.058 | -0.010 | 0.126 | 0.091 | 0.434 |
| CXCL6 | IL1B | 0.118 | -0.020 | 0.256 | 0.094 | 0.434 |
| AXIN1 | IL1B | 0.098 | -0.017 | 0.214 | 0.095 | 0.434 |
| TRAIL | IL1B | 0.088 | -0.021 | 0.197 | 0.113 | 0.434 |
| uPA | IL1B | 0.097 | -0.024 | 0.219 | 0.115 | 0.434 |
| TGF.alpha | IL1B | 0.083 | -0.021 | 0.187 | 0.117 | 0.434 |
| MMP.10 | IL1B | 0.102 | -0.026 | 0.229 | 0.118 | 0.434 |
| CD40 | IL1B | 0.107 | -0.028 | 0.242 | 0.118 | 0.434 |
| IL18 | IL1B | 0.100 | -0.026 | 0.226 | 0.119 | 0.434 |
| GDNF | IL1B | -0.066 | -0.150 | 0.018 | 0.123 | 0.434 |
| TRANCE | IL1B | -0.096 | -0.221 | 0.029 | 0.131 | 0.447 |
| SLAMF1 | IL1B | 0.054 | -0.018 | 0.126 | 0.141 | 0.458 |
| IFN.gamma | IL1B | 0.139 | -0.048 | 0.326 | 0.144 | 0.458 |
| CCL11 | IL1B | 0.101 | -0.043 | 0.245 | 0.168 | 0.511 |
| CD6 | IL1B | 0.114 | -0.052 | 0.280 | 0.177 | 0.511 |
| NRTN | IL1B | -0.033 | -0.081 | 0.016 | 0.182 | 0.511 |
| IL7 | IL1B | 0.046 | -0.022 | 0.113 | 0.183 | 0.511 |
| IL.10RB | IL1B | 0.060 | -0.031 | 0.150 | 0.193 | 0.523 |
| CD5 | IL1B | 0.117 | -0.066 | 0.299 | 0.208 | 0.523 |
| CCL25 | IL1B | 0.065 | -0.037 | 0.168 | 0.209 | 0.523 |
| LIF.R | IL1B | 0.024 | -0.013 | 0.061 | 0.210 | 0.523 |
| OPG | IL1B | 0.060 | -0.037 | 0.157 | 0.225 | 0.545 |
| LIF | IL1B | 0.034 | -0.027 | 0.095 | 0.268 | 0.632 |
| CXCL9 | IL1B | -0.060 | -0.170 | 0.051 | 0.287 | 0.657 |
| ADA | IL1B | 0.034 | -0.032 | 0.101 | 0.306 | 0.657 |
| IL5 | IL1B | -0.040 | -0.117 | 0.037 | 0.309 | 0.657 |
| CXCL10 | IL1B | 0.074 | -0.072 | 0.220 | 0.317 | 0.657 |
| TNFB | IL1B | -0.038 | -0.114 | 0.037 | 0.318 | 0.657 |
| IL.24 | IL1B | -0.062 | -0.186 | 0.062 | 0.321 | 0.657 |
| IL10 | IL1B | 0.039 | -0.042 | 0.120 | 0.342 | 0.684 |
| FGF.19 | IL1B | 0.077 | -0.086 | 0.240 | 0.349 | 0.684 |
| CCL20 | IL1B | 0.073 | -0.085 | 0.231 | 0.361 | 0.691 |
| CD8A | IL1B | 0.065 | -0.082 | 0.212 | 0.382 | 0.697 |
| CCL4 | IL1B | 0.060 | -0.077 | 0.197 | 0.385 | 0.697 |
| TWEAK | IL1B | 0.038 | -0.049 | 0.126 | 0.387 | 0.697 |
| IL.12B | IL1B | -0.053 | -0.176 | 0.070 | 0.398 | 0.705 |
| NT.3 | IL1B | 0.030 | -0.045 | 0.105 | 0.433 | 0.752 |
| IL.17C | IL17A | -0.166 | -0.319 | -0.013 | 0.034 | 0.755 |
| EN.RAGE | IL17A | 0.111 | 0.005 | 0.218 | 0.040 | 0.755 |
| CXCL11 | IL17A | -0.206 | -0.403 | -0.008 | 0.041 | 0.755 |
| CD8A | IL17A | -0.123 | -0.255 | 0.008 | 0.066 | 0.755 |
| CDCP1 | IL17A | -0.053 | -0.113 | 0.007 | 0.085 | 0.755 |
| CCL25 | IL17A | -0.078 | -0.170 | 0.015 | 0.100 | 0.755 |
| IL.12B | IL17A | -0.091 | -0.201 | 0.020 | 0.106 | 0.755 |
| IL10 | IL17A | 0.059 | -0.013 | 0.132 | 0.108 | 0.755 |
| CCL19 | IL17A | -0.077 | -0.173 | 0.018 | 0.112 | 0.755 |
| NRTN | IL17A | 0.035 | -0.009 | 0.078 | 0.117 | 0.755 |
| AXIN1 | IL17A | -0.080 | -0.185 | 0.025 | 0.134 | 0.755 |
| CXCL10 | IL17A | -0.098 | -0.229 | 0.034 | 0.144 | 0.755 |
| CCL20 | IL17A | -0.104 | -0.246 | 0.038 | 0.148 | 0.755 |
| CD40 | IL17A | -0.089 | -0.212 | 0.034 | 0.153 | 0.755 |
| IL4 | IL17A | -0.058 | -0.138 | 0.022 | 0.155 | 0.755 |
| IL.20 | IL17A | -0.034 | -0.082 | 0.015 | 0.175 | 0.755 |
| FGF.19 | IL17A | -0.097 | -0.243 | 0.050 | 0.194 | 0.755 |
| CXCL5 | IL17A | -0.104 | -0.266 | 0.059 | 0.209 | 0.755 |
| MCP.4 | IL17A | -0.104 | -0.271 | 0.062 | 0.216 | 0.755 |
| FGF.5 | IL17A | -0.050 | -0.130 | 0.030 | 0.220 | 0.755 |
| MCP.1 | IL17A | -0.093 | -0.248 | 0.061 | 0.234 | 0.755 |
| TNFB | IL17A | -0.041 | -0.110 | 0.027 | 0.234 | 0.755 |
| CST5 | IL17A | -0.081 | -0.218 | 0.055 | 0.241 | 0.755 |
| TRANCE | IL17A | -0.066 | -0.180 | 0.048 | 0.250 | 0.755 |
| CD5 | IL17A | -0.096 | -0.262 | 0.070 | 0.253 | 0.755 |
| LIF.R | IL17A | -0.019 | -0.053 | 0.014 | 0.255 | 0.755 |
| ARTN | IL17A | -0.040 | -0.111 | 0.030 | 0.256 | 0.755 |
| CXCL6 | IL17A | 0.070 | -0.057 | 0.197 | 0.274 | 0.755 |
| IL.10RA | IL17A | -0.023 | -0.066 | 0.019 | 0.278 | 0.755 |
| MCP.3 | IL17A | -0.092 | -0.258 | 0.075 | 0.278 | 0.755 |
| MMP.1 | IL17A | -0.109 | -0.307 | 0.090 | 0.280 | 0.755 |
| CD6 | IL17A | -0.081 | -0.232 | 0.071 | 0.294 | 0.755 |
| TNFRSF9 | IL17A | -0.061 | -0.178 | 0.056 | 0.303 | 0.755 |
| CD244 | IL17A | -0.056 | -0.165 | 0.053 | 0.310 | 0.755 |
| uPA | IL17A | 0.057 | -0.054 | 0.168 | 0.313 | 0.755 |
| FGF.23 | IL17A | -0.060 | -0.181 | 0.061 | 0.325 | 0.755 |
| IL.24 | IL17A | -0.054 | -0.166 | 0.059 | 0.344 | 0.755 |
| IL13 | IL17A | -0.024 | -0.075 | 0.027 | 0.344 | 0.755 |
| ST1A1 | IL17A | 0.046 | -0.052 | 0.145 | 0.351 | 0.755 |
| TNFSF14 | IL17A | 0.058 | -0.065 | 0.182 | 0.351 | 0.755 |
| IL.17A | IL17A | 0.029 | -0.033 | 0.091 | 0.359 | 0.755 |
| Beta.NGF | IL17A | 0.011 | -0.013 | 0.036 | 0.365 | 0.755 |
| IL.2RB | IL17A | -0.023 | -0.073 | 0.028 | 0.374 | 0.755 |
| IL7 | IL17A | -0.027 | -0.089 | 0.034 | 0.381 | 0.755 |
| IL8 | IL17A | -0.064 | -0.209 | 0.082 | 0.389 | 0.755 |
| PD.L1 | IL17A | -0.040 | -0.133 | 0.053 | 0.394 | 0.755 |
| CXCL9 | IL17A | -0.043 | -0.144 | 0.057 | 0.394 | 0.755 |
| IL.20RA | IL17A | 0.024 | -0.032 | 0.080 | 0.402 | 0.755 |
| IL.17C | IL1B | 0.066 | -0.106 | 0.238 | 0.448 | 0.763 |
| IL.1.alpha | IL17A | -0.131 | -0.450 | 0.188 | 0.417 | 0.767 |
| NT.3 | IL17A | 0.027 | -0.042 | 0.095 | 0.441 | 0.778 |
| SLAMF1 | IL17A | 0.025 | -0.041 | 0.092 | 0.447 | 0.778 |
| CXCL1 | IL17A | 0.038 | -0.060 | 0.135 | 0.448 | 0.778 |
| CD244 | IL1B | 0.044 | -0.076 | 0.165 | 0.469 | 0.784 |
| CCL20 | CRP | 0.153 | 0.018 | 0.288 | 0.027 | 0.811 |
| IL10 | CRP | 0.078 | 0.009 | 0.148 | 0.027 | 0.811 |
| MMP.10 | CRP | 0.111 | 0.000 | 0.223 | 0.049 | 0.811 |
| SLAMF1 | CRP | 0.061 | -0.002 | 0.124 | 0.058 | 0.811 |
| Flt3L | CRP | -0.086 | -0.176 | 0.003 | 0.058 | 0.811 |
| X4E.BP1 | CRP | 0.038 | -0.002 | 0.078 | 0.062 | 0.811 |
| IL.15RA | IL1B | 0.024 | -0.048 | 0.097 | 0.506 | 0.816 |
| CCL28 | IL1B | -0.015 | -0.059 | 0.030 | 0.514 | 0.816 |
| IL.22.RA1 | IL1B | -0.042 | -0.173 | 0.090 | 0.533 | 0.816 |
| CCL3 | IL1B | 0.065 | -0.143 | 0.273 | 0.538 | 0.816 |
| ARTN | IL1B | -0.024 | -0.102 | 0.054 | 0.540 | 0.816 |
| CDCP1 | IL1B | -0.020 | -0.088 | 0.047 | 0.548 | 0.816 |
| MCP.4 | IL1B | 0.056 | -0.129 | 0.240 | 0.550 | 0.816 |
| IL13 | IL1B | 0.017 | -0.040 | 0.073 | 0.562 | 0.820 |
| IL.22.RA1 | IL17A | 0.042 | -0.078 | 0.161 | 0.487 | 0.826 |
| LAP.TGF.beta.1 | IL17A | -0.032 | -0.125 | 0.061 | 0.494 | 0.826 |
| CCL4 | IL17A | -0.041 | -0.165 | 0.083 | 0.510 | 0.838 |
| CST5 | IL1B | -0.042 | -0.193 | 0.110 | 0.588 | 0.845 |
| IL33 | IL1B | 0.016 | -0.051 | 0.084 | 0.631 | 0.857 |
| STAMBP | IL1B | 0.008 | -0.026 | 0.042 | 0.639 | 0.857 |
| VEGFA | IL1B | 0.030 | -0.099 | 0.159 | 0.646 | 0.857 |
| DNER | IL1B | 0.019 | -0.064 | 0.102 | 0.646 | 0.857 |
| IL.10RA | IL1B | 0.011 | -0.036 | 0.058 | 0.649 | 0.857 |
| Beta.NGF | IL1B | 0.006 | -0.021 | 0.034 | 0.652 | 0.857 |
| Flt3L | IL1B | 0.023 | -0.081 | 0.126 | 0.667 | 0.864 |
| CCL23 | IL17A | 0.040 | -0.095 | 0.176 | 0.556 | 0.864 |
| CCL28 | IL17A | 0.012 | -0.028 | 0.052 | 0.557 | 0.864 |
| IFN.gamma | IL17A | -0.049 | -0.220 | 0.122 | 0.571 | 0.864 |
| TGF.alpha | IL17A | 0.027 | -0.069 | 0.122 | 0.580 | 0.864 |
| DNER | IL17A | -0.021 | -0.096 | 0.055 | 0.586 | 0.864 |
| SIRT2 | IL17A | 0.012 | -0.037 | 0.062 | 0.616 | 0.864 |
| CASP.8 | IL17A | 0.020 | -0.060 | 0.101 | 0.617 | 0.864 |
| IL18 | IL17A | 0.029 | -0.087 | 0.144 | 0.625 | 0.864 |
| LIF | IL17A | -0.014 | -0.069 | 0.042 | 0.628 | 0.864 |
| IL33 | IL17A | -0.015 | -0.076 | 0.047 | 0.635 | 0.864 |
| OSM | IL17A | -0.028 | -0.153 | 0.098 | 0.663 | 0.864 |
| IL.18R1 | IL17A | 0.033 | -0.119 | 0.185 | 0.669 | 0.864 |
| IL6 | IL17A | -0.037 | -0.209 | 0.136 | 0.675 | 0.864 |
| STAMBP | IL17A | 0.006 | -0.024 | 0.037 | 0.679 | 0.864 |
| OPG | IL17A | -0.018 | -0.107 | 0.070 | 0.680 | 0.864 |
| TSLP | IL17A | 0.014 | -0.053 | 0.081 | 0.682 | 0.864 |
| GDNF | IL17A | -0.016 | -0.093 | 0.061 | 0.686 | 0.864 |
| HGF | IL17A | 0.025 | -0.102 | 0.152 | 0.700 | 0.870 |
| CCL11 | IL17A | -0.024 | -0.155 | 0.108 | 0.723 | 0.873 |
| IL.15RA | IL17A | 0.011 | -0.055 | 0.078 | 0.731 | 0.873 |
| SCF | IL17A | -0.017 | -0.121 | 0.087 | 0.742 | 0.873 |
| IL2 | IL17A | -0.010 | -0.071 | 0.051 | 0.749 | 0.873 |
| MCP.2 | IL17A | -0.023 | -0.171 | 0.125 | 0.759 | 0.873 |
| CX3CL1 | IL17A | 0.015 | -0.082 | 0.112 | 0.764 | 0.873 |
| TWEAK | IL17A | 0.012 | -0.068 | 0.091 | 0.774 | 0.873 |
| TNF | IL17A | -0.019 | -0.154 | 0.117 | 0.785 | 0.873 |
| CSF.1 | IL17A | -0.010 | -0.091 | 0.070 | 0.801 | 0.873 |
| IL.10RB | IL17A | -0.010 | -0.093 | 0.072 | 0.805 | 0.873 |
| ADA | IL17A | 0.007 | -0.053 | 0.068 | 0.807 | 0.873 |
| IL.20RA | IL1B | -0.012 | -0.075 | 0.050 | 0.693 | 0.886 |
| X4E.BP1 | IL1B | -0.009 | -0.055 | 0.037 | 0.706 | 0.890 |
| TNFSF14 | IL1B | 0.025 | -0.112 | 0.162 | 0.718 | 0.893 |
| MMP.1 | IL1B | 0.038 | -0.182 | 0.258 | 0.732 | 0.897 |
| EN.RAGE | IL1B | -0.019 | -0.139 | 0.101 | 0.755 | 0.904 |
| LAP.TGF.beta.1 | IL1B | 0.015 | -0.087 | 0.118 | 0.766 | 0.904 |
| TNF | IL1B | 0.022 | -0.128 | 0.172 | 0.771 | 0.904 |
| TNFRSF9 | IL1B | 0.019 | -0.111 | 0.149 | 0.776 | 0.904 |
| FGF.5 | IL1B | -0.011 | -0.101 | 0.078 | 0.799 | 0.919 |
| MMP.10 | IL17A | 0.010 | -0.107 | 0.127 | 0.865 | 0.925 |
| SIRT2 | IL1B | -0.006 | -0.060 | 0.048 | 0.824 | 0.934 |
| IL.2RB | IL1B | -0.006 | -0.061 | 0.050 | 0.841 | 0.934 |
| FGF.23 | IL1B | 0.013 | -0.121 | 0.147 | 0.846 | 0.934 |
| MCP.2 | IL1B | 0.015 | -0.148 | 0.179 | 0.853 | 0.934 |
| CXCL11 | IL1B | 0.019 | -0.204 | 0.241 | 0.869 | 0.940 |
| TRAIL | IL17A | 0.006 | -0.094 | 0.106 | 0.898 | 0.950 |
| VEGFA | IL17A | -0.007 | -0.124 | 0.110 | 0.909 | 0.950 |
| IL5 | IL17A | 0.003 | -0.067 | 0.073 | 0.928 | 0.959 |
| MCP.3 | IL6 | 0.191 | 0.039 | 0.343 | 0.014 | 0.959 |
| IL8 | IL6 | 0.139 | 0.005 | 0.273 | 0.042 | 0.959 |
| IL.17C | IL6 | 0.145 | 0.001 | 0.289 | 0.048 | 0.959 |
| IL.20RA | IL6 | 0.044 | -0.009 | 0.096 | 0.100 | 0.959 |
| SLAMF1 | IL6 | 0.045 | -0.017 | 0.106 | 0.152 | 0.959 |
| DNER | IL6 | -0.049 | -0.119 | 0.021 | 0.166 | 0.959 |
| OSM | IL6 | 0.078 | -0.038 | 0.195 | 0.184 | 0.959 |
| IL18 | IL6 | 0.069 | -0.038 | 0.177 | 0.203 | 0.959 |
| IL.17A | IL6 | 0.037 | -0.021 | 0.095 | 0.213 | 0.959 |
| IL.1.alpha | IL6 | 0.182 | -0.116 | 0.480 | 0.229 | 0.959 |
| TRANCE | IL6 | -0.063 | -0.169 | 0.044 | 0.247 | 0.959 |
| CCL11 | IL6 | 0.071 | -0.052 | 0.193 | 0.256 | 0.959 |
| ADA | IL6 | 0.032 | -0.024 | 0.088 | 0.262 | 0.959 |
| IL7 | IL6 | -0.033 | -0.090 | 0.025 | 0.263 | 0.959 |
| TWEAK | IL6 | -0.041 | -0.115 | 0.034 | 0.279 | 0.959 |
| CSF.1 | IL6 | 0.040 | -0.034 | 0.115 | 0.286 | 0.959 |
| CXCL11 | IL6 | 0.100 | -0.088 | 0.288 | 0.293 | 0.959 |
| MCP.1 | IL6 | 0.076 | -0.069 | 0.221 | 0.299 | 0.959 |
| TNFB | IL6 | -0.034 | -0.098 | 0.031 | 0.303 | 0.959 |
| IL13 | IL6 | -0.023 | -0.071 | 0.025 | 0.344 | 0.959 |
| GDNF | IL6 | 0.033 | -0.039 | 0.105 | 0.371 | 0.959 |
| CXCL1 | IL6 | 0.041 | -0.051 | 0.132 | 0.377 | 0.959 |
| TRAIL | IL6 | 0.038 | -0.055 | 0.132 | 0.416 | 0.959 |
| MMP.1 | IL6 | -0.076 | -0.263 | 0.110 | 0.417 | 0.959 |
| ST1A1 | IL6 | -0.038 | -0.130 | 0.055 | 0.420 | 0.959 |
| FGF.19 | IL6 | -0.055 | -0.194 | 0.083 | 0.428 | 0.959 |
| STAMBP | IL6 | 0.012 | -0.017 | 0.040 | 0.431 | 0.959 |
| CCL20 | IL6 | 0.052 | -0.082 | 0.186 | 0.442 | 0.959 |
| CCL4 | IL6 | 0.045 | -0.071 | 0.161 | 0.444 | 0.959 |
| IL5 | IL6 | -0.025 | -0.091 | 0.040 | 0.449 | 0.959 |
| IL4 | IL6 | 0.029 | -0.047 | 0.105 | 0.449 | 0.959 |
| AXIN1 | IL6 | 0.038 | -0.062 | 0.137 | 0.454 | 0.959 |
| IL.18R1 | IL6 | 0.053 | -0.089 | 0.195 | 0.459 | 0.959 |
| IL.10RA | IL6 | -0.015 | -0.055 | 0.025 | 0.464 | 0.959 |
| CXCL6 | IL6 | 0.044 | -0.075 | 0.163 | 0.468 | 0.959 |
| HGF | IL6 | 0.043 | -0.076 | 0.162 | 0.475 | 0.959 |
| MCP.2 | IL6 | -0.049 | -0.188 | 0.089 | 0.481 | 0.959 |
| TNF | IL6 | -0.045 | -0.172 | 0.082 | 0.482 | 0.959 |
| NRTN | IL6 | 0.014 | -0.027 | 0.056 | 0.487 | 0.959 |
| X4E.BP1 | IL6 | -0.013 | -0.052 | 0.026 | 0.508 | 0.959 |
| CD5 | IL6 | 0.050 | -0.106 | 0.206 | 0.522 | 0.959 |
| Flt3L | IL6 | 0.027 | -0.061 | 0.115 | 0.540 | 0.959 |
| IL33 | IL6 | 0.017 | -0.040 | 0.075 | 0.553 | 0.959 |
| IL.24 | IL6 | 0.031 | -0.074 | 0.137 | 0.559 | 0.959 |
| CD6 | IL6 | 0.040 | -0.103 | 0.182 | 0.583 | 0.959 |
| CCL19 | IL6 | 0.025 | -0.066 | 0.115 | 0.590 | 0.959 |
| IFN.gamma | IL6 | 0.044 | -0.117 | 0.204 | 0.591 | 0.959 |
| CCL3 | IL6 | -0.048 | -0.225 | 0.129 | 0.592 | 0.959 |
| EN.RAGE | IL6 | -0.026 | -0.128 | 0.075 | 0.609 | 0.959 |
| FGF.21 | IL6 | 0.054 | -0.158 | 0.265 | 0.616 | 0.959 |
| LIF | IL6 | 0.012 | -0.039 | 0.064 | 0.637 | 0.959 |
| FGF.5 | IL6 | -0.017 | -0.093 | 0.058 | 0.647 | 0.959 |
| IL.22.RA1 | IL6 | -0.026 | -0.138 | 0.086 | 0.649 | 0.959 |
| VEGFA | IL6 | -0.025 | -0.135 | 0.084 | 0.650 | 0.959 |
| CCL23 | IL6 | 0.028 | -0.099 | 0.156 | 0.658 | 0.959 |
| CX3CL1 | IL6 | 0.020 | -0.071 | 0.111 | 0.664 | 0.959 |
| MCP.4 | IL6 | 0.034 | -0.123 | 0.191 | 0.670 | 0.959 |
| ARTN | IL6 | -0.014 | -0.080 | 0.052 | 0.671 | 0.959 |
| CXCL5 | IL6 | -0.032 | -0.185 | 0.122 | 0.682 | 0.959 |
| OPG | IL6 | -0.016 | -0.099 | 0.067 | 0.700 | 0.959 |
| MMP.10 | IL6 | 0.021 | -0.089 | 0.131 | 0.709 | 0.959 |
| SIRT2 | IL6 | 0.008 | -0.038 | 0.054 | 0.719 | 0.959 |
| SCF | IL6 | 0.018 | -0.080 | 0.115 | 0.720 | 0.959 |
| IL2 | IL6 | 0.010 | -0.047 | 0.068 | 0.721 | 0.959 |
| CD40 | IL6 | 0.020 | -0.096 | 0.136 | 0.737 | 0.959 |
| CDCP1 | IL6 | -0.010 | -0.067 | 0.048 | 0.743 | 0.959 |
| LIF.R | IL6 | 0.005 | -0.027 | 0.037 | 0.763 | 0.959 |
| NT.3 | IL6 | 0.009 | -0.055 | 0.073 | 0.787 | 0.959 |
| CXCL10 | IL6 | 0.017 | -0.108 | 0.141 | 0.790 | 0.959 |
| IL6 | IL6 | 0.021 | -0.141 | 0.183 | 0.794 | 0.959 |
| IL.2RB | IL6 | 0.006 | -0.041 | 0.053 | 0.797 | 0.959 |
| CD244 | IL6 | -0.013 | -0.116 | 0.089 | 0.800 | 0.959 |
| CD8A | IL6 | 0.015 | -0.111 | 0.140 | 0.816 | 0.959 |
| uPA | IL6 | 0.012 | -0.093 | 0.116 | 0.822 | 0.959 |
| IL.15RA | IL6 | 0.007 | -0.055 | 0.069 | 0.825 | 0.959 |
| CCL28 | IL6 | 0.004 | -0.034 | 0.042 | 0.826 | 0.959 |
| IL.20 | IL6 | -0.005 | -0.051 | 0.041 | 0.827 | 0.959 |
| IL10 | IL6 | -0.007 | -0.076 | 0.062 | 0.847 | 0.959 |
| PD.L1 | IL6 | 0.008 | -0.079 | 0.096 | 0.850 | 0.959 |
| IL.12B | IL6 | 0.010 | -0.095 | 0.115 | 0.853 | 0.959 |
| TSLP | IL6 | -0.006 | -0.069 | 0.058 | 0.859 | 0.959 |
| CXCL9 | IL6 | 0.008 | -0.087 | 0.102 | 0.871 | 0.959 |
| LAP.TGF.beta.1 | IL6 | -0.006 | -0.093 | 0.081 | 0.883 | 0.959 |
| CCL25 | IL6 | -0.006 | -0.094 | 0.082 | 0.886 | 0.959 |
| IL.10RB | IL6 | 0.005 | -0.072 | 0.083 | 0.893 | 0.959 |
| CASP.8 | IL6 | -0.004 | -0.080 | 0.071 | 0.907 | 0.959 |
| TNFRSF9 | IL6 | -0.006 | -0.117 | 0.104 | 0.909 | 0.959 |
| CST5 | IL6 | 0.007 | -0.122 | 0.136 | 0.917 | 0.959 |
| IL2 | IL1B | -0.004 | -0.071 | 0.064 | 0.914 | 0.967 |
| IL.20 | IL1B | -0.003 | -0.057 | 0.052 | 0.924 | 0.967 |
| CCL19 | IL1B | 0.005 | -0.102 | 0.111 | 0.931 | 0.967 |
| IL4 | IL1B | -0.004 | -0.093 | 0.086 | 0.936 | 0.967 |
| FGF.21 | IL17A | -0.007 | -0.234 | 0.219 | 0.948 | 0.969 |
| CX3CL1 | CRP | -0.077 | -0.170 | 0.015 | 0.100 | 0.974 |
| IL4 | CRP | -0.064 | -0.141 | 0.014 | 0.107 | 0.974 |
| TGF.alpha | CRP | 0.073 | -0.018 | 0.164 | 0.115 | 0.974 |
| MCP.3 | CRP | 0.128 | -0.032 | 0.287 | 0.116 | 0.974 |
| IL.1.alpha | IL1B | -0.008 | -0.362 | 0.345 | 0.963 | 0.975 |
| TSLP | IL1B | -0.002 | -0.076 | 0.073 | 0.967 | 0.975 |
| CXCL5 | IL1B | 0.003 | -0.178 | 0.184 | 0.975 | 0.975 |
| CCL3 | IL17A | -0.004 | -0.193 | 0.185 | 0.968 | 0.978 |
| CXCL5 | CRP | 0.107 | -0.050 | 0.264 | 0.179 | 0.986 |
| IL8 | CRP | 0.092 | -0.048 | 0.232 | 0.196 | 0.986 |
| IL18 | CRP | -0.072 | -0.183 | 0.039 | 0.200 | 0.986 |
| CD5 | CRP | -0.104 | -0.264 | 0.056 | 0.200 | 0.986 |
| CD8A | CRP | -0.081 | -0.210 | 0.047 | 0.212 | 0.986 |
| STAMBP | CRP | -0.019 | -0.048 | 0.011 | 0.216 | 0.986 |
| CDCP1 | CRP | -0.036 | -0.095 | 0.023 | 0.228 | 0.986 |
| CD6 | CRP | -0.085 | -0.232 | 0.061 | 0.249 | 0.986 |
| TWEAK | CRP | -0.041 | -0.118 | 0.036 | 0.293 | 0.986 |
| AXIN1 | CRP | -0.054 | -0.156 | 0.048 | 0.294 | 0.986 |
| uPA | CRP | -0.055 | -0.163 | 0.052 | 0.309 | 0.986 |
| IL.17A | CRP | -0.031 | -0.091 | 0.029 | 0.310 | 0.986 |
| TSLP | CRP | -0.033 | -0.098 | 0.032 | 0.311 | 0.986 |
| CXCL11 | CRP | -0.094 | -0.288 | 0.100 | 0.339 | 0.986 |
| FGF.21 | CRP | 0.104 | -0.114 | 0.321 | 0.346 | 0.986 |
| HGF | CRP | -0.058 | -0.180 | 0.065 | 0.352 | 0.986 |
| IL.10RB | CRP | 0.036 | -0.043 | 0.116 | 0.367 | 0.986 |
| ARTN | CRP | 0.031 | -0.037 | 0.099 | 0.369 | 0.986 |
| EN.RAGE | CRP | 0.047 | -0.057 | 0.152 | 0.372 | 0.986 |
| CD244 | CRP | -0.046 | -0.152 | 0.059 | 0.384 | 0.986 |
| IL.17C | CRP | 0.065 | -0.086 | 0.216 | 0.396 | 0.986 |
| OPG | CRP | 0.036 | -0.049 | 0.122 | 0.399 | 0.986 |
| TNFSF14 | CRP | -0.051 | -0.171 | 0.069 | 0.400 | 0.986 |
| SIRT2 | CRP | -0.020 | -0.067 | 0.027 | 0.403 | 0.986 |
| CCL11 | CRP | -0.051 | -0.179 | 0.076 | 0.423 | 0.986 |
| MMP.1 | CRP | -0.078 | -0.270 | 0.114 | 0.423 | 0.986 |
| NT.3 | CRP | 0.027 | -0.039 | 0.092 | 0.424 | 0.986 |
| CCL19 | CRP | 0.035 | -0.058 | 0.128 | 0.457 | 0.986 |
| FGF.19 | CRP | -0.050 | -0.192 | 0.093 | 0.491 | 0.986 |
| TRANCE | CRP | -0.038 | -0.149 | 0.072 | 0.495 | 0.986 |
| ADA | CRP | -0.018 | -0.077 | 0.040 | 0.536 | 0.986 |
| TRAIL | CRP | -0.028 | -0.125 | 0.068 | 0.562 | 0.986 |
| IL2 | CRP | -0.017 | -0.076 | 0.042 | 0.574 | 0.986 |
| VEGFA | CRP | -0.031 | -0.144 | 0.082 | 0.585 | 0.986 |
| IL.18R1 | CRP | -0.040 | -0.187 | 0.107 | 0.590 | 0.986 |
| ST1A1 | CRP | -0.023 | -0.118 | 0.072 | 0.632 | 0.986 |
| IL33 | CRP | 0.014 | -0.045 | 0.073 | 0.641 | 0.986 |
| MCP.4 | CRP | -0.037 | -0.199 | 0.125 | 0.650 | 0.986 |
| IL.1.alpha | CRP | 0.070 | -0.239 | 0.379 | 0.654 | 0.986 |
| CCL4 | CRP | 0.027 | -0.093 | 0.147 | 0.657 | 0.986 |
| IL.20RA | CRP | 0.012 | -0.042 | 0.067 | 0.659 | 0.986 |
| TNFRSF9 | CRP | -0.025 | -0.139 | 0.089 | 0.663 | 0.986 |
| LIF.R | CRP | -0.007 | -0.040 | 0.026 | 0.667 | 0.986 |
| CCL28 | CRP | -0.008 | -0.047 | 0.031 | 0.669 | 0.986 |
| FGF.23 | CRP | 0.025 | -0.092 | 0.143 | 0.670 | 0.986 |
| CASP.8 | CRP | -0.017 | -0.094 | 0.061 | 0.672 | 0.986 |
| IL.20 | CRP | -0.009 | -0.057 | 0.038 | 0.701 | 0.986 |
| IL.15RA | CRP | -0.012 | -0.076 | 0.052 | 0.707 | 0.986 |
| CXCL1 | CRP | -0.018 | -0.112 | 0.077 | 0.712 | 0.986 |
| IL7 | CRP | -0.010 | -0.070 | 0.050 | 0.746 | 0.986 |
| TNFB | CRP | 0.011 | -0.056 | 0.077 | 0.753 | 0.986 |
| CXCL9 | CRP | -0.015 | -0.113 | 0.082 | 0.757 | 0.986 |
| IL5 | CRP | 0.009 | -0.059 | 0.077 | 0.787 | 0.986 |
| GDNF | CRP | 0.010 | -0.064 | 0.085 | 0.788 | 0.986 |
| MCP.1 | CRP | 0.020 | -0.130 | 0.171 | 0.789 | 0.986 |
| Beta.NGF | CRP | 0.003 | -0.021 | 0.027 | 0.790 | 0.986 |
| CXCL6 | CRP | -0.015 | -0.138 | 0.108 | 0.808 | 0.986 |
| IL.24 | CRP | 0.012 | -0.097 | 0.121 | 0.825 | 0.986 |
| IL.10RA | CRP | -0.004 | -0.046 | 0.037 | 0.831 | 0.986 |
| LIF | CRP | 0.006 | -0.048 | 0.059 | 0.831 | 0.986 |
| CCL3 | CRP | -0.019 | -0.202 | 0.164 | 0.834 | 0.986 |
| PD.L1 | CRP | -0.009 | -0.100 | 0.081 | 0.837 | 0.986 |
| CD40 | CRP | -0.012 | -0.132 | 0.107 | 0.838 | 0.986 |
| IFN.gamma | CRP | -0.017 | -0.183 | 0.149 | 0.841 | 0.986 |
| FGF.5 | CRP | -0.008 | -0.086 | 0.070 | 0.843 | 0.986 |
| TNF | CRP | 0.013 | -0.118 | 0.144 | 0.846 | 0.986 |
| LAP.TGF.beta.1 | CRP | -0.008 | -0.097 | 0.082 | 0.865 | 0.986 |
| DNER | CRP | 0.006 | -0.067 | 0.079 | 0.866 | 0.986 |
| OSM | CRP | 0.009 | -0.112 | 0.130 | 0.884 | 0.986 |
| IL.2RB | CRP | -0.003 | -0.052 | 0.045 | 0.889 | 0.986 |
| CCL23 | CRP | 0.009 | -0.123 | 0.140 | 0.897 | 0.986 |
| IL.22.RA1 | CRP | 0.006 | -0.110 | 0.122 | 0.917 | 0.986 |
| CCL25 | CRP | 0.005 | -0.086 | 0.096 | 0.920 | 0.986 |
| SCF | CRP | 0.005 | -0.096 | 0.105 | 0.927 | 0.986 |
| IL.12B | CRP | -0.004 | -0.112 | 0.104 | 0.942 | 0.986 |
| MCP.2 | CRP | -0.004 | -0.148 | 0.139 | 0.951 | 0.986 |
| IL6 | CRP | 0.004 | -0.163 | 0.172 | 0.958 | 0.986 |
| IL13 | CRP | -0.001 | -0.051 | 0.048 | 0.958 | 0.986 |
| CXCL10 | CRP | -0.003 | -0.131 | 0.126 | 0.967 | 0.986 |
| CSF.1 | CRP | -0.001 | -0.079 | 0.077 | 0.976 | 0.986 |
| NRTN | CRP | 0.000 | -0.043 | 0.042 | 0.989 | 0.989 |
| FGF.23 | IL6 | 0.002 | -0.112 | 0.116 | 0.967 | 0.989 |
| TGF.alpha | IL6 | -0.001 | -0.090 | 0.089 | 0.985 | 0.989 |
| Beta.NGF | IL6 | 0.000 | -0.023 | 0.023 | 0.986 | 0.989 |
| TNFSF14 | IL6 | -0.001 | -0.117 | 0.116 | 0.989 | 0.989 |
| X4E.BP1 | IL17A | 0.000 | -0.042 | 0.042 | 0.999 | 0.999 |


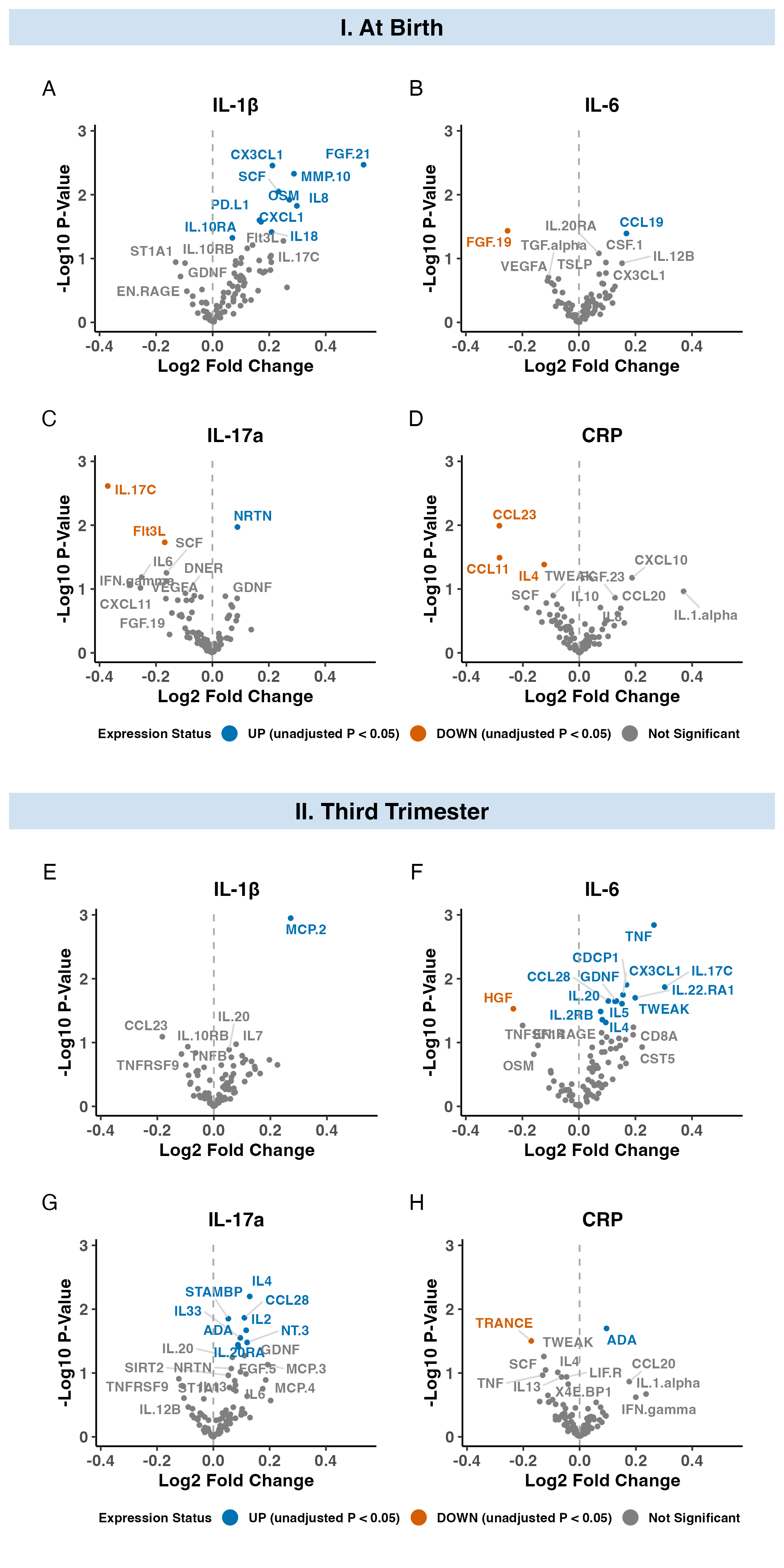


# **Supplementary Figure 5.** Volcano plots for differential expression of neonatal inflammatory markers relative to maternal inflammation levels at birth (I) and during third trimester (II) comparing high vs. low maternal inflammation groups for each maternal marker: IL-1β (A, E), IL-6 (B, F), IL-17a (C, G), and CRP (D, H).

Panels A–D correspond to the primary analysis of maternal inflammation measured at birth (n = 194); panels E–H correspond to the secondary analysis of maternal inflammation measured in the third trimester (n = 235). Each point corresponds to one neonatal inflammatory marker. Neonatal inflammatory markers are colored by statistical significance and direction of differential expression.


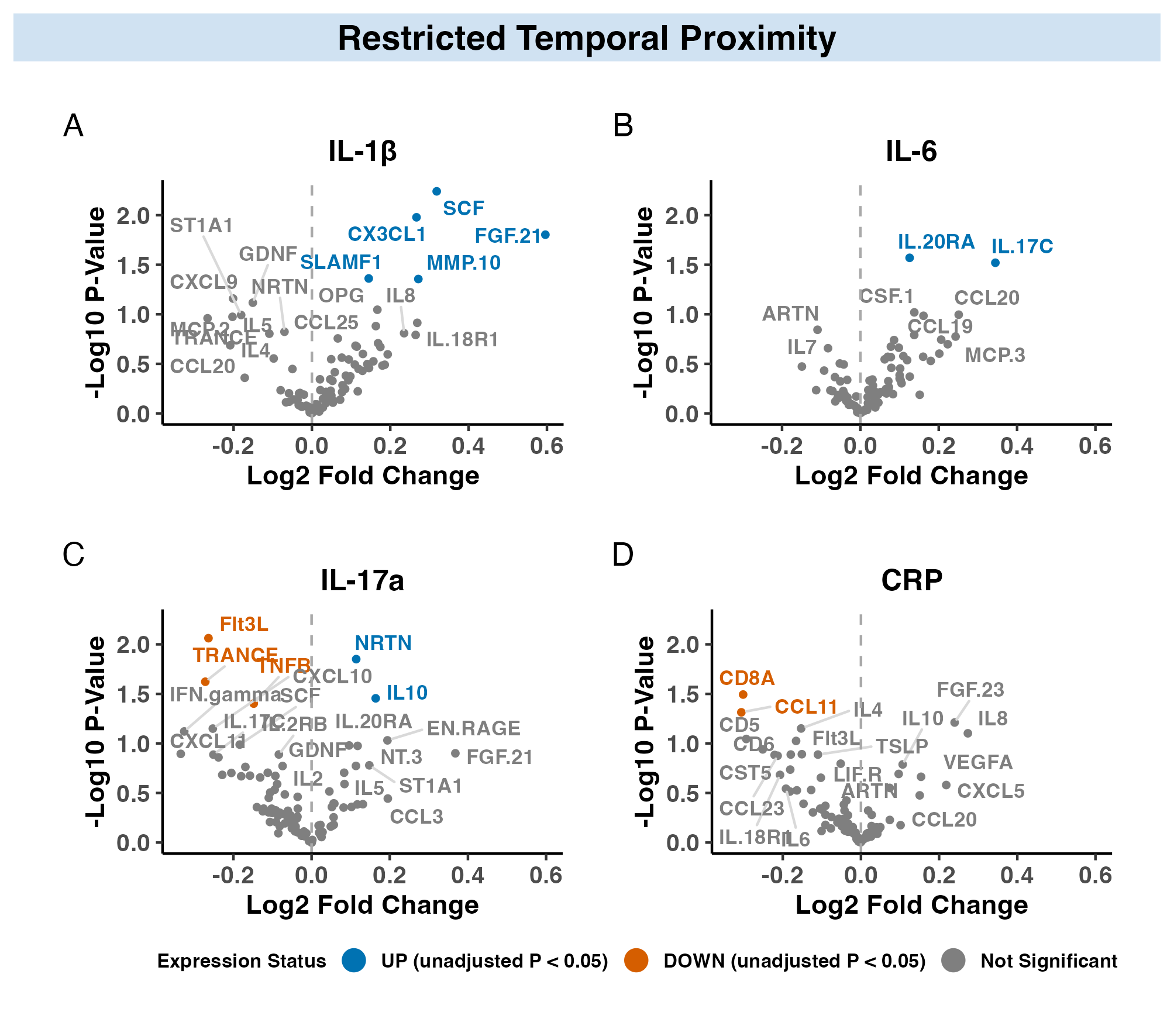


# **Supplementary Figure 6**. Volcano plots for differential expression of neonatal inflammatory markers relative to maternal inflammation levels at birth in first sensitivity analysis comparing high vs. low maternal inflammation groups for each maternal marker: IL-1β (A), IL-6 (B), IL-17a (C), and CRP (D) in a subset of n=105 with maternal inflammatory marker and neonatal DBS collected with restricted temporal proximity.

Each point corresponds to one neonatal inflammatory marker. Markers are colored by statistical significance and direction of differential expression.

# **Supplementary Table 14.** Differential expression results for all neonatal inflammatory markers between high vs. low maternal inflammation at birth (primary analysis; n=194), ordered by adjusted p-value

| **Neonatal  inflammatory marker** | **High Maternal Inflammatory Marker** | **logFC** | **t-statistic** | | **p-value** | **Adjusted p-value** | **Cohen's d** |
| --- | --- | --- | --- | --- | --- | --- | --- |
| FGF.21 | IL1B | 0.534 | 2.967 | 0.003 | | 0.144 | 0.413 |
| CX3CL1 | IL1B | 0.211 | 2.957 | 0.003 | | 0.144 | 0.585 |
| MMP.10 | IL1B | 0.288 | 2.861 | 0.005 | | 0.144 | 0.506 |
| SCF | IL1B | 0.232 | 2.643 | 0.009 | | 0.205 | 0.372 |
| OSM | IL1B | 0.271 | 2.537 | 0.012 | | 0.221 | 0.453 |
| IL.17C | IL17A | -0.371 | -3.073 | 0.002 | | 0.224 | -0.379 |
| IL8 | IL1B | 0.298 | 2.456 | 0.015 | | 0.229 | 0.402 |
| PD.L1 | IL1B | 0.166 | 2.258 | 0.025 | | 0.306 | 0.406 |
| CXCL1 | IL1B | 0.171 | 2.235 | 0.027 | | 0.306 | 0.325 |
| IL18 | IL1B | 0.209 | 2.084 | 0.038 | | 0.394 | 0.301 |
| IL.10RA | IL1B | 0.070 | 1.995 | 0.047 | | 0.436 | 0.338 |
| IL.17C | IL1B | 0.250 | 1.947 | 0.053 | | 0.443 | 0.467 |
| Flt3L | IL1B | 0.141 | 1.881 | 0.062 | | 0.472 | 0.353 |
| IL.10RB | IL1B | 0.122 | 1.828 | 0.069 | | 0.490 | 0.295 |
| NRTN | IL17A | 0.089 | 2.578 | 0.011 | | 0.492 | 0.386 |
| CD6 | IL1B | 0.206 | 1.704 | 0.090 | | 0.496 | 0.244 |
| IL.18R1 | IL1B | 0.204 | 1.677 | 0.095 | | 0.496 | 0.280 |
| IL10 | IL1B | 0.102 | 1.664 | 0.098 | | 0.496 | 0.163 |
| HGF | IL1B | 0.165 | 1.619 | 0.107 | | 0.496 | 0.231 |
| IL7 | IL1B | 0.081 | 1.609 | 0.109 | | 0.496 | 0.320 |
| ST1A1 | IL1B | -0.131 | -1.588 | 0.114 | | 0.496 | -0.165 |
| MCP.3 | IL1B | 0.207 | 1.587 | 0.114 | | 0.496 | 0.419 |
| GDNF | IL1B | -0.098 | -1.571 | 0.118 | | 0.496 | -0.214 |
| CSF.1 | IL1B | 0.099 | 1.568 | 0.119 | | 0.496 | 0.222 |
| IL.17A | IL1B | 0.080 | 1.538 | 0.126 | | 0.503 | 0.182 |
| TNFRSF9 | IL1B | 0.129 | 1.444 | 0.150 | | 0.540 | 0.339 |
| IL6 | IL1B | 0.204 | 1.439 | 0.152 | | 0.540 | 0.201 |
| CCL23 | IL1B | 0.167 | 1.422 | 0.157 | | 0.540 | 0.357 |
| CD5 | IL1B | 0.185 | 1.413 | 0.159 | | 0.540 | 0.172 |
| CST5 | IL1B | 0.178 | 1.387 | 0.167 | | 0.540 | -0.049 |
| TSLP | IL1B | 0.076 | 1.376 | 0.170 | | 0.540 | 0.269 |
| OPG | IL1B | 0.099 | 1.330 | 0.185 | | 0.544 | 0.204 |
| EN.RAGE | IL1B | -0.114 | -1.312 | 0.191 | | 0.544 | -0.091 |
| FGF.5 | IL1B | 0.084 | 1.304 | 0.194 | | 0.544 | 0.265 |
| TRAIL | IL1B | 0.105 | 1.300 | 0.195 | | 0.544 | 0.254 |
| Flt3L | IL17A | -0.169 | -2.375 | 0.019 | | 0.569 | -0.339 |
| uPA | IL1B | 0.109 | 1.257 | 0.210 | | 0.569 | 0.236 |
| CCL25 | IL1B | 0.097 | 1.230 | 0.220 | | 0.578 | -0.007 |
| CD40 | IL1B | 0.113 | 1.176 | 0.241 | | 0.612 | 0.165 |
| IL.15RA | IL1B | 0.060 | 1.164 | 0.246 | | 0.612 | 0.106 |
| CCL4 | IL1B | 0.109 | 1.115 | 0.266 | | 0.634 | 0.277 |
| DNER | IL1B | 0.070 | 1.104 | 0.271 | | 0.634 | 0.173 |
| AXIN1 | IL1B | 0.089 | 1.093 | 0.276 | | 0.634 | 0.169 |
| IL.1.alpha | IL1B | 0.263 | 1.077 | 0.283 | | 0.634 | 0.397 |
| NRTN | IL1B | -0.038 | -1.026 | 0.306 | | 0.657 | -0.184 |
| TGF.alpha | IL1B | 0.090 | 1.024 | 0.307 | | 0.657 | 0.108 |
| CXCL11 | IL1B | 0.161 | 0.999 | 0.319 | | 0.664 | 0.122 |
| TRANCE | IL1B | -0.091 | -0.987 | 0.325 | | 0.664 | -0.115 |
| IL.20 | IL1B | 0.039 | 0.954 | 0.341 | | 0.668 | 0.217 |
| SLAMF1 | IL1B | 0.056 | 0.954 | 0.341 | | 0.668 | 0.211 |
| CCL19 | IL1B | 0.080 | 0.923 | 0.357 | | 0.684 | 0.206 |
| CCL20 | IL1B | 0.107 | 0.873 | 0.384 | | 0.716 | 0.044 |
| CXCL9 | IL1B | -0.070 | -0.864 | 0.389 | | 0.716 | -0.181 |
| IL.24 | IL1B | 0.073 | 0.838 | 0.403 | | 0.719 | 0.064 |
| ADA | IL1B | 0.038 | 0.832 | 0.407 | | 0.719 | 0.044 |
| VEGFA | IL1B | 0.074 | 0.767 | 0.444 | | 0.762 | 0.074 |
| IFN.gamma | IL1B | 0.136 | 0.762 | 0.447 | | 0.762 | 0.020 |
| MCP.1 | IL1B | 0.114 | 0.744 | 0.458 | | 0.762 | 0.276 |
| CXCL6 | IL1B | 0.073 | 0.718 | 0.474 | | 0.762 | 0.069 |
| IL33 | IL1B | 0.035 | 0.699 | 0.485 | | 0.762 | 0.123 |
| CCL28 | IL1B | -0.025 | -0.697 | 0.487 | | 0.762 | -0.150 |
| IL5 | IL1B | -0.038 | -0.694 | 0.489 | | 0.762 | -0.188 |
| X4E.BP1 | IL1B | -0.021 | -0.667 | 0.505 | | 0.775 | -0.134 |
| MCP.2 | IL1B | -0.071 | -0.641 | 0.522 | | 0.787 | -0.126 |
| STAMBP | IL1B | 0.014 | 0.602 | 0.548 | | 0.790 | 0.037 |
| MMP.1 | IL1B | 0.099 | 0.602 | 0.548 | | 0.790 | 0.068 |
| CD244 | IL1B | 0.056 | 0.600 | 0.549 | | 0.790 | 0.014 |
| IL.2RB | IL1B | 0.025 | 0.573 | 0.567 | | 0.803 | 0.158 |
| SCF | IL17A | -0.163 | -1.925 | 0.056 | | 0.814 | -0.342 |
| IL6 | IL17A | -0.250 | -1.857 | 0.065 | | 0.814 | -0.323 |
| VEGFA | IL17A | -0.163 | -1.791 | 0.075 | | 0.814 | -0.311 |
| IFN.gamma | IL17A | -0.294 | -1.743 | 0.083 | | 0.814 | -0.249 |
| CXCL11 | IL17A | -0.255 | -1.672 | 0.096 | | 0.814 | -0.290 |
| DNER | IL17A | -0.096 | -1.578 | 0.116 | | 0.814 | -0.222 |
| IL.2RB | IL17A | -0.064 | -1.531 | 0.127 | | 0.814 | -0.210 |
| X4E.BP1 | IL17A | 0.046 | 1.522 | 0.130 | | 0.814 | 0.256 |
| LIF.R | IL17A | -0.041 | -1.509 | 0.133 | | 0.814 | -0.267 |
| GDNF | IL17A | 0.088 | 1.481 | 0.140 | | 0.814 | 0.256 |
| CCL23 | IL17A | -0.165 | -1.477 | 0.141 | | 0.814 | -0.176 |
| TNFB | IL17A | -0.074 | -1.449 | 0.149 | | 0.814 | -0.277 |
| TNFRSF9 | IL17A | -0.123 | -1.446 | 0.150 | | 0.814 | -0.174 |
| IL.10RB | IL17A | -0.092 | -1.444 | 0.150 | | 0.814 | -0.229 |
| LIF.R | IL1B | 0.013 | 0.459 | 0.647 | | 0.901 | 0.064 |
| IL.15RA | IL17A | 0.066 | 1.354 | 0.177 | | 0.907 | 0.155 |
| LAP.TGF.beta.1 | IL1B | -0.032 | -0.439 | 0.661 | | 0.908 | -0.075 |
| IL.12B | IL1B | 0.043 | 0.413 | 0.680 | | 0.910 | 0.288 |
| TNFB | IL1B | -0.022 | -0.405 | 0.686 | | 0.910 | -0.156 |
| TNF | IL1B | 0.035 | 0.392 | 0.695 | | 0.910 | -0.012 |
| MCP.4 | IL1B | -0.052 | -0.364 | 0.716 | | 0.910 | 0.028 |
| IL2 | IL1B | 0.020 | 0.364 | 0.717 | | 0.910 | 0.089 |
| CCL3 | IL1B | 0.074 | 0.341 | 0.733 | | 0.910 | 0.006 |
| ARTN | IL1B | 0.020 | 0.333 | 0.740 | | 0.910 | 0.083 |
| LIF | IL1B | -0.014 | -0.311 | 0.756 | | 0.910 | -0.011 |
| IL13 | IL1B | -0.013 | -0.309 | 0.758 | | 0.910 | 0.044 |
| CDCP1 | IL1B | 0.018 | 0.304 | 0.761 | | 0.910 | -0.012 |
| NT.3 | IL1B | 0.016 | 0.275 | 0.784 | | 0.915 | -0.017 |
| IL4 | IL1B | 0.018 | 0.273 | 0.785 | | 0.915 | -0.029 |
| NT.3 | IL17A | 0.071 | 1.312 | 0.191 | | 0.919 | 0.175 |
| CSF.1 | IL17A | -0.073 | -1.198 | 0.232 | | 0.919 | -0.185 |
| FGF.19 | IL17A | -0.144 | -1.186 | 0.237 | | 0.919 | -0.225 |
| IL18 | IL17A | -0.110 | -1.148 | 0.252 | | 0.919 | -0.208 |
| IL5 | IL17A | 0.060 | 1.134 | 0.258 | | 0.919 | 0.129 |
| OSM | IL17A | -0.117 | -1.130 | 0.260 | | 0.919 | -0.165 |
| ST1A1 | IL17A | 0.088 | 1.122 | 0.263 | | 0.919 | 0.206 |
| IL.12B | IL17A | -0.108 | -1.104 | 0.271 | | 0.919 | -0.058 |
| IL2 | IL17A | 0.057 | 1.103 | 0.271 | | 0.919 | 0.200 |
| IL.17A | IL17A | 0.053 | 1.067 | 0.287 | | 0.919 | 0.129 |
| CXCL9 | IL17A | -0.082 | -1.062 | 0.290 | | 0.919 | -0.196 |
| CD8A | IL1B | 0.025 | 0.238 | 0.812 | | 0.924 | 0.012 |
| SIRT2 | IL1B | -0.009 | -0.236 | 0.813 | | 0.924 | -0.215 |
| TNFSF14 | IL1B | 0.022 | 0.209 | 0.835 | | 0.925 | 0.098 |
| FGF.19 | IL1B | -0.026 | -0.204 | 0.839 | | 0.925 | -0.064 |
| CCL11 | IL1B | 0.028 | 0.196 | 0.845 | | 0.925 | 0.161 |
| FGF.23 | IL1B | -0.016 | -0.180 | 0.857 | | 0.928 | -0.020 |
| Beta.NGF | IL1B | 0.003 | 0.160 | 0.873 | | 0.934 | -0.028 |
| CCL23 | CRP | -0.283 | -2.596 | 0.010 | | 0.937 | -0.440 |
| IL.24 | IL17A | 0.084 | 1.012 | 0.313 | | 0.960 | 0.125 |
| IL.20RA | IL1B | -0.005 | -0.108 | 0.914 | | 0.961 | -0.160 |
| IL.22.RA1 | IL1B | -0.010 | -0.102 | 0.919 | | 0.961 | 0.017 |
| CASP.8 | IL1B | 0.005 | 0.082 | 0.935 | | 0.962 | 0.027 |
| TWEAK | IL1B | -0.005 | -0.074 | 0.941 | | 0.962 | 0.163 |
| CXCL5 | IL1B | 0.005 | 0.035 | 0.972 | | 0.983 | 0.030 |
| CCL11 | CRP | -0.283 | -2.156 | 0.032 | | 0.983 | -0.380 |
| IL4 | CRP | -0.124 | -2.054 | 0.041 | | 0.983 | -0.315 |
| CXCL10 | CRP | 0.187 | 1.845 | 0.067 | | 0.983 | 0.306 |
| IL.1.alpha | CRP | 0.370 | 1.612 | 0.109 | | 0.983 | 0.208 |
| TWEAK | CRP | -0.092 | -1.536 | 0.126 | | 0.983 | -0.282 |
| FGF.23 | CRP | 0.127 | 1.495 | 0.136 | | 0.983 | 0.248 |
| SCF | CRP | -0.117 | -1.391 | 0.166 | | 0.983 | -0.228 |
| GDNF | CRP | -0.079 | -1.366 | 0.174 | | 0.983 | -0.225 |
| IL10 | CRP | 0.075 | 1.300 | 0.195 | | 0.983 | 0.223 |
| MCP.1 | CRP | -0.187 | -1.294 | 0.197 | | 0.983 | -0.239 |
| CCL20 | CRP | 0.147 | 1.284 | 0.201 | | 0.983 | 0.236 |
| IL.17A | CRP | -0.063 | -1.271 | 0.205 | | 0.983 | -0.189 |
| FGF.19 | CRP | -0.143 | -1.198 | 0.232 | | 0.983 | -0.185 |
| IL8 | CRP | 0.135 | 1.169 | 0.244 | | 0.983 | 0.183 |
| IL.22.RA1 | CRP | -0.104 | -1.150 | 0.252 | | 0.983 | -0.198 |
| LAP.TGF.beta.1 | CRP | -0.077 | -1.139 | 0.256 | | 0.983 | -0.189 |
| IL7 | CRP | -0.053 | -1.101 | 0.272 | | 0.983 | -0.201 |
| TNFRSF9 | CRP | -0.086 | -1.024 | 0.307 | | 0.983 | -0.181 |
| IL.12B | CRP | 0.099 | 1.023 | 0.307 | | 0.983 | 0.126 |
| LIF.R | CRP | -0.027 | -1.001 | 0.318 | | 0.983 | -0.170 |
| CD8A | CRP | -0.097 | -0.999 | 0.319 | | 0.983 | -0.154 |
| HGF | CRP | -0.096 | -0.996 | 0.321 | | 0.983 | -0.144 |
| MCP.4 | CRP | -0.133 | -0.979 | 0.329 | | 0.983 | -0.179 |
| OPG | CRP | -0.068 | -0.970 | 0.333 | | 0.983 | -0.165 |
| IFN.gamma | CRP | 0.160 | 0.957 | 0.340 | | 0.983 | 0.164 |
| IL18 | CRP | 0.090 | 0.941 | 0.348 | | 0.983 | 0.167 |
| STAMBP | CRP | 0.020 | 0.929 | 0.354 | | 0.983 | 0.173 |
| TRANCE | CRP | -0.079 | -0.903 | 0.368 | | 0.983 | -0.144 |
| CXCL5 | CRP | 0.124 | 0.889 | 0.375 | | 0.983 | 0.133 |
| LIF | CRP | -0.036 | -0.866 | 0.388 | | 0.983 | -0.142 |
| TSLP | CRP | -0.045 | -0.863 | 0.389 | | 0.983 | -0.167 |
| FGF.5 | CRP | -0.051 | -0.840 | 0.402 | | 0.983 | -0.153 |
| EN.RAGE | CRP | -0.066 | -0.815 | 0.416 | | 0.983 | -0.147 |
| IL.20 | CRP | -0.030 | -0.799 | 0.426 | | 0.983 | -0.141 |
| ARTN | CRP | 0.045 | 0.797 | 0.427 | | 0.983 | 0.129 |
| MCP.2 | CRP | 0.082 | 0.792 | 0.430 | | 0.983 | 0.141 |
| DNER | CRP | -0.046 | -0.774 | 0.440 | | 0.983 | -0.146 |
| CASP.8 | CRP | 0.044 | 0.764 | 0.446 | | 0.983 | 0.137 |
| IL.20RA | CRP | -0.028 | -0.686 | 0.493 | | 0.983 | -0.074 |
| IL.2RB | CRP | 0.026 | 0.635 | 0.526 | | 0.983 | 0.100 |
| FGF.21 | CRP | 0.102 | 0.597 | 0.551 | | 0.983 | 0.114 |
| TGF.alpha | CRP | 0.048 | 0.581 | 0.562 | | 0.983 | 0.119 |
| CXCL9 | CRP | 0.044 | 0.578 | 0.564 | | 0.983 | 0.114 |
| IL.17C | CRP | 0.070 | 0.578 | 0.564 | | 0.983 | 0.055 |
| MCP.3 | CRP | -0.070 | -0.568 | 0.571 | | 0.983 | -0.123 |
| MMP.10 | CRP | 0.054 | 0.562 | 0.575 | | 0.983 | 0.067 |
| CDCP1 | CRP | 0.031 | 0.553 | 0.581 | | 0.983 | 0.110 |
| TNF | CRP | -0.045 | -0.547 | 0.585 | | 0.983 | -0.066 |
| CCL28 | CRP | -0.018 | -0.536 | 0.593 | | 0.983 | -0.078 |
| IL5 | CRP | -0.027 | -0.520 | 0.603 | | 0.983 | -0.057 |
| IL.15RA | CRP | 0.023 | 0.476 | 0.634 | | 0.983 | 0.098 |
| Flt3L | CRP | -0.032 | -0.454 | 0.650 | | 0.983 | -0.091 |
| CX3CL1 | CRP | 0.031 | 0.453 | 0.651 | | 0.983 | 0.045 |
| CD244 | CRP | 0.037 | 0.420 | 0.675 | | 0.983 | 0.088 |
| ADA | CRP | 0.017 | 0.399 | 0.690 | | 0.983 | 0.086 |
| IL6 | CRP | -0.054 | -0.398 | 0.691 | | 0.983 | -0.064 |
| IL.10RA | CRP | 0.013 | 0.379 | 0.705 | | 0.983 | 0.065 |
| CXCL11 | CRP | 0.054 | 0.355 | 0.723 | | 0.983 | 0.063 |
| CCL3 | CRP | 0.070 | 0.345 | 0.731 | | 0.983 | 0.071 |
| IL.24 | CRP | 0.028 | 0.336 | 0.738 | | 0.983 | 0.071 |
| CST5 | CRP | -0.040 | -0.332 | 0.741 | | 0.983 | -0.003 |
| AXIN1 | CRP | 0.025 | 0.323 | 0.747 | | 0.983 | 0.054 |
| IL2 | CRP | -0.015 | -0.310 | 0.757 | | 0.983 | -0.053 |
| IL33 | CRP | 0.013 | 0.286 | 0.775 | | 0.983 | 0.052 |
| CD6 | CRP | 0.032 | 0.281 | 0.779 | | 0.983 | 0.050 |
| TNFB | CRP | 0.012 | 0.237 | 0.813 | | 0.983 | 0.061 |
| CCL4 | CRP | -0.021 | -0.225 | 0.822 | | 0.983 | -0.062 |
| NT.3 | CRP | -0.012 | -0.220 | 0.826 | | 0.983 | -0.012 |
| uPA | CRP | -0.018 | -0.217 | 0.829 | | 0.983 | -0.030 |
| CCL19 | CRP | -0.017 | -0.214 | 0.831 | | 0.983 | -0.038 |
| OSM | CRP | 0.022 | 0.212 | 0.832 | | 0.983 | 0.033 |
| MMP.1 | CRP | -0.032 | -0.209 | 0.835 | | 0.983 | -0.042 |
| ST1A1 | CRP | -0.015 | -0.195 | 0.846 | | 0.983 | -0.046 |
| SLAMF1 | CRP | -0.010 | -0.180 | 0.858 | | 0.983 | -0.033 |
| Beta.NGF | CRP | 0.003 | 0.175 | 0.861 | | 0.983 | 0.048 |
| CD5 | CRP | 0.021 | 0.173 | 0.863 | | 0.983 | 0.037 |
| TRAIL | CRP | -0.011 | -0.142 | 0.887 | | 0.983 | -0.022 |
| CXCL1 | CRP | 0.010 | 0.139 | 0.889 | | 0.983 | 0.039 |
| X4E.BP1 | CRP | -0.004 | -0.138 | 0.890 | | 0.983 | -0.023 |
| IL.18R1 | CRP | -0.015 | -0.126 | 0.900 | | 0.983 | -0.024 |
| IL13 | CRP | 0.005 | 0.118 | 0.906 | | 0.983 | 0.004 |
| IL.10RB | CRP | -0.006 | -0.100 | 0.921 | | 0.983 | -0.020 |
| SIRT2 | CRP | 0.003 | 0.098 | 0.922 | | 0.983 | 0.062 |
| CCL25 | CRP | 0.006 | 0.081 | 0.936 | | 0.983 | 0.046 |
| VEGFA | CRP | 0.007 | 0.072 | 0.943 | | 0.983 | 0.035 |
| PD.L1 | CRP | 0.004 | 0.059 | 0.953 | | 0.983 | -0.013 |
| TNFSF14 | CRP | 0.005 | 0.053 | 0.957 | | 0.983 | 0.010 |
| NRTN | CRP | -0.002 | -0.051 | 0.959 | | 0.983 | 0.002 |
| CD40 | CRP | -0.003 | -0.036 | 0.971 | | 0.983 | -0.004 |
| CSF.1 | CRP | -0.002 | -0.035 | 0.972 | | 0.983 | 0.002 |
| CXCL10 | IL1B | 0.002 | 0.021 | 0.983 | | 0.983 | 0.013 |
| IL8 | IL17A | -0.097 | -0.824 | 0.411 | | 0.986 | -0.116 |
| EN.RAGE | IL17A | 0.068 | 0.822 | 0.412 | | 0.986 | 0.151 |
| FGF.21 | IL17A | 0.138 | 0.786 | 0.433 | | 0.986 | 0.122 |
| HGF | IL17A | -0.070 | -0.714 | 0.476 | | 0.986 | -0.154 |
| CST5 | IL17A | -0.087 | -0.710 | 0.478 | | 0.986 | -0.186 |
| CX3CL1 | IL17A | -0.049 | -0.704 | 0.482 | | 0.986 | -0.051 |
| TRANCE | IL17A | -0.061 | -0.686 | 0.494 | | 0.986 | -0.077 |
| CCL28 | IL17A | 0.023 | 0.679 | 0.498 | | 0.986 | 0.119 |
| IL4 | IL17A | -0.041 | -0.655 | 0.514 | | 0.986 | -0.161 |
| IL.1.alpha | IL17A | -0.152 | -0.652 | 0.515 | | 0.986 | 0.027 |
| FGF.23 | IL17A | -0.052 | -0.608 | 0.544 | | 0.986 | -0.106 |
| CCL4 | IL17A | -0.056 | -0.596 | 0.552 | | 0.986 | -0.045 |
| IL.20RA | IL17A | 0.024 | 0.588 | 0.557 | | 0.986 | 0.017 |
| MMP.1 | IL17A | -0.089 | -0.574 | 0.567 | | 0.986 | -0.088 |
| IL13 | IL17A | 0.023 | 0.556 | 0.579 | | 0.986 | 0.131 |
| MCP.1 | IL17A | -0.081 | -0.551 | 0.582 | | 0.986 | -0.010 |
| IL.22.RA1 | IL17A | 0.050 | 0.546 | 0.586 | | 0.986 | 0.082 |
| TNFSF14 | IL17A | 0.055 | 0.540 | 0.590 | | 0.986 | 0.073 |
| IL10 | IL17A | 0.031 | 0.525 | 0.600 | | 0.986 | 0.020 |
| IL33 | IL17A | 0.025 | 0.522 | 0.602 | | 0.986 | 0.081 |
| ARTN | IL17A | -0.030 | -0.518 | 0.605 | | 0.986 | -0.063 |
| CD5 | IL17A | 0.064 | 0.509 | 0.611 | | 0.986 | 0.040 |
| CXCL10 | IL17A | -0.052 | -0.501 | 0.617 | | 0.986 | -0.054 |
| IL.20 | IL17A | -0.019 | -0.483 | 0.630 | | 0.986 | -0.039 |
| SLAMF1 | IL17A | 0.026 | 0.471 | 0.638 | | 0.986 | 0.093 |
| IL7 | IL17A | -0.022 | -0.458 | 0.648 | | 0.986 | -0.026 |
| MMP.10 | IL17A | -0.044 | -0.452 | 0.652 | | 0.986 | 0.003 |
| TRAIL | IL17A | -0.031 | -0.406 | 0.685 | | 0.986 | -0.060 |
| TNF | IL17A | -0.033 | -0.393 | 0.695 | | 0.986 | -0.110 |
| CXCL5 | IL17A | -0.053 | -0.379 | 0.705 | | 0.986 | -0.045 |
| STAMBP | IL17A | 0.008 | 0.363 | 0.717 | | 0.986 | 0.039 |
| IL.10RA | IL17A | -0.012 | -0.346 | 0.730 | | 0.986 | -0.053 |
| OPG | IL17A | -0.024 | -0.341 | 0.733 | | 0.986 | -0.060 |
| CD40 | IL17A | 0.031 | 0.335 | 0.738 | | 0.986 | 0.041 |
| IL.18R1 | IL17A | -0.038 | -0.325 | 0.745 | | 0.986 | -0.059 |
| uPA | IL17A | 0.025 | 0.306 | 0.760 | | 0.986 | 0.031 |
| MCP.2 | IL17A | -0.029 | -0.273 | 0.785 | | 0.986 | -0.079 |
| CASP.8 | IL17A | 0.015 | 0.253 | 0.800 | | 0.986 | 0.019 |
| ADA | IL17A | -0.011 | -0.245 | 0.807 | | 0.986 | -0.103 |
| TGF.alpha | IL17A | 0.018 | 0.211 | 0.833 | | 0.986 | -0.013 |
| CCL20 | IL17A | -0.024 | -0.207 | 0.836 | | 0.986 | -0.077 |
| SIRT2 | IL17A | -0.007 | -0.189 | 0.850 | | 0.986 | -0.135 |
| FGF.5 | IL17A | -0.012 | -0.188 | 0.851 | | 0.986 | 0.014 |
| CCL25 | IL17A | -0.014 | -0.184 | 0.854 | | 0.986 | -0.122 |
| PD.L1 | IL17A | -0.012 | -0.174 | 0.862 | | 0.986 | 0.018 |
| CCL19 | IL17A | -0.014 | -0.172 | 0.863 | | 0.986 | 0.024 |
| CD6 | IL17A | 0.020 | 0.171 | 0.865 | | 0.986 | -0.002 |
| MCP.3 | IL17A | 0.021 | 0.168 | 0.867 | | 0.986 | 0.131 |
| CXCL1 | IL17A | -0.012 | -0.157 | 0.875 | | 0.986 | -0.062 |
| LAP.TGF.beta.1 | IL17A | 0.010 | 0.150 | 0.881 | | 0.986 | 0.019 |
| TSLP | IL17A | -0.006 | -0.106 | 0.915 | | 0.986 | 0.038 |
| TWEAK | IL17A | -0.006 | -0.097 | 0.922 | | 0.986 | 0.058 |
| CCL3 | IL17A | -0.020 | -0.096 | 0.924 | | 0.986 | -0.049 |
| Beta.NGF | IL17A | 0.001 | 0.065 | 0.948 | | 0.986 | -0.011 |
| MCP.4 | IL17A | -0.009 | -0.064 | 0.949 | | 0.986 | 0.024 |
| CD244 | IL17A | 0.005 | 0.058 | 0.954 | | 0.986 | -0.042 |
| CXCL6 | IL17A | 0.005 | 0.056 | 0.955 | | 0.986 | -0.040 |
| AXIN1 | IL17A | 0.004 | 0.051 | 0.959 | | 0.986 | -0.001 |
| LIF | IL17A | -0.002 | -0.046 | 0.963 | | 0.986 | -0.008 |
| CDCP1 | IL17A | -0.002 | -0.034 | 0.973 | | 0.986 | -0.040 |
| CD8A | IL17A | -0.003 | -0.027 | 0.979 | | 0.986 | -0.018 |
| CCL11 | IL17A | -0.002 | -0.017 | 0.986 | | 0.986 | 0.068 |
| FGF.19 | IL6 | -0.252 | -2.104 | 0.037 | | 0.996 | -0.345 |
| CCL19 | IL6 | 0.168 | 2.063 | 0.040 | | 0.996 | 0.396 |
| IL.20RA | IL6 | 0.071 | 1.743 | 0.083 | | 0.996 | 0.221 |
| CSF.1 | IL6 | 0.095 | 1.582 | 0.115 | | 0.996 | 0.255 |
| IL.12B | IL6 | 0.153 | 1.568 | 0.119 | | 0.996 | 0.403 |
| CX3CL1 | IL6 | 0.096 | 1.382 | 0.169 | | 0.996 | 0.276 |
| TSLP | IL6 | 0.072 | 1.360 | 0.176 | | 0.996 | 0.227 |
| TGF.alpha | IL6 | -0.108 | -1.295 | 0.197 | | 0.996 | -0.201 |
| CASP.8 | IL6 | -0.073 | -1.261 | 0.209 | | 0.996 | -0.151 |
| VEGFA | IL6 | -0.112 | -1.221 | 0.224 | | 0.996 | -0.182 |
| EN.RAGE | IL6 | -0.097 | -1.175 | 0.242 | | 0.996 | -0.122 |
| PD.L1 | IL6 | 0.082 | 1.167 | 0.245 | | 0.996 | 0.190 |
| CCL25 | IL6 | 0.087 | 1.154 | 0.250 | | 0.996 | 0.060 |
| ST1A1 | IL6 | -0.089 | -1.133 | 0.258 | | 0.996 | -0.146 |
| CD6 | IL6 | 0.127 | 1.099 | 0.273 | | 0.996 | 0.164 |
| CCL20 | IL6 | 0.120 | 1.022 | 0.308 | | 0.996 | 0.129 |
| FGF.23 | IL6 | -0.085 | -0.987 | 0.325 | | 0.996 | -0.160 |
| TWEAK | IL6 | -0.058 | -0.954 | 0.341 | | 0.996 | -0.077 |
| CD8A | IL6 | 0.093 | 0.947 | 0.345 | | 0.996 | 0.139 |
| IL33 | IL6 | 0.044 | 0.911 | 0.363 | | 0.996 | 0.170 |
| IL.24 | IL6 | 0.072 | 0.869 | 0.386 | | 0.996 | 0.113 |
| CD5 | IL6 | 0.109 | 0.869 | 0.386 | | 0.996 | 0.109 |
| MCP.3 | IL6 | 0.103 | 0.825 | 0.410 | | 0.996 | 0.235 |
| CCL28 | IL6 | 0.028 | 0.818 | 0.414 | | 0.996 | 0.110 |
| IL8 | IL6 | 0.095 | 0.811 | 0.419 | | 0.996 | 0.126 |
| IL4 | IL6 | 0.048 | 0.783 | 0.435 | | 0.996 | 0.111 |
| TNFSF14 | IL6 | -0.073 | -0.724 | 0.470 | | 0.996 | -0.042 |
| NT.3 | IL6 | 0.039 | 0.720 | 0.472 | | 0.996 | 0.109 |
| Flt3L | IL6 | 0.051 | 0.708 | 0.480 | | 0.996 | 0.149 |
| IL.2RB | IL6 | 0.029 | 0.691 | 0.490 | | 0.996 | 0.162 |
| MCP.2 | IL6 | -0.072 | -0.687 | 0.493 | | 0.996 | -0.112 |
| FGF.21 | IL6 | 0.119 | 0.680 | 0.497 | | 0.996 | 0.115 |
| IL.22.RA1 | IL6 | -0.062 | -0.679 | 0.498 | | 0.996 | -0.096 |
| TNFB | IL6 | 0.035 | 0.677 | 0.499 | | 0.996 | 0.064 |
| ADA | IL6 | 0.029 | 0.673 | 0.501 | | 0.996 | 0.055 |
| IL5 | IL6 | -0.036 | -0.673 | 0.502 | | 0.996 | -0.128 |
| CCL4 | IL6 | 0.061 | 0.653 | 0.515 | | 0.996 | 0.147 |
| OPG | IL6 | -0.045 | -0.634 | 0.527 | | 0.996 | -0.124 |
| IL.17C | IL6 | 0.076 | 0.614 | 0.540 | | 0.996 | 0.174 |
| IL.20 | IL6 | -0.024 | -0.612 | 0.541 | | 0.996 | -0.048 |
| MMP.10 | IL6 | 0.058 | 0.598 | 0.550 | | 0.996 | 0.103 |
| IL13 | IL6 | 0.025 | 0.595 | 0.553 | | 0.996 | 0.143 |
| LIF.R | IL6 | 0.016 | 0.595 | 0.553 | | 0.996 | 0.086 |
| CXCL5 | IL6 | -0.082 | -0.584 | 0.560 | | 0.996 | -0.107 |
| FGF.5 | IL6 | 0.035 | 0.568 | 0.570 | | 0.996 | 0.125 |
| CXCL10 | IL6 | 0.058 | 0.561 | 0.575 | | 0.996 | 0.135 |
| MMP.1 | IL6 | 0.084 | 0.541 | 0.589 | | 0.996 | 0.030 |
| SLAMF1 | IL6 | 0.029 | 0.524 | 0.601 | | 0.996 | 0.129 |
| CD244 | IL6 | -0.046 | -0.523 | 0.602 | | 0.996 | -0.123 |
| DNER | IL6 | -0.030 | -0.492 | 0.624 | | 0.996 | -0.134 |
| uPA | IL6 | -0.039 | -0.473 | 0.636 | | 0.996 | -0.024 |
| IL.10RB | IL6 | 0.030 | 0.466 | 0.641 | | 0.996 | 0.079 |
| CST5 | IL6 | 0.056 | 0.456 | 0.649 | | 0.996 | -0.062 |
| LIF | IL6 | 0.019 | 0.438 | 0.662 | | 0.996 | 0.116 |
| IL.10RA | IL6 | 0.014 | 0.418 | 0.676 | | 0.996 | 0.092 |
| AXIN1 | IL6 | 0.031 | 0.395 | 0.693 | | 0.996 | 0.063 |
| CCL11 | IL6 | 0.050 | 0.373 | 0.710 | | 0.996 | 0.130 |
| TRANCE | IL6 | 0.031 | 0.357 | 0.721 | | 0.996 | 0.094 |
| MCP.4 | IL6 | 0.048 | 0.349 | 0.727 | | 0.996 | 0.106 |
| IL.1.alpha | IL6 | 0.075 | 0.324 | 0.747 | | 0.996 | 0.198 |
| CCL23 | IL6 | -0.036 | -0.320 | 0.749 | | 0.996 | 0.028 |
| SIRT2 | IL6 | -0.010 | -0.298 | 0.766 | | 0.996 | -0.128 |
| IL.15RA | IL6 | -0.015 | -0.297 | 0.767 | | 0.996 | -0.088 |
| LAP.TGF.beta.1 | IL6 | -0.020 | -0.297 | 0.767 | | 0.996 | -0.064 |
| TNFRSF9 | IL6 | -0.025 | -0.288 | 0.774 | | 0.996 | 0.033 |
| IL6 | IL6 | -0.038 | -0.279 | 0.781 | | 0.996 | -0.055 |
| OSM | IL6 | 0.029 | 0.276 | 0.783 | | 0.996 | 0.125 |
| TRAIL | IL6 | 0.021 | 0.267 | 0.790 | | 0.996 | 0.096 |
| HGF | IL6 | -0.025 | -0.251 | 0.802 | | 0.996 | -0.027 |
| CD40 | IL6 | 0.023 | 0.246 | 0.806 | | 0.996 | 0.019 |
| CXCL9 | IL6 | 0.018 | 0.232 | 0.817 | | 0.996 | 0.031 |
| IL10 | IL6 | 0.013 | 0.218 | 0.828 | | 0.996 | -0.017 |
| TNF | IL6 | -0.018 | -0.215 | 0.830 | | 0.996 | -0.061 |
| GDNF | IL6 | 0.012 | 0.195 | 0.845 | | 0.996 | 0.054 |
| IL18 | IL6 | 0.017 | 0.173 | 0.863 | | 0.996 | 0.035 |
| CDCP1 | IL6 | -0.009 | -0.165 | 0.869 | | 0.996 | -0.049 |
| IL2 | IL6 | -0.008 | -0.162 | 0.871 | | 0.996 | -0.002 |
| IL.18R1 | IL6 | 0.018 | 0.159 | 0.874 | | 0.996 | 0.031 |
| IL7 | IL6 | -0.007 | -0.148 | 0.882 | | 0.996 | -0.001 |
| CXCL11 | IL6 | 0.022 | 0.145 | 0.885 | | 0.996 | -0.002 |
| STAMBP | IL6 | 0.003 | 0.140 | 0.889 | | 0.996 | -0.014 |
| CXCL1 | IL6 | -0.007 | -0.097 | 0.923 | | 0.996 | 0.004 |
| Beta.NGF | IL6 | 0.001 | 0.076 | 0.939 | | 0.996 | -0.009 |
| MCP.1 | IL6 | -0.009 | -0.063 | 0.950 | | 0.996 | 0.091 |
| IL.17A | IL6 | -0.003 | -0.052 | 0.959 | | 0.996 | -0.002 |
| IFN.gamma | IL6 | -0.008 | -0.045 | 0.965 | | 0.996 | -0.044 |
| ARTN | IL6 | 0.002 | 0.034 | 0.973 | | 0.996 | 0.034 |
| SCF | IL6 | -0.002 | -0.027 | 0.979 | | 0.996 | -0.032 |
| X4E.BP1 | IL6 | 0.001 | 0.019 | 0.985 | | 0.996 | -0.019 |
| CCL3 | IL6 | 0.002 | 0.012 | 0.991 | | 0.996 | -0.017 |
| NRTN | IL6 | 0.000 | -0.007 | 0.995 | | 0.996 | -0.002 |
| CXCL6 | IL6 | -0.001 | -0.006 | 0.996 | | 0.996 | -0.007 |
| CXCL6 | CRP | 0.001 | 0.005 | 0.996 | | 0.996 | 0.018 |
| LogFC between groups and p values were calculated with limma package in R. FDR correction method is used to calculate adjusted p-values.  Significance is defined as FDR-adjusted p-value <0.05 (*)  Cohen’s d is reported to indicate observed effect size for the respective comparison.  Models were adjusted for maternal inflammatory marker batch, child age at DBS collection, time between maternal sample date and child birthdate, and the first two principal components (PC1 and PC2) of the collinear neonatal covariates. | | | | | | | |

# **Supplementary Table 15.** Differential expression results for all neonatal inflammatory markers between high vs. low maternal inflammation in the third trimester (secondary analysis; n=235), ordered by adjusted p-value

| **Neonatal  inflammatory marker** | **High Maternal Inflammatory Marker** | **logFC** | **t-value** | **p-value** | **Adjusted p-value** | **Cohen's d** |
| --- | --- | --- | --- | --- | --- | --- |
| MCP.2 | IL1B | 0.272 | 3.299 | 0.001 | 0.103 | 0.435 |
| TNF | IL6 | 0.265 | 3.225 | 0.001 | 0.133 | 0.437 |
| CDCP1 | IL6 | 0.168 | 2.520 | 0.012 | 0.252 | 0.363 |
| IL.17C | IL6 | 0.304 | 2.489 | 0.014 | 0.252 | 0.378 |
| CX3CL1 | IL6 | 0.156 | 2.385 | 0.018 | 0.252 | 0.383 |
| IL.22.RA1 | IL6 | 0.199 | 2.344 | 0.020 | 0.252 | 0.325 |
| GDNF | IL6 | 0.132 | 2.300 | 0.022 | 0.252 | 0.355 |
| CCL28 | IL6 | 0.104 | 2.300 | 0.022 | 0.252 | 0.314 |
| IL5 | IL6 | 0.128 | 2.296 | 0.023 | 0.252 | 0.339 |
| TWEAK | IL6 | 0.152 | 2.262 | 0.025 | 0.252 | 0.378 |
| HGF | IL6 | -0.232 | -2.189 | 0.030 | 0.272 | -0.249 |
| IL.20 | IL6 | 0.077 | 2.149 | 0.033 | 0.273 | 0.292 |
| IL.2RB | IL6 | 0.083 | 2.027 | 0.044 | 0.336 | 0.333 |
| IL4 | IL6 | 0.095 | 1.984 | 0.048 | 0.343 | 0.349 |
| TNFSF14 | IL6 | -0.199 | -1.937 | 0.054 | 0.353 | -0.219 |
| CD8A | IL6 | 0.193 | 1.908 | 0.058 | 0.353 | 0.206 |
| IL33 | IL6 | 0.080 | 1.818 | 0.070 | 0.404 | 0.314 |
| CXCL10 | IL6 | 0.192 | 1.785 | 0.076 | 0.409 | 0.209 |
| SLAMF1 | IL6 | 0.098 | 1.746 | 0.082 | 0.412 | 0.271 |
| CXCL9 | IL6 | 0.141 | 1.724 | 0.086 | 0.412 | 0.200 |
| CD40 | IL6 | 0.164 | 1.703 | 0.090 | 0.412 | 0.198 |
| LAP.TGF.beta.1 | IL6 | 0.115 | 1.672 | 0.096 | 0.412 | 0.203 |
| IL7 | IL6 | 0.081 | 1.655 | 0.099 | 0.412 | 0.245 |
| EN.RAGE | IL6 | -0.145 | -1.601 | 0.111 | 0.412 | -0.155 |
| IL.12B | IL6 | 0.146 | 1.598 | 0.111 | 0.412 | 0.253 |
| CST5 | IL6 | 0.224 | 1.570 | 0.118 | 0.412 | 0.146 |
| LIF.R | IL6 | 0.047 | 1.565 | 0.119 | 0.412 | 0.228 |
| MCP.2 | IL6 | 0.131 | 1.545 | 0.124 | 0.412 | 0.193 |
| SCF | IL6 | 0.112 | 1.539 | 0.125 | 0.412 | 0.179 |
| IL4 | IL17A | 0.129 | 2.757 | 0.006 | 0.432 | 0.446 |
| CCL28 | IL17A | 0.110 | 2.486 | 0.014 | 0.432 | 0.322 |
| STAMBP | IL17A | 0.053 | 2.474 | 0.014 | 0.432 | 0.337 |
| FGF.23 | IL6 | 0.091 | 1.480 | 0.140 | 0.433 | 0.260 |
| TSLP | IL6 | 0.081 | 1.477 | 0.141 | 0.433 | 0.254 |
| IL2 | IL17A | 0.117 | 2.317 | 0.021 | 0.449 | 0.351 |
| IL33 | IL17A | 0.096 | 2.210 | 0.028 | 0.449 | 0.329 |
| NT.3 | IL17A | 0.120 | 2.143 | 0.033 | 0.449 | 0.292 |
| ADA | IL17A | 0.087 | 2.108 | 0.036 | 0.449 | 0.251 |
| IL.20RA | IL17A | 0.088 | 2.076 | 0.039 | 0.449 | 0.306 |
| OSM | IL6 | -0.160 | -1.434 | 0.153 | 0.454 | -0.104 |
| X4E.BP1 | IL6 | 0.041 | 1.401 | 0.163 | 0.467 | 0.167 |
| CD6 | IL6 | 0.155 | 1.360 | 0.175 | 0.488 | 0.161 |
| GDNF | IL17A | 0.111 | 1.942 | 0.053 | 0.522 | 0.222 |
| IL.20 | IL17A | 0.068 | 1.915 | 0.057 | 0.522 | 0.289 |
| LIF | IL6 | 0.047 | 1.284 | 0.200 | 0.539 | 0.213 |
| IL.24 | IL6 | 0.104 | 1.271 | 0.205 | 0.539 | 0.123 |
| CXCL11 | IL6 | 0.166 | 1.250 | 0.213 | 0.543 | 0.141 |
| IL2 | IL6 | 0.062 | 1.206 | 0.229 | 0.559 | 0.169 |
| CCL20 | IL6 | 0.146 | 1.183 | 0.238 | 0.559 | 0.118 |
| ADA | IL6 | 0.049 | 1.168 | 0.244 | 0.559 | 0.174 |
| FGF.5 | IL6 | 0.067 | 1.152 | 0.251 | 0.559 | 0.130 |
| IL.10RA | IL6 | 0.037 | 1.146 | 0.253 | 0.559 | 0.124 |
| IL10 | IL6 | 0.058 | 1.141 | 0.255 | 0.559 | 0.165 |
| IL.20RA | IL6 | 0.049 | 1.120 | 0.264 | 0.564 | 0.212 |
| TGF.alpha | IL6 | -0.100 | -1.091 | 0.276 | 0.578 | -0.176 |
| TNFB | IL6 | 0.048 | 1.065 | 0.288 | 0.589 | 0.110 |
| VEGFA | IL6 | -0.099 | -1.043 | 0.298 | 0.596 | -0.115 |
| MCP.3 | IL17A | 0.193 | 1.795 | 0.074 | 0.619 | 0.277 |
| TRANCE | IL6 | 0.079 | 0.964 | 0.336 | 0.650 | 0.081 |
| CCL25 | IL6 | 0.079 | 0.958 | 0.339 | 0.650 | 0.071 |
| NRTN | IL17A | 0.064 | 1.729 | 0.085 | 0.652 | 0.260 |
| FGF.5 | IL17A | 0.096 | 1.671 | 0.096 | 0.666 | 0.260 |
| Flt3L | IL17A | 0.116 | 1.634 | 0.104 | 0.666 | 0.123 |
| SIRT2 | IL17A | 0.053 | 1.610 | 0.109 | 0.666 | 0.046 |
| TNFRSF9 | IL17A | -0.123 | -1.546 | 0.123 | 0.676 | -0.263 |
| MCP.4 | IL17A | 0.186 | 1.526 | 0.128 | 0.676 | 0.159 |
| IL10 | IL17A | 0.076 | 1.510 | 0.132 | 0.676 | 0.226 |
| CCL11 | IL6 | 0.119 | 0.886 | 0.377 | 0.704 | 0.129 |
| NRTN | IL6 | 0.033 | 0.875 | 0.383 | 0.704 | 0.150 |
| IL5 | IL17A | 0.079 | 1.429 | 0.154 | 0.716 | 0.186 |
| ST1A1 | IL17A | -0.107 | -1.404 | 0.162 | 0.716 | -0.236 |
| IL13 | IL17A | 0.057 | 1.381 | 0.168 | 0.716 | 0.258 |
| IL6 | IL17A | 0.175 | 1.357 | 0.176 | 0.716 | 0.211 |
| IL.17A | IL17A | 0.071 | 1.348 | 0.179 | 0.716 | 0.199 |
| CCL23 | IL6 | -0.089 | -0.838 | 0.403 | 0.721 | -0.059 |
| ARTN | IL6 | 0.046 | 0.816 | 0.415 | 0.721 | 0.091 |
| CD244 | IL6 | 0.070 | 0.789 | 0.431 | 0.721 | 0.063 |
| SIRT2 | IL6 | -0.026 | -0.760 | 0.448 | 0.721 | -0.208 |
| MCP.4 | IL6 | 0.094 | 0.755 | 0.451 | 0.721 | 0.094 |
| IL18 | IL6 | 0.077 | 0.747 | 0.456 | 0.721 | 0.174 |
| PD.L1 | IL6 | 0.056 | 0.744 | 0.458 | 0.721 | 0.035 |
| CCL4 | IL6 | 0.067 | 0.738 | 0.461 | 0.721 | 0.117 |
| uPA | IL6 | -0.060 | -0.728 | 0.468 | 0.721 | 0.003 |
| CCL19 | IL6 | 0.054 | 0.723 | 0.470 | 0.721 | 0.040 |
| MCP.3 | IL6 | 0.078 | 0.711 | 0.478 | 0.721 | 0.089 |
| STAMBP | IL6 | 0.015 | 0.694 | 0.488 | 0.725 | 0.048 |
| FGF.23 | IL17A | 0.080 | 1.314 | 0.190 | 0.729 | 0.182 |
| CD5 | IL6 | 0.081 | 0.662 | 0.509 | 0.739 | 0.052 |
| FGF.21 | IL6 | -0.106 | -0.654 | 0.514 | 0.739 | -0.124 |
| CASP.8 | IL6 | -0.037 | -0.604 | 0.547 | 0.750 | -0.102 |
| ST1A1 | IL6 | -0.047 | -0.599 | 0.550 | 0.750 | -0.102 |
| IL13 | IL6 | 0.025 | 0.593 | 0.554 | 0.750 | 0.125 |
| CXCL1 | IL6 | -0.050 | -0.581 | 0.562 | 0.750 | -0.017 |
| IL.17A | IL6 | 0.031 | 0.580 | 0.562 | 0.750 | 0.063 |
| IL.10RB | IL6 | -0.033 | -0.561 | 0.575 | 0.751 | -0.114 |
| IL.18R1 | IL6 | -0.062 | -0.555 | 0.579 | 0.751 | -0.068 |
| DNER | IL6 | 0.032 | 0.536 | 0.592 | 0.757 | -0.004 |
| MCP.1 | IL6 | 0.070 | 0.488 | 0.626 | 0.789 | 0.102 |
| AXIN1 | IL6 | 0.041 | 0.451 | 0.652 | 0.802 | 0.034 |
| IL.15RA | IL6 | -0.023 | -0.436 | 0.664 | 0.802 | -0.106 |
| TRAIL | IL6 | -0.033 | -0.419 | 0.676 | 0.802 | -0.070 |
| TNFRSF9 | IL6 | -0.034 | -0.417 | 0.677 | 0.802 | -0.063 |
| IFN.gamma | IL6 | -0.073 | -0.413 | 0.680 | 0.802 | -0.102 |
| OPG | IL6 | 0.027 | 0.402 | 0.688 | 0.802 | 0.007 |
| IL6 | IL6 | 0.051 | 0.389 | 0.698 | 0.802 | 0.138 |
| Flt3L | IL6 | 0.025 | 0.348 | 0.728 | 0.827 | -0.075 |
| IL.1.alpha | IL6 | 0.063 | 0.322 | 0.748 | 0.839 | 0.015 |
| CD8A | IL17A | 0.120 | 1.202 | 0.231 | 0.849 | 0.070 |
| IL.12B | IL17A | -0.104 | -1.160 | 0.247 | 0.865 | -0.123 |
| LIF.R | IL17A | -0.034 | -1.144 | 0.254 | 0.865 | -0.166 |
| CCL3 | IL17A | 0.204 | 1.107 | 0.270 | 0.886 | 0.090 |
| CCL3 | IL6 | -0.048 | -0.254 | 0.799 | 0.886 | -0.056 |
| CXCL6 | IL6 | -0.025 | -0.241 | 0.810 | 0.887 | -0.006 |
| IL8 | IL6 | 0.028 | 0.218 | 0.828 | 0.896 | 0.029 |
| VEGFA | IL17A | -0.089 | -0.954 | 0.341 | 0.905 | -0.203 |
| PD.L1 | IL17A | 0.071 | 0.948 | 0.344 | 0.905 | 0.036 |
| CCL25 | IL17A | -0.074 | -0.912 | 0.363 | 0.905 | -0.225 |
| CD6 | IL17A | 0.102 | 0.910 | 0.364 | 0.905 | 0.039 |
| IL.15RA | IL17A | 0.045 | 0.876 | 0.382 | 0.905 | 0.119 |
| IL.17C | IL17A | 0.101 | 0.828 | 0.408 | 0.905 | 0.119 |
| IL.10RB | IL17A | -0.047 | -0.801 | 0.424 | 0.905 | -0.147 |
| IL.2RB | IL17A | 0.032 | 0.801 | 0.424 | 0.905 | 0.144 |
| MCP.1 | IL17A | 0.111 | 0.792 | 0.429 | 0.905 | 0.142 |
| TRAIL | IL17A | 0.060 | 0.769 | 0.443 | 0.905 | 0.031 |
| CCL11 | IL17A | 0.102 | 0.767 | 0.444 | 0.905 | 0.097 |
| ARTN | IL17A | 0.043 | 0.762 | 0.447 | 0.905 | 0.152 |
| TSLP | IL17A | -0.041 | -0.761 | 0.447 | 0.905 | -0.149 |
| CXCL11 | IL17A | 0.099 | 0.751 | 0.454 | 0.905 | 0.086 |
| IL18 | IL17A | -0.075 | -0.743 | 0.458 | 0.905 | -0.030 |
| TNFB | IL17A | -0.031 | -0.694 | 0.489 | 0.905 | -0.167 |
| IL.1.alpha | IL17A | 0.130 | 0.678 | 0.499 | 0.905 | 0.055 |
| TNFSF14 | IL17A | -0.069 | -0.674 | 0.501 | 0.905 | -0.133 |
| IL7 | IL17A | 0.032 | 0.664 | 0.507 | 0.905 | 0.071 |
| TNF | IL17A | -0.055 | -0.663 | 0.508 | 0.905 | -0.137 |
| FGF.19 | IL17A | 0.078 | 0.657 | 0.512 | 0.905 | 0.081 |
| CD40 | IL17A | 0.062 | 0.645 | 0.520 | 0.905 | -0.021 |
| IL.22.RA1 | IL17A | 0.054 | 0.643 | 0.521 | 0.905 | 0.136 |
| AXIN1 | IL17A | 0.057 | 0.639 | 0.523 | 0.905 | 0.014 |
| LIF | IL17A | 0.022 | 0.622 | 0.534 | 0.905 | 0.120 |
| EN.RAGE | IL17A | -0.055 | -0.619 | 0.537 | 0.905 | -0.032 |
| X4E.BP1 | IL17A | -0.017 | -0.603 | 0.547 | 0.905 | -0.107 |
| Beta.NGF | IL17A | 0.010 | 0.597 | 0.551 | 0.905 | 0.082 |
| CXCL5 | IL6 | 0.029 | 0.191 | 0.849 | 0.908 | -0.014 |
| MCP.2 | IL17A | 0.046 | 0.551 | 0.582 | 0.940 | 0.034 |
| CXCL6 | IL17A | 0.054 | 0.523 | 0.602 | 0.954 | 0.040 |
| CD5 | IL17A | 0.060 | 0.498 | 0.619 | 0.956 | -0.056 |
| OSM | IL17A | -0.050 | -0.451 | 0.653 | 0.956 | -0.055 |
| SLAMF1 | IL17A | 0.024 | 0.436 | 0.663 | 0.956 | 0.056 |
| CCL23 | IL17A | 0.044 | 0.425 | 0.671 | 0.956 | 0.062 |
| CCL4 | IL17A | 0.037 | 0.416 | 0.678 | 0.956 | 0.014 |
| MMP.10 | IL17A | 0.035 | 0.399 | 0.690 | 0.956 | -0.035 |
| OPG | IL17A | 0.027 | 0.398 | 0.691 | 0.956 | -0.057 |
| CXCL10 | IL17A | 0.042 | 0.393 | 0.695 | 0.956 | 0.037 |
| CX3CL1 | IL17A | 0.025 | 0.388 | 0.699 | 0.956 | 0.050 |
| TRANCE | IL17A | -0.030 | -0.368 | 0.714 | 0.956 | -0.062 |
| uPA | IL17A | -0.028 | -0.346 | 0.729 | 0.956 | -0.063 |
| CXCL1 | IL17A | 0.028 | 0.338 | 0.736 | 0.956 | 0.022 |
| DNER | IL17A | -0.019 | -0.331 | 0.741 | 0.956 | -0.165 |
| TWEAK | IL17A | 0.021 | 0.309 | 0.757 | 0.956 | 0.012 |
| IFN.gamma | IL17A | 0.049 | 0.283 | 0.778 | 0.956 | 0.005 |
| CCL20 | IL17A | -0.034 | -0.282 | 0.778 | 0.956 | -0.111 |
| CD244 | IL17A | -0.024 | -0.271 | 0.787 | 0.956 | -0.190 |
| HGF | IL17A | -0.028 | -0.267 | 0.789 | 0.956 | -0.070 |
| LAP.TGF.beta.1 | IL17A | -0.016 | -0.239 | 0.812 | 0.964 | -0.114 |
| CXCL9 | IL17A | 0.019 | 0.229 | 0.819 | 0.964 | -0.019 |
| IL.18R1 | IL17A | 0.023 | 0.211 | 0.833 | 0.964 | -0.036 |
| IL8 | IL17A | 0.026 | 0.205 | 0.838 | 0.964 | -0.026 |
| FGF.21 | IL17A | -0.030 | -0.191 | 0.849 | 0.964 | -0.028 |
| ADA | CRP | 0.096 | 2.342 | 0.020 | 0.967 | 0.378 |
| TRANCE | CRP | -0.172 | -2.165 | 0.031 | 0.967 | -0.323 |
| TWEAK | CRP | -0.127 | -1.928 | 0.055 | 0.967 | -0.257 |
| SCF | CRP | -0.121 | -1.704 | 0.090 | 0.967 | -0.215 |
| IL4 | CRP | -0.078 | -1.664 | 0.097 | 0.967 | -0.251 |
| TNF | CRP | -0.131 | -1.613 | 0.108 | 0.967 | -0.219 |
| LIF.R | CRP | -0.046 | -1.582 | 0.115 | 0.967 | -0.227 |
| IL13 | CRP | -0.064 | -1.579 | 0.116 | 0.967 | -0.203 |
| CCL20 | CRP | 0.176 | 1.495 | 0.136 | 0.967 | 0.194 |
| X4E.BP1 | CRP | -0.041 | -1.448 | 0.149 | 0.967 | -0.233 |
| IL.1.alpha | CRP | 0.236 | 1.247 | 0.214 | 0.967 | 0.201 |
| VEGFA | CRP | -0.113 | -1.219 | 0.224 | 0.967 | -0.148 |
| IFN.gamma | CRP | 0.200 | 1.180 | 0.239 | 0.967 | 0.110 |
| MMP.10 | CRP | -0.096 | -1.125 | 0.262 | 0.967 | -0.152 |
| CCL11 | CRP | -0.142 | -1.084 | 0.279 | 0.967 | -0.143 |
| CXCL6 | CRP | -0.109 | -1.074 | 0.284 | 0.967 | -0.160 |
| CCL23 | CRP | -0.110 | -1.068 | 0.287 | 0.967 | -0.118 |
| ARTN | CRP | 0.059 | 1.063 | 0.289 | 0.967 | 0.157 |
| IL.22.RA1 | CRP | -0.085 | -1.020 | 0.309 | 0.967 | -0.132 |
| IL.10RB | CRP | -0.058 | -1.009 | 0.314 | 0.967 | -0.159 |
| CXCL9 | CRP | 0.075 | 0.948 | 0.344 | 0.967 | 0.113 |
| Beta.NGF | CRP | -0.015 | -0.933 | 0.352 | 0.967 | -0.135 |
| SLAMF1 | CRP | -0.050 | -0.920 | 0.359 | 0.967 | -0.107 |
| SIRT2 | CRP | 0.030 | 0.912 | 0.363 | 0.967 | 0.140 |
| IL18 | CRP | 0.082 | 0.820 | 0.413 | 0.967 | 0.103 |
| LAP.TGF.beta.1 | CRP | -0.053 | -0.797 | 0.427 | 0.967 | -0.103 |
| IL.24 | CRP | -0.061 | -0.762 | 0.447 | 0.967 | -0.140 |
| LIF | CRP | -0.027 | -0.757 | 0.450 | 0.967 | -0.103 |
| NRTN | CRP | -0.027 | -0.738 | 0.462 | 0.967 | -0.104 |
| IL8 | CRP | 0.091 | 0.737 | 0.462 | 0.967 | 0.108 |
| ST1A1 | CRP | -0.055 | -0.731 | 0.466 | 0.967 | -0.092 |
| CXCL11 | CRP | 0.094 | 0.723 | 0.470 | 0.967 | 0.096 |
| IL.17C | CRP | -0.086 | -0.716 | 0.475 | 0.967 | -0.052 |
| IL2 | CRP | -0.035 | -0.708 | 0.480 | 0.967 | -0.100 |
| CSF.1 | CRP | 0.041 | 0.705 | 0.481 | 0.967 | 0.079 |
| TNFSF14 | CRP | -0.070 | -0.695 | 0.488 | 0.967 | -0.083 |
| IL10 | CRP | 0.034 | 0.688 | 0.492 | 0.967 | 0.111 |
| Flt3L | CRP | 0.048 | 0.688 | 0.492 | 0.967 | 0.114 |
| TNFRSF9 | CRP | -0.051 | -0.656 | 0.513 | 0.967 | -0.064 |
| IL.2RB | CRP | 0.026 | 0.652 | 0.515 | 0.967 | 0.136 |
| CASP.8 | CRP | 0.037 | 0.622 | 0.534 | 0.967 | 0.083 |
| CXCL1 | CRP | 0.051 | 0.621 | 0.535 | 0.967 | 0.115 |
| MCP.4 | CRP | -0.074 | -0.618 | 0.537 | 0.967 | -0.073 |
| MCP.1 | CRP | -0.086 | -0.617 | 0.538 | 0.967 | -0.102 |
| FGF.19 | CRP | 0.071 | 0.607 | 0.544 | 0.967 | 0.071 |
| NT.3 | CRP | 0.031 | 0.562 | 0.574 | 0.967 | 0.090 |
| TSLP | CRP | -0.030 | -0.557 | 0.578 | 0.967 | -0.078 |
| CXCL5 | CRP | 0.079 | 0.539 | 0.591 | 0.967 | 0.111 |
| IL.12B | CRP | -0.046 | -0.517 | 0.606 | 0.967 | -0.089 |
| CD244 | CRP | -0.044 | -0.506 | 0.614 | 0.967 | -0.040 |
| FGF.5 | CRP | 0.028 | 0.489 | 0.625 | 0.967 | 0.072 |
| IL7 | CRP | -0.023 | -0.478 | 0.633 | 0.967 | -0.048 |
| CD5 | CRP | -0.054 | -0.456 | 0.649 | 0.967 | -0.020 |
| OSM | CRP | -0.049 | -0.446 | 0.656 | 0.967 | -0.054 |
| IL.20 | CRP | 0.015 | 0.438 | 0.662 | 0.967 | 0.075 |
| GDNF | CRP | 0.024 | 0.434 | 0.665 | 0.967 | 0.088 |
| FGF.21 | CRP | 0.064 | 0.404 | 0.687 | 0.967 | 0.039 |
| AXIN1 | CRP | 0.034 | 0.392 | 0.695 | 0.967 | 0.070 |
| CD6 | CRP | -0.042 | -0.375 | 0.708 | 0.967 | -0.008 |
| OPG | CRP | 0.023 | 0.352 | 0.725 | 0.967 | 0.062 |
| MCP.2 | CRP | 0.029 | 0.348 | 0.728 | 0.967 | 0.049 |
| TRAIL | CRP | -0.027 | -0.345 | 0.730 | 0.967 | -0.039 |
| CCL28 | CRP | -0.015 | -0.345 | 0.731 | 0.967 | -0.035 |
| FGF.23 | CRP | 0.021 | 0.344 | 0.731 | 0.967 | 0.049 |
| IL6 | CRP | 0.043 | 0.338 | 0.735 | 0.967 | 0.035 |
| CXCL10 | CRP | 0.032 | 0.303 | 0.762 | 0.967 | -0.006 |
| STAMBP | CRP | 0.006 | 0.296 | 0.767 | 0.967 | 0.045 |
| CCL4 | CRP | -0.025 | -0.285 | 0.776 | 0.967 | -0.044 |
| IL5 | CRP | 0.014 | 0.265 | 0.792 | 0.967 | 0.058 |
| CCL3 | CRP | -0.041 | -0.227 | 0.820 | 0.967 | -0.014 |
| IL.17A | CRP | -0.012 | -0.226 | 0.822 | 0.967 | -0.041 |
| MMP.1 | CRP | 0.028 | 0.199 | 0.842 | 0.967 | 0.053 |
| CDCP1 | CRP | 0.013 | 0.198 | 0.843 | 0.967 | 0.023 |
| uPA | CRP | -0.015 | -0.184 | 0.854 | 0.967 | 0.013 |
| IL.18R1 | CRP | -0.018 | -0.161 | 0.872 | 0.967 | -0.009 |
| IL.20RA | CRP | -0.006 | -0.141 | 0.888 | 0.967 | -0.012 |
| HGF | CRP | 0.014 | 0.134 | 0.894 | 0.967 | 0.039 |
| CST5 | CRP | 0.018 | 0.131 | 0.896 | 0.967 | -0.008 |
| CD40 | CRP | -0.010 | -0.110 | 0.912 | 0.967 | 0.022 |
| IL33 | CRP | 0.004 | 0.104 | 0.918 | 0.967 | 0.021 |
| DNER | CRP | -0.006 | -0.101 | 0.919 | 0.967 | 0.026 |
| PD.L1 | CRP | 0.007 | 0.101 | 0.920 | 0.967 | 0.027 |
| MCP.3 | CRP | -0.010 | -0.089 | 0.929 | 0.967 | -0.032 |
| CX3CL1 | CRP | 0.005 | 0.073 | 0.942 | 0.967 | 0.052 |
| TGF.alpha | CRP | 0.006 | 0.067 | 0.947 | 0.967 | 0.042 |
| TNFB | CRP | 0.003 | 0.066 | 0.947 | 0.967 | -0.017 |
| CD8A | CRP | -0.006 | -0.065 | 0.948 | 0.967 | 0.023 |
| EN.RAGE | CRP | 0.005 | 0.057 | 0.955 | 0.967 | 0.017 |
| CCL25 | CRP | -0.004 | -0.050 | 0.960 | 0.967 | -0.048 |
| IL.10RA | CRP | 0.001 | 0.043 | 0.966 | 0.967 | 0.029 |
| IL.15RA | CRP | 0.002 | 0.041 | 0.967 | 0.967 | 0.035 |
| CCL19 | CRP | -0.003 | -0.041 | 0.967 | 0.967 | -0.057 |
| Beta.NGF | IL6 | 0.001 | 0.086 | 0.932 | 0.983 | 0.028 |
| MMP.1 | IL6 | -0.011 | -0.074 | 0.941 | 0.983 | -0.041 |
| MMP.10 | IL6 | -0.004 | -0.047 | 0.962 | 0.983 | -0.129 |
| CSF.1 | IL6 | 0.003 | 0.047 | 0.963 | 0.983 | -0.053 |
| FGF.19 | IL6 | 0.004 | 0.035 | 0.972 | 0.983 | -0.035 |
| MMP.1 | IL17A | 0.022 | 0.151 | 0.880 | 0.987 | -0.020 |
| CXCL5 | IL17A | 0.019 | 0.127 | 0.899 | 0.987 | -0.051 |
| CCL19 | IL17A | 0.008 | 0.108 | 0.914 | 0.987 | -0.029 |
| CDCP1 | IL17A | -0.007 | -0.098 | 0.922 | 0.987 | -0.013 |
| TGF.alpha | IL17A | -0.008 | -0.093 | 0.926 | 0.987 | -0.096 |
| IL.24 | IL17A | -0.006 | -0.078 | 0.938 | 0.987 | 0.019 |
| IL.10RA | IL17A | -0.002 | -0.071 | 0.944 | 0.987 | -0.021 |
| NT.3 | IL6 | 0.001 | 0.015 | 0.988 | 0.988 | 0.055 |
| CCL23 | IL1B | -0.182 | -1.752 | 0.081 | 0.991 | -0.266 |
| IL7 | IL1B | 0.079 | 1.622 | 0.106 | 0.991 | 0.196 |
| IL.10RB | IL1B | -0.092 | -1.577 | 0.116 | 0.991 | -0.257 |
| IL.20 | IL1B | 0.054 | 1.522 | 0.129 | 0.991 | 0.227 |
| TNFB | IL1B | -0.065 | -1.460 | 0.146 | 0.991 | -0.306 |
| TNFRSF9 | IL1B | -0.115 | -1.439 | 0.151 | 0.991 | -0.263 |
| Flt3L | IL1B | 0.100 | 1.407 | 0.161 | 0.991 | 0.085 |
| CCL28 | IL1B | 0.062 | 1.376 | 0.170 | 0.991 | 0.160 |
| CXCL1 | IL1B | 0.112 | 1.329 | 0.185 | 0.991 | 0.151 |
| CXCL5 | IL1B | 0.196 | 1.324 | 0.187 | 0.991 | 0.118 |
| CXCL6 | IL1B | 0.133 | 1.292 | 0.197 | 0.991 | 0.115 |
| IL.24 | IL1B | 0.103 | 1.267 | 0.206 | 0.991 | 0.185 |
| CCL3 | IL1B | 0.225 | 1.220 | 0.224 | 0.991 | 0.106 |
| CCL25 | IL1B | -0.099 | -1.216 | 0.225 | 0.991 | -0.275 |
| STAMBP | IL1B | 0.026 | 1.214 | 0.226 | 0.991 | 0.156 |
| FGF.5 | IL1B | 0.069 | 1.200 | 0.231 | 0.991 | 0.182 |
| IL.17C | IL1B | 0.144 | 1.184 | 0.238 | 0.991 | 0.172 |
| LIF.R | IL1B | -0.035 | -1.166 | 0.245 | 0.991 | -0.142 |
| IL6 | IL1B | 0.146 | 1.126 | 0.261 | 0.991 | 0.152 |
| IL.15RA | IL1B | -0.056 | -1.090 | 0.277 | 0.991 | -0.153 |
| TSLP | IL1B | -0.059 | -1.089 | 0.277 | 0.991 | -0.198 |
| DNER | IL1B | -0.062 | -1.068 | 0.287 | 0.991 | -0.233 |
| FGF.21 | IL1B | 0.164 | 1.027 | 0.305 | 0.991 | 0.131 |
| LAP.TGF.beta.1 | IL1B | 0.069 | 1.020 | 0.309 | 0.991 | 0.074 |
| IL8 | IL1B | 0.128 | 1.014 | 0.312 | 0.991 | 0.084 |
| IL13 | IL1B | 0.042 | 0.999 | 0.319 | 0.991 | 0.201 |
| MCP.4 | IL1B | 0.122 | 0.999 | 0.319 | 0.991 | 0.078 |
| TGF.alpha | IL1B | -0.089 | -0.985 | 0.326 | 0.991 | -0.229 |
| CSF.1 | IL1B | -0.057 | -0.984 | 0.326 | 0.991 | -0.235 |
| CDCP1 | IL1B | 0.062 | 0.935 | 0.351 | 0.991 | 0.132 |
| SIRT2 | IL1B | 0.030 | 0.903 | 0.367 | 0.991 | -0.071 |
| Beta.NGF | IL1B | -0.014 | -0.861 | 0.390 | 0.991 | -0.154 |
| SCF | IL1B | 0.061 | 0.840 | 0.402 | 0.991 | 0.082 |
| CXCL11 | IL1B | 0.109 | 0.828 | 0.409 | 0.991 | 0.073 |
| TNFSF14 | IL1B | -0.082 | -0.803 | 0.423 | 0.991 | -0.172 |
| GDNF | IL1B | 0.046 | 0.793 | 0.429 | 0.991 | 0.059 |
| IL.18R1 | IL1B | -0.084 | -0.760 | 0.448 | 0.991 | -0.190 |
| CX3CL1 | IL1B | 0.050 | 0.759 | 0.448 | 0.991 | 0.109 |
| IL.17A | IL1B | 0.038 | 0.719 | 0.473 | 0.991 | 0.121 |
| IL4 | IL1B | 0.032 | 0.675 | 0.501 | 0.991 | 0.130 |
| OSM | IL1B | 0.071 | 0.641 | 0.522 | 0.991 | 0.065 |
| MMP.10 | IL1B | 0.055 | 0.635 | 0.526 | 0.991 | -0.014 |
| EN.RAGE | IL1B | -0.056 | -0.629 | 0.530 | 0.991 | -0.014 |
| TWEAK | IL1B | 0.041 | 0.612 | 0.541 | 0.991 | 0.040 |
| AXIN1 | IL1B | 0.054 | 0.604 | 0.546 | 0.991 | -0.001 |
| MMP.1 | IL1B | 0.075 | 0.520 | 0.604 | 0.991 | 0.062 |
| MCP.1 | IL1B | 0.071 | 0.504 | 0.614 | 0.991 | 0.087 |
| FGF.23 | IL1B | -0.030 | -0.499 | 0.618 | 0.991 | -0.065 |
| NRTN | IL1B | -0.018 | -0.494 | 0.622 | 0.991 | -0.066 |
| IL.2RB | IL1B | 0.018 | 0.446 | 0.656 | 0.991 | 0.097 |
| CCL19 | IL1B | -0.033 | -0.442 | 0.659 | 0.991 | -0.136 |
| CD5 | IL1B | -0.051 | -0.423 | 0.673 | 0.991 | -0.194 |
| uPA | IL1B | -0.034 | -0.415 | 0.679 | 0.991 | -0.092 |
| IFN.gamma | IL1B | -0.071 | -0.408 | 0.683 | 0.991 | -0.133 |
| IL5 | IL1B | 0.022 | 0.405 | 0.686 | 0.991 | 0.039 |
| PD.L1 | IL1B | -0.029 | -0.384 | 0.702 | 0.991 | -0.178 |
| IL.12B | IL1B | -0.034 | -0.375 | 0.708 | 0.991 | -0.059 |
| IL33 | IL1B | -0.016 | -0.363 | 0.717 | 0.991 | -0.047 |
| CCL11 | IL1B | -0.047 | -0.354 | 0.724 | 0.991 | -0.054 |
| CXCL10 | IL1B | 0.038 | 0.351 | 0.726 | 0.991 | -0.022 |
| ADA | IL1B | -0.015 | -0.351 | 0.726 | 0.991 | -0.122 |
| MCP.3 | IL1B | -0.036 | -0.335 | 0.738 | 0.991 | -0.067 |
| IL18 | IL1B | -0.032 | -0.312 | 0.755 | 0.991 | 0.030 |
| IL.1.alpha | IL1B | 0.058 | 0.303 | 0.763 | 0.991 | 0.010 |
| CXCL9 | IL1B | 0.024 | 0.300 | 0.764 | 0.991 | -0.044 |
| CST5 | IL1B | 0.042 | 0.296 | 0.768 | 0.991 | -0.017 |
| IL10 | IL1B | 0.014 | 0.273 | 0.785 | 0.991 | 0.062 |
| CCL20 | IL1B | 0.032 | 0.265 | 0.792 | 0.991 | -0.054 |
| CASP.8 | IL1B | -0.016 | -0.262 | 0.794 | 0.991 | -0.061 |
| CCL4 | IL1B | 0.022 | 0.249 | 0.804 | 0.991 | -0.039 |
| CD40 | IL1B | 0.023 | 0.237 | 0.813 | 0.991 | -0.090 |
| VEGFA | IL1B | 0.020 | 0.216 | 0.829 | 0.991 | -0.033 |
| IL.22.RA1 | IL1B | 0.014 | 0.165 | 0.869 | 0.991 | 0.096 |
| CD6 | IL1B | 0.017 | 0.151 | 0.880 | 0.991 | -0.076 |
| NT.3 | IL1B | -0.008 | -0.142 | 0.887 | 0.991 | -0.031 |
| HGF | IL1B | -0.015 | -0.138 | 0.891 | 0.991 | -0.069 |
| CD244 | IL1B | 0.012 | 0.135 | 0.893 | 0.991 | -0.152 |
| IL.20RA | IL1B | -0.006 | -0.132 | 0.895 | 0.991 | -0.026 |
| OPG | IL1B | 0.008 | 0.119 | 0.905 | 0.991 | -0.108 |
| IL.10RA | IL1B | 0.004 | 0.116 | 0.908 | 0.991 | 0.001 |
| X4E.BP1 | IL1B | -0.003 | -0.109 | 0.913 | 0.991 | -0.033 |
| IL2 | IL1B | -0.005 | -0.104 | 0.917 | 0.991 | 0.002 |
| TNF | IL1B | -0.008 | -0.093 | 0.926 | 0.991 | -0.036 |
| LIF | IL1B | 0.003 | 0.090 | 0.929 | 0.991 | 0.050 |
| ARTN | IL1B | 0.004 | 0.071 | 0.943 | 0.991 | 0.039 |
| CD8A | IL1B | 0.005 | 0.051 | 0.960 | 0.991 | -0.119 |
| TRANCE | IL1B | -0.002 | -0.029 | 0.977 | 0.991 | -0.044 |
| SLAMF1 | IL1B | 0.002 | 0.028 | 0.978 | 0.991 | 0.004 |
| TRAIL | IL1B | 0.002 | 0.027 | 0.979 | 0.991 | -0.115 |
| FGF.19 | IL1B | -0.001 | -0.013 | 0.990 | 0.991 | -0.040 |
| ST1A1 | IL1B | 0.001 | 0.011 | 0.991 | 0.991 | -0.018 |
| CST5 | IL17A | -0.006 | -0.044 | 0.965 | 0.997 | -0.062 |
| CASP.8 | IL17A | 0.002 | 0.030 | 0.976 | 0.998 | 0.012 |
| CSF.1 | IL17A | 0.000 | -0.003 | 0.998 | 0.999 | -0.063 |
| SCF | IL17A | 0.000 | 0.001 | 0.999 | 0.999 | -0.058 |

LogFC between groups and p values were calculated with limma package in R. FDR correction method is used to calculate adjusted p-values.

Significance is defined as FDR-adjusted p-value <0.05 (*)

Cohen’s d is reported to indicate observed effect size for the respective comparison.

Models were adjusted for maternal inflammatory marker batch, child age at DBS collection, time between maternal sample date and child birthdate, and the first two principal components (PC1 and PC2) of the collinear neonatal covariates.

# **Supplementary Table 16**. Differential expression results for all neonatal inflammatory markers between high and low maternal inflammation with restricted temporal proximity (sensitivity analysis; n=105), ordered by adjusted p-value

| **Neonatal  inflammatory marker** | **High Maternal Inflammatory Marker** | **logFC** | **t-statistic** | **p-value** | **Adjusted p-value** | **Cohen's d** |
| --- | --- | --- | --- | --- | --- | --- |
| SCF | IL1B | 0.319 | 2.822 | 0.006 | 0.482 | 0.488 |
| CX3CL1 | IL1B | 0.267 | 2.607 | 0.011 | 0.482 | 0.580 |
| FGF.21 | IL1B | 0.596 | 2.456 | 0.016 | 0.482 | 0.606 |
| Flt3L | IL-17A | -0.264 | -2.677 | 0.009 | 0.649 | -0.609 |
| NRTN | IL-17A | 0.114 | 2.498 | 0.014 | 0.649 | 0.504 |
| TRANCE | IL-17A | -0.272 | -2.294 | 0.024 | 0.724 | -0.498 |
| IL10 | IL-17A | 0.164 | 2.136 | 0.035 | 0.724 | 0.370 |
| TNFB | IL-17A | -0.148 | -2.085 | 0.040 | 0.724 | -0.498 |
| CXCL10 | IL-17A | -0.253 | -1.826 | 0.071 | 0.724 | -0.455 |
| IFN.gamma | IL-17A | -0.326 | -1.795 | 0.076 | 0.724 | -0.407 |
| EN.RAGE | IL-17A | 0.194 | 1.695 | 0.093 | 0.724 | 0.238 |
| SCF | IL-17A | -0.184 | -1.647 | 0.103 | 0.724 | -0.393 |
| IL.20RA | IL-17A | 0.096 | 1.637 | 0.105 | 0.724 | 0.168 |
| NT.3 | IL-17A | 0.118 | 1.632 | 0.106 | 0.724 | 0.241 |
| FGF.21 | IL-17A | 0.368 | 1.544 | 0.126 | 0.724 | 0.300 |
| CXCL11 | IL-17A | -0.335 | -1.537 | 0.127 | 0.724 | -0.403 |
| IL.2RB | IL-17A | -0.084 | -1.530 | 0.129 | 0.724 | -0.310 |
| IL.17C | IL-17A | -0.251 | -1.527 | 0.130 | 0.724 | -0.288 |
| FGF.19 | IL-17A | -0.238 | -1.494 | 0.138 | 0.724 | -0.346 |
| ST1A1 | IL-17A | 0.148 | 1.394 | 0.166 | 0.724 | 0.217 |
| GDNF | IL-17A | 0.113 | 1.387 | 0.169 | 0.724 | 0.263 |
| IL.20 | IL-17A | -0.074 | -1.385 | 0.169 | 0.724 | -0.317 |
| TNFRSF9 | IL-17A | -0.169 | -1.374 | 0.172 | 0.724 | -0.352 |
| IL4 | IL-17A | -0.112 | -1.298 | 0.197 | 0.724 | -0.379 |
| IL2 | IL-17A | 0.083 | 1.297 | 0.197 | 0.724 | 0.294 |
| CCL11 | IL-17A | -0.205 | -1.295 | 0.198 | 0.724 | -0.195 |
| MCP.1 | IL-17A | -0.228 | -1.268 | 0.208 | 0.724 | -0.239 |
| IL18 | IL-17A | -0.158 | -1.258 | 0.211 | 0.724 | -0.248 |
| TSLP | IL-17A | -0.093 | -1.254 | 0.213 | 0.724 | -0.180 |
| CD8A | IL-17A | -0.180 | -1.249 | 0.214 | 0.724 | -0.262 |
| CXCL9 | IL-17A | -0.132 | -1.233 | 0.220 | 0.724 | -0.427 |
| IL5 | IL-17A | 0.084 | 1.141 | 0.257 | 0.789 | 0.116 |
| ARTN | IL-17A | -0.088 | -1.140 | 0.257 | 0.789 | -0.290 |
| SLAMF1 | IL1B | 0.145 | 2.044 | 0.043 | 0.811 | 0.480 |
| MMP.10 | IL1B | 0.272 | 2.039 | 0.044 | 0.811 | 0.464 |
| FGF.5 | IL-17A | -0.094 | -1.057 | 0.293 | 0.839 | -0.249 |
| X4E.BP1 | IL-17A | 0.046 | 1.031 | 0.305 | 0.839 | 0.329 |
| CCL25 | IL-17A | -0.106 | -1.015 | 0.313 | 0.839 | -0.189 |
| IL7 | IL-17A | -0.068 | -0.984 | 0.327 | 0.839 | -0.246 |
| LIF.R | IL-17A | -0.036 | -0.947 | 0.346 | 0.839 | -0.244 |
| IL.12B | IL-17A | -0.110 | -0.931 | 0.354 | 0.839 | -0.191 |
| CCL3 | IL-17A | 0.195 | 0.920 | 0.360 | 0.839 | 0.184 |
| IL.17A | IL-17A | 0.056 | 0.840 | 0.403 | 0.839 | 0.203 |
| SLAMF1 | IL-17A | 0.058 | 0.836 | 0.405 | 0.839 | 0.139 |
| CCL20 | IL-17A | 0.131 | 0.827 | 0.410 | 0.839 | 0.113 |
| CXCL6 | IL-17A | 0.117 | 0.823 | 0.412 | 0.839 | 0.036 |
| MMP.10 | IL-17A | 0.102 | 0.781 | 0.437 | 0.839 | 0.211 |
| IL33 | IL-17A | 0.052 | 0.779 | 0.438 | 0.839 | 0.179 |
| MCP.4 | IL-17A | -0.140 | -0.776 | 0.440 | 0.839 | -0.233 |
| TGF.alpha | IL-17A | 0.082 | 0.770 | 0.443 | 0.839 | -0.003 |
| CD6 | IL-17A | -0.123 | -0.751 | 0.454 | 0.839 | -0.193 |
| IL.10RA | IL-17A | -0.035 | -0.750 | 0.455 | 0.839 | -0.198 |
| OSM | IL-17A | -0.102 | -0.749 | 0.456 | 0.839 | -0.368 |
| VEGFA | IL-17A | -0.091 | -0.718 | 0.474 | 0.839 | -0.278 |
| CD40 | IL-17A | -0.097 | -0.714 | 0.477 | 0.839 | -0.182 |
| DNER | IL-17A | -0.061 | -0.712 | 0.478 | 0.839 | -0.097 |
| CD5 | IL-17A | -0.125 | -0.703 | 0.484 | 0.839 | -0.157 |
| MCP.2 | IL-17A | -0.110 | -0.684 | 0.495 | 0.839 | -0.219 |
| CD244 | IL-17A | -0.081 | -0.677 | 0.500 | 0.839 | -0.215 |
| CST5 | IL-17A | -0.101 | -0.673 | 0.503 | 0.839 | -0.246 |
| AXIN1 | IL-17A | -0.076 | -0.655 | 0.514 | 0.839 | -0.153 |
| CASP.8 | IL-17A | -0.054 | -0.626 | 0.533 | 0.839 | -0.250 |
| CCL4 | IL-17A | -0.086 | -0.621 | 0.536 | 0.839 | -0.163 |
| CCL19 | IL-17A | -0.062 | -0.606 | 0.546 | 0.839 | -0.178 |
| LIF | IL-17A | -0.036 | -0.602 | 0.549 | 0.839 | -0.188 |
| OPG | IL-17A | 0.056 | 0.591 | 0.556 | 0.839 | 0.161 |
| ADA | IL-17A | -0.037 | -0.571 | 0.569 | 0.845 | -0.144 |
| CXCL9 | IL1B | -0.201 | -1.835 | 0.069 | 0.854 | -0.307 |
| GDNF | IL1B | -0.151 | -1.790 | 0.076 | 0.854 | -0.401 |
| OPG | IL1B | 0.167 | 1.712 | 0.090 | 0.854 | 0.188 |
| ST1A1 | IL1B | -0.181 | -1.652 | 0.102 | 0.854 | -0.323 |
| TRANCE | IL1B | -0.203 | -1.631 | 0.106 | 0.854 | -0.453 |
| MCP.2 | IL1B | -0.266 | -1.613 | 0.110 | 0.854 | -0.396 |
| IL.18R1 | IL1B | 0.269 | 1.560 | 0.122 | 0.854 | 0.336 |
| CCL25 | IL1B | 0.163 | 1.520 | 0.132 | 0.854 | -0.024 |
| NRTN | IL1B | -0.070 | -1.450 | 0.150 | 0.854 | -0.373 |
| IL8 | IL1B | 0.235 | 1.432 | 0.155 | 0.854 | 0.390 |
| IL5 | IL1B | -0.109 | -1.426 | 0.157 | 0.854 | -0.327 |
| MCP.3 | IL1B | 0.265 | 1.410 | 0.162 | 0.854 | 0.479 |
| IL.10RA | IL1B | 0.066 | 1.365 | 0.175 | 0.854 | 0.302 |
| IL18 | IL1B | 0.169 | 1.302 | 0.196 | 0.854 | 0.308 |
| CCL20 | IL1B | -0.209 | -1.274 | 0.205 | 0.854 | -0.319 |
| DNER | IL1B | 0.111 | 1.268 | 0.208 | 0.854 | 0.120 |
| IL.10RB | IL1B | 0.114 | 1.256 | 0.212 | 0.854 | 0.154 |
| CD40 | IL1B | 0.175 | 1.252 | 0.214 | 0.854 | 0.077 |
| IL.10RB | IL-17A | -0.045 | -0.503 | 0.616 | 0.858 | -0.161 |
| MMP.1 | IL-17A | -0.107 | -0.497 | 0.620 | 0.858 | -0.177 |
| IL6 | IL-17A | -0.091 | -0.494 | 0.622 | 0.858 | -0.193 |
| TRAIL | IL-17A | -0.054 | -0.490 | 0.625 | 0.858 | -0.270 |
| CCL23 | IL-17A | -0.068 | -0.469 | 0.640 | 0.858 | -0.153 |
| IL.22.RA1 | IL-17A | 0.057 | 0.439 | 0.662 | 0.858 | 0.091 |
| SIRT2 | IL-17A | 0.023 | 0.436 | 0.664 | 0.858 | 0.027 |
| CCL28 | IL-17A | 0.018 | 0.421 | 0.675 | 0.858 | 0.134 |
| IL.24 | IL-17A | 0.053 | 0.418 | 0.677 | 0.858 | 0.040 |
| CXCL5 | IL-17A | -0.082 | -0.409 | 0.684 | 0.858 | -0.130 |
| CX3CL1 | IL-17A | -0.041 | -0.401 | 0.689 | 0.858 | -0.061 |
| uPA | IL-17A | 0.049 | 0.400 | 0.690 | 0.858 | -0.083 |
| HGF | IL-17A | -0.050 | -0.361 | 0.719 | 0.881 | -0.286 |
| CXCL1 | IL1B | 0.130 | 1.154 | 0.251 | 0.887 | 0.240 |
| CD6 | IL1B | 0.193 | 1.148 | 0.254 | 0.887 | 0.071 |
| IL.17A | IL1B | 0.076 | 1.100 | 0.274 | 0.887 | 0.223 |
| IL4 | IL1B | -0.098 | -1.088 | 0.279 | 0.887 | -0.313 |
| X4E.BP1 | IL1B | 0.049 | 1.077 | 0.284 | 0.887 | 0.161 |
| TWEAK | IL1B | 0.094 | 1.073 | 0.286 | 0.887 | 0.219 |
| CCL23 | IL1B | 0.157 | 1.045 | 0.298 | 0.887 | 0.304 |
| OSM | IL1B | 0.142 | 1.004 | 0.318 | 0.887 | 0.334 |
| TGF.alpha | IL1B | 0.110 | 0.995 | 0.322 | 0.887 | 0.216 |
| MCP.1 | IL1B | 0.186 | 0.993 | 0.323 | 0.887 | 0.422 |
| CD5 | IL1B | 0.180 | 0.981 | 0.329 | 0.887 | 0.018 |
| CST5 | IL1B | 0.146 | 0.942 | 0.348 | 0.887 | 0.006 |
| TNFRSF9 | IL1B | 0.119 | 0.927 | 0.356 | 0.887 | 0.196 |
| SIRT2 | IL1B | -0.050 | -0.926 | 0.357 | 0.887 | -0.365 |
| TNF | IL-17A | -0.042 | -0.291 | 0.772 | 0.894 | -0.189 |
| CDCP1 | IL-17A | -0.019 | -0.289 | 0.773 | 0.894 | -0.006 |
| LAP.TGF.beta.1 | IL-17A | 0.028 | 0.275 | 0.784 | 0.894 | 0.057 |
| FGF.23 | IL-17A | -0.035 | -0.268 | 0.789 | 0.894 | -0.099 |
| IL.15RA | IL-17A | 0.019 | 0.260 | 0.795 | 0.894 | 0.004 |
| IL.1.alpha | IL-17A | -0.085 | -0.247 | 0.805 | 0.894 | 0.010 |
| IL13 | IL-17A | -0.013 | -0.245 | 0.807 | 0.894 | -0.130 |
| TWEAK | IL-17A | -0.021 | -0.245 | 0.807 | 0.894 | -0.128 |
| HGF | IL1B | 0.129 | 0.897 | 0.372 | 0.901 | 0.208 |
| IL2 | IL1B | 0.058 | 0.874 | 0.384 | 0.906 | 0.260 |
| CXCL1 | IL-17A | 0.023 | 0.206 | 0.837 | 0.911 | -0.136 |
| CSF.1 | IL-17A | -0.017 | -0.200 | 0.842 | 0.911 | -0.085 |
| TNFSF14 | IL-17A | -0.026 | -0.185 | 0.854 | 0.913 | -0.222 |
| PD.L1 | IL-17A | -0.018 | -0.171 | 0.864 | 0.914 | -0.056 |
| IL.18R1 | IL-17A | 0.025 | 0.151 | 0.880 | 0.920 | -0.073 |
| PD.L1 | IL1B | 0.087 | 0.816 | 0.417 | 0.939 | 0.114 |
| IL.12B | IL1B | 0.098 | 0.804 | 0.423 | 0.939 | 0.385 |
| CCL3 | IL1B | -0.171 | -0.780 | 0.437 | 0.939 | -0.196 |
| Beta.NGF | IL1B | 0.021 | 0.754 | 0.453 | 0.939 | 0.147 |
| LIF | IL1B | 0.047 | 0.753 | 0.453 | 0.939 | 0.219 |
| uPA | IL1B | 0.093 | 0.735 | 0.464 | 0.939 | 0.185 |
| IL7 | IL1B | 0.052 | 0.726 | 0.470 | 0.939 | 0.137 |
| CD8A | CRP | -0.301 | -2.173 | 0.032 | 0.941 | -0.346 |
| CCL11 | CRP | -0.306 | -1.997 | 0.049 | 0.941 | -0.392 |
| FGF.23 | CRP | 0.240 | 1.891 | 0.061 | 0.941 | 0.405 |
| IL4 | CRP | -0.153 | -1.827 | 0.071 | 0.941 | -0.374 |
| IL8 | CRP | 0.274 | 1.774 | 0.079 | 0.941 | 0.315 |
| CD5 | CRP | -0.293 | -1.711 | 0.090 | 0.941 | -0.260 |
| Flt3L | CRP | -0.166 | -1.689 | 0.094 | 0.941 | -0.439 |
| CD6 | CRP | -0.251 | -1.591 | 0.115 | 0.941 | -0.244 |
| CX3CL1 | CRP | -0.151 | -1.533 | 0.128 | 0.941 | -0.331 |
| TRANCE | CRP | -0.180 | -1.529 | 0.129 | 0.941 | -0.289 |
| TSLP | CRP | -0.110 | -1.528 | 0.130 | 0.941 | -0.383 |
| CST5 | CRP | -0.221 | -1.523 | 0.131 | 0.941 | -0.220 |
| CCL23 | CRP | -0.213 | -1.514 | 0.133 | 0.941 | -0.309 |
| AXIN1 | IL1B | 0.078 | 0.646 | 0.520 | 0.955 | 0.030 |
| CXCL6 | IL1B | 0.085 | 0.575 | 0.567 | 0.955 | -0.021 |
| LIF.R | IL1B | 0.022 | 0.551 | 0.583 | 0.955 | -0.030 |
| CXCL10 | IL1B | -0.080 | -0.549 | 0.584 | 0.955 | 0.018 |
| CASP.8 | IL1B | 0.048 | 0.539 | 0.591 | 0.955 | 0.108 |
| MMP.1 | IL1B | 0.117 | 0.527 | 0.599 | 0.955 | -0.001 |
| IL33 | IL1B | 0.036 | 0.517 | 0.606 | 0.955 | 0.098 |
| CD8A | IL1B | 0.077 | 0.513 | 0.609 | 0.955 | -0.080 |
| IL.20RA | IL1B | -0.030 | -0.494 | 0.622 | 0.955 | -0.174 |
| EN.RAGE | IL1B | -0.059 | -0.488 | 0.627 | 0.955 | 0.001 |
| ADA | IL1B | -0.033 | -0.484 | 0.629 | 0.955 | -0.233 |
| CSF.1 | IL1B | 0.041 | 0.460 | 0.646 | 0.955 | 0.079 |
| CCL28 | IL1B | -0.020 | -0.460 | 0.647 | 0.955 | -0.103 |
| CCL19 | IL1B | 0.049 | 0.455 | 0.650 | 0.955 | 0.175 |
| TRAIL | IL1B | 0.050 | 0.438 | 0.663 | 0.955 | 0.120 |
| Flt3L | IL1B | 0.043 | 0.405 | 0.686 | 0.955 | 0.195 |
| FGF.5 | IL1B | 0.038 | 0.404 | 0.687 | 0.955 | 0.152 |
| IL10 | IL1B | 0.032 | 0.389 | 0.698 | 0.955 | 0.020 |
| IL.15RA | IL1B | 0.028 | 0.369 | 0.713 | 0.955 | -0.017 |
| IL.24 | IL1B | -0.047 | -0.356 | 0.722 | 0.955 | -0.121 |
| CXCL5 | IL1B | 0.072 | 0.349 | 0.728 | 0.955 | 0.067 |
| LAP.TGF.beta.1 | IL1B | -0.037 | -0.347 | 0.729 | 0.955 | -0.222 |
| FGF.19 | IL1B | 0.054 | 0.325 | 0.746 | 0.955 | -0.047 |
| IFN.gamma | IL1B | -0.058 | -0.303 | 0.762 | 0.955 | 0.101 |
| CXCL11 | IL1B | -0.066 | -0.290 | 0.772 | 0.955 | -0.035 |
| IL6 | IL1B | 0.054 | 0.281 | 0.779 | 0.955 | 0.042 |
| VEGFA | IL1B | 0.036 | 0.273 | 0.786 | 0.955 | 0.041 |
| STAMBP | IL1B | 0.008 | 0.237 | 0.813 | 0.955 | 0.020 |
| IL.20 | IL1B | 0.013 | 0.237 | 0.813 | 0.955 | 0.146 |
| TNF | IL1B | -0.034 | -0.223 | 0.824 | 0.955 | -0.121 |
| CDCP1 | IL1B | -0.014 | -0.207 | 0.837 | 0.955 | -0.138 |
| TNFSF14 | IL1B | -0.028 | -0.199 | 0.843 | 0.955 | -0.026 |
| IL.22.RA1 | IL1B | -0.026 | -0.196 | 0.845 | 0.955 | -0.061 |
| IL13 | IL1B | -0.011 | -0.187 | 0.852 | 0.955 | -0.140 |
| CCL4 | IL1B | -0.026 | -0.178 | 0.859 | 0.955 | 0.054 |
| TSLP | IL1B | 0.013 | 0.164 | 0.870 | 0.955 | 0.125 |
| IL.17C | IL1B | 0.028 | 0.163 | 0.871 | 0.955 | 0.323 |
| CD244 | IL1B | 0.020 | 0.161 | 0.872 | 0.955 | -0.201 |
| CCL11 | IL1B | 0.024 | 0.143 | 0.887 | 0.955 | 0.180 |
| IL.2RB | IL1B | -0.007 | -0.122 | 0.903 | 0.955 | 0.019 |
| TNFB | IL1B | 0.009 | 0.122 | 0.903 | 0.955 | -0.207 |
| Beta.NGF | IL-17A | 0.002 | 0.073 | 0.942 | 0.973 | 0.033 |
| ARTN | IL1B | 0.007 | 0.085 | 0.932 | 0.974 | 0.062 |
| IL8 | IL-17A | -0.007 | -0.041 | 0.967 | 0.989 | -0.118 |
| IL.20RA | IL-6 | 0.126 | 2.245 | 0.027 | 0.990 | 0.491 |
| IL.17C | IL-6 | 0.345 | 2.198 | 0.030 | 0.990 | 0.534 |
| CSF.1 | IL-6 | 0.138 | 1.682 | 0.096 | 0.990 | 0.401 |
| CCL20 | IL-6 | 0.251 | 1.655 | 0.101 | 0.990 | 0.372 |
| CCL19 | IL-6 | 0.161 | 1.643 | 0.103 | 0.990 | 0.399 |
| ARTN | IL-6 | -0.109 | -1.475 | 0.143 | 0.990 | -0.344 |
| CX3CL1 | IL-6 | 0.137 | 1.409 | 0.162 | 0.990 | 0.344 |
| MCP.3 | IL-6 | 0.243 | 1.389 | 0.168 | 0.990 | 0.367 |
| CCL11 | IL-6 | 0.206 | 1.350 | 0.180 | 0.990 | 0.361 |
| IL33 | IL-6 | 0.086 | 1.344 | 0.182 | 0.990 | 0.320 |
| MCP.4 | IL-6 | 0.223 | 1.289 | 0.200 | 0.990 | 0.318 |
| IL2 | IL-6 | 0.078 | 1.256 | 0.212 | 0.990 | 0.328 |
| GDNF | IL-6 | 0.098 | 1.241 | 0.218 | 0.990 | 0.291 |
| IL7 | IL-6 | -0.083 | -1.235 | 0.220 | 0.990 | -0.269 |
| MCP.1 | IL-6 | 0.202 | 1.158 | 0.250 | 0.990 | 0.326 |
| Flt3L | IL-6 | 0.110 | 1.123 | 0.264 | 0.990 | 0.264 |
| CST5 | IL-6 | 0.160 | 1.113 | 0.268 | 0.990 | 0.211 |
| ADA | IL-6 | 0.070 | 1.113 | 0.268 | 0.990 | 0.248 |
| NT.3 | IL-6 | 0.078 | 1.108 | 0.270 | 0.990 | 0.262 |
| LIF | IL-6 | 0.062 | 1.075 | 0.285 | 0.990 | 0.264 |
| AXIN1 | IL-6 | 0.119 | 1.063 | 0.290 | 0.990 | 0.238 |
| CD5 | IL-6 | 0.180 | 1.052 | 0.295 | 0.990 | 0.216 |
| IL.20 | IL-6 | -0.053 | -1.013 | 0.314 | 0.990 | -0.191 |
| X4E.BP1 | IL-6 | -0.042 | -0.997 | 0.321 | 0.990 | -0.254 |
| FGF.19 | IL-6 | -0.149 | -0.965 | 0.337 | 0.990 | -0.253 |
| IL4 | IL-6 | 0.079 | 0.945 | 0.347 | 0.990 | 0.198 |
| SCF | IL-6 | 0.102 | 0.935 | 0.352 | 0.990 | 0.174 |
| ST1A1 | IL-6 | -0.093 | -0.900 | 0.370 | 0.990 | -0.175 |
| IL18 | IL-6 | 0.100 | 0.825 | 0.411 | 0.990 | 0.189 |
| CD6 | IL-6 | 0.126 | 0.800 | 0.425 | 0.990 | 0.162 |
| DNER | IL-6 | -0.065 | -0.791 | 0.431 | 0.990 | -0.200 |
| CD40 | IL-6 | 0.100 | 0.767 | 0.445 | 0.990 | 0.151 |
| CCL28 | IL-6 | 0.031 | 0.749 | 0.455 | 0.990 | 0.205 |
| IL.10RA | IL-6 | -0.034 | -0.743 | 0.459 | 0.990 | -0.175 |
| CD8A | IL-6 | 0.103 | 0.735 | 0.464 | 0.990 | 0.154 |
| STAMBP | IL-6 | 0.023 | 0.730 | 0.467 | 0.990 | 0.192 |
| PD.L1 | IL-6 | 0.071 | 0.721 | 0.473 | 0.990 | 0.164 |
| TSLP | IL-6 | -0.051 | -0.716 | 0.476 | 0.990 | -0.144 |
| IL8 | IL-6 | 0.105 | 0.682 | 0.497 | 0.990 | 0.195 |
| SIRT2 | IL-6 | 0.032 | 0.640 | 0.524 | 0.990 | 0.125 |
| TNFRSF9 | IL-6 | 0.073 | 0.608 | 0.545 | 0.990 | 0.167 |
| IL.12B | IL-6 | 0.065 | 0.573 | 0.568 | 0.990 | 0.183 |
| LIF.R | IL-6 | 0.020 | 0.554 | 0.581 | 0.990 | 0.100 |
| CCL3 | IL-6 | -0.113 | -0.551 | 0.583 | 0.990 | -0.116 |
| TNF | IL-6 | -0.077 | -0.547 | 0.586 | 0.990 | -0.119 |
| OSM | IL-6 | 0.072 | 0.544 | 0.587 | 0.990 | 0.161 |
| TWEAK | IL-6 | -0.044 | -0.535 | 0.594 | 0.990 | -0.134 |
| TNFSF14 | IL-6 | -0.071 | -0.534 | 0.594 | 0.990 | -0.102 |
| TRANCE | IL-6 | 0.061 | 0.520 | 0.604 | 0.990 | 0.101 |
| IL.17A | IL-6 | 0.033 | 0.513 | 0.609 | 0.990 | 0.120 |
| IL10 | IL-6 | 0.039 | 0.513 | 0.609 | 0.990 | 0.059 |
| CCL25 | IL-6 | 0.052 | 0.511 | 0.610 | 0.990 | 0.047 |
| CXCL6 | IL-6 | 0.067 | 0.485 | 0.629 | 0.990 | 0.089 |
| FGF.5 | IL-6 | -0.041 | -0.470 | 0.640 | 0.990 | -0.092 |
| IL6 | IL-6 | 0.083 | 0.470 | 0.640 | 0.990 | 0.063 |
| LAP.TGF.beta.1 | IL-6 | 0.046 | 0.465 | 0.643 | 0.990 | 0.076 |
| IL.1.alpha | IL-6 | 0.151 | 0.456 | 0.649 | 0.990 | 0.166 |
| IL.22.RA1 | IL-6 | -0.057 | -0.454 | 0.651 | 0.990 | -0.136 |
| SLAMF1 | IL-6 | 0.030 | 0.442 | 0.659 | 0.990 | 0.149 |
| Beta.NGF | IL-6 | -0.011 | -0.422 | 0.674 | 0.990 | -0.106 |
| NRTN | IL-6 | 0.019 | 0.409 | 0.684 | 0.990 | 0.078 |
| IL.10RB | IL-6 | 0.035 | 0.406 | 0.685 | 0.990 | 0.068 |
| CASP.8 | IL-6 | -0.033 | -0.401 | 0.689 | 0.990 | -0.092 |
| CXCL10 | IL-6 | -0.054 | -0.397 | 0.692 | 0.990 | -0.045 |
| MCP.2 | IL-6 | -0.059 | -0.377 | 0.707 | 0.990 | -0.106 |
| CXCL9 | IL-6 | -0.038 | -0.371 | 0.712 | 0.990 | -0.079 |
| CXCL1 | IL-6 | 0.038 | 0.356 | 0.723 | 0.990 | 0.102 |
| EN.RAGE | IL-6 | -0.036 | -0.323 | 0.748 | 0.990 | -0.062 |
| MMP.1 | IL-6 | -0.064 | -0.310 | 0.758 | 0.990 | -0.090 |
| TGF.alpha | IL-6 | -0.032 | -0.307 | 0.760 | 0.990 | -0.040 |
| IL.18R1 | IL-6 | 0.046 | 0.283 | 0.778 | 0.990 | 0.096 |
| IL.15RA | IL-6 | 0.019 | 0.271 | 0.787 | 0.990 | 0.040 |
| OPG | IL-6 | -0.022 | -0.241 | 0.810 | 0.990 | -0.071 |
| FGF.23 | IL-6 | -0.030 | -0.232 | 0.817 | 0.990 | -0.056 |
| VEGFA | IL-6 | -0.029 | -0.232 | 0.817 | 0.990 | 0.009 |
| uPA | IL-6 | 0.026 | 0.222 | 0.825 | 0.990 | 0.081 |
| IL5 | IL-6 | -0.015 | -0.206 | 0.837 | 0.990 | -0.046 |
| FGF.21 | IL-6 | 0.038 | 0.164 | 0.870 | 0.990 | 0.088 |
| IL13 | IL-6 | -0.008 | -0.145 | 0.885 | 0.990 | -0.046 |
| CDCP1 | IL-6 | -0.009 | -0.144 | 0.886 | 0.990 | -0.021 |
| CCL23 | IL-6 | 0.015 | 0.108 | 0.914 | 0.990 | 0.085 |
| IL.2RB | IL-6 | -0.006 | -0.108 | 0.914 | 0.990 | 0.022 |
| IL.24 | IL-6 | 0.013 | 0.106 | 0.916 | 0.990 | -0.002 |
| IFN.gamma | IL-6 | 0.017 | 0.097 | 0.923 | 0.990 | 0.078 |
| CD244 | IL-6 | 0.011 | 0.095 | 0.924 | 0.990 | -0.014 |
| HGF | IL-6 | 0.011 | 0.084 | 0.933 | 0.990 | 0.046 |
| CCL4 | IL-6 | 0.010 | 0.074 | 0.941 | 0.990 | 0.047 |
| CXCL11 | IL-6 | -0.008 | -0.038 | 0.970 | 0.990 | -0.005 |
| TNFB | IL-6 | -0.002 | -0.034 | 0.973 | 0.990 | -0.062 |
| TRAIL | IL-6 | 0.003 | 0.028 | 0.978 | 0.990 | 0.034 |
| CXCL5 | IL-6 | -0.005 | -0.026 | 0.979 | 0.990 | 0.002 |
| IL.1.alpha | IL1B | 0.019 | 0.052 | 0.959 | 0.991 | 0.257 |
| LIF.R | CRP | -0.052 | -1.415 | 0.160 | 0.993 | -0.292 |
| IL10 | CRP | 0.107 | 1.406 | 0.163 | 0.993 | 0.262 |
| HGF | CRP | -0.181 | -1.339 | 0.184 | 0.993 | -0.281 |
| ARTN | CRP | 0.097 | 1.281 | 0.203 | 0.993 | 0.254 |
| IL.18R1 | CRP | -0.207 | -1.268 | 0.208 | 0.993 | -0.256 |
| VEGFA | CRP | 0.154 | 1.242 | 0.217 | 0.993 | 0.299 |
| CSF.1 | CRP | -0.103 | -1.229 | 0.222 | 0.993 | -0.224 |
| CXCL5 | CRP | 0.218 | 1.126 | 0.263 | 0.993 | 0.208 |
| SLAMF1 | CRP | 0.074 | 1.084 | 0.281 | 0.993 | 0.229 |
| IL6 | CRP | -0.192 | -1.072 | 0.286 | 0.993 | -0.267 |
| TNFRSF9 | CRP | -0.127 | -1.053 | 0.295 | 0.993 | -0.192 |
| FGF.19 | CRP | -0.164 | -1.047 | 0.297 | 0.993 | -0.225 |
| MCP.1 | CRP | -0.182 | -1.030 | 0.306 | 0.993 | -0.246 |
| CCL20 | CRP | 0.150 | 0.970 | 0.334 | 0.993 | 0.280 |
| CCL28 | CRP | -0.037 | -0.888 | 0.377 | 0.993 | -0.184 |
| MCP.4 | CRP | -0.147 | -0.834 | 0.406 | 0.993 | -0.202 |
| IL13 | CRP | -0.044 | -0.821 | 0.414 | 0.993 | -0.148 |
| AXIN1 | CRP | -0.091 | -0.798 | 0.426 | 0.993 | -0.088 |
| CXCL6 | CRP | -0.104 | -0.750 | 0.455 | 0.993 | -0.103 |
| TNFSF14 | CRP | -0.099 | -0.733 | 0.465 | 0.993 | -0.148 |
| LIF | CRP | -0.042 | -0.717 | 0.475 | 0.993 | -0.182 |
| Beta.NGF | CRP | 0.018 | 0.717 | 0.475 | 0.993 | 0.218 |
| IFN.gamma | CRP | -0.123 | -0.683 | 0.496 | 0.993 | -0.177 |
| TNF | CRP | -0.091 | -0.639 | 0.524 | 0.993 | -0.103 |
| X4E.BP1 | CRP | 0.028 | 0.637 | 0.525 | 0.993 | 0.168 |
| IL.20 | CRP | -0.033 | -0.630 | 0.530 | 0.993 | -0.143 |
| IL.22.RA1 | CRP | -0.075 | -0.595 | 0.553 | 0.993 | -0.160 |
| PD.L1 | CRP | -0.056 | -0.562 | 0.576 | 0.993 | -0.113 |
| TWEAK | CRP | -0.046 | -0.556 | 0.580 | 0.993 | -0.162 |
| CXCL10 | CRP | 0.074 | 0.538 | 0.592 | 0.993 | 0.099 |
| IL18 | CRP | -0.060 | -0.488 | 0.627 | 0.993 | -0.085 |
| TRAIL | CRP | -0.050 | -0.468 | 0.641 | 0.993 | -0.095 |
| TNFB | CRP | -0.033 | -0.465 | 0.643 | 0.993 | -0.012 |
| SCF | CRP | -0.049 | -0.444 | 0.658 | 0.993 | -0.101 |
| CCL3 | CRP | -0.091 | -0.437 | 0.663 | 0.993 | -0.057 |
| FGF.21 | CRP | 0.101 | 0.431 | 0.668 | 0.993 | 0.113 |
| CDCP1 | CRP | 0.027 | 0.412 | 0.681 | 0.993 | 0.184 |
| uPA | CRP | -0.049 | -0.409 | 0.683 | 0.993 | -0.084 |
| IL.2RB | CRP | -0.021 | -0.388 | 0.699 | 0.993 | -0.075 |
| MMP.10 | CRP | 0.050 | 0.387 | 0.700 | 0.993 | 0.072 |
| TGF.alpha | CRP | 0.040 | 0.384 | 0.702 | 0.993 | 0.090 |
| LAP.TGF.beta.1 | CRP | -0.037 | -0.372 | 0.711 | 0.993 | -0.003 |
| IL2 | CRP | -0.023 | -0.368 | 0.713 | 0.993 | -0.080 |
| CXCL11 | CRP | -0.077 | -0.358 | 0.721 | 0.993 | -0.102 |
| IL7 | CRP | -0.024 | -0.347 | 0.729 | 0.993 | -0.071 |
| IL.12B | CRP | 0.040 | 0.347 | 0.730 | 0.993 | 0.026 |
| OPG | CRP | -0.032 | -0.346 | 0.730 | 0.993 | -0.024 |
| CCL19 | CRP | -0.035 | -0.346 | 0.730 | 0.993 | -0.036 |
| OSM | CRP | 0.045 | 0.340 | 0.734 | 0.993 | 0.018 |
| CCL25 | CRP | 0.034 | 0.332 | 0.741 | 0.993 | 0.190 |
| CD40 | CRP | -0.041 | -0.311 | 0.757 | 0.993 | 0.015 |
| IL.10RB | CRP | -0.026 | -0.305 | 0.761 | 0.993 | -0.031 |
| IL.1.alpha | CRP | -0.101 | -0.299 | 0.765 | 0.993 | -0.141 |
| FGF.5 | CRP | -0.026 | -0.292 | 0.771 | 0.993 | -0.101 |
| CD244 | CRP | -0.033 | -0.283 | 0.778 | 0.993 | 0.051 |
| CASP.8 | CRP | -0.022 | -0.265 | 0.792 | 0.993 | -0.042 |
| SIRT2 | CRP | -0.013 | -0.258 | 0.797 | 0.993 | 0.028 |
| IL.15RA | CRP | 0.018 | 0.251 | 0.802 | 0.993 | 0.076 |
| IL.17C | CRP | 0.038 | 0.236 | 0.814 | 0.993 | -0.056 |
| MCP.3 | CRP | 0.042 | 0.234 | 0.815 | 0.993 | -0.002 |
| ADA | CRP | 0.015 | 0.233 | 0.817 | 0.993 | 0.136 |
| IL5 | CRP | -0.016 | -0.217 | 0.829 | 0.993 | -0.033 |
| IL.17A | CRP | -0.013 | -0.194 | 0.846 | 0.993 | -0.014 |
| MCP.2 | CRP | 0.026 | 0.164 | 0.870 | 0.993 | 0.053 |
| IL.24 | CRP | 0.018 | 0.144 | 0.886 | 0.993 | 0.040 |
| DNER | CRP | -0.011 | -0.135 | 0.893 | 0.993 | -0.010 |
| IL33 | CRP | 0.008 | 0.121 | 0.904 | 0.993 | 0.060 |
| STAMBP | CRP | -0.004 | -0.114 | 0.909 | 0.993 | 0.022 |
| IL.20RA | CRP | -0.006 | -0.109 | 0.913 | 0.993 | 0.005 |
| CXCL1 | CRP | -0.011 | -0.104 | 0.917 | 0.993 | -0.007 |
| GDNF | CRP | 0.008 | 0.103 | 0.918 | 0.993 | 0.036 |
| EN.RAGE | CRP | -0.010 | -0.092 | 0.927 | 0.993 | -0.097 |
| NRTN | CRP | -0.004 | -0.091 | 0.928 | 0.993 | -0.015 |
| IL.10RA | CRP | 0.002 | 0.047 | 0.963 | 0.994 | 0.018 |
| MMP.1 | CRP | -0.006 | -0.029 | 0.977 | 0.994 | 0.016 |
| ST1A1 | CRP | -0.002 | -0.022 | 0.983 | 0.994 | 0.003 |
| CXCL9 | CRP | -0.002 | -0.016 | 0.987 | 0.994 | -0.037 |
| CCL4 | CRP | -0.002 | -0.012 | 0.991 | 0.994 | -0.046 |
| NT.3 | CRP | -0.001 | -0.007 | 0.994 | 0.994 | 0.032 |
| MCP.4 | IL1B | -0.006 | -0.030 | 0.976 | 0.996 | 0.083 |
| NT.3 | IL1B | 0.001 | 0.012 | 0.990 | 0.996 | -0.039 |
| FGF.23 | IL1B | -0.001 | -0.005 | 0.996 | 0.996 | -0.009 |
| MMP.10 | IL-6 | -0.001 | -0.005 | 0.996 | 0.996 | 0.052 |
| MCP.3 | IL-17A | 0.000 | 0.002 | 0.998 | 0.999 | 0.006 |
| STAMBP | IL-17A | 0.000 | 0.001 | 0.999 | 0.999 | 0.085 |

LogFC between groups and p-values are calculated with limma package in R.

FDR correction method is used to calculate adjusted p-values.

Significance is defined as FDR-adjusted p-value <0.05 (*)

Cohen’s d is reported to indicate the observed effect size for the respective comparison.

Models were adjusted for maternal inflammatory marker batch, child age at DBS collection, and the first two principal components (PC1 and PC2) of the collinear neonatal covariates.
